# Genomic signatures of recombination in a natural population of the bdelloid rotifer *Adineta vaga*

**DOI:** 10.1038/s41467-020-19614-y

**Published:** 2020-12-18

**Authors:** Olga A. Vakhrusheva, Elena A. Mnatsakanova, Yan R. Galimov, Tatiana V. Neretina, Evgeny S. Gerasimov, Sergey A. Naumenko, Svetlana G. Ozerova, Arthur O. Zalevsky, Irina A. Yushenova, Fernando Rodriguez, Irina R. Arkhipova, Aleksey A. Penin, Maria D. Logacheva, Georgii A. Bazykin, Alexey S. Kondrashov

**Affiliations:** 1 Skolkovo Institute of Science and Technology, Moscow, 121205 Russian Federation; 2 Department of General Ecology and Hydrobiology, Faculty of Biology, M. V. Lomonosov Moscow State University, Moscow, 119234 Russian Federation; 3 Koltzov Institute of Developmental Biology of the Russian Academy of Sciences, Moscow, 119334 Russian Federation; 4 Faculty of Biology, M. V. Lomonosov Moscow State University, Moscow, 119234 Russian Federation; 5 Institute for Information Transmission Problems of the Russian Academy of Sciences (Kharkevich Institute), Moscow, 127051 Russian Federation; 6 A. N. Belozersky Institute of Physico-Chemical Biology, M. V. Lomonosov Moscow State University, Moscow, 119992 Russian Federation; 7 Martsinovsky Institute of Medical Parasitology, Tropical and Vector-Borne Diseases, Sechenov University, Moscow, 119435 Russian Federation; 8 Department of Biostatistics, Harvard Chan School of Public Health, Boston, MA 02115 USA; 9 Faculty of Bioengineering and Bioinformatics, M. V. Lomonosov Moscow State University, Moscow, 119234 Russian Federation; 10 Shemyakin-Ovchinnikov Institute of Bioorganic Chemistry of the Russian Academy of Sciences, Moscow, 117997 Russian Federation; 11 Josephine Bay Paul Center for Comparative Molecular Biology and Evolution, Marine Biological Laboratory, Woods Hole, MA 02543 USA; 12 Department of Ecology and Evolutionary Biology, University of Michigan, Ann Arbor, MI 48109 USA; 13 Present Address: Medkvadrat, Moscow, 115409 Russian Federation

**Keywords:** Evolutionary genetics, Molecular evolution, Genetic variation, Genetics, Evolutionary biology

## Abstract

Sexual reproduction is almost ubiquitous among extant eukaryotes. As most asexual lineages are short-lived, abandoning sex is commonly regarded as an evolutionary dead end. Still, putative anciently asexual lineages challenge this view. One of the most striking examples are bdelloid rotifers, microscopic freshwater invertebrates believed to have completely abandoned sexual reproduction tens of Myr ago. Here, we compare whole genomes of 11 wild-caught individuals of the bdelloid rotifer *Adineta vaga* and present evidence that some patterns in its genetic variation are incompatible with strict clonality and lack of genetic exchange. These patterns include genotype proportions close to Hardy-Weinberg expectations within loci, lack of linkage disequilibrium between distant loci, incongruent haplotype phylogenies across the genome, and evidence for hybridization between divergent lineages. Analysis of triallelic sites independently corroborates these findings. Our results provide evidence for interindividual genetic exchange and recombination in *A. vaga*, a species previously thought to be anciently asexual.

## Abstract (web summary)

Ancient, asexual lineages are rare as a lack of recombination is usually an evolutionary dead end. Here, authors compare complete genomes of 11 individual bdelloid rotifers that suggest evidence of regular genetic exchange between individuals in a species that was previously thought to be asexual.

## Introduction

Sexual reproduction, which involves alternation of meiosis and syngamy, is the ancestral condition of extant eukaryotes. While transitions to asexuality in eukaryotes are frequent, they usually result in a quick extinction^1,2^. Still, a number of alleged ancient asexual lineages, sometimes referred to as ‘evolutionary scandals’^3,4^, challenge the indispensability of sexual reproduction for the long-term evolutionary success. The list of such lineages includes darwinulid ostracods^5,6^, oribatid mites^7^, timema stick insects^8^, and bdelloid rotifers^9–12^, the most prominent of these groups, that underwent an extensive adaptive radiation after presumably losing sex tens of millions of years ago.

The main reason to assume that bdelloids lack meiotic sex is the fact that not a single male has ever been conclusively identified in them, despite hundreds of thousands of bdelloid individuals examined by many researchers^10^. In contrast, the available data on bdelloid genomes are ambiguous. The initial analysis of the first genome sequence of a laboratory strain of a bdelloid rotifer *Adineta vaga* failed to detect homologous chromosomes^11^, which is hardly compatible with conventional meiosis. This finding, however, was not confirmed by sequencing of the genome of a closely related species *A. ricciae*, which did not reveal any rearrangements that would preclude chromosome pairing^13^.

A separate line of genomic evidence for or against long-term asexuality can in principle be obtained from the degree of interallelic divergence. Lack of recombination between homologous chromosomes in asexuals is expected to lead to a gradual accumulation of differences between the two alleles^14^ at a locus, resulting in high interallelic divergence (‘Meselson effect’^14^). However, sequenced genomes of bdelloids were found to be very different in terms of such divergence, with the estimates ranging from ~0.03% to ~5% in different species^13^. Therefore, patterns of genomic organization observed in bdelloid rotifers do not provide conclusive evidence for sexuality or asexuality in this group.

Failure to find males in bdelloids does not exclude the possibility of cryptic sexual reproduction or other forms of interindividual genetic exchange and recombination in their populations^15–18^. Indeed, recent analyses based on several genomic regions suggested genetic exchanges in this group^15,16^. However, whole-genome evidence for recombination in bdelloids has been lacking. Moreover, the findings of ref. ^16^ have lately been explained by experimental artifacts arising from accidental contamination^17^. Therefore, it remains unclear if bdelloids regularly engage in any form of genetic exchange.

Here, we study variation in a wild population of *A. vaga* at the whole-genome level. A number of patterns in this variation, both within and between individual loci, suggest that interindividual genetic exchange and recombination regularly occur in this species. We conclude that *A. vaga* cannot be a strictly clonal species evolving in the absence of genetic exchange.

## Results

### Population genomics of *Adineta vaga*

**Fig. 1:**
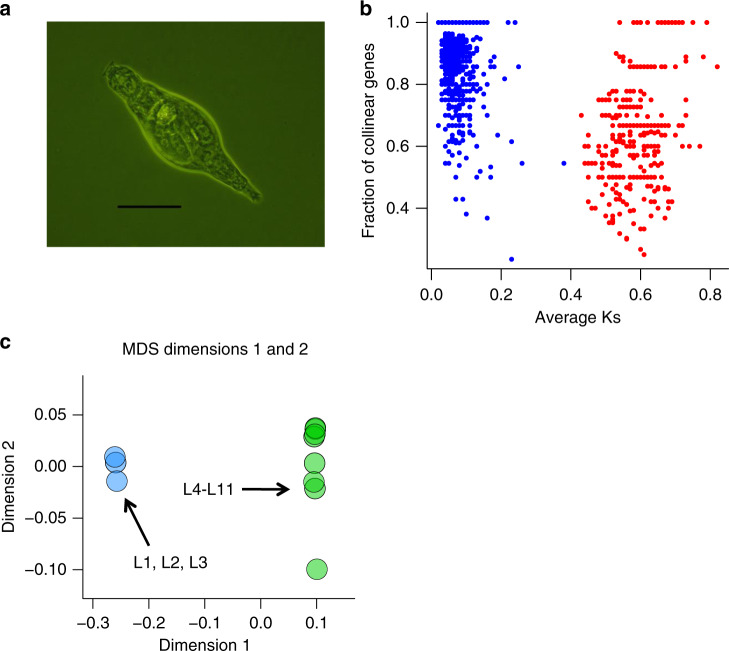
Whole-genome sequencing of 11 wild-caught individuals of the bdelloid rotifer *A. vaga*. **a** Microphotograph of an *A. vaga* individual. Scale bar is approximately 100 μm, based on the characteristic size of *A. vaga* mastax^51^. The photograph was taken for the illustrative purposes. From each of the 11 wild-caught rotifers matching the morphological description of *A. vaga*, a clonal lineage has been established and sequenced on the Illumina HiSeq to the coverage of ~40–100×. Rotifers were collected in one of the two locations: the Moscow region of Russia or the Kostroma region of Russia, 550 km to NE. All sequenced clonal lineages collected in the same location were started from individuals sampled from separate trees at least 20 m apart. **b** Tetraploidy in the *A. vaga* L1 reference genome. The plot shows the mean number of synonymous substitutions between collinear genes per site (Ks) versus the fraction of collinear genes for each syntenic block. Only blocks with 5 or more collinear genes are shown (*n* = 1769). Blocks with average Ks values <0.4 are in blue; those with average Ks ≥ 0.4 in red (see Supplementary Methods). **c** Genetic differentiation among the 11 sequenced individuals L1-L11. The first two dimensions from multidimensional scaling (MDS) analysis of pairwise identity-by-state distances are shown. Individuals of the small cluster (L1-L3) and of the large cluster (L4-L11) are shown in blue and green respectively (see Supplementary Methods). Clustering does not reflect geography of sampling: the small cluster is comprised of three individuals (L1-L3) collected in the Moscow region of Russia, while the large cluster contains the rest of individuals collected in the Moscow region (L4, L6-L10), as well as two individuals (L5 and L11) collected in the Kostroma region. See also Supplementary Fig. 8 for a SNP-based neighbor-joining tree of L1-L11.

To elucidate the mode of reproduction of bdelloids, we sequenced genomes of 11 wild-caught *A. vaga* individuals (Fig. 1a). For this, we established 11 clonal lineages, L1-L11, each started from a single rotifer matching the morphological criteria of *A. vaga* (Supplementary Table 1). We confirmed species identity of these individuals using mitochondrial marker-based phylogeny; while the samples clustered into distinct clades (see below), they all were genetically most similar to *A. vaga* (Supplementary Note 1; Supplementary Figs. 1–3). Each sample was sequenced on Illumina HiSeq to the coverage of ~40–100× (see “Methods” section; Supplementary Data 1 and Supplementary Table 2). In addition, we sequenced one of the lineages, L1, on the MiSeq platform, which allowed us to generate a de novo genome assembly for this lineage (see “Methods” section; Supplementary Tables 3, 4; Supplementary Figs. 4, 5). In terms of completeness, the obtained assembly carries ~90% of complete nearly universal eukaryotic and metazoan single-copy orthologs^19^, closely resembling the previously published bdelloid genomes of *A. vaga*^11^ and *A. ricciae*^13^ (Supplementary Figs. 6, 7; Supplementary Methods).

The analysis of the L1 assembly, hereafter referred to as the reference genome, revealed the same patterns of tetraploidy as those in the previously published genome of *A. vaga*^11^. Specifically, a large number of genomic segments could be assigned into pairs of collinear allelic regions representing two haplotypes, and into more distantly related clusters that probably arose from an ancient whole-genome duplication (Fig. 1b). We obtained a non-redundant haploid representation of the *A. vaga* L1 genome and mapped all the sequenced individual genomes against it (see “Methods” section; Supplementary Table 3).

Analysis of single-nucleotide differences between the sequenced individuals revealed presence of two genetic clusters (Fig. 1c and Supplementary Fig. 8; Supplementary Methods; Supplementary Tables 5–7). The average pairwise genotypic distance was 1.22% for individuals belonging to different clusters, 0.66% for the 3 individuals belonging to the small cluster (L1-L3), and 0.54% for the 8 individuals belonging to the large cluster (L4-L11; Supplementary Table 7). Individuals from the small and the large clusters exhibit notable difference in the levels of intraindividual heterozygosity: the average genome-wide fraction of heterozygous sites per individual is 1.98% for the small cluster but only 0.63% for the large cluster (Supplementary Data 2 and Supplementary Table 8; Supplementary Fig. 9; Supplementary Methods). The corresponding values for silent (four-fold degenerate) sites of the protein-coding regions are 3.75% and 1.21% respectively (Supplementary Data 2; Supplementary Discussion). To reduce the potential effect of population structure, we focused most of the subsequent analysis on the 8 individuals (L4-L11) forming the large cluster.

### Signatures of recombination in *A. vaga* genomes

In obligate asexuals, all genomic loci are linked, and the extent of linkage disequilibrium (LD) is not expected to depend on the physical distance separating loci (Supplementary Fig. 10). In contrast, in a sexual population, LD decays with increasing distance between loci^20^, which constitutes a prominent signature of recombination. We investigated the patterns of LD in the population of *A. vaga* on a genome-wide scale (see “Methods” section). For each individual, we reconstructed the haplotypes by read-based phasing^21^ (see “Methods” section; Supplementary Tables 9, 10). We obtained two sets of aggressively filtered phased haplotype blocks: phased dataset 1 which was used as the main dataset, and the auxiliary phased dataset 2 subjected to even more stringent filtering. Both datasets were filtered based on the presence of conflicting haplotype evidence in aligned reads, and the phased dataset 2 was further filtered using estimated probabilities of phasing errors^21^ (see “Methods” section).

**Fig. 2:**
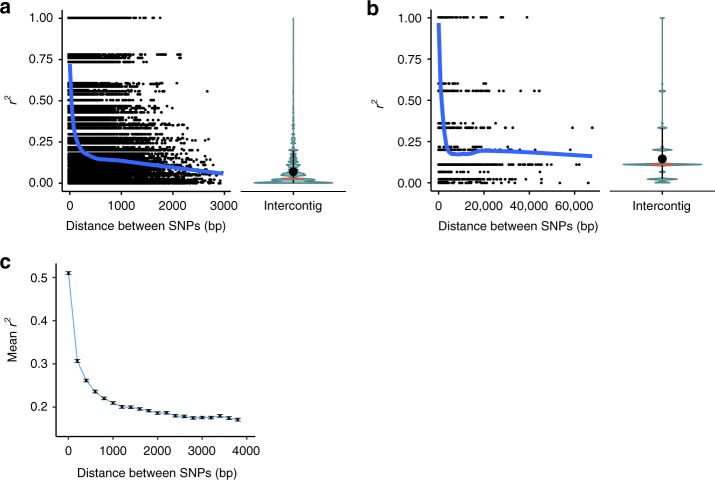
Decay of linkage disequilibrium (LD) with physical distance in *A. vaga*. **a**, **b** LD is measured as *r*^2^. Decay of *r*^2^ with physical distance is estimated using phased haplotype data (**a**) and unphased genotype data (**b**). Second-degree LOESS regression curves of *r*^2^ versus physical distance (smoothing parameter set to 0.4) are shown in blue. Violin plots show the distributions of *r*^2^ values for the pairs of SNPs located on different contigs. Ends of the whiskers represent the 10–90th percentile range, with the mean and median values shown as a black dot and a red horizontal bar, respectively. **a** *r*^2^ was calculated using biallelic sites residing within the segments of the reference genome where haplotypes had been reconstructed for all the individuals forming the large cluster (L4-L11). See also Supplementary Fig. 11. **b** Estimates of *r*^2^ based on the unphased genotype data were obtained using biallelic sites homozygous in all genomes forming the large cluster. **c** LD is expressed as the squared correlation coefficient between genotypes. Squared correlation coefficients were computed for comparisons of 10,000 randomly drawn biallelic sites versus the remaining biallelic sites (minor allele count in L4-L11 ≥ 4). SNP pairs were subdivided by distance into bins of 200 bp; the displayed data are for the bins with pairs of SNPs separated by ≤4000 bp. Black dots show the mean values of squared correlation coefficient between genotypes for different distance bins (*x*-coordinates correspond to the left edges of bins). Error bars indicate 95% bootstrap confidence intervals based on 1000 replicates within each bin. Source data are provided as a Source Data file (**c**).

Contrary to what would be expected if *A. vaga* were strictly asexual, we found that among the individuals L4-L11, the LD within genome segments from the phased dataset 1 rapidly declined with the distance between polymorphic loci (Fig. 2a and Supplementary Fig. 11), reaching the mean level observed for different contigs at ~2600–2700 nucleotides. This decay of LD is within the range observed in strictly sexual species: similar to that in *Drosophila melanogaster*^22,23^ and faster than in the human genome^24^.

Next, we tested for recombination on a per-segment basis, both assessing correlation of *r*^2^ with distance within individual segments^25^ and applying common tests for recombination (sum of the distances^26^ and PHI tests^27^) to each segment independently (see “Methods” section). All these analyses also suggest that recombination is present. Specifically, out of 434 segments, 362 demonstrated significant negative correlation of *r*^2^ with the physical distance at the 0.05 significance level (with 159 remaining significant after correcting for multiple testing). According to the sum of the distances and the PHI tests, recombination was detected in 108 and 190 segments out of 434 (*P* < 0.05 after the Bonferroni correction; Supplementary Fig. 12).

Even in the absence of recombination, the observed LD decay (assessed through decline in *r*^2^, as well as through other tests) could arise artefactually. First, it might be caused by erroneous mapping of reads to paralogous regions. However, the decay persists in subsets of polymorphic loci covered by blocks of collinear genes^28^ (Supplementary Fig. 13a), making this explanation unlikely. Existence of two haplotypes in the L1 genome in these subsets of loci is additionally confirmed by the presence of highly similar genes collinear between the two putative haplotypes.

Second, LD decay could appear due to phasing errors, including those arising from PCR template switching^29^. We assessed the accuracy of phasing by comparing the haplotype phases inferred for the same individual from different sets of reads (Supplementary Note 2). Namely, we contrasted haplotypes recovered from Illumina HiSeq reads to those assembled using PacBio (for individual L1) and Illumina MiSeq reads (for individuals L1, L2, and L11). We showed that the incidence of putative switch errors assessed by identifying inconsistencies between haplotype phases determined for single nucleotide polymorphisms (SNPs) using different sets of reads is low: estimates of the fraction of contigs (among those harboring phased haplotype blocks) with switch errors for phased datasets 1 and 2 are below 2% and 1%, respectively (Supplementary Data 3). As expected, segments from the more stringently filtered phased dataset 2 displayed higher accuracy of phasing than segments from the dataset 1 (Supplementary Note 2; Supplementary Data 3). Importantly, although the segments from the phased dataset 1 displayed an increase in the fraction of inconsistently phased SNP pairs with distance, which could potentially be mistaken for LD decay when different individuals are considered, there was no such trend for the phased dataset 2 (Supplementary Fig. 14). We repeated the LD analysis among L4-L11 individuals based on the phased dataset 2, which recapitulated the decay of LD, indicating that the signal of LD decay is independent of the severity of filtering (Supplementary Fig. 13b).

To further ensure that the decay of LD is not explained by phasing errors, we independently assessed it from unphased genotype data using two different approaches: inferring haplotypes from variable homozygous sites (Fig. 2b) or estimating LD directly from correlations between genotypes (Fig. 2c; Supplementary Methods). LD decay was observed in both these analyses. As these approaches do not rely on phasing, the observed decline in LD with distance between sites is not due to phasing errors.

As an alternative approach to assess LD, we additionally estimated correlation in zygosity between pairs of sites within individual genomes at different distances using the maximum likelihood method^30,31^. This analysis, which employs an individual-based measure of LD and does not depend on phasing, also revealed a decline in LD with distance (Supplementary Fig. 15; Supplementary Methods). Together, these findings rule out phasing errors as an explanation for the observed LD decay.

### Signatures of reciprocal recombination in *A. vaga*

Even in the absence of phasing artifacts, LD decay could potentially arise without reciprocal recombination due to gene conversion between allelic regions. Gene conversion is a non-reciprocal process of DNA transfer between homologous chromosomes which leads to copying of a DNA segment from one homologous chromosome onto the other^32–34^; importantly, it can result from resolution of both crossover and non-crossover events^32^. Therefore, although signatures of gene conversion are frequently associated with reciprocal recombination, they do not necessarily imply it. Gene conversion has been previously proposed to inflate the rate of LD decay between tightly linked loci in humans^35,36^, and it has been suggested to act within diploid loci of a single *A. vaga* individual^11^.

**Fig. 3:**
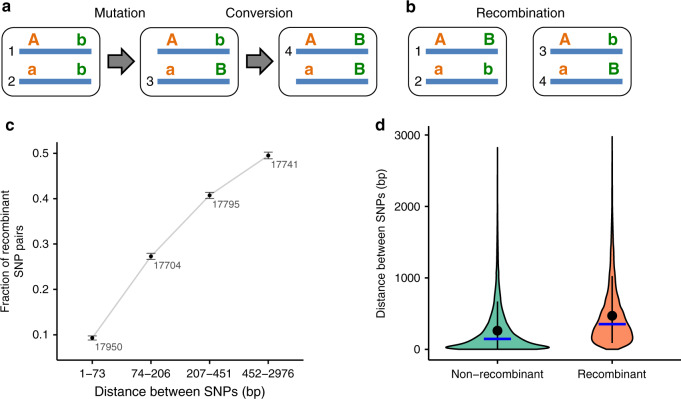
Modified four-gamete test suggests reciprocal recombination in *A. vaga*. **a** Emergence of four haplotypes in the absence of genetic exchange and reciprocal recombination due to mutation and conversion. Boxes represent individuals. **b** Schematic representation of a recombinant pair of sites which could not be the result of gene conversion. For a pair of SNPs, all four haplotypes are present in two individuals. Such pairs are regarded as passing the modified four-gamete test. **c** Dependence of the fraction of SNP pairs passing the modified four-gamete test on the physical distance between the SNPs in a pair. For each pairwise combination of individuals L4-L11, only those pairs of polymorphic sites that are simultaneously heterozygous in both individuals are considered. SNP pairs meeting the requirements of the modified four-gamete test were subdivided into 4 distance bins with approximately equal numbers of cases. Black dots show the fractions of recombinant SNP pairs for different distance bins. The total number of analyzed SNP pairs for each bin is shown next to the corresponding dot. Error bars indicate 95% bootstrap confidence intervals based on 1000 replicates within each bin. Comparisons between all bin pairs were significant (two-sided *P* < 6 × 10^−4^, permutation test). Note that distances between bins on the *x*-axis are not to scale. **d** Violin plot showing the distribution of distances between SNPs in a pair for non-recombinant (*n* = 48,657) and recombinant (*n* = 22,533) pairs of SNPs. Whiskers indicate the 10–90th percentile range; mean and median distances for each group are shown with a black dot and a blue horizontal bar, respectively. Mean = 261.7 and median = 146 base pairs, non-recombinant; 470.6 and 353, recombinant. Difference in the mean distances between SNPs in non-recombinant and recombinant pairs was significant (two-sided *P* < 1 × 10^−4^, permutation test). Source data are provided as a Source Data file (**c**, **d**).

To systematically check whether the distribution of SNPs across haplotypes could be ascribed solely to the action of gene conversion, we employed a modified version of the Hudson’s four-gamete test^37^. In the classical Hudson’s four-gamete test^37^, the presence of all four possible types of haplotypes for a pair of biallelic polymorphic loci within a population is interpreted as evidence for reciprocal recombination, because recurrent mutations are rare. However, a mutation followed by gene conversion would suffice to explain the presence of all four haplotypes without assuming reciprocal genetic exchange between homologous regions (Fig. 3a). Nevertheless, the action of within-locus gene conversion can only produce a homozygous genotype from a heterozygous one, but not vice versa. Therefore, it cannot produce a pair of individuals, each heterozygous at two loci, carrying all four haplotypes (Fig. 3b; Supplementary Note 3); while such a pair can obviously arise through reciprocal recombination (or, in principle, through non-reciprocal recombination during transformation). We use this logic to perform the modified four-gamete test, in which a signal cannot be driven by gene conversion alone: we look for pairs of SNPs simultaneously heterozygous in two individuals and represented in these two individuals by all four haplotypes, referred to as ‘recombinant’ pairs. We find that among the pairs of SNPs each heterozygous in two individuals, the fraction of those giving rise to all four possible haplotypes in these individuals is low when these SNPs are positioned nearby, but increases rapidly with the physical distance between SNPs (Fig. 3c, d; Supplementary Fig. 16; Supplementary Note 3). Importantly, although recurrent mutations can give rise to pairs of recombinant SNPs passing this modified four-gamete test, the fraction of such pairs resulting from recurrent mutations is not expected to increase with physical distance. Hence, if not explained by phasing artifacts, this observation is incompatible with the action of gene conversion as the sole cause of the LD decay.

To see whether this analysis is likely to be significantly affected by phasing errors, we applied the modified four-gamete test to two pairs of individuals for which more than one phased dataset was available (L2-L1 and L11-L1) and compared its results to the results of the four-gamete test applied to different phased datasets obtained for the same individual (Supplementary Note 2). When analyzing two different individuals, we expect to detect recombinant pairs of SNPs stemming not only from phasing errors but also from true recombination events (if any). Indeed, as expected from true recombination, the fraction of recombinant SNP pairs inferred from comparison of different individuals is two orders of magnitude or more higher than that of the same individual using different data (of the order of 10^−3^ or less; Supplementary Note 2 and Supplementary Figs. 17, 18).

Thus, our findings suggest reciprocal recombination in *A. vaga* (Supplementary Note 4; Supplementary Fig. 19). However, the observed patterns are compatible not only with recombination accompanied by interindividual genetic exchanges, but also with reciprocal mitotic recombination not followed by any form of DNA transfer^38^.

### Signatures of genetic exchange between individuals

**Fig. 4:**
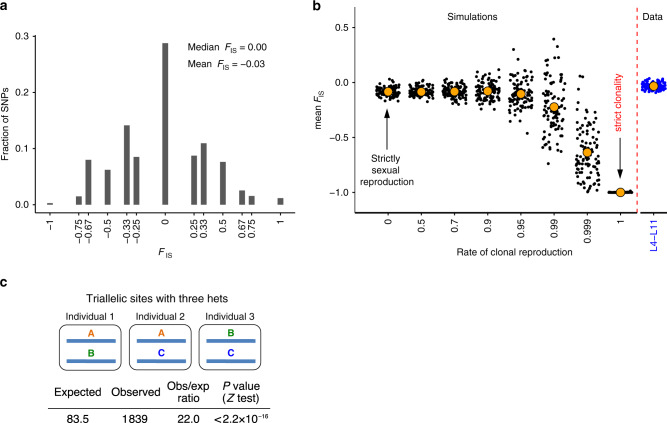
Analysis of biallelic and triallelic sites suggests genetic exchange in *A. vaga*. **a** Distribution of values of inbreeding coefficient (*F*_IS_) for the sites biallelic in individuals L4-L11 (minor allele count ≥4, *n* = 440,564). Under Hardy-Weinberg equilibrium *F*_IS_ is 0, negative and positive values of *F*_IS_ indicate excess of heterozygotes and homozygotes, respectively. **b** Values of *F*_IS_ observed in L4-L11 versus expected under different rates of clonal reproduction. Plot shows mean *F*_IS_ values computed for random samples of SNPs (*n* = 200) drawn from the data (blue dots) or populations simulated in SLiM^41^ with different rates of clonal reproduction (black dots). Parameters were chosen so that the population-scaled mutation rate 4*N*_e_*μ* was close to that estimated from the data (Supplementary Table 8): *N*_e_ = 2500, *μ* = 10^−6^. All simulations were run for 200,000 generations and replicated 100 times. For each replicate of each simulation, we randomly chose 8 individuals (matching the number of the analyzed individuals L4-L11), retained only those SNPs that had minor allele count ≥4 and then randomly sampled 200 SNPs. This yielded a total of 100 sets of SNPs per a simulation. Individual black dots represent mean *F*_IS_ among SNPs in each such set. To allow comparison with the data, we analogously computed mean *F*_IS_ for 100 random sets of SNPs (*n* = 200) biallelic among L4-L11 (minor allele count ≥4). Mean *F*_IS_ values for each such set are shown with blue dots. Orange circles represent the average of mean *F*_IS_ values across all SNP sets for each simulation or across all SNP sets drawn from the data. **c** A schematic representation of a triallelic site with alleles A, B, and C and all three their possible heterozygous combinations (abbreviated as ‘hets’ in the Figure) present among the sequenced individuals. The bottom part of the panel shows observed and expected numbers of such sites among L4-L11. Source data are provided as a Source Data file (**a**, **b**).

To address the possibility that the inferred recombination is not associated with any transfer of genetic material between individuals, we analyzed the patterns of variation within individual biallelic SNPs. Under obligate asexual reproduction, two alleles at a locus accumulate mutations independently of each other. In a finite population, this creates a major excess of heterozygotes relative to the Hardy-Weinberg expectation^39,40^, leading to negative values of the inbreeding coefficient *F*_IS_. While the expected value of *F*_IS_ under the Hardy-Weinberg equilibrium (HWE) is 0, under strict clonality, its expected value is −1. We analyzed the distribution of *F*_IS_ for the sites biallelic among the L4-L11 individuals and found that the distribution of *F*_IS_ values was centered around 0 (median = 0.0, mean = −0.03; Fig. 4a; Supplementary Table 11; Supplementary Fig. 20), suggesting that the population is close to HWE. Characteristic *F*_IS_ values for the genomic regions with high-confidence ploidy were similar (mean = −0.03 and −0.04 for allelic regions and allelic genes, respectively, median = 0.0 in both cases; Supplementary Table 11). To explicitly assess what values of *F*_IS_ are expected under different rates of clonal reproduction within the analyzed sample of 8 individuals, we conducted individual-based simulations^41^ of populations ranging the cloning rate from 0 (strictly sexual reproduction) to 1 (strictly clonal reproduction), subsampling the resulting populations by randomly drawing 8 individuals and assessing the distribution of *F*_IS_ values in the simulated datasets (see “Methods” section). In line with previous results^39^, we find that it takes very little sexual reproduction to achieve mean *F*_IS_ values close to those expected in strictly sexual populations: upwards of ~1% of sexual reproduction, *F*_IS_ is similar to that in populations propagating exclusively sexually^39^ (Fig. 4b). Importantly, the *F*_IS_ values observed in L4-L11 are significantly higher than those expected under strict clonality (cloning rate = 1.0, *P* value < 0.01) or very rare sexual reproduction (cloning rate = 0.999, *P* = 0.03); however, they are consistent with any among the simulated scenarios with the rate of sexual reproduction ≥1% (Fig. 4b; see “Methods” section).

Conceivably, consistency with Hardy–Weinberg expectations could be achieved even in a strictly clonal population due to interplay between mutation and within-locus gene conversion (Supplementary Note 5). However, the conditions for that are extremely restrictive: for a particular set of parameters characterizing mutation and random drift, gene conversion must occur at a precisely specified rate. Specifically, we have shown that with realistic mutation rates, for a clonal population to be at HWE, gene conversion should occur at a rate $\alpha \approx \frac{1}{{2N_{\rm{e}}}}$, where *N*_e_ is the effective population size (Supplementary Note 5). Moreover, slight deviations of *α* from this equilibrium value will significantly move the population away from HWE (Supplementary Note 5; Supplementary Figs. 21–23). Because such fine-tuning of unrelated processes is unlikely, the observed proportions of genotypes found to be overall close to those expected under HWE (mean *F*_IS_ close to zero) argue against strict clonality and suggest that genetic exchanges occur in *A. vaga*.

Independent evidence for genetic exchange between individuals is provided by triallelic SNPs^42^ carrying three out of four possible nucleotides. Triallelic SNPs can arise in sexual, as well as in asexual organisms through multiple mutations affecting the same genomic site. At a polymorphic site with three alleles A, B, and C, three heterozygous genotypes, A/B, A/C, and B/C, are possible (Fig. 4c). Genetic exchanges between individuals will lead to frequent coexistence of all these genotypes. By contrast, in a population of asexuals, all three heterozygous genotypes can only arise through recurrent or back mutations, which are relatively rare.

Indeed, a triallelic SNP can only originate through at least one mutation at a biallelic site. The probability of such a mutation in the history of a sample of genotypes can be estimated as P3 = N3/(N2 + N3), where N2 and N3 stand for the numbers of sites with two and three alleles, respectively. In the absence of genetic exchange, the emergence of a triallelic site carrying all three possible heterozygotes would require yet another mutation, so that the expected number of triallelic SNPs with three heterozygotes is P3 × N3. In our data, the resulting expected number of triallelic SNPs carrying three heterozygotes is 83.5, but in fact we identified 1839 such SNPs (*P* < 2.2 × 10^−16^, one-sample *Z*-test; Supplementary Data 4; Supplementary Note 6; Supplementary Fig. 24), which constitutes a 22-fold enrichment. A similar excess was observed in the genomic regions with high-confidence ploidy (Supplementary Data 4).

To ensure that triallelic sites with three heterozygotes are not likely to stem from cross-sample contamination, we separately considered those sites carrying all three heterozygotes among the individuals L4-L11 where the least frequent heterozygous genotype was present only in a single individual (*n* = 607). We assessed how such private heterozygous genotypes were distributed among different individuals. If L4-L11 were in fact clonal, but a fraction of samples were contaminated, we would expect those samples to harbor the majority of private heterozygous genotypes. However, private heterozygous genotypes were distributed almost uniformly among the individuals L4-L11, with different individuals possessing similar numbers of such genotypes (average number per individual 75.9, with the minimum and maximum values of 64 and 88 sites, respectively; Supplementary Table 12; Supplementary Note 6). This argues against contamination as the source of sites harboring all three heterozygotes and lends support to genetic exchange as the main mechanism of their emergence. Analysis of mitochondrial variation in full mitogenomes also argues against contamination between the sequenced cultures (Supplementary Notes 7, 8 and Supplementary Tables 13–18).

### Distinguishing between mechanisms of genetic exchange

Our data suggest that variation in *A. vaga* was shaped by recombination, and is consistent with genetic exchanges between individuals. Still, the observed patterns could emerge through at least two mechanisms of genetic exchange: horizontal gene transfer (HGT), likely as a result of transformation, and meiotic sex. HGT is supported by widespread incorporation of genes from a variety of non-metazoan species into bdelloid genomes^13,43,44^, which demonstrates their intrinsic propensity for acquiring foreign DNA. Meiotic sex can involve either conventional meiosis or the so-called *Oenothera*-like meiosis, a highly atypical version of meiosis observed in a few plants. This kind of meiosis involves segregation without pairing between homologous chromosomes and mostly without crossing over^15^. The *Oenothera*-like meiosis was suggested as a mode of genetic exchange for the bdelloid species *Macrotrachela quadricornifera* based on the observed pattern of allele sharing for several genomic regions in three individuals^15^.

In contrast to strict clonality, any form of genetic exchange is expected to result in incongruent phylogenies of the two haplotypes of an individual^15^ (Supplementary Fig. 25). However, the way in which this incongruence manifests at different loci can be utilized to discriminate between *Oenothera*-like meiosis and the other two possibilities. The key feature of *Oenothera*-like meiosis is the presence of two haplotype complexes, such that all chromosomes belonging to the same complex are always inherited together^15^. Therefore, the pattern of incongruence should be identical across the whole genome^15^. By contrast, both conventional meiosis and HGT should create different patterns of incongruence between haplotypes at different loci^15^.

**Fig. 5:**
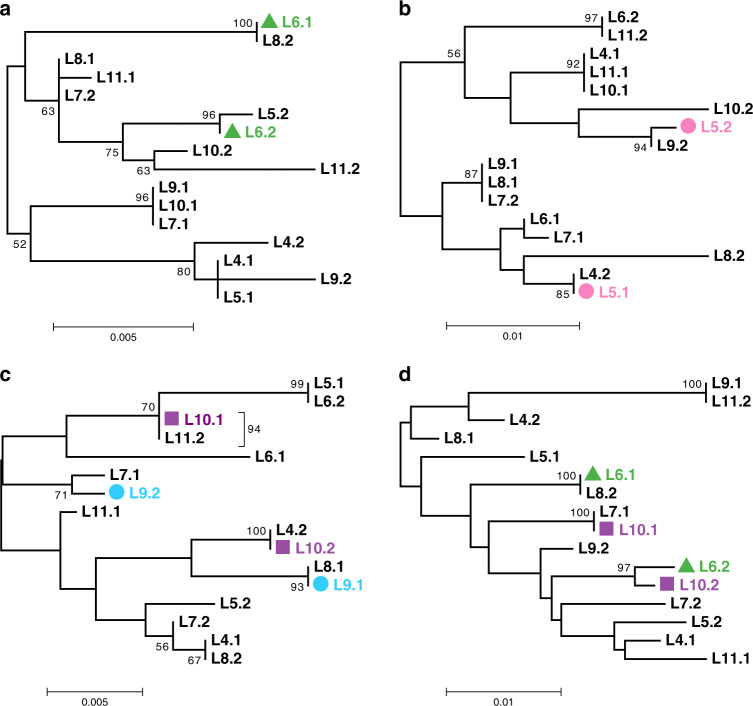
Phylogenetics of L4-L11 haplotypes indicates interindividual genetic exchange. Midpoint-rooted phylogenetic trees constructed for four phased segments exhibiting incongruent groupings of individuals with respect to two haplotypes. Overall, in 52 out of 303 tested segments, two haplotypes of at least one individual had their reciprocal closest counterparts in two different individuals (see “Methods” section and Table 1). The displayed phylogenies were constructed for four phased segments located on different contigs in the L1 diploid assembly and spanning 896 (**a**), 604 (**b**), 1348 (**c**), and 1198 (**d**) base pairs. Trees were built using the maximum likelihood method in PhyML^74^ under the GTR + G model with 1000 bootstrap replicates. The bootstrap support values ≥50% are shown next to branches. Bootstrap support for the grouping of L10.1 and L11.2 in the panel **c** is shown next to the bracket. Bootstrap support values are rounded to the nearest integer. Only those SNPs that satisfied all filtering criteria and were simultaneously phased in L4-L11 were used to reconstruct phylogenies (*n* = 23, 21, 33, and 57 non-singleton SNPs in **a**–**d**, respectively); the remaining sites were treated as monomorphic (see “Methods” section). Indices 1 and 2 designate the two haplotypes of a single individual. In these four segments, the two haplotypes of the same individual clustered with haplotypes from two different individuals with high bootstrap support values (≥70%) for L5 (**b**; pink), L6 (**a**, **d**; green), L9 (**c**; blue), and L10 (**c**, **d**; violet). Source data are provided as a Source Data file.

We compared phylogenies of the two haplotypes harbored by each individual at different genomic loci (see “Methods” section). We observed multiple cases when the two haplotypes of a single individual clustered with haplotypes from different individuals (Table 1; Fig. 5). Cases of incongruence were detected in all individuals L4-L11 and supported different patterns of clustering of the two haplotypes of the same individual at different loci (Table 1; Fig. 5; Supplementary Data 5 and Supplementary Table 19; Supplementary Discussion). We recapitulated the results of this analysis using haplotypes reconstructed based on the set of SNPs additionally filtered to account for potential artifacts of index hopping and heterozygote undercalling (see “Methods” section; Supplementary Table 20), indicating that the incongruence is not a result of either of these processes. The finding of multiple patterns of incongruence between the two haplotypes for each individual lends further support to genetic exchanges in *A. vaga*, and excludes *Oenothera*-like meiosis as its sole cause (Supplementary Discussion and Supplementary Fig. 26). Both HGT and conventional meiosis can explain the data more easily than atypical meiosis.

**Table 1:** Incongruent groupings of the two haplotypes in individuals L4-L11.

Individual	Analyzed phased segments	Incongruent phased segments	Different patterns of incongruence	Observed patterns of incongruence
L4	303	9	7	L5-L9 (1), L5-L11 (1), L6-L7 (1), L6-L8 (1), L6-L11 (3), L7-L11 (1), L10-L11 (1)
L5	303	9	8	L4-L7 (1), L4-L9 (1), L4-L10 (2), L6-L9 (1), L6-L10 (1), L6-L11 (1), L7-L11 (1), L9-L10 (1)
L6	303	13	9	L4-L10 (1), L5-L8 (2), L5-L9 (1), L7-L10 (2), L7-L11 (1), L8-L9 (1), L8-L10 (1), L8-L11 (3), L9-L11 (1)
L7	303	4	2	L4-L8 (1), L5-L9 (3)
L8	303	11	8	L4-L5 (1), L4-L7 (1), L5-L7 (1), L5-L10 (1), L6-L7 (1), L6-L9 (2), L6-L11 (3), L9-L10 (1)
L9	303	5	5	L4-L8 (1), L5-L10 (1), L5-L11 (1), L6-L8 (1), L7-L8 (1)
L10	303	7	6	L4-L9 (1), L4-L11 (2), L5-L6 (1), L6-L7 (1), L7-L8 (1), L7-L11 (1)
L11	303	7	6	L4-L7 (1), L5-L6 (1), L5-L8 (2), L6-L10 (1), L7-L8 (1), L7-L9 (1)

For each individual, we computed the number of phased segments such that the reciprocal closest counterparts of the two haplotypes were found in two different individuals (see “Methods” section). Only incongruent groupings of the two haplotypes with strong bootstrap support (≥70%) were considered. The numbers of identified incongruent segments along with the numbers of different patterns of incongruence observed for each individual, L4-L11, are shown. For each individual, each unique pair of other individuals harboring reciprocal closest counterparts of its two haplotypes at least at one locus constitutes a separate pattern of incongruence. The patterns of incongruence observed for each individual are listed, with the number of segments for which each pattern was observed given in parentheses. For this analysis, we used the segments of the *A. vaga* genome harboring at least 15 non-singleton SNPs simultaneously phased in all individuals L4-L11. Segments with more than two highly similar hits (≥90% identity) in the L1 genome, as well as segments harboring multiple paralogous regions were removed from this analysis. In total, out of the 303 analyzed phased genomic segments, 52 exhibited incongruent groupings of the two haplotypes at least in one individual with strong bootstrap support. Overall numbers of occurrences for each pattern of incongruence listed in the rightmost column are shown in Supplementary Table 19.

**Fig. 6:**
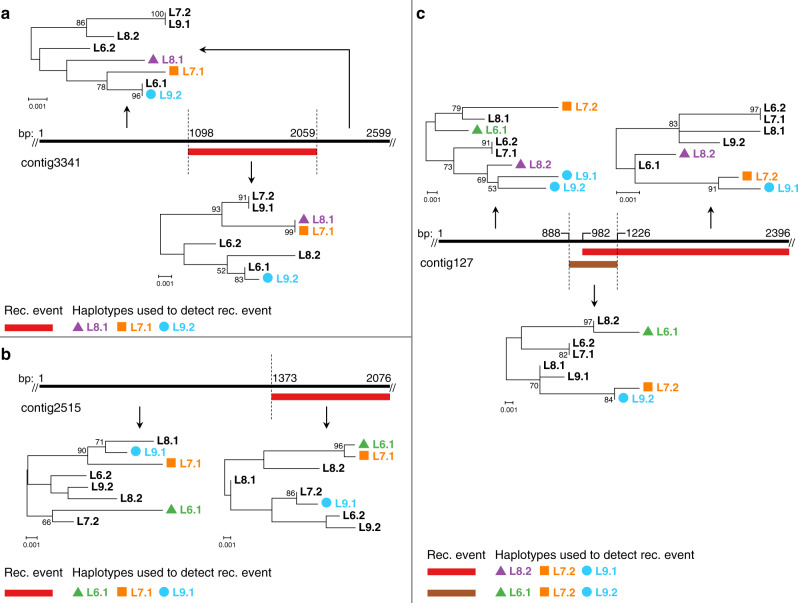
Examples of genomic regions suggestive of recombination events. Schematic representation of three genomic segments (black bars) with recombination events (colored bars) inferred according to different methods implemented in RDP4 (ref. ^77^). Each genomic segment was phased in four individuals (phased dataset 2, individuals L6-L9; see “Methods” section). The three shown segments were positioned on different contigs in the L1 diploid assembly. Numbers adjacent to the black bars indicate the coordinates of the segment start, end, and inferred breakpoints (ends of colored rectangles). Fragments delineated by boundaries of recombination events (dashed lines) were used to construct corresponding phylogenetic trees (connected with arrows to them). In phylogenies, haplotypes used by RDP4 to identify a recombination event are identified with colored shapes, with indices 1 and 2 designating the two haplotypes of a single individual. Trees were built using the maximum likelihood method in PhyML^74^ under the GTR + G model with 1000 bootstrap replicates and midpoint-rooted. The bootstrap support values ≥50% are shown next to branches. Bootstrap support values are rounded to the nearest integer. Only those SNPs that satisfied all filtering criteria and were simultaneously phased in L6-L9 were used in the analysis. For further information on the shown segments and inferred recombination events (including RDP4 *P* values), see Supplementary Data 6 and “Methods” section. Haplotypes reconstructed for L6-L9 in these three segments are provided as Supplementary Data 7.

We also studied some local examples of putative past recombination events that were identified using stringently filtered haplotypes of individuals L6-L9. Schematics of three such cases are depicted in Fig. 6. Although providing only anecdotal evidence, the patterns of haplotype phylogenies observed in adjacent regions of *A. vaga* genomes delineated by putative recombination breakpoints are also suggestive of genetic exchange (Fig. 6; Supplementary Data 6, 7).

We sought to characterize genomic segments exhibiting incongruence between the two haplotypes. A large fraction of incongruent segments had a substantial overlap with genes (0.77); however, this fraction was not significantly different from that among the subset of analyzed segments remaining after removal of incongruent ones (*P* = 0.9962; Supplementary Note 9). Usually, a segment inferred to be incongruent harbored variants falling into several functional categories including synonymous, missense, intronic or intergenic variants (Supplementary Data 8). These findings argue against convergent mutations independently acquired and fixed by positive selection in multiple lineages as the explanation for the haplotype incongruence in *A. vaga*.

The patterns of incongruence in the large cluster (L4-L11) are not sufficient to distinguish between HGT and conventional meiosis. However, haplotype phylogenies for all 11 individuals from both clusters (L1-L11), based on the genomic segments where haplotypes were reconstructed for all these individuals, can shed some light on this issue. Although the total number of such segments was low (*n* = 152), we detected several cases (*n* = 11) of well-supported incongruent grouping of haplotypes for individuals from the small cluster L1-L3 (Supplementary Table 21; see “Methods” section). Intriguingly, in all such cases when the two haplotypes (H1 and H2) of an individual from the small cluster had unambiguous closest counterparts in different individuals, one haplotype (H1) always clustered with a haplotype from another individual from the small cluster, and the other haplotype (H2), with a haplotype from the large cluster.

**Fig. 7:**
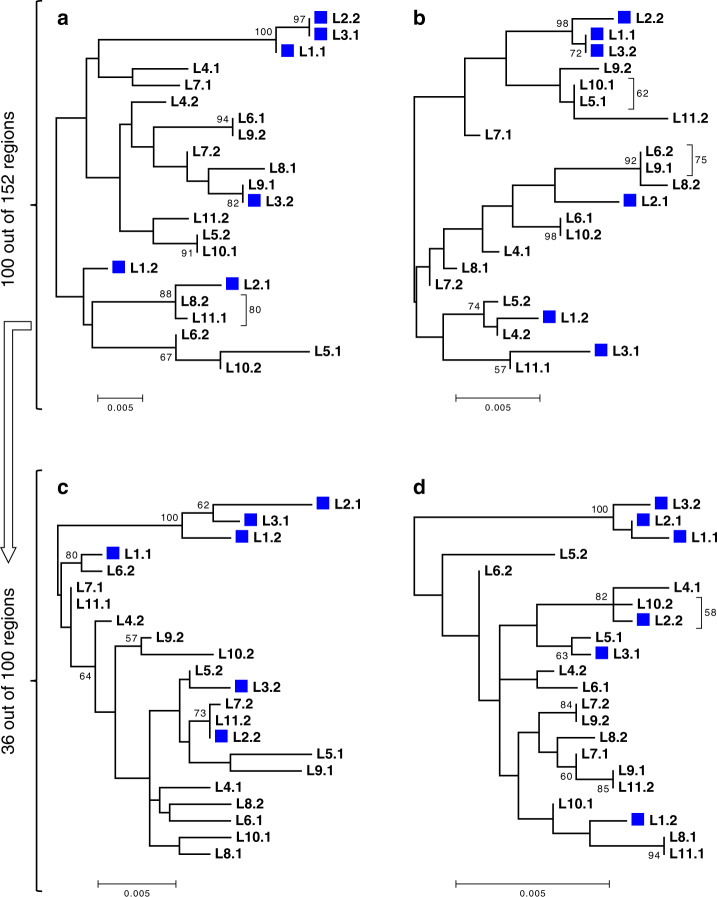
Phylogenetics of L1-L11 haplotypes hints at hybrid origin of the small cluster. Midpoint-rooted phylogenetic trees constructed for four segments simultaneously phased in all 11 *A. vaga* individuals (L1-L11) from both genetic clusters and suggestive of the hybrid origin of the individuals of the small cluster. Haplotypes from the individuals of the small cluster are represented by blue boxes. Overall, in 100 out of 152 tested segments, we detected a well-supported monophyletic group formed by three haplotypes, one haplotype from each of the individuals L1, L2, and L3 (as in **a**–**d**). Among those 100 segments, in 36 cases midpoint rooting separated a monophyletic clade comprising three haplotypes of L1, L2, and L3 from the rest of the tree (as in **c**, **d**). The displayed phylogenies were constructed for four phased segments located on different contigs in the L1 diploid assembly and spanning 1058 (**a**), 1452 (**b**), 1782 (**c**), and 1824 (**d**) base pairs. Trees were built using the maximum likelihood method in PhyML^74^ under the GTR + G model with 1000 bootstrap replicates. The bootstrap support values ≥50% are shown next to branches or next to brackets. Bootstrap support values are rounded to the nearest integer. Only those SNPs that satisfied all filtering criteria and were simultaneously phased in L1-L11 were used to reconstruct phylogenies (*n* = 46, 32, 43, and 30 non-singleton SNPs in **a**–**d**, respectively); the remaining sites were treated as monomorphic (see “Methods” section). Indices 1 and 2 designate the two haplotypes of a single individual. Source data are provided as a Source Data file.

To gain more insight into the relationships between haplotypes from the small and large clusters, we analyzed midpoint-rooted maximum likelihood phylogenies for all 152 segments phased in L1-L11 without pre-filtering segments for incongruent groupings of haplotypes. Inspection of the resulting phylogenies revealed that in the majority of these cases, L1, L2, and L3 were clustered by one of the two haplotypes but not by the other one. In total, in 100 out of the 152 segments, three haplotypes (one from each of the individuals L1-L3) formed a well-supported clade, while the other three haplotypes from L1-L3 were intermingled with haplotypes from L4-L11 (Fig. 7a–d). In 36 out of these 100 cases, midpoint rooting separated a tree into a monophyletic group containing 3 haplotypes from L1, L2, and L3 and the rest of haplotypes (Fig. 7c, d; see “Methods” section). There were no cases of a monophyletic group made up of all six haplotypes carried by L1, L2, and L3. Consistently, in 117 segments out of the 152, the unconstrained maximum likelihood tree was significantly different from the maximum likelihood tree in which all 6 haplotypes of the 3 individuals from the small cluster (L1-L3) were constrained to be monophyletic (Swofford–Olsen–Waddell–Hillis test^45,46^, *P* < 0.05 after the Bonferroni correction, see “Methods” section).

**Fig. 8:**
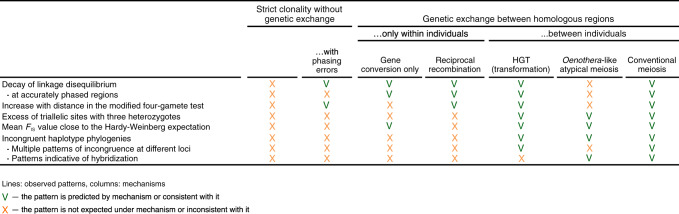
Patterns in *A. vaga* genetic variation data versus different mechanisms of genetic exchange. Note that although gene conversion and reciprocal mitotic recombination cannot increase the similarity between haplotypes harbored by different individuals and are not expected to create incongruent haplotype phylogenies where reciprocal closest counterparts of the two haplotypes of a single individual are found in two different individuals (as in Fig. 5; such cases are referred to as ‘incongruent haplotype phylogenies’ in this Figure), these processes might introduce ambiguous signals of haplotype groupings into phylogenies^4,15,18^.

These results would be difficult to explain under the HGT mechanism; however, they are consistent with conventional meiosis if we assume that the three individuals from the small cluster, L1-L3, are of hybrid origin, representing offspring from a cross of individuals from a population closely related to that of the large cluster and some other genetically distinct population (Fig. 8). This scenario also provides an explanation for the higher heterozygosity observed in the small cluster, as it reflects the presence of two relatively diverged haplotypes (Supplementary Fig. 9).

Interestingly, analysis of mitochondrial variation suggests that a single event of sexual reproduction involving individuals from different populations would not suffice to produce the genetic composition of individuals L1-L3 (Supplementary Figs. 1–3, 27–29, and Supplementary Notes 7, 8). While the mitochondrial haplotypes of L2 and L3 are very similar to those of individuals L4-L11, L1 carries a highly divergent mitochondrial haplotype, implying its distinct ancestry (Supplementary Figs. 1–3, 28 and Supplementary Note 7). This suggests repeated hybridization events, because the origin of the small cluster seems to require at least two reciprocal crosses between the population of the large cluster and another genetically distant population (Supplementary Note 7).

### Estimating hypothetical frequency of recombination

What incidence of meiosis or HGT would be required to alone explain the observed rate of LD decay? To address this question, we need to know *c*, the recombination rate per nucleotide per generation (Supplementary Note 10). *c* can be inferred from the ratio of the population-scaled recombination rate 4*N*_e_*c* to the population-scaled mutation rate 4*N*_e_*μ*, if *μ*, the mutation rate, is known. For individuals L4-L11, 4*N*_e_*c* turned out to be of the order of 10^−2^ (Supplementary Note 10). The level of genetic variation suggests that 4*N*_e_*μ* is also ~10^−2^ (Supplementary Note 10 and Supplementary Table 8). Thus, *c*∼*μ*. Unfortunately, there are no data on mutation rate in *A. vaga*. If it is within the range of 10^−9^–10^−8^, as it is the case for a variety of multicellular eukaryotes^23,47^, *c* is also ~10^−9^–10^−8^. To obtain such *c*, 1 meiosis must occur every ~10–100 generations, or, alternatively, 1 act of acquisition of a piece of DNA from another individual (transformation) must occur per genome every ~1–10 generations (Supplementary Note 11).

## Discussion

We analyzed whole genomes of 11 *A. vaga* individuals, and discovered that variation within the natural population of this bdelloid rotifer is thoroughly randomized. Within a locus, genotype proportions are overall close to Hardy–Weinberg expectations, and alleles at different loci that are distant enough from each other are in linkage equilibrium. This lack of statistical associations between alleles constitutes evidence^48^ for interindividual genetic exchanges and recombination in this species. In particular, the modified four-gamete test rules out gene conversion as the sole cause of LD decay, while analyses of triallelic sites and haplotype phylogenies suggest that genetic exchanges take place (Fig. 8).

However, interindividual genetic exchanges can occur due to two very different mechanisms. One is sexual reproduction *sensu stricto*, which involves mating between individuals and reciprocal meiotic recombination, and the other is HGT (perhaps as a result of transformation) followed by non-reciprocal recombination that incorporates the acquired DNA into the genome of the individual. Can we distinguish between these two options?

The strongest evidence for conventional sex is the existence of hybrids. If our interpretation is correct, and three individuals, L1-L3, that constitute the small cluster are, indeed, of hybrid origin, this suggests the existence of sexual reproduction (Fig. 8). Notably, incongruence between nuclear and mitochondrial data found for L1 and L2-L3 also argues for sexual reproduction. However, sex apparently must occur rather often, every ~10–100 generations, to alone produce the observed rate of LD decay—and it is difficult to imagine that hypothetical bdelloid males were overlooked by generations of zoologists if they are that common. HGT also must be rather common to alone lead to the observed LD decline, but, at least, there are no observational data that directly contradict this possibility. Of course, HGT alone cannot explain the existence of hybrids (Fig. 8).

One could argue that our estimate of the required prevalence of meiosis is inflated. There are three possible causes of this. First, if the true mutation rate in *A. vaga* is substantially below the assumed range of 10^−9^–10^−8^, the estimates of *c* and of meiosis frequency would be lower. However, in eukaryotes, reports of mutation rates below 10^−9^ are restricted to unicellular species^49^. Second, mitotic recombination and gene conversion can also contribute to LD decay (it is noteworthy that gene conversion was recently proposed to be an important determinant of LD patterns in cyclically parthenogenetic *Daphnia pulex*^50^). This could affect our calculations based on the assumption that the only source of LD decay is reciprocal meiotic recombination and consequently bias upwards estimates of the prevalence of meiosis. Finally, it is possible that both sex and HGT work together to randomize the population.

In summary, neither sexual reproduction nor HGT provides a simple explanation for our data, and we have to conclude that the mechanisms of interindividual genetic exchanges and recombination in the bdelloid rotifer *A. vaga* remain obscure. Still, the data on genetic variation strongly suggest regular occurrence of these processes in *A. vaga*.

## Methods

### Establishment of clonal cultures of *A. vaga*

We collected rotifers from clumps of moss which grew on trunks of aspen *Populus tremula* at 120–170 cm height. Clumps of moss were sampled in two geographically distant areas: the first near the Hydrobiological Station “Lake Glubokoe”, in Ruza district of the Moscow region, Russia (L1-L4, L6-L10), and the second in the vicinity of Shilovo village, Kostroma region, Russia (L5 and L11). Approximate sampling coordinates are provided in Supplementary Table 1. All sequenced individuals collected in the same area were sampled from different trees at least 20 m apart. Individual rotifers were isolated and identified to the species level based on morphological criteria^51^. Clonal cultures were established from individuals identified as *Adineta vaga*, which were rinsed in Milli-Q water and transferred to 96-well cell culture plates. To confirm that just a single individual was transferred to each well, plates were visually inspected daily for the next 3 days after inoculation. When a culture reached the size of ~30 individuals, it was transferred to a separate Petri dish containing Milli-Q water. Cultures were kept at 15–20 °C and fed *E. coli* (DH5α strain) grown overnight at 37 °C in LB medium. When the total number of rotifers in the dish reached ~1000, they were harvested for DNA extraction. In total, 11 cultures (lineages) of *A. vaga*, L1-L11, each originating from a separate tree, were established. The species identity of cultures L1-L11 was additionally confirmed by the number of pharyngeal teeth in the rake organ (four U-shaped hooks on each side of the mouth), a distinctive feature of *A. vaga*. We checked that the individuals L1-L11 cluster with reference *A. vaga* isolates using *COX1* marker phylogeny (Supplementary Note 1 and Supplementary Figs. 1–3).

### DNA extraction

Total DNA was isolated on Promega Wizard SV Genomic DNA Purification Kit columns (Promega, United States) according to the manufacturer’s protocol.

### Library preparation

DNA was fragmented using Covaris S2 sonicator. Libraries were prepared using TruSeq DNA sample preparation kit (Illumina) according to the protocol, with the following modification: for ligation, we used adapters from the TruSeq RNA sample preparation kit, as they have lower concentration and are thus more suitable for low-input samples.

### Genomic DNA sequencing

We sequenced the genomic DNA of 11 *A*. *vaga* lineages, L1-L11, to the coverage of ~40–100× (as determined relative to the L1 diploid assembly, see below) with Illumina paired-end libraries on the Illumina HiSeq platforms 2000 or 2500 using TruSeq SBS kits v.3 or v.4 respectively. The exact numbers of paired-end reads (2 × 98, 2 × 100, or 2 × 101 base pairs [bp]) generated for each lineage and the resulting coverage are presented in Supplementary Data 1.

In addition, we sequenced one of the lineages, L1, on the Illumina MiSeq platform to produce de novo genome assembly (see section “Reference genome assembly and filtration”). To minimize any bias in the way different samples were analyzed, the MiSeq reads obtained for L1 were used only for assembly of the *A. vaga* reference genome. Variant calls for the lineage L1 were produced in the same way as for the rest of the samples, using alignments of HiSeq reads.

### Removal of adapter sequences and quality trimming

We used Trimmomatic (V0.33)^52^ to remove adapter sequences and perform quality trimming of raw sequencing reads. The parameters for quality trimming were set to “LEADING:20 TRAILING:20 SLIDINGWINDOW:5:20”. Reads shorter than 50 bp were discarded. Numbers of reads left after adapter and quality filtering for each individual are given in Supplementary Data 1. We performed quality control checks on the reads with FastQC (v0.11.3, https://www.bioinformatics.babraham.ac.uk/projects/fastqc/).

### Reference genome assembly and filtration

The first genome of a bdelloid rotifer, *A. vaga*, was published in 2013^11^. However, we found that the genomes of individuals L1-L11 exhibit substantial nucleotide divergence from the published *A. vaga* genome, which prevented us from using the published genome as a reference: the average identity of BLAST hits for HiSeq reads from L1-L11 to the published assembly was only ~87–88% (Supplementary Table 2). This finding is in line with previous reports showing that morphological species of bdelloid rotifers frequently comprise complexes of divergent cryptic species^53^.

To obtain a genome assembly which could be used as a reference, we additionally sequenced one of our lineages, L1, on the MiSeq platform, which made it possible to generate a de novo genome assembly of reasonable quality (Supplementary Table 3). For this purpose, we generated a separate Illumina library for L1 and sequenced it on the MiSeq system with Miseq reagent kits v.2 or v.3. Three sequencing runs were performed, yielding the totals of 10,172,970 (2 × 301 bp), 15,397,651 (2 × 251 bp) and 20,061,190 (2 × 261 bp) reads. Trimming of low-quality bases and adapter removal was performed using Trimmomatic (V0.33)^52^ with the parameters for quality trimming set to “LEADING:20 TRAILING:20 SLIDINGWINDOW:5:20”. Reads shorter than 50 bp were discarded. The total of 34,403,183 reads left after these steps were used to produce a de novo genome assembly.

Assembly of the L1 genome was carried out with SPAdes (version 3.6.0)^54^ based on the MiSeq reads. SPAdes was run in diploid mode (--diploid option) without preliminary read error correction (--only-assembler option). K-mer sizes were set to: -k 21,33,55,77,99,127. The initial assembly had an N50 of 18 kilobases (kb) and contained 233.8 megabases (Mb) of sequence in 51,852 contigs ≥500 bp in length (Supplementary Table 3). The obtained contigs displayed a high level of concordance with PacBio reads independently obtained for the L1 lineage (Supplementary Methods).

A bimodal distribution of the GC-content in the contigs suggested presence of bacterial contamination. However, contaminant-derived contigs were easily distinguished from the target *A. vaga* contigs by a significantly lower coverage and higher GC-content (Supplementary Fig. 4). Taxonomic classification of the initial contigs was performed with the Blobology pipeline^55^ (revision bc2300c, https://github.com/blaxterlab/blobology) based on their BLAST^56^ hits to nt database with *E*-value cut-off set to 1 × 10^−5^. Taxon-annotated GC-coverage plots^55^ were created with the R script (makeblobplot.R) provided as a part of the same pipeline. Coverage of contigs was estimated from HiSeq reads obtained from sequencing of the initial Illumina library constructed for the lineage L1 (not included in the assembly).

The resulting taxon-annotated GC-coverage plots were used to partition the contigs into sets of target and contaminant contigs (Supplementary Fig. 4). To filter out contaminant sequences, we first removed from the assembly all contigs with GC-content ≥0.5 or coverage with HiSeq reads ≤3. To ensure that the final assembly comprised only contigs truly originating from *A. vaga*, we performed a BLAST^56^ search with the pre-filtered set of contigs against the published *A. vaga* genome^11^. BLAST searches were performed with blastn from BLAST+ (version 2.2.31) with the following parameters: -evalue 1e-10 -outfmt “6 qseqid sseqid pident length mismatch gapopen qstart qend sstart send evalue bitscore qlen slen” -task dc-megablast.

Only contigs with at least one dc-megablast hit to the published assembly with *E*-value ≤1 × 10^−100^ and a minimum alignment length of 500 bp were retained. The resulting filtered assembly had an N50 of 22 kb, with 19,068 contigs ≥500 bp in length covering 197 Mb, comparable with 218 Mb reported for the published *A. vaga* assembly^11^. Summary assembly statistics for the initial and filtered sets of contigs are shown in Supplementary Table 3. The filtered assembly was used as a reference in the subsequent analyses. The assembly statistics were generated with QUAST (v5.0.0)^57^. Results of BUSCO analysis are presented and discussed in Supplementary Methods and Supplementary Figs. 6, 7.

### Construction of non-redundant haploid sub-assembly

Due to high heterozygosity, the two haplotypes of the *A. vaga* genome assemble into separate contigs at the majority of loci^11^. Still, in a substantial portion of the genome, the two haplotypes collapse into a single contig, leading to a mosaic organization of the assembly with alternating ploidy levels. To reduce redundancy of the assembly and to ensure that only truly diploid loci are analyzed, we obtained a reduced haploid representation of the *A. vaga* genome^58^.

Briefly, we searched for the pairs of reciprocally highly similar genomic segments within the L1 assembly, discarding genomic regions without haplotypic counterparts and non-reciprocal best matches. From each pair of reciprocal best matches we retained only a single segment. This was achieved by using blastn from BLAST+ (version 2.2.31), blastz^59^ and single_cov2 commands from the all_bz^60^ program (v.15). See Supplementary Methods for details. The resulting haploid sub-assembly spanned 76,098,573 bp in haploid segments (haploid contigs) ≥500 bp in length (Supplementary Table 3), suggesting that ~77% of the original assembly corresponds to loci represented in the assembly by two haplotypes.

### Annotation of protein-coding genes

We predicted protein-coding genes in the filtered diploid assembly of the *A. vaga* L1 genome using AUGUSTUS (v.2.7)^61^ and GeneMark.ES Suite (version 4.32)^62^. Intron and transcribed region hints for AUGUSTUS were prepared with STAR aligner (v. 2.4.2a)^63^. For this purpose, RNA-seq reads generated for the first published *A. vaga* genome^11^ (available at http://www.genoscope.cns.fr/adineta/data/Avaga_rnaseq_sort.bam) were mapped on the L1 diploid assembly with strict mapping parameters. The list of putative splice junctions (a total of 119,058 suggested intron boundaries) was obtained taking into account only uniquely mapped reads (16% of the available RNA-seq reads, 21 million reads). The initial set of predictions comprised 78,303 gene models originating from 75,877 loci. After a quality check a total of 61,531 gene models remained^58^. The details on gene prediction procedure are provided in Supplementary Methods. Difference in the number of genes predicted for the *A. vaga* L1 genome in this study and that reported for the first published *A. vaga* genome is also discussed in Supplementary Methods. The filtered gene models predicted in the diploid assembly were transferred to the coordinate system of the haploid sub-assembly, only those gene models that were fully contained within the haploid contigs were retained (*n* = 23,802).

### Identification of allelic regions and allelic genes

To obtain a subset of the *A. vaga* genome with high-confidence ploidy, we identified genomic regions that could be assigned into pairs of highly similar segments with conserved gene order. We initially searched for collinear groups of genes within the assembled *A. vaga* reference genome using MCScanX^28^ (available at http://chibba.pgml.uga.edu/mcscan2/MCScanX.zip; accessed August 28, 2017) with an *E*-value cut-off of ≤1 × 10^−5^. MCScanX was run on the results of all-versus-all blastp search of the proteins predicted in the L1 diploid assembly. BLAST results were restricted to hits with *E*-value ≤1 × 10^−10^ with the maximum number of target sequences to output per query sequence set to 5.

To focus on the genomic regions for which ploidy could be inferred with high certainty, we extracted the subset of ‘allelic regions’ defined as collinear blocks with a high degree of collinearity (fraction of collinear genes in a block ≥0.7) and low synonymous divergence (average Ks ≤ 0.2). Coordinates of allelic regions were mapped into the coordinate system of the haploid sub-assembly, and only those regions that were fully contained within boundaries of the haploid contigs were retained. In addition, we delineated the subset of ‘allelic genes’ composed of collinear gene pairs embedded in allelic regions. Detailed descriptions of the procedure and of the obtained subsets are available in Supplementary Methods.

In contrast with the first published genome of *A. vaga* where multiple instances of collinear regions residing on the same contig and organized as palindromes were detected, we identified only a single palindrome. Although our relatively low N50 value does not allow a detailed analysis, this finding is in line with the findings of Nowell et al. who have not detected palindromes in the genome assembly of another *Adineta* species, *A. ricciae*^13^.

### Mapping of Illumina reads

Trimmed Illumina HiSeq reads for each sequenced individual were aligned to the original (diploid) filtered L1 assembly and to the haploid sub-assembly with Bowtie 2 (version 2.3.2)^64^ with parameters “--no-mixed --no-discordant” and the maximum insert size of 800 bp. Alignments of reads to the original assembly were used to filter out ambiguously mapped reads. Variant calling was performed from end-to-end alignments of filtered reads uniquely mapped to the haploid sub-assembly. Only properly paired reads with a high quality of mapping (MAPQ ≥ 20) were used for analyses. See Supplementary Methods for details on filtering.

### Variant calling and filtering

Variant calls were generated using the SAMtools^65^ mpileup utility (v.1.4.1) with the parameters “-aa -u -t DP,AD,ADF,ADR” followed by the command “bcftools call” with the “-m” option. The obtained raw genotype calls were subjected to stringent filtering as follows. For the main analyses, we excluded sites (1) with SNPs within 10 bp of an indel, (2) with missing genotypes or QUAL value <50, (3) located on contigs from the haploid sub-assembly shorter than 1000 bp, (4) within repetitive regions, (5) with low coverage (DP < 10 in any of the samples), (6) with an extremely high depth of coverage, or (7) within the windows that were outliers for SNP density. Filtering was carried out using combinations of BCFtools (v.1.4.1, https://samtools.github.io/bcftools/), VCFtools (v. 0.1.15)^66^, bedtools (v2.26.0)^67^, and SnpSift (v.4.3 s)^68^ utilities. Details on filtering criteria and SNP datasets used for different analyses are provided in Supplementary Methods. The total numbers of sites included in the main SNP datasets (SNP datasets I and II) are listed in Supplementary Table 5.

To assess the reliability of the resulting SNP calls^69^, we compared SNPs identified with SAMtools to SNPs called for the individuals L1-L11 with GATK (version 4.1.2.0)^70^. In the stringent SNP dataset I (*n* = 2,282,099) generated with SAMtools, the average proportion of SNPs identically called with GATK for a particular individual is ~95% (for details, see Supplementary Methods and Supplementary Table 6).

Pairwise genotypic distances between individuals were computed using the compute_genotypic_distances.pl script (https://github.com/vakh57/bdelloid_scripts).

### MDS analysis

Multidimensional scaling analysis of identity-by-state pairwise distances between the sequenced *A. vaga* individuals was performed with PLINK (v1.90b5.4)^71^ based on a thinned subset of SNPs (*n* = 66,483) from the stringent SNP dataset I with minor allele count ≥2. See Supplementary Methods for details.

### Computational phasing of genotypes

Local haplotypes were assembled for each individual L1-L11 separately, using HapCUT2 (revision bd1a739, https://github.com/vibansal/HapCUT2)^21^ with the “--error_analysis_mode 1” option to compute switch error scores. For LD-related analyses, we obtained two sets of aggressively filtered phased haplotype blocks: phased dataset 1 which was used as the main dataset, and the auxiliary phased dataset 2 subjected to even more stringent filtering. Briefly, from both datasets, we discarded phased blocks covered by reads supporting more than two different ‘haplotypes’ for a pair of SNP sites in a single individual. The logic behind this filtering step is that each individual can carry no more than two different haplotypes for a pair of SNP sites. Those pairs of sites with support for more than two ‘haplotypes’ in the aligned reads from a single individual are likely to stem from PCR template switches^29^ or from paralogous alignments and other artifacts and to be associated with phasing errors. Phased blocks left after this step were included in phased dataset 1. Statistics on the sizes of the resulting blocks and on the numbers of SNPs spanned by them are provided in Supplementary Tables 9 and 10. Phased dataset 2 was filtered further on the basis of presence in a block of SNPs with switch or mismatch quality values <100. All blocks comprising more than one such SNP were discarded from phased dataset 2. Blocks harboring one such SNP were split at the corresponding site, and the chunks of the original block resulting from the split were analyzed separately. Filtering of phased blocks was carried out using the get_conflicting_variants_indices.pl and filter_hapcut2_haplotype_blocks.pl scripts (https://github.com/vakh57/bdelloid_scripts).

For each individual, HapCUT2 assigns to haplotypes only those SNPs at which this individual is heterozygous. We complemented the phased haplotype blocks with the data on homozygous SNPs embedded within the phased blocks. For this purpose, we searched for homozygous SNPs flanked by heterozygous SNPs belonging to the same phased block, and assigned the homozygous SNP to both haplotypes of this block. Finally, we identified genomic segments encompassing groups of SNPs where genotypes for all individuals L4-L11, or for all individuals L1-L11, are simultaneously phased. For LD-related analyses, groups of SNPs representing different phased genomic segments were processed separately. See Supplementary Methods for details on filtering and processing of phased haplotype data. The analyses performed to assess the quality of phasing are described in Supplementary Note 2.

### Analysis of linkage disequilibrium (LD)

To analyze patterns of LD in *A. vaga* from phased SNP data, we calculated *r*^2^ values individually for each phased segment using VCFtools (version 0.1.15)^66^. If not stated otherwise, the reported results are based on the analysis of SNPs from the phased dataset 1. For this analysis, we additionally excluded all sites which were likely to be falsely called as homozygous in some individuals. For this purpose, for each individual, we looked for sites which were called as homozygous but were nevertheless represented in the aligned reads from this individual by two nucleotides, each supported by at least two reads. Such sites were excluded from analysis in all individuals. The reported results are for variants with a minor allele count of at least 4 among individuals L4-L11 (Fig. 2a) or L1-L11 (Supplementary Fig. 13c). The results obtained for the more severely filtered phased dataset 2 are shown in Supplementary Fig. 13b. The decay of LD with physical distance was fitted using second-degree LOESS regression with the smoothing parameter set to 0.4 as implemented in the *geom_smooth* function from the ggplot2 package (version 3.2.1) in R. LD decay among L4-L11 based on the phased dataset 1 (the same data as in Fig. 2a) was also fitted by first degree and second degree LOESS with the smoothing parameter selected according to the bias-corrected Akaike information criterion (the *loess.as* function from the fANCOVA R package version 0.5–1; Supplementary Fig. 11). To determine the baseline *r*^2^ values, we computed *r*^2^ for SNPs residing on different contigs.

See Supplementary Methods for details and for description of the approaches used to assess the LD decay from the unphased genotype data. Detailed explanation of the modified four-gamete test is provided in Supplementary Note 3.

### Inferring signatures of recombination

We tested for recombination within 434 phased genomic segments of the *A. vaga* genome harboring at least 15 non-singleton SNPs simultaneously phased in all individuals L4-L11. These segments were distributed between 352 contigs belonging to the L1 diploid assembly and spanned a total of 364,361 base pairs. For each such segment, we reconstructed sequences of the two haplotypes in each individual based on the corresponding sequence from the haploid sub-assembly and the set of phased SNPs using BCFtools (v.1.4.1). As previously, sites that were likely to be falsely called as homozygous in some individuals were not considered. Only those SNPs that satisfied all filtering criteria and were simultaneously phased in L4-L11 were used to reconstruct haplotypes; the remaining sites were treated as monomorphic. For each such segment, we assessed whether the decay of *r*^2^ is significantly correlated with physical distance^25^ and performed the sum of distances^26^ and pairwise homoplasy index^27^ (PHI) tests. PHI tests were performed using PhiPack (available at http://www.maths.otago.ac.nz/~dbryant/software/PhiPack.tar.gz; accessed July 1, 2018) and the other two types of tests using LDhat (version 2.2)^72^. Details are given in Supplementary Methods.

### Testing for Hardy–Weinberg equilibrium

*F*_IS_ values were computed for biallelic SNPs (belonging to the stringent SNP dataset II, see Supplementary Methods) common among the individuals of the large cluster, L4-L11. To define common SNPs, we used a minor allele count cut-off of 4. For each locus, we computed *F*_IS_ as $1 - \frac{{H_{\mathrm{o}}}}{{H_{\mathrm{e}}}}$, where *H*_o_ and *H*_e_ stand for the observed and expected numbers of heterozygous genotypes, respectively. The values expected under Hardy-Weinberg equilibrium were obtained with the populations program from the Stacks pipeline (version 2.4)^73^. Fractional expected genotype counts were rounded to the nearest integer number using the compute_Fis.sh script (https://github.com/vakh57/bdelloid_scripts). We performed this analysis for whole-genome variants, as well as for subsets of the variants residing within the regions of the *A. vaga* genome with high-confidence ploidy (allelic regions and allelic genes). The overall numbers of analyzed SNPs and summary statistics on *F*_IS_ for each dataset are provided in Supplementary Table 11.

### Simulating different rates of clonal reproduction

To explicitly assess the values of *F*_IS_ expected under different rates of clonal reproduction with the number of sampled individuals equal to that of the analyzed individuals (*n* = 8), we ran forward population genetics simulations in SLiM (version 3.2)^41^.

Prior to running simulations, we obtained estimates of the population-scaled mutation rate 4*N*_e_*μ* for the sequenced individuals using the maximum likelihood approach implemented in the program mlRho (version 2.9)^31^ (Supplementary Note 10). Estimates of 4*N*_e_*μ* for the individuals belonging to the large cluster (L4-L11) were on the order of 10^−2^ (ranging from 0.0072 to 0.0094, Supplementary Table 8). Simulation parameters were chosen accordingly, so that 4*N*_e_*μ* in simulations was close to that estimated from the data: *N*_e_ = 2500 individuals, mutation rate *μ* = 10^−6^ per base pair per generation. For all simulations, we used a chromosome length of 10^6^ base pairs. All simulations involving any sexual reproduction had recombination rate of 10^−6^ per base pair per sexual generation. The recombination rate in the strictly clonal case is 0. We assumed the rate of neutral mutations to the rate of deleterious mutations to be 1:0.4. Deleterious mutations were drawn from the gamma distribution (mean selection coefficient of −0.01, shape parameter 0.1), and were assumed to be additive. We simulated cloning rates ranging from 0 (strictly sexual reproduction) to 1 (strictly clonal reproduction), running 100 replicates of each cloning rate. All simulations were run for 200,000 generations, at this point *F*_IS_ was measured for subsamples of the resulting SNPs.

To allow direct comparison of simulations with the data in terms of the sample size and allele frequencies, we employed the following procedure. For each replicate of each simulation, we randomly chose 8 individuals (matching the number of the analyzed individuals L4-L11), retained only those biallelic SNPs that had a minor allele count of at least 4 in the resulting dataset and then randomly sampled 200 SNPs. This yielded a total of 100 sets of SNPs per a simulation. For each resulting set of SNPs, we calculated the mean *F*_IS_. We analogously computed the mean *F*_IS_ for 100 random sets of SNPs (*n* = 200) biallelic among L4-L11 (minor allele count ≥ 4). *F*_IS_ observed in L4-L11 was found to be significantly higher than that expected under strict clonality (*P* < 0.01) and cloning rate = 0.999 (*P* = 0.03). *P* values for those simulated cloning rates lower than 0.999 (cloning rates 0, 0.5, 0.7, 0.9, 0.95, 0.99) were non-significant (all *P* values > 0.05). For each cloning rate, one-sided *P* value was computed as the fraction of 100 random sets of SNPs with mean *F*_IS_ equal or higher than the minimum mean *F*_IS_ (−0.0917) across 100 random sets of SNPs drawn from L4-L11. No correction for multiple comparisons was applied.

### Identifying incongruent groupings of haplotypes

We conducted detection of incongruent groupings of haplotypes among three sets of phased segments. Sets A and B (see below) include segments phased in all individuals of the large cluster (L4-L11), while set C includes segments phased in individuals from both the small and the large cluster (L1-L11).

First, to detect incongruent groupings of haplotypes which may suggest genetic exchanges among L4-L11, we started from the same set of 434 phased genomic segments harboring at least 15 non-singleton SNPs simultaneously phased in L4-L11 which was used to perform tests for recombination. As previously, sites that were likely to be falsely called as homozygous in some individuals were not considered.

To ensure that the analysis of incongruence is not affected by the presence of repetitive regions or close paralogs which could be potentially mistaken for haplotypes (alleles), we further filtered the set of the phased segments based on the BLAST search against the L1 diploid assembly. For each haplotype (*n* = 16) of each considered segment (*n* = 434), we tabulated the number of unique hits in the L1 diploid assembly (only a single high-scoring alignment per the same L1 contig was considered for each query, BLAST option “-max_hsps 1”). The median number of hits per haplotype was equal to 4; usually there were 2 highly similar hits (≥90% nucleotide identity) corresponding to two L1 haplotypes present in the assembly and 2 hits with lower identity (~65–85%) likely representing paralogs (Supplementary Fig. 30). However, haplotypes from some segments had hits to a substantially larger number of contigs. In most such cases there were two highly similar full-length hits (‘haplotypes’) and a number of short low-identity hits covering a small portion of a segment. To ensure that genomic segments containing sequences with a large number of diverged copies in the *A. vaga* genome do not introduce a spurious signal of incongruence, we excluded a segment if any among its haplotypes had hits to more than 10 contigs in the diploid assembly regardless of the hit identity. In the remaining set, an allele had an average of 2.07 high-identity (≥90%) hits and an average of 3.02 low-identity (<90%) hits. We further removed segments with alleles harboring more than 2 high-identity hits. After these steps, out of the original 434 phased segments, we were left with 303 segments spanning a total of 243,455 base pairs and distributed among 263 contigs of the L1 diploid assembly. These 303 segments were used to look for incongruent groupings of haplotypes (set A). The analogous procedure was applied to the sets B and C (see below).

For each of the two haplotypes within each individual L4-L11, we computed the nucleotide distance (proportion of nucleotide differences) to the other haplotype within the same individual and to each haplotype in all other individuals. For each haplotype, we then identified the closest haplotypic counterpart in other individuals. To test the robustness of this matching, we compared the number of nucleotide differences between the haplotype and its closest (N1) and second closest (N2) counterpart. The haplotype was defined as having an unambiguous closest counterpart if the difference between N2 and N1 was 3 SNPs or more (N2–N1 ≥ 3).

To analyze the patterns of haplotype clustering among individuals, we selected, for each individual, those phased segments where the closest counterpart was unambiguously identified for both its haplotypes, H1 and H2. Such cases can be subdivided according to whether the closest counterparts of the two haplotypes (H1´ and H2´) were found in the same individual or in two different individuals. To exclude from consideration groupings of haplotypes that could be caused by gene conversion, we further required the distances separating pairs of identified haplotypic counterparts from different individuals (H1-H1´ and H2-H2´) to be shorter than the distances separating the two haplotypes within the same individual (Supplementary Discussion). Finally, we retained only reciprocal best matches. That is, we required that H1 and H2 were also identified as the closest counterparts of the haplotypes H1´ and H2´, respectively. In total, we were able to identify the reciprocal closest counterparts for both haplotypes of at least one individual for 90 out of the 303 analyzed phased genomic segments. This procedure was carried out using the find_haplotypic.counterparts.pl script (https://github.com/vakh57/bdelloid_scripts).

Among these 90 segments, only in 12 segments we found a pair of individuals such that their haplotypes represented reciprocal closest counterparts (congruent grouping). By contrast, in 79 segments, we found at least one individual such that its two haplotypes had reciprocal closest counterparts in two different individuals (incongruent grouping; for one segment, both a congruent and an incongruent grouping were observed for different individuals).

To check whether the identified congruent and incongruent groupings were reliable, for each of the 90 segments, we built an unrooted phylogenetic tree using the maximum likelihood method in PhyML (version 3.1)^74^ under the GTR + G model (four substitution rate categories, the gamma shape parameter estimated from the data) with 1000 bootstrap replicates. Trees were rooted at the midpoint using the ETE 3 toolkit^75^. Bootstrap support values for haplotype groupings were extracted with the aid of the print_nodes_2leaves.py script (https://github.com/vakh57/bdelloid_scripts). In total, in 10 of the 12 segments with congruent grouping, this grouping received strong (≥70%) bootstrap support (Supplementary Discussion). In 52 of the 79 segments with incongruent grouping(s), at least one of the incongruent groupings received strong bootstrap support.

The resulting numbers of segments with well-supported incongruent groupings of haplotypes for each individual are shown in Table 1. Per individual numbers of segments with congruent groupings of the two haplotypes, as well as patterns of haplotype groupings found in such segments, are provided in Supplementary Data 5. We also computed, for each pair of individuals, the overall number of cases where there existed a third individual harboring one haplotype clustered with a haplotype of the first individual from the pair and the other haplotype clustered with a haplotype of the second individual (Supplementary Table 19). Select phylogenetic trees were visualized in MEGA7 (version 7.0.26)^76^, the root was placed at the midpoint.

Second, to check whether the signal of incongruence is driven by inaccuracies in SNP calling including those potentially introduced by index hopping and heterozygote undercalling, we repeated the analysis for L4-L11 applying additional filters to SNPs and using haplotypes reconstructed using only those SNPs that passed these extra filters. Namely, for this analysis, we excluded sites (1) called as homozygous in any individual L4-L11 but represented in the aligned reads from this individual by more than one nucleotide (even if a second nucleotide was supported only by a single read), (2) sites called as heterozygous in any individual but with one of the two alleles supported by less than 30% of the aligned reads, (3) sites with alleles supported only by forward or reverse reads in any of the individuals. Additional SNP filtering led to a drop in the number of available phased segments from 303 to 190 (set B). The numbers of phased segments displaying incongruent groupings of the two haplotypes detected for each individual L4-L11 among these 190 segments are given in Supplementary Table 20. In total, out of the 190 segments from the set B, 40 exhibited incongruent groupings of the two haplotypes at least in one individual, with 25 segments showing strong bootstrap support (≥70%) for such groupings.

Third, to see whether genetic exchange in *A. vaga* can potentially involve individuals from different clusters, we looked for cases of incongruent haplotype groupings among all individuals L1-L11. In this case our inference is based on 152 segments harboring at least 15 non-singleton SNPs simultaneously phased in all individuals L1-L11 (set C). These 152 segments were distributed between 138 contigs belonging to the L1 diploid assembly and spanned a total of 114,592 base pairs. Phased segments included in the set C are based on SNPs passing the same additional stringent criteria aimed to reduce the potential influence of SNP calling inaccuracies which were applied to the set B (see above). The numbers of phased segments from the set C displaying incongruent groupings of the two haplotypes with strong bootstrap support for each individual L1-L11 are given in Supplementary Table 21.

### Local examples of recombination

To identify local examples of recombination events, we narrowed the dataset to four individuals (L6-L9), only requiring that a segment was simultaneously phased in them. To minimize the probability of phasing errors, we based this analysis on the stringently filtered phased dataset 2 (see Supplementary Note 2). We focused on relatively long segments (>2000 bp), each carrying at least 15 non-singleton SNPs simultaneously phased in L6-L9 (*n* = 49). Out of these segments, 38 tested positive for recombination according to the PHI test^27^ (at *P* < 0.05 after the Bonferroni correction). We then analyzed these segments with RDP4 (v.4.97)^77^. Three of the identified segments, positioned at three distinct contigs of the L1 diploid assembly (coordinates are given in Supplementary Data 6), are shown in Fig. 6. For these three segments, we visually checked alignments of reads in the corresponding regions of the genome. Among recombination detection methods implemented in RDP4, we used the following six: RDP^78^, GENECONV^79^, Chimaera^80^, MaxChi^81^, BootScan^82^, and SiScan^83^. We employed RDP4 with the default settings except that: (1) sequences were treated as linear, (2) we used window size of 10 variable sites for RDP and of 20 variable sites for Chimaera and MaxChi, (3) we used window size of 100 and step size of 10 nucleotides for BootScan and SiScan. We considered only those recombination events that were identified at least by three of the six methods with a *P* value cut-off <0.05 (with the default RDP4 Bonferroni correction on). Putative recombination events that according to RDP4 could be due to factors other than recombination were not used. For all shown recombination events, identification of the recombinant sequence and of minor/major parental sequences was ambiguous. Supplementary Data 6 contains the list of all recombination events detected for the three shown segments (including those events predicted with too few methods and not depicted in Fig. 6) along with the associated *P* values. All three segments overlapped with genes with unknown function predicted in the L1 diploid assembly (Supplementary Data 6).

### Phylogenetic analysis of haplotypes in L1-L11

We additionally analyzed maximum likelihood phylogenies for all 152 segments from the set C (see above) without pre-filtering segments for incongruent groupings of haplotypes. For each segment from the set C, we obtained an unrooted phylogenetic tree based on reconstructed sequences of both haplotypes for each individual L1-L11. As previously, trees were built using the maximum likelihood method in PhyML^74^ under the GTR + G model (four substitution rate categories, the gamma shape parameter estimated from the data) with 1000 bootstrap replicates. Trees were rooted at the midpoint.

We searched for monophyletic groups with strong bootstrap support (≥70%) comprising exclusively haplotypes from L1, L2, and L3 using the ETE 3 toolkit^75^ to parse the resulting midpoint-rooted phylogenetic trees. Then we tabulated the number of haplotypes forming each such group. In total, out of 152 analyzed segments (from 138 contigs of the L1 diploid assembly), 119 had a monophyletic group formed by three haplotypes, one haplotype from each of the individuals L1, L2, and L3, with 100 segments (from 93 contigs) exhibiting strong bootstrap support for such a group. Out of these 100 segments with a well-supported monophyletic group of 3 haplotypes, in 6 cases such a group was contained within a poorly supported monophyletic group of 4 haplotypes from L1, L2, and L3. In total, we detected only 8 segments for which there existed a monophyletic group comprising 4 out of 6 haplotypes of L1, L2, and L3, however, in no cases was such a grouping well supported. We did not detect a single segment with monophyletic grouping of 5 or 6 haplotypes from L1, L2, and L3. We also did not detect a single case supporting simultaneous clustering of L1, L2, and L3 based on both haplotypes; in other words, if there existed a monophyletic clade with three haplotypes from L1, L2, and L3, the other 3 haplotypes from L1-L3 never made up a monophyletic group.

Next, we searched for cases where a root (placed at the midpoint) would separate a monophyletic clade comprising three haplotypes of L1, L2, and L3 from the rest of the tree. In total, such pattern was detected for 36 out of 152 segments. These 36 segments belonged to 35 contigs of the L1 diploid assembly.

### Swofford–Olsen–Waddell–Hillis tests

To further check whether the presence of two distinct groups among the haplotypes of individuals L1-L3 is statistically supported, for each of the 152 segments, we performed the Swofford–Olsen–Waddell–Hillis (SOWH)^45^ test by comparing the unconstrained maximum likelihood tree to the maximum likelihood tree in which all 6 haplotypes from L1-L3 were constrained to be monophyletic. SOWH tests were carried out using SOWHAT^46^ program (revision 907c289, https://github.com/josephryan/sowhat) and one-sided *P* values for each segment were computed based on 10,000 simulated bootstrap replicates. Out of 152 segments, in 148 the unconstrained maximum likelihood tree was significantly different from the tree in which all 6 haplotypes of individuals L1-L3 were constrained to be monophyletic at the significance level of 0.05. After applying the Bonferroni correction, difference between constrained and unconstrained trees remained significant for 117 segments.

### Statistical analysis

Confidence intervals (CIs) demonstrated in Fig. 3c were determined by bootstrapping SNP pairs in each distance bin 1000 times and computing 95% bootstrap percentile CI for the fraction of recombinant SNP pairs with functions *boot* and *boot.ci* from the boot package (version 1.3.24) in R (version 3.6.3). Significance of the difference between proportions of recombinant SNP pairs for different distance bins was assessed by 10,000 times permuting SNP pairs between the two compared bins. Two-sided *P* value was computed for each pair of compared bins and adjusted with the Bonferroni correction.

To assess the significance of the difference in the mean distances between SNPs in non-recombinant and recombinant pairs, we used the permutation test performed by randomly relabeling SNP pairs as non-recombinant or recombinant (this procedure was repeated 10,000 times). The two-sided *P* value was computed as the fraction of permutations with the absolute difference between the mean distances separating SNPs from the two groups at least as extreme as in the data.

To test for recombination in individual phased genomic segments, for each segment we computed correlation of *r*^2^ with distance^25^ and sum of distances between pairs of sites carrying all four haplotypes^26^ in the actual and permuted data as implemented in LDhat^72^. One-sided *P* values for each segment were obtained from 10,000 permutations and adjusted for multiple comparisons using the Bonferroni correction. Significance of the PHI statistic^27^ for each segment was assessed under the assumption of a normal distribution of the PHI statistic; the obtained one-sided *P* values were corrected by applying the Bonferroni correction.

Significance of the difference between the observed and expected proportions of triallelic sites harboring all three heterozygous genotypes was assessed using one-sample *Z*-test for proportions (function *prop.test* from the stats R package [version 3.6.3] employed without continuity correction, two-sided test). 95% CIs around the regression lines in Supplementary Fig. 19a, b were obtained using the *geom_smooth* function from the ggplot2 package (version 3.2.1) in R.

### Reporting summary

Further information on research design is available in the Nature Research Reporting Summary linked to this article.

## Supplementary information

Supplementary Information

Peer Review

Reporting summary

Description of Additional Supplementary Files

Supplementary Data 1

Supplementary Data 2

Supplementary Data 3

Supplementary Data 4

Supplementary Data 5

Supplementary Data 6

Supplementary Data 7

Supplementary Data 8

Supplementary Data 9

## Data Availability

The Illumina sequencing data have been deposited at NCBI under BioProject ID PRJNA498886. Individual SRA accession numbers for deposited HiSeq reads are provided in Supplementary Table 22. MiSeq reads included in the obtained assembly of the *A. vaga* genome (L1) have been deposited with SRA accession numbers SRR8133179, SRR8133180, and SRR8133181. PacBio reads used to assess accuracy of phasing for L1 have been deposited at NCBI under BioProject ID PRJNA558051. The assembled (diploid) contigs for *A. vaga* (L1) are available at NCBI: the Whole Genome Shotgun project has been deposited at DDBJ/ENA/GenBank under the accession WJQV00000000. The version described in this paper is version WJQV01000000. The L1 diploid contigs are also available at 10.6084/m9.figshare.11620518.v2. The dataset accessible through this link also includes files containing haploid sub-assembly of the L1 genome, annotation of protein-coding genes in the GTF format produced for the L1 diploid contigs, and coordinates of gene models transferred to the haploid sub-assembly. Raw and filtered SNPs identified in L1-L11 (SNP dataset I) are available at 10.6084/m9.figshare.11625780.v2. The data used in the analysis of mitochondrial variation are available at 10.6084/m9.figshare.12008790.v2 and 10.6084/m9.figshare.11396955.v2. This analysis also involved publicly available sequences of *Philodina citrina* and *Rotaria rotatoria* mitochondrial genomes (the respective GenBank accession numbers: FR856884.1 and GQ304898.1). For annotation, we used a publicly available RNA-seq dataset (generated for the *A. vaga* genome^11^ published in 2013) which can be downloaded at http://www.genoscope.cns.fr/adineta/data/Avaga_rnaseq_sort.bam. BUSCO analysis involved publicly available assemblies of *A. vaga* genome^11^ downloaded from http://www.genoscope.cns.fr/adineta/data/Adineta_vaga_v2.0.scaffolds.fa.gz and of *A. ricciae* genome^13^ available at GenBank under the accession GCA_900240375.1. GenBank accession numbers for reference *COX1* sequences used in Supplementary Figs. 1–3 are given in Supplementary Data 9. Haplotype sequences reconstructed for L6-L9 in the three segments used to produce Fig. 6 are provided as Supplementary Data 7. All other data supporting the findings of this study are available from the corresponding author upon request. Source data are provided with this paper.

## Source data

Source Data file

## Code availability

Custom scripts employed for the analysis of the sequencing data for L1-L11 are available at https://github.com/vakh57/bdelloid_scripts. The scripts provided in this repository are licensed under the GNU General Public License v3.0.
