# Supplementary material for: Genomic signatures of recombination in a natural population of the bdelloid rotifer Adineta vaga
Source: Nat Commun. 2020 Dec 18;11:6421. doi: 10.1038/s41467-020-19614-y (PMC7749112; doi:10.1038/s41467-020-19614-y)
Supplement: Supplementary file 1 — Supplementary Information [file 41467_2020_19614_MOESM1_ESM.pdf]

Supplementary Information

Genomic signatures of recombination in a natural population of the bdelloid rotifer *Adineta vaga*

Vakhrusheva *et al*.

Table of contents

Supplementary Methods 3
Supplementary Notes 17
Supplementary Discussion 42
Supplementary Figures 45
Supplementary Tables 78
Supplementary References 90

Supplementary Information also includes Supplementary Data 1–9 supplied as separate files.

Supplementary Methods

I. Obtaining PacBio reads for L1 and assessing the accuracy of the *A. vaga* L1 genome assembly

To assess the accuracy of L1 MiSeq-based genome assembly and HiSeq-based haplotype phasing, we obtained PacBio reads for L1, using the independent replicate of L1 culture reared in Marine Biological Laboratory, Woods Hole, USA. For PacBio library, DNA was extracted from rotifer eggs, and a 20-kb library was constructed using BluePippin selection to sequence 15 SMRT cells on a PacBio RS II sequencer (Pacific Biosciences) at the Johns Hopkins University Deep Sequencing and Microarray Core facility with P6-C4 chemistry (accession number PRJNA558051).

We evaluated the concordance between the PacBio reads and the L1 diploid assembly by mapping the reads of insert (ROI) to the L1 unfiltered contigs using the RS_ReadsOfInsert_Mapping.1 protocol on the SMRT Analysis Portal (v2.3.0). Briefly, reads of insert are generated from the consensus sequence determined using subreads, regardless of the number of polymerase passes. Mapping of PacBio reads (filtering parameters: minimum full passes = 1, minimum predicted accuracy = 90%) was carried out with BLASR (mapping parameters: maximum divergence = 30%, minimum anchor size = 12). Overall, the mean concordance to the assembly was 0.95 (Supplementary Fig. 5) from a total of 12,566 mapped ROI (mapped ROI mean length: 9,726 base pairs [bp]). Note that possible causes of 5% discordance (in addition to assembly inaccuracies) may include: (i) PacBio sequencing errors remaining in reads of insert, and (ii) alignment of reads to both haplotypes present in the L1 diploid assembly.

II. BUSCO analysis

To assess the completeness of the *A. vaga* L1 genome assembly and to compare it with the previously published bdelloid genomes[1,2], we conducted BUSCO (v. 3.1.0)[3] analysis using eukaryotic and metazoan datasets (versions eukaryota_odb9 and metazoa_odb9). Out of 303 eukaryotic and 978 metazoan Benchmarking Universal Single-Copy Orthologs (BUSCOs), only 2.6% (n = 8) and 8.3% (n = 81) respectively were not detected in the L1 genome assembly. L1 assembly has 92.1% (n = 279) eukaryotic and 88.9% (n = 869) metazoan BUSCOs identified as complete. As is shown in Supplementary Figs. 6 and 7, in terms of completeness and distribution of BUSCO categories, L1 assembly is very similar to the previously reported bdelloid genomes of *A. vaga*[1] and *A. ricciae*[2]. However, as compared to these genome assemblies, L1 assembly displayed a slightly increased proportion of fragmented genes, most probably due to lower N50.

III. Construction of non-redundant haploid subset of the *A. vaga* genome (haploid sub-assembly)

Due to high heterozygosity, the two haplotypes of the *A. vaga* genome assemble into separate contigs at the majority of loci[1]. Still, in a substantial portion of the genome, the two haplotypes collapse into a single contig, leading to a mosaic organization of the assembly with alternating ploidy levels. This complicates application of standard variant calling and population genomics methods that presume uniform ploidy across all loci and are mainly targeted at diploid variant calls made

against a haploid assembly. To overcome this difficulty, we obtained a reduced haploid representation of the *A. vaga* genome[4].

To retrieve a haploid subset of the L1 assembly, we first searched for the pairs of highly similar genomic segments within the assembly likely corresponding to two haplotypes. After assigning some haplotype segments to such pairs, we retained only a single segment from a pair and discarded genomic regions without haplotypic counterparts. This procedure aims at reducing redundancy of the assembly, while simultaneously ensuring that only truly diploid loci are included into the haploid representation.

To achieve this, for each contig, we identified the subset of the assembly likely containing its haplotypic counterparts. We started by carrying out all-versus-all BLAST[5] search of the filtered set of contigs within the assembly. BLAST searches were performed with blastn from BLAST+ (version 2.2.31) with the following parameters: -evalue 1e-10 -outfmt "6 qseqid sseqid pident length mismatch gapopen qstart qend sstart send evalue bitscore qlen slen" -task dc-megablast -max_hsps 1.

Next, for each contig we selected those among the remaining contigs that had regions of high similarity to it (at least one blastn dc-megablast alignment with the considered contig with E-value $\leq 1\times10^{-50}$ and alignment percent identity $\geq$ 90%). We used all_bz (v.15)[6] to create pairwise blastz[7] alignments between each contig and its counterparts. The initial blastz output was further processed with single_cov2 to remove secondary alignments in the contig regions where a hit to more than one other contig region was found. This resulted, for each contig, in a set of pairwise alignments such that each position of the contig was aligned to no more than one position of the matched contigs. Thus, the resulting set of alignments for a contig could be viewed as the set of best BLAST hits between it and the rest of the assembly.

To extract only those pairs of genomic segments that are reciprocal best matches, we devised a procedure analogous to identifying reciprocal best BLAST hits among genes. For each alignment between contig A and contig B belonging to the set of best matching alignments of contig A (let us denote it by 'forward alignment'), we determine if there is a corresponding alignment between contig B and contig A among the set of best matching alignments of contig B ('reverse alignment'). We retain the pair of the aligned segments if the coordinates of the forward and reverse alignments are identical or if their shared span covers ≥ 80% of the longer alignment. In the latter case, the boundaries of the reciprocally best matching segments were defined as the overlap between the forward and reverse alignments.

The resulting set of reciprocally best matching genomic segments is likely to represent pairs of haplotypes. To obtain a non-redundant haploid subset of the assembly, from each such pair of best matching segments, we selected the one from the longer contig, and discarded the remaining one. To increase continuity of the haploid sub-assembly, if a pair of non-redundant haploid segments was located on the same contig and separated by ≤ 200 bp, we took a union of such segments. The summary statistics for the resulting haploid sub-assembly are shown in Supplementary Table 3.

The final haploid sub-assembly[4] spanned 76,098,573 bp in haploid segments (further also referred to as 'haploid contigs') ≥ 500 bp in length (Supplementary Table 3), suggesting that ~77% of the original assembly corresponds to loci represented in the assembly by two haplotypes.

IV. Annotation of protein-coding genes

We predicted protein-coding genes in the filtered diploid assembly of the *A. vaga* L1 genome using AUGUSTUS (v.2.7)[8] and GeneMark.ES Suite (version 4.32)[9]. Intron and transcribed region hints for AUGUSTUS were prepared with STAR aligner (v. 2.4.2a)[10]. For this purpose, RNA-seq reads available at http://www.genoscope.cns.fr/adineta/data/Avaga_rnaseq_sort.bam were mapped on the L1 diploid assembly with strict mapping parameters. The list of putative splice junctions (a total of 119,058 suggested intron boundaries) was obtained taking into account only uniquely mapped reads (16% of the available RNA-seq reads, 21 million reads). Coverage profile for all mapped reads (+23% of reads that mapped to multiple loci, 61 million of total mapped reads) was assessed using alignments produced by STAR. To compile the final set of intron and exonpart hints for AUGUSTUS, we combined predictions of splice junctions with the coverage profile and filtered poorly supported junctions (splice junction support cut-off > 1 unique reads, exonpart support cut-off > 5X coverage). GeneMark.ES was run to obtain a set of initial gene predictions. These predictions were ranked by blastp alignment quality (*E*-value, query coverage, gaps, positives), and 500 top-scoring models were used to generate the training set for AUGUSTUS. Species model was trained and then used to generate the final hinted predictions with AUGUSTUS. All predicted models (from AUGUSTUS and from GeneMark.ES) were combined together and scored according to the blastp alignment score and support from RNA-seq reads. To get rid of chimeric genes and mispredicted gene fragments, best gene models were selected at each locus. The initial set of predictions comprised 78,303 gene models originating from 75,877 loci. We separately ran BUSCO analysis on this initial set of gene models and found that all eukaryotic BUSCO groups detected within the L1 genome assembly were also present in the set of predicted genes, confirming the completeness of the annotation.

We performed a quality check on the initial gene predictions, discarding gene models with putative annotation errors and those likely corresponding to incomplete genes. First, we removed annotations at the contig boundaries, which left us with 72,406 out of 78,303 gene models. Next, we checked coding sequence (CDS) regions of each gene model, excluding a gene model from further analyses if the length of the corresponding CDS was not in multiple of three (n = 6,919).

We further filtered out gene models if the corresponding CDS carried a premature stop codon (n = 691) or was lacking a canonical termination codon (n = 1,426). A total of 63,370 gene models originating from 61,531 loci (genes) remained after these filtering steps. For each locus, the longest transcript among those that passed all filters was retained, providing a total of 61,531 gene models which were used in downstream analyses.

This number is higher than that reported by Flot *et al.* in 2013 for the first published genome assembly of *A. vaga* (n = 49,300)[1]. Most probably this difference is due to different algorithms used to predict genes (AUGUSTUS employed in the current study versus GAZE used by Flot *et al.*) and different gene filtering strategies. Another estimate of the number of *A. vaga* genes obtained by Nowell *et al.*[2] was quite different from that reported by Flot *et al.*: reannotation of the 2013 *A. vaga* genome assembly carried out by Nowell *et al.* yielded 67,364 genes (57,431 high-quality CDS)[2]. The number of gene models predicted for *A. vaga* in the current study is similar to that reported by Nowell *et al.* The pipeline employed by Nowell *et al.*[2] also involved AUGUSTUS making the choice of the gene prediction software a likely explanation for the discrepancies in the number of genes between different studies. To further compare the annotation of protein-coding genes produced in the current study

to the set of predictions generated previously for the 2013 *A. vaga* genome, we computed different annotation metrics (Supplementary Table 4). This analysis showed that in terms of characteristic CDS length, intron length and number of introns per gene our set of gene predictions is very similar to that reported earlier for the first published *A. vaga* genome[2].

We transferred the filtered gene models predicted in the L1 diploid assembly to the coordinate system of the L1 haploid sub-assembly, retaining only those gene models that were fully contained within the haploid segments. Gene models partially overlapping with haploid segments were discarded. This procedure yielded 23,802 gene models with coordinates mapped to the coordinate system of the haploid sub-assembly.

V. Identification of allelic regions and allelic genes

To test the robustness of our results against erroneous identification of haplotype pairs, we separately analyzed the fraction of the haploid sub-assembly covered by long blocks of genes that are collinear between the two haplotypes in the diploid genome.

To obtain a subset of the *A. vaga* genome with high-confidence ploidy, we identified genomic regions that could be assigned into pairs of highly similar segments with conserved gene order. We initially searched for collinear groups of genes within the assembled *A. vaga* L1 reference genome. For this, we first ran an all-versus-all blastp search of the proteins predicted in the *A. vaga* L1 genome (carried out with BLAST+ 2.2.31). BLAST results were restricted to hits with E-value $\leq 1\times10^{-10}$ with the maximum number of target sequences to output per query sequence set to 5, and self-to-self hits were discarded. Next, to identify collinear groups of genes, we ran MCScanX[11] (available at http://chibba.pgml.uga.edu/mcscan2/MCScanX.zip; accessed August 28, 2017) on the output of blastp with an E-value cut-off of $\leq 1\times10^{-5}$. This resulted in a total of 1,770 detected syntenic blocks.

Next, for each syntenic block (out of 1,769 blocks remaining after excluding one block formed by two genomic regions located on the same contig), we calculated the fraction of collinear genes and the average value of Ks (see below). The number of synonymous substitutions per synonymous site (Ks), as well as the number of nonsynonymous substitutions per nonsynonymous site (Ka), for each matched pair of collinear genes within the block was computed using the script add_ka_and_ks_to_collinearity.pl distributed as a part of the MCScanX package. The average value of Ks for a syntenic block was computed across those pairs of collinear genes for which both Ks and Ka were greater than 0 and less than 1. The average Ks values were rounded to two decimal places. The fraction of collinear genes for a block was computed as the number of collinear gene pairs divided by the maximum number of genes between two genomic regions forming a collinear block.

Presence of gene duplications might inflate the total number of genes covered by a collinear block and cause a downward bias in the estimated fractions of collinear genes. To account for this, we subtracted the total number of tandem duplications identified by MCScanX within each genomic region from the total number of genes covered by the considered region.

Distribution of average Ks values per collinear block versus fractions of collinear genes revealed two clearly distinguishable groups of blocks (Fig. 1b). The group with high fractions of collinear genes and low average values of Ks (Fig. 1b,

blue dots) is likely to correspond to pairs of haplotypes, while the other group exhibiting lower extent of collinearity and higher synonymous divergence (Fig. 1b, red dots) most probably stems from an ancient whole-genome duplication. Such genome organization confirms the same patterns of tetraploidy that have already been reported for the first published genome of *A. vaga*[1].

To focus on the genomic regions for which the ploidy could be inferred with high certainty, we extracted a subset of the collinear blocks with a high degree of collinearity and low synonymous divergence (hereafter referred to as 'allelic regions'). These are the regions that are most likely to be represented in the assembly by two haplotypes. We delineate the allelic regions as a subset of genomic segments possessing a within-genome counterpart with a high fraction of collinear genes (fraction of collinear genes in a block ≥ 0.7) and low average values of Ks (average Ks ≤ 0.2) (Fig. 1b). A total of 1,387 collinear blocks satisfied these criteria, with 1,354 blocks remaining after removal of the conflicting synteny segments encompassing overlapping genomic regions. In addition to the subset of allelic regions, we specify the subset of the genes embedded in allelic regions (hereafter referred to as 'allelic genes'). The initial subset of allelic genes was composed of 12,489 collinear gene pairs residing within the allelic regions and filtered for the individual values of Ks (Ks ≤ 0.2; only those gene pairs for which both Ks and Ka were greater than 0 and less than 1 were considered).

To delineate haploid equivalent of the allelic regions, for each of the 1,354 pairs of collinear allelic segments, we left only a single segment, retaining the one from a longer contig in a pair and discarding its counterpart. This non-redundant subset of unique non-overlapping allelic regions spanned 34,691,452 base pairs.

Having thus obtained a non-redundant haploid representation of regions with high-confidence ploidy, we mapped it into the coordinate system of the original haploid segments identified in the previous step (see section «Construction of non-redundant haploid subset of the *A. vaga* genome» of Supplementary Methods). We retained only those allelic segments that were fully contained within boundaries of the original haploid segments; those with partial overlaps were discarded. A total of 833 allelic segments spanning 19,300,566 base pairs and encompassing 7,245 allelic genes remained after this step. We used these final subregions of the haploid segments throughout the paper as portions of the genome with high-confidence ploidy. The designations 'allelic regions' and 'allelic genes' in the main text of the paper refer to these final sets of 833 regions and 7,245 genes respectively.

VI. Mapping of Illumina reads

We aligned adapter- and quality-trimmed Illumina HiSeq paired-end reads (2×98 bp, 2×100 bp or 2×101 bp before trimming) generated for each sequenced individual to:

1) Original filtered diploid contigs.

2) Non-redundant haploid segments (see section «Construction of non-redundant haploid subset of the *A. vaga* genome» of Supplementary Methods).

Alignments of reads to the diploid contigs were used to filter out ambiguously mapping reads, prior to performing the alignment of reads to the haploid sub-assembly. Actual identification of variable sites was performed on read alignments to the haploid sub-assembly, which allowed us to assume diploid samples during variant calling.

We mapped reads with Bowtie 2 (version 2.3.2)[12]. The choice of this aligner was motivated by its ability to find global end-to-end alignments of reads to the reference genome. This is advantageous compared to local read alignment when dealing with the genome rich in repetitive sequences as is the case with genomes of bdelloid rotifers bearing remnants of a whole-genome duplication[1].

First, we mapped trimmed reads from each individual to the original filtered L1 contigs using Bowtie 2 with the parameters "--no-mixed --no-discordant" setting the maximum insert size to 800 bp and allowing to report up to 5 distinct alignments. The overall alignment rates for different individuals ranged from 74.29% to 93.43%.

Assuming that most genomic loci in the L1 assembly are represented by two haplotypes, we would expect no more than two 'true' alignments per read pair. Those reads mapping to more than two genomic locations are likely to produce spurious alignments to paralogous regions.

To avoid using erroneous alignments for variant identification, which could result in false positive variant calls, we removed reads mapping to more than two loci in the L1 diploid assembly prior to aligning the reads to the haploid sub-assembly. For this purpose, for each pair of reads, we tabulated the number of properly paired alignments in the L1 diploid assembly, leaving only reads that were mapped in a proper pair to one or two locations.

For each sequenced individual, subsets of reads that passed this filtering step were in turn remapped to the non-redundant haploid segments. Alignment of the filtered subsets of reads against the haploid sub-assembly was performed with Bowtie 2 with the parameters "--no-mixed --no-discordant" specifying the maximum insert size of 800 bp with only the best alignment of the pair of reads reported.

To further reduce the number of erroneous mappings, we parsed SAM files with the reads mapped against the haploid sub-assembly and removed alignments of reads for which more than one valid alignment in the haploid genome was found (those with XS tag set). We filtered out alignments of both paired-end reads, irrespective of whether a secondary alignment was found for a single read or for both reads forming the pair.

We used SAMtools (v.1.4.1)[13] to convert the filtered SAM files to sorted BAM files and perform additional filtering on the mapping quality (MAPQ) retaining only those reads that have MAPQ $\geq$ 20. The resulting BAM files with paired-end alignments left after the above-described filtering steps were used for variant calling.

VII. Variant calling and filtering

Throughout the analyses, we use two main genotypic data sets. SNP dataset I includes SNP calls for sites variable among the individuals L1-L11. SNP dataset II comprises calls for both variable and invariant sites.

A stringently filtered subset of the SNP dataset I was used for local haplotype reconstruction via read-based phasing. We devised SNP filtering approach for this dataset in such a way as to maximally reduce the percentage of false positive variant calls which can potentially lead to phasing errors. For this, prior to performing read-based phasing, we removed from the SNP dataset I all sites with more than two nucleotides present in the aligned reads, even if some of the nucleotides did not occur in any of the genotypes. For these purposes, all nucleotides present in the aligned reads were treated as putative alleles in the process of variant calling, regardless of whether they were supported by any of the called genotypes.

Conversely, the SNP dataset II was primarily intended for a survey of triallelic SNPs and computing the pairwise genotypic distances. Treating all nucleotides at a particular site present in reads but not appearing in the resulting genotypes as alleles might lead to erroneous classification of sites with respect to the number of alleles. Therefore, for the purposes of building the SNP dataset II, to minimize the number of sites misidentified as being triallelic due to sequencing errors, only those nucleotides at a given site supported by at least one of the genotypes were regarded as alleles.

Single-nucleotide variants were called from read alignments to the haploid sub-assembly. As a result, we were calling diploid variants, because homologous sites of both haplotypes were aligned to the same site of the sub-assembly. Genotype calls in both datasets were generated using the SAMtools[13] mpileup utility (v.1.4.1) with the parameters "-aa -u -t DP,AD,ADF,ADR" followed by the command "bcftools call" with the "-m" option. To identify all alleles present in the reads, including those potentially absent from called genotypes, and to skip invariant sites, "bcftools call" was run with the additional parameters "-A" and "-v". These additional parameters were employed to produce genotype calls included in the SNP dataset I.

Next, we performed stringent filtering of the obtained raw genotype calls. We successively applied a series of filters, removing sites falling into one or more of the following categories from the datasets:

1) SNPs residing within 10 bp of an indel.
2) Sites with missing genotypes or QUAL value < 50.
3) Sites located on haploid segments shorter than 1,000 bp.
4) Sites residing in repetitive regions.*
5) Sites with low coverage (DP < 10 in any of the samples).
6) Sites with extremely high depth of coverage.**
7) Sites residing within the windows outliers for SNP density.***

*Annotation of repetitive regions in the haploid sub-assembly of the *A. vaga* genome was carried out with RepeatMasker (version open-4.0.7, http://www.repeatmasker.org/).

**For SNP dataset I, which includes only variable sites, we removed from further consideration sites identified as being outliers with respect to the mean coverage across 11 individuals, the total coverage summed across 11 individuals or individual coverage values as determined for each sample separately. Identification of outliers was performed using the interquartile range method in R (version 3.3.2). For SNP dataset II, we discarded all sites with depth of coverage DP > 300 in any of the individuals.

***Genomic regions with unusually high densities of variable positions are likely to stem from reads mapping to paralogous regions. To avoid using false positive variant calls resulting from misalignment of such reads, we searched for outlier regions with respect to SNP density and discarded variants falling within such regions. For this purpose, we conducted a sliding window analysis (using a window length of 1,000 bp and a step size of 500 bp) of the *A. vaga* haploid sub-assembly, computing fractions of variable sites based on the SNP dataset I in each window. Detection of outliers was performed using the interquartile range method in R (version 3.3.2).

Filtering was carried out using combinations of BCFtools (v.1.4.1, https://samtools.github.io/bcftools/), VCFtools (v. 0.1.15)[14], bedtools (v2.26.0)[15], and SnpSift (v.4.3s)[16] utilities.

The total numbers of sites in the raw SNP datasets and the numbers of sites remaining after successive application of various filters are listed in Supplementary Table 5. The final subsets of the SNP dataset I and II that passed all the filters are further referred to as the stringent SNP datasets I and II respectively.

To assess the reliability of the resulting SNP calls[17] (SNP dataset I), we compared SNPs identified with SAMtools to SNPs called for the individuals L1-L11 with GATK[18] using two versions of the HaplotypeCaller (3.5 and 4.1.2.0). Sets of SNP calls produced with the two versions of HaplotypeCaller under default parameters displayed 97% consistency (indels were not considered). The set of SNPs generated with the more recent version (4.1.2.0) of GATK was further used to estimate concordance between the SAMtools- and GATK-called SNPs. On average, 88.6% of raw SNP calls from the SNP dataset I (n = 3,318,352) generated with SAMtools for a particular individual, L1-L11, were identically called with GATK (Supplementary Table 6). This rate of SNP recovery is similar to that reported for different variant callers on human data[19,20]. Importantly, after filtering the fraction of SAMtools-called SNPs recovered with GATK substantially increased: in the stringent SNP dataset I (n = 2,282,099), the average proportion of SNPs identically called with GATK for a particular individual is 94.8% (Supplementary Table 6). This indicates that the employed filtering indeed resulted in reduction of the proportion of low-confidence SNP calls in the dataset.

However, we noticed that when applied to the SNP dataset II, QUAL 50 filtering based on the BCFtools QUAL field (as in the stringent SNP dataset II) removed disproportionally more invariant than variable sites: 97.8% of variable but only 68.8% of invariant sites were retained. Inconsistency in filtering of variable and invariant sites can potentially lead to underestimated proportions of invariant sites in the genomes of analyzed individuals and therefore bias upwards the estimates of heterozygosity and genetic distances. To avoid introducing biases into analyses involving invariant sites, we generated an additional SNP dataset, further referred to as SNP dataset III. SNP dataset III was obtained from the raw SNP dataset II in the same way as the stringent SNP dataset II, with two exceptions: QUAL 20 was used as a threshold value for filtering (leaving similar proportions of variable [99.1%] and invariant [99.7%] sites), and, both variable and invariant sites residing within 10 bp of an indel were excluded. The SNP dataset III contained a total of 58,163,647 sites, 3.93% (n = 2,285,700) and 96.07% (n = 55,877,947) of which were called as variable and invariant among L1-L11 respectively. These proportions were similar to those obtained if filtering based on the QUAL field was completely omitted (3.94% and 96.06% respectively), indicating that QUAL 20 filtering does not asymmetrically remove invariant sites and, therefore, is suitable for the purposes of analyses involving both variable and invariant sites.

VIII. Analysis of population structure

We performed multidimensional scaling (MDS) analysis of identity-by-state (IBS) pairwise distances between the sequenced *A. vaga* individuals with PLINK (v1.90b5.4)[21].

For the MDS analysis, we used a thinned subset of biallelic SNPs from the stringent SNP dataset I with minor allele count ≥ 2. Specifically, a list of biallelic variants with minor allele count ≥ 2 among L1-L11 was thinned in such a way that the resulting dataset did not contain any variants within 1,000 bp of one another. The resulting subset of SNPs (n = 66,483) was retained for the MDS analysis.

Visual inspection of the two-dimensional MDS plot (dimensions 1 and 2) revealed that three individuals (L1, L2, and L3) form a separate group (Fig. 1c).

Next, we inferred a neighbor-joining tree of individuals L1-L11 using a matrix of genetic distances calculated from L1-L11 biallelic SNPs (Supplementary Fig. 8). The neighbor-joining tree is based on another thinned subset of biallelic SNPs (n = 449,218) from the stringent SNP dataset I. For this analysis, we did not exclude singleton variants (unlike for the MDS analysis) and only required that the final dataset used to construct the tree did not contain any variants within 100 bp of one another. The neighbor-joining tree (1,000 bootstrap replicates) was built with the *aboot* function from the R package *poppr*[22,23] (v2.8.6). The tree is based on distances calculated as fractions of alleles different between individuals (*aboot* function invoked with "distance = bitwise.dist"). Note that here fractions of different alleles are computed for biallelic sites (invariant sites are not included), therefore the resulting distances underlying the neighbor-joining tree (Supplementary Fig. 8) are by construction significantly larger than genotypic distances computed taking invariant sites into consideration (see below and Supplementary Table 7).

In line with the MDS analysis, the SNP-based neighbor-joining tree (rooted at the midpoint; Supplementary Fig. 8) revealed subdivision of the sequenced *A. vaga* individuals into two main groups: L1-L3 (hereafter referred to as 'the small cluster') and L4-L11 (hereafter referred to as 'the large cluster'). Accordingly, IBS clustering of the sequenced individuals (performed using PLINK) carried out with the fixed number of clusters set to two resulted in two clusters with the same composition as inferred from the neighbor-joining tree (L1-L3 and L4-L11).

The average pairwise genotypic distance was 1.22% for individuals belonging to different clusters, 0.66% for the three individuals belonging to the small cluster (L1-L3), and 0.54% for the eight individuals belonging to the large cluster (L4-L11; Supplementary Table 7). For a pair of *A. vaga* individuals, the genotypic distance was calculated in the following way: the distance at each assessed genomic site was computed as the difference in the number of non-reference variants (0, 1 or 2), then the resulting values were summed over all analyzed sites and divided by $2n$ (where n = 58,118,767 is the number of analyzed sites; this analysis was based on monomorphic and biallelic sites from the SNP dataset III). Pairwise genotypic distances between individuals were computed using the compute_genotypic_distances.pl script (https://github.com/vakh57/bdelloid_scripts).

Out of the eleven individuals used in the study, nine (L1-L4 and L6-L10) were sampled from the Moscow region and two, L5 and L11, sampled from the Kostroma region, 550 km to the NE. Despite this distance between the two sampling locations, L5 and L11 belong to the large cluster, together with individuals L4 and L6-L10 (Supplementary Fig. 8).

To reduce the potential effect of population structure, we focused most of the subsequent analyses on the large cluster. If not indicated otherwise, the reported results are based on the analysis of this cluster.

IX. Estimating heterozygosity in genomes of the sequenced *A. vaga* individuals

For each individual, we computed a proportion of heterozygous sites using sites from the SNP dataset III (see section «Variant calling and filtering» of Supplementary Methods). This dataset includes both variable and invariant sites that were simultaneously called in all individuals L1-L11 and passed multiple filtering

steps (n = 58,163,647). We further removed sites where more than two nucleotides were present in the aligned reads from a single individual and each was supported by more than one read. 58,158,930 sites retained after this step were used to assess whole-genome levels of heterozygosity in L1-L11. We separately assessed levels of heterozygosity at silent (four-fold degenerate, n = 3,612,576) and replacement (zero-fold degenerate, n = 14,099,199) sites (Supplementary Data 2). Identification of silent and replacement sites was carried out relative to the L1 haplotype present in the haploid sub-assembly. Conceivably, sites annotated as zero-fold degenerate relative to one of the two haplotypes are not necessarily zero-fold degenerate relative to the other haplotype. To avoid this ambiguity, we regarded as zero-fold degenerate only those sites at which 4 different nucleotides corresponded to 4 different amino acids.

We also computed proportions of heterozygous sites for each individual in 5 kb non-overlapping windows. The analysis is based on the haploid contigs containing at least one complete 5 kb window. Only those windows in which no less than 60% of sites were simultaneously called in all individuals L1-L11 were used (n = 10,349). Density plots (Supplementary Fig. 9) were created with ggplot2 (https://ggplot2.tidyverse.org).

X. Computational phasing of genotypes

We performed computational phasing of genotypes using biallelic SNPs from the stringent SNP dataset I (n = 1,774,991) and the strictly filtered alignments of reads to the haploid sub-assembly (see sections «Mapping of Illumina reads» and «Variant calling and filtering» of Supplementary Methods). To mitigate the impact of sequencing errors on haplotype reconstruction, we applied stringent criteria for inclusion of SNPs in the dataset subjected to phasing, discarding all sites with more than two different nucleotides present in the aligned reads across the individuals L1-L11. Local haplotypes were assembled for each sample L1-L11 individually, using HapCUT2[24] (revision bd1a739, https://github.com/vibansal/HapCUT2) with the "--error_analysis_mode 1" option to compute switch error scores.

Phased haplotype blocks were aggressively filtered before being used for subsequent analyses. The logic behind the main filtering step is that each individual can carry no more than two different haplotypes for a pair of SNP sites. Those pairs of sites with support for more than two 'haplotypes' in the aligned reads from a single individual are likely to stem from PCR template switches[25] or from paralogous alignments and other artifacts and to be associated with phasing errors. We discarded phased blocks encompassing such sites prior to the analysis, as their presence might create artifactual evidence for LD decay.

For this purpose, we parsed fragment matrix files generated by HapCUT2 for each individual, L1-L11, and extracted information on the haplotypes supported by reads for each pair of SNPs phased in a given individual. We designated pairs of SNPs represented by more than two 'haplotypes' in the aligned reads from a single individual as 'conflicting'. Most such cases with the third (least frequent) 'haplotype' supported only by a single read are likely to have originated from single nucleotide sequencing errors or from PCR template switches recovered only in a single read. Thus, we narrowed the list of the conflicting SNP pairs down to those present in reads as three distinct haplotypes each supported at least by two reads. We also regarded as 'conflicting' all SNP pairs represented by four haplotypes in a single individual irrespective of the number of reads supporting different 'haplotype' variants. Having obtained lists of conflicting SNP pairs for each individual, we removed phased blocks

encompassing such SNPs from further consideration. Note that a significant fraction of PCR template switches is likely to be filtered out at this step, as PCR template switches are expected to be present in the reads from a single individual as alternative 'haplotypes' at low read counts[25].

Phased blocks remaining after applying this filter were used as the main phased dataset (further referred to as 'phased dataset 1'). Statistics on the lengths of phased blocks included in the phased dataset 1 for different individuals and on the numbers of variants spanned by such blocks are provided in Supplementary Table 9 and Supplementary Table 10.

To ensure that LD decay cannot be explained solely by phasing errors and to see whether the patterns in LD decay depend on the stringency of filtering criteria, we also obtained a more strictly filtered phased dataset ('phased dataset 2'). For this purpose, in addition to removing phased blocks with conflicting pairs of SNPs, we subjected sets of locally phased haplotypes to further filtering. We used phred-scaled estimated probabilities of switch errors and mismatches generated by HapCUT2. For each phased block left after removal of blocks with conflicting pairs of SNPs, we considered SNPs with values of switch or mismatch quality < 100 as problematic. All blocks comprising more than one problematic SNP were completely discarded from the dataset. Blocks with a single problematic SNP were split at the corresponding site, and the chunks of the original block resulting from the split were analyzed separately. Detection of conflicting SNP pairs and subsequent filtering of HapCUT2 output files was conducted using the get_conflicting_variants_indices.pl and filter_hapcut2_haplotype_blocks.pl scripts respectively (https://github.com/vakh57/bdelloid_scripts).

For both filtered datasets, the resulting files with the phased blocks in the HapCUT2 format were converted to VCF format using the utility HapCutToVcf from fgbio (version 0.2.0-SNAPSHOT, http://fulcrumgenomics.github.io/fgbio/).

For each individual, HapCUT2 assigns to haplotypes only those SNPs at which that individual is heterozygous; consequently, all homozygous sites are omitted from the output. However, sites that are in a homozygous state in some individuals may occur in a heterozygous state in other individuals. Therefore, data on the homozygous sites are essential when exploring haplotypic data across several individuals simultaneously. To complement the phased haplotype blocks with the data on SNPs at which a given individual is homozygous, we searched for cases where a homozygous site is embedded within a phased block. For this, for each homozygous site, we identified closest flanking SNPs at which the individual in question is heterozygous. We regarded a homozygous SNP as embedded in a phased block, if both its left and right closest heterozygous SNPs were phased and belonged to the same phased block. In this case, we assigned the homozygous SNP to the block encompassing its heterozygous neighbors, assuming that both haplotypes carry the same variant.

After adding 'phasing' information for the homozygous variants, we further processed the VCF files and identified haplotype blocks nested within other blocks, removing such cases from the analysis.

Next, we identified genomic segments encompassing groups of variable sites where genotypes for all the individuals L4-L11, or for all the individuals L1-L11 are simultaneously phased. In the text of the paper, we refer to such genomic segments harboring at least two sites simultaneously phased in L4-L11 or in L1-L11 as 'phased genomic segments'. For each such phased genomic segment, we extracted the corresponding portion of the VCF file into a separate VCF file using awk (version of

awk 3.1.7). We also obtained subsets of the variants belonging to individual phased genomic segments applying different thresholds on a minor allele count among individuals L4-L11 or L1-L11 using BCFtools (v.1.4.1, https://samtools.github.io/bcftools/).

For the purposes of calculating r^2 values and other LD-related analyses, groups of variants representing different phased genomic segments were processed separately.

XI. Analysis of linkage disequilibrium (LD)

Assessing LD decay from the phased haplotype data

Using the phased haplotype data, we calculated r^2 values (for SNP pairs residing within the same phased segment) individually for each phased segment with VCFtools (version 0.1.15)[14]. If not stated otherwise, the reported results are based on the analysis of SNPs from the phased dataset 1 (Fig. 2a, Supplementary Figs. 11, 13a and 13c). For this analysis, we additionally excluded all sites which were likely to be falsely called as homozygous in some individuals. For this purpose, for each individual, we looked for sites which were called as homozygous but were nevertheless represented in the aligned reads from this individual by two nucleotides, each supported by at least two reads. Such sites were excluded from analysis in all individuals. The reported results are for variants with a minor allele count (MAC) of at least 4 among individuals L4-L11 or L1-L11. The results obtained for the more severely filtered phased dataset 2 were qualitatively similar (Supplementary Fig. 13b). We also recapitulated the main findings on the subset of those SNPs from the phased dataset 1 that reside within the allelic regions of the *A. vaga* genome (Supplementary Fig. 13a).

To determine the baseline r^2 values, we computed r^2 for sites residing on different contigs in the original L1 diploid assembly. If the total number of site pairs from different contigs in the dataset exceeded 10,000,000, we thinned the dataset by randomly drawing 10,000,000 pairs of sites. In this case, the displayed distributions of inter-contig r^2 values and the corresponding mean and median r^2 values are based on the thinned datasets.

The decay of LD (expressed as r^2) with physical distance (expressed in base pairs) was fitted using second-degree LOESS regression with the smoothing parameter set to 0.4 as implemented in the *geom_smooth* function from the ggplot2 package (version 3.2.1) and the *loess* function from the stats package (version 3.6.3) in R. LD decay among L4-L11 based on the phased dataset 1 (the same data as in Fig. 2a) was also fitted by first- and second degree LOESS with the smoothing parameter selected according to the bias-corrected Akaike information criterion (the *loess.as* function from the fANCOVA R package [version 0.5-1]; Supplementary Fig. 11). We also estimated the rate of short-range decay of r^2 with physical distance by applying nonlinear regression based on mutation-recombination-drift model (see section «Estimation of the population-scaled recombination rate» of Supplementary Note 10).

Assessing LD decay from the unphased genotype data

To make sure that the observed LD decay was not an artifact of phasing, we assessed LD decay directly from the unphased genotype data by using two approaches. The first approach is based on inferring haplotypes on the basis of variable homozygous sites. The rationale behind is as follows. For each individual, it

is possible to determine haplotypes for sites at which this individual is homozygous, as phase of homozygous SNPs on the same contig is already 'known'. We make use of this by comparing haplotypes of variable sites at which each individual is nonetheless homozygous. For this purpose, we selected biallelic sites variable among individuals L4-L11 such that each individual is homozygous at each site (n = 18,995). We further filtered out sites with the least frequent genotype private to a single individual, leaving only those sites where each of two homozygous genotypes (0/0 and 1/1) was present at least in two individuals (n = 3,410). This requirement automatically filters out variants with minor allele count below 4. To retain only truly homozygous genotypes, we excluded all sites at which more than one nucleotide occurred in reads in any single individual. We also required genotypes to be simultaneously supported by forward and reverse reads in all individuals. These filters resulted in the final set of 2,573 variable sites. We converted GT field of such sites in the VCF file to the format of a phased genotype (0/0 → 0|0; 1/1 → 1|1) and used the resulting VCF file containing 2,573 sites to compute r^2 values with VCFtools (Fig. 2b).

The second approach to inferring LD decay from the unphased genotype data relies on calculation of squared correlation coefficients between genotypes using VCFtools[14] command --geno-r2 (https://vcftools.github.io/man_latest.html)[26]. This command computes the same unphased LD measure as PLINK[21,26]. Namely, for each pair of SNPs, it gives the squared correlation coefficient between numbers of non-reference variants (which could be 0, 1 or 2) at two corresponding sites in the considered individuals. Note that each genotyped genomic site could be represented by a vector of length n, where n is equal to the number of individuals and the i-th element of a vector represents a genotype (0, 1 or 2) of the i-th individual. Therefore, squared correlation coefficients could be computed for a pair of sites, each encoded as a vector of genotypes. As previously, sites that were likely to be falsely called as homozygous in some individuals were not considered. This analysis was also carried out for variants with MAC ≥ 4 among individuals L4-L11. Squared correlation coefficients were computed for comparisons of 10,000 randomly drawn biallelic sites versus the rest of the biallelic sites using VCFtools (v. 0.1.15)[14]. SNP pairs were binned according to the distance separating the pair at resolution of 200 base pairs and the mean squared correlation coefficient between genotypes was determined for each bin. Fig. 2c shows only bins with SNPs at a distance of ≤ 4,000 base pairs. 95% bootstrap percentile confidence intervals for the mean genotypic correlation coefficient at different distances were derived from 1,000 bootstrap replicates using functions *boot* and *boot.ci* from the boot package (version 1.3.24) in R.

Estimating correlation of zygosity (Δ)

As an alternative approach to assess dependence of LD on physical distance, we compared the extent of correlation of zygosity (Δ) between pairs of sites at different distances. Δ is a measure reflecting non-independence between loci[27,28] which could be assessed from a genome of a single diploid individual. The relationship between Δ and physical distance is expected to mirror the relationship between conventional measures of LD and distance[29,30]. We obtained maximum likelihood estimates of Δ at different distances using the method proposed by M. Lynch[27] and implemented in the program mlRho[28]. Estimates of Δ for each individual L1-L11 were obtained by supplying mlRho (version 2.9) with nucleotide counts observed at genomic sites covered by no less than 20 reads. Plots displaying relationship between Δ and physical distance for one individual from the small cluster

(L1) and three individuals from the large cluster (L4, L7 and L11) are shown in Supplementary Fig. 15.

XII. Signatures of recombination within individual phased genomic regions

We explored signatures of recombination within the individual phased genomic regions by applying two permutation tests to the segments of the *A. vaga* genome harboring at least 15 non-singleton SNPs simultaneously phased in all the L4-L11 individuals. A total of 434 segments that satisfy these conditions were distributed between 352 contigs belonging to the original L1 diploid assembly. For this analysis, we additionally excluded all sites that were likely to be falsely called as homozygous in some individuals. For this purpose, for each individual, we looked for sites which were called as homozygous but were nevertheless represented in the aligned reads from this individual by two nucleotides, each supported by at least two reads. Such sites were excluded from analysis in all individuals.

First, for each such segment we assessed whether the decay of r^2 is significantly correlated with physical distance[31]. Then, we performed the sum of distances test[32], assessing whether the sum of distances between variable sites harboring all four possible haplotypes is significantly larger than that expected by chance based on the value of the statistic in the permuted data. Both tests were carried out using LDhat (version 2.2)[33], and the one-sided *P* value for each considered segment was obtained from 10,000 permutations.

We also tested for recombination applying pairwise homoplasy index[34] (PHI) test as implemented in the PhiPack (available at http://www.maths.otago.ac.nz/~dbryant/software/PhiPack.tar.gz; accessed July 1, 2018) to the same set of 434 segments. The window size for computing the PHI statistic was set to 100 nucleotides, and significance was assessed under the assumption of a normal distribution of the PHI statistic. Split decomposition networks of the selected genomic segments for which results of the PHI test remained significant after applying the Bonferroni correction were built and visualized with SplitsTree (version 4.14.6)[35].

Out of the 434 segments, 362 demonstrated significant negative correlation of r^2 with the physical distance at the 0.05 significance level (with 159 remaining significant after correcting for multiple testing). The sum of the distances and PHI tests also suggested the presence of recombination. 296 and 362 segments out of 434 showed evidence for recombination at the 0.05 significance level according to the sum of the distances and the PHI test, respectively, with the results for 108 and 190 segments remaining significant after the Bonferroni correction. Contradictory groupings of different individuals produced by different sets of variable sites were visualized through split decomposition networks constructed for select phased genomic segments (Supplementary Fig. 12).

Supplementary Notes

Supplementary Note 1: Inferring phylogeny of the individuals L1-L11 and reference bdelloid isolates for the *COX1* gene

We confirmed species identity of the sequenced individuals L1-L11 using mitochondrial marker-based phylogeny. For this, we inferred maximum likelihood phylogeny of the *COX1* gene for the individuals L1-L11 and reference isolates identified in the previous works as different bdelloid species.

To extract *COX1*-containing regions for the individuals L1-L11, we used the *COX1* sequence from the first published *A. vaga* genome[1] as a query and carried out blastn search against individual genomes of L1-L11. For this purpose, for each individual L2-L11, we first assembled a genome from the available Illumina HiSeq reads using SPAdes (version 3.6.0)[36]. The resulting genome assemblies for L2-L11 were highly fragmented (with N50 in the range ~1,600–4,300 bp) and as such did not suit the purposes of the main analyses, but allowed to extract *COX1* sequences. For L1, the *COX1* sequence was extracted from the L1 genome assembly based on MiSeq reads (see Methods).

For comparison, we used reference *COX1* sequences from different species belonging to the bdelloid genus *Adineta* from the dataset analyzed in the paper by Fontaneto *et al.*[37] as well as the *COX1* sequence from the published *A. vaga* genome[1]. In addition, we took sequences for several more distantly related bdelloid isolates analyzed in the first paper on genetic exchanges in bdelloids by Signorovitch *et al.*[38] These latter isolates belong to another bdelloid genus *Macrotrachela* (species *M. quadricornifera*).

The sequence of *COX1* present in the published *A. vaga* genome[1] is identical to that available in GenBank under the accession number JX184001.1 (ref. [39]). The accession numbers for reference *COX1* sequences for different *Adineta* isolates analyzed by Fontaneto *et al.* were taken from Table 1 of the corresponding paper[37].

Reference *COX1* sequences were downloaded from Genbank with NCBI efetch command from E-utilities library[40] and aligned with sequences of *COX1* from individuals L1-L11 using MUSCLE (version 3.8.31)[41].

COX1 phylogenies were reconstructed using RAxML (version 8.2.12)[42] with 1,000 bootstrap replicates under the GTR+G model and visualized in Dendroscope (version 3.5.10)[43].

The length of the *COX1* fragment from the first published genome of *A. vaga*[1] and of the corresponding fragments extracted for L1-L11 was 1,542 bp. Length of the reference *COX1* fragments for different *Adineta* isolates (Table 1 in Fontaneto *et al.*)[37] ranged from 332 to 661 bp. The robust maximum likelihood phylogeny inferring method implemented in RAxML allows to find the maximum likelihood tree, however, bootstrap support of the topology for short and gap-rich alignments could be low. Because of this we decided to provide three phylogenetic trees:

- the first tree (Supplementary Fig. 1) shows individuals L1-L11 and a limited number of reference *Adineta* isolates with the longest available *COX1* fragments (including the reference strain sequenced by Flot *et al.* to produce the first *A. vaga* genome assembly[1]). This approach provides high bootstrap support and allows to confirm the placement of the individuals L1-L11 within the *A. vaga* species.

- the second tree (Supplementary Fig. 2) shows individuals L1-L11, the reference *A. vaga* strain[1] and those *Adineta* isolates that were identified to the species level in Fontaneto *et al.* (Table 1 of the corresponding paper)[37].

- the third tree (Supplementary Fig. 3) is an extended version of the second tree. In addition to the isolates shown in the second tree, it also includes *Adineta* isolates with unknown species identity (those referred to as *Adineta* spp. in Fontaneto *et al.*)[37] and isolates of *M. quadricornifera* analyzed by Signorovitch *et al*[38].

GenBank accession numbers for reference *COX1* sequences used in Supplementary Figs. 1-3 are given in Supplementary Data 9.

All three phylogenies confirm that based on the *COX1* marker the individuals L1-L11 are clustered with the reference isolates identified as *A. vaga* in previous works. Interestingly, according to the *COX1* phylogeny, individuals L2-L11 sequenced in the current study and sampled in Russia turned out to be closely related to some reference isolates collected in UK. This is in line with the findings of previous works revealing no obvious geographical clustering among bdelloid isolates[37].

Supplementary Note 2: Assessing accuracy of haplotype phasing

Estimating phasing error rate

We sought to estimate phasing error rate in our data. For this purpose, we compared results of phasing for several *A. vaga* clonal cultures sequenced more than once on different instruments. Among the 11 clonal cultures sequenced in the current study, we sequenced 3 (L1, L2 and L11) on two or more instruments.

Specifically, L1 was sequenced from three independent libraries using Illumina HiSeq, Illumina MiSeq and PacBio sequencing platforms. Libraries were generated from replicates of L1 cultures reared in two different laboratories (Koltzov Institute of Developmental Biology of RAS, Moscow, Russia for Illumina HiSeq and MiSeq, and Marine Biological Laboratory, Woods Hole, USA for PacBio). MiSeq reads were used only for assembly of the *A. vaga* reference genome but not for variant calling; conversely, HiSeq reads were used to produce variant calls but were not included in the assembly. PacBio reads were not included in the primary analyses and were used here exclusively to assess the quality of the assembly and phasing data.

L11 was sequenced from two independent libraries on the Illumina HiSeq and the Illumina MiSeq platforms. L2 was also sequenced on the Illumina HiSeq and the Illumina MiSeq platforms, but the same library was used in both cases. HiSeq reads generated for L2 and L11 were employed for variant calling and downstream analyses, while MiSeq reads obtained for these two cultures were only utilized to estimate the quality of phasing.

Inconsistencies in phasing results inferred for the same individual from different subsets of reads could have different sources. First, they could stem from PCR template switches arising in the process of Illumina library preparation. Since L1 and L11 libraries sequenced on HiSeq and MiSeq instruments were constructed independently, each of the two libraries created for the same individual is expected to possess its own set of PCR template switching products. Second, inconsistencies could result from erroneous mapping of reads to paralogous regions. We would expect MiSeq reads to be less prone to produce spurious alignments due to greater

read length (~250–300 bp before trimming) as compared to HiSeq reads (~100 bp before trimming). Thus, although libraries sequenced on HiSeq and MiSeq instruments are expected to have comparable fractions of PCR template switching products, MiSeq-based haplotype assemblies are likely to be more accurate.

For L2, the same library was sequenced first on the HiSeq and then on the MiSeq platform, and inconsistencies in phasing are probably less likely to be caused by PCR template switches, as pools of sequenced fragments are expected to share to some extent products of the same switching events. However, as it has been shown that PCR template switching tends to occur in late cycles of PCR and its products are usually present at low copy numbers[25], it is conceivable that products of a PCR template switching event could be recovered only in HiSeq or only in MiSeq reads and that some of such cases could possibly contribute to inconsistencies between HiSeq- and MiSeq-based haplotype phases inferred for L2. We note that we did not attempt to estimate how frequently products of the same PCR template switching event were recovered both in HiSeq and MiSeq reads obtained for L2.

We compared the phased blocks recovered from HiSeq reads and used in our main analyses with those recovered from MiSeq (in the case of L1, L2 and L11) or PacBio (in the case of L1) reads. For this, we computationally phased the genotypes with HapCUT2[24] using the same set of biallelic SNPs from the stringent SNP dataset I (n = 1,774,991) which was used to perform phasing from HiSeq reads (for details, see section «Computational phasing of genotypes» of Supplementary Methods). However, this time phasing was based on the alignments of MiSeq or PacBio reads.

Processing of paired-end MiSeq reads

For L1, we used the same set of MiSeq reads which were included in the L1 genome assembly. For L2, we performed two sequencing runs yielding 15,005,814 (2×251 bp) reads and 15,204,556 (2×300 bp) reads, for a total of 18,511,031 reads left after trimming. For L11, we performed two sequencing runs yielding 17,151,928 (2×251 bp) reads and 10,286,070 (2×251 bp) reads, for a total of 21,039,278 reads left after trimming. MiSeq reads were processed analogously to Hiseq reads. Trimmed Illumina MiSeq reads for L1, L2 and L11 were aligned to the haploid sub-assembly (L1) with Bowtie 2 (version 2.3.2)[12] with parameters "--no-mixed --no-discordant" and the maximum insert size of 800 bp. Phasing was performed from end-to-end alignments of those reads that were uniquely mapped in a proper pair to the haploid sub-assembly and had a high mapping quality (MAPQ ≥ 20).

Processing of PacBio reads

For L1, we additionally obtained long reads generated by PacBio sequencing technology (these reads were also used to assess the accuracy of the L1 diploid assembly, see section «Obtaining PacBio reads for L1 and assessing the accuracy of the *A. vaga* L1 genome assembly» of Supplementary Methods). We used subreads (hereafter referred to as reads) from two PacBio sequencing runs of the same library. The mean read lengths for the first and the second run were 8,146 and 8,361 bp respectively. After removing reads with similarity to spike-in control and reads with GC-content > 45% we were left with a total of 746,128 reads. These reads were mapped to the haploid sub-assembly (L1) with the aligner minimap2 (version 2.16-r922)[44] supporting alignment of long noisy PacBio reads (options "-ax map-pb"). We did not consider non-primary alignments (minimap2 option "--secondary=no") and filtered out alignments with MAPQ < 60. This resulted in the average per contig coverage with PacBio reads equal to 40.31X (median per contig coverage 43.47X).

We also computed coverage for haploid contigs no shorter than 1,000 bp, as our analyses are based on SNPs belonging to this subset of haploid contigs. The average coverage per contig from this subset was equal to 51.19X (median per contig coverage 52.16X).

Filtering of phased haplotype blocks

In the case of MiSeq-based phasing, in addition to raw phased haplotype blocks generated by HapCut2, we obtained two filtered sets of blocks applying the same filtering criteria which were applied to HiSeq-based phased data used for the main analysis. The first and the second filtered sets were subjected to the filtering procedures analogous to those applied to the HiSeq-based 'phased dataset 1' and 'phased dataset 2' respectively. In short, for both filtered sets, we excluded blocks encompassing conflicting pairs of SNPs. For the second filtered set, we further processed blocks based on the HapCut2 switch and mismatch quality scores. For details, see section «Computational phasing of genotypes» of Supplementary Methods describing filtering steps applied to the core phased data used for the analysis.

We did not subject PacBio-phased blocks to filtering, as the main filtering step applied to blocks phased using Illumina reads (exclusion of blocks with SNP pairs represented by more than two 'haplotypes' in a single individual) appears to be ill-suited for PacBio data with their high error rate (~14%).

Comparison of phasing results

First, we compared the results of phasing based on different sets of reads (HiSeq vs PacBio for L1 and HiSeq vs MiSeq for L1, L2 and L11) using subcommand "compare" of the WhatsHap[45] program (version 0.14.1). Inconsistencies between the haplotype phases recovered from different sets of reads for the same individual were regarded as switch errors.

When raw phased blocks were compared, the fraction of haploid contigs (among haploid contigs with intersecting phased blocks between the two compared phased datasets and ≥ 10 heterozygous SNP pairs assessed) exhibiting inconsistencies between haplotype phases inferred from HiSeq and MiSeq reads was 0.023 for L1, 0.037 for L2 and 0.058 for L11 (Supplementary Data 3). However, exclusion of blocks encompassing conflicting SNP pairs greatly reduced these fractions (Supplementary Data 3). The fraction of haploid contigs with putative switch errors decreased to 0.0007 for L1, 0.0030 for L2 and 0.0154 for L11.

This shows that the phased dataset 1 employed in the majority of our analyses indeed displays greater phasing accuracy than the raw phased blocks. Further filtering according to the HapCut2 switch and mismatch quality scores (applied to the phased dataset 2) pushed the fractions of haploid contigs with presumable switch errors down to 0, 0.0006 and 0.0031 for L1, L2 and L11 respectively.

Note that the estimates of the fraction of contigs with presumable switch errors are likely to reflect both errors stemming from HiSeq-based phasing and errors stemming from MiSeq-based phasing (or PacBio-based phasing, see below).

Higher discordance between HiSeq- and MiSeq-based phased blocks for L11 is probably associated with a higher genomic divergence of L11 (large cluster) from L1 and L2 (small cluster), leading to a higher rate of spurious read mapping. Our analyses are mainly based on individuals from the large cluster (L4-L11), which are probably all likely to exhibit higher levels of phasing errors than the individuals from the small cluster (L1-L3). However, although the estimated fraction of haploid contigs with discordant phasing for L11 is 0.0154 for the phased dataset 1, it becomes

significantly lower (0.0031) for the phased dataset 2. As phased dataset 2 shows a decay of LD with distance similar to the LD decay observed for phased dataset 1 (Supplementary Fig. 13b and Fig. 2a), the LD decay reported in our study does not appear to be seriously affected by phasing errors.

Comparison of L1 haplotype phases between the HiSeq- and PacBio-phased sets shows a higher rate of putative phasing errors than the comparison between the HiSeq- and MiSeq-phased sets (Supplementary Data 3). Indeed, L1 HiSeq-based phased blocks from the phased dataset 2 show a perfect consistency with the MiSeq-based blocks subjected to analogous filtering (among the 4,894 assessed haploid contigs, there are 0 contigs displaying discordance between haplotype phases for these two sets). However, 37 out of the 5,364 (0.0069) assessed haploid contigs harboring haplotype blocks from the phased dataset 2 display phase inconsistency if checked against PacBio-phased blocks. It is hard to say whether this disparity is driven by errors stemming from phasing based on short but accurate Illumina reads or errors associated with long but noisy PacBio reads. Importantly, we did not filter haplotype blocks recovered from PacBio reads, as our main filtering step involving removal of blocks covering pairs of SNPs represented by more than two 'haplotype' variants in a single individual does not appear to be feasible for PacBio data with their high error rate.

Next, to see to what extent the results of our study could be affected by phasing errors, we note that the modified four-gamete test could be applied not only to phased haplotypes from different individuals, but also to sets of haplotypes assembled for the same individual from different sets of reads. We contrasted the fractions of recombinant SNP pairs detected when comparing phased blocks from two different individuals to the fractions of SNP pairs inferred as recombinant when comparing phased blocks recovered for the same individual from different sets of reads.

For this purpose, we applied the modified four-gamete test to the results of phasing based on different sets of reads for L1, L2 and L11. Here, we proceeded in a pairwise manner by comparing two phased datasets at a time and looking for recombinant pairs of sites in these two datasets. In the case of the same individual, pairs of 'recombinant' sites would correspond to discordances between haplotype phases recovered for the same individual from different sets of reads.

First, we applied the modified four-gamete test to haplotypes reconstructed for the same individual (L1, L2 or L11) from different sets of reads. Here, all SNP pairs passing the modified four-gamete test are likely to correspond to phasing errors, as only a single individual is considered and we do not expect recombination events. Nevertheless, we observed that sets of haplotypes subjected only to basic filtering (exclusion of blocks encompassing conflicting SNP pairs, such filtering was applied to the main phased dataset 1) displayed an increase in the fraction of 'recombinant' SNP pairs with distance (Supplementary Fig. 14). The observed increase in the rate of discordant phasing with distance is not surprising, as haplotype assembly is expected to be less accurate at larger distances. However, the overall fraction of SNP pairs with discordant phasing recovered from different sets of reads was very low for all assessed distances (in most cases, of the order of 10^{-4}–10^{-3}; Supplementary Fig. 14). Sets of haplotypes subjected to further filtering according to the switch and mismatch quality scores (such filtering was applied to the phased dataset 2) exhibited even lower fractions of SNP pairs discordantly phased in different datasets obtained for the same individual (Supplementary Fig. 14). Interestingly, for the phased sets subjected to the switch and mismatch quality filtering (corresponding to the filtering applied to

the phased dataset 2), there was no obvious general trend of increase in the fraction of inconsistently phased SNP pairs (‘recombinant’ SNP pairs) with distance (Supplementary Fig. 14).

Importantly, the trend of LD decay detected when comparing haplotypes from different individuals was similar when assessed based on haplotypes from the main phased dataset 1 (from which blocks with conflicting SNP pairs were removed, Fig. 2a) and from the phased dataset 2 (additionally subjected to the switch and mismatch quality filtering, Supplementary Fig. 13b). As the rate of phasing discordances for the ‘phased dataset 2’ appears to be very low and no clear trend of increase in the rate of phasing errors with distance is observed, it does not seem plausible that the LD decay reported for *A. vaga* in the current study could be explained by phasing errors.

Nevertheless, we sought to further estimate whether the LD decay is likely to be significantly affected by phasing artifacts. For this, we applied the modified four-gamete test to two pairs of individuals for which more than one phased dataset was available (L2-L1 and L11-L1) and compared its results to the results of the four-gamete test applied to different phased datasets obtained for the same individual. When analyzing two different individuals, we expect to detect recombinant pairs of SNPs stemming not only from phasing errors but also from true recombination events (if any). Indeed, as expected from true recombination, the fraction of recombinant SNP pairs inferred from comparison of different individuals is two orders of magnitude or more higher than that of the same individual using different data (of the order of 10^{-3} or less; Supplementary Figs. 17 and 18).

For example, in the comparison of L2 haplotypes assembled from HiSeq and MiSeq reads, the fraction of apparently ‘recombinant’ SNP pairs among all pairs of heterozygous sites within 1 to 230 bp is 5×10^{-5}, while the fraction of recombinant SNP pairs at the same distance inferred from comparison of L1 and L2 haplotypes from HiSeq reads is 8.7×10^{-3}. The fractions of recombinant SNP pairs observed when applying the modified four-gamete test to the pair of individuals L11-L1 were as high as 0.39 at distances >1,940 bp apart, while the corresponding fraction for comparison of L11 haplotypes recovered from HiSeq and MiSeq reads was only 4.7×10^{-4}. For some distance bins, the fractions of recombinant SNP pairs inferred from comparisons of different individuals are up to four orders of magnitude higher than the corresponding fractions in comparisons of different phased datasets for the same individual (Supplementary Figs. 17 and 18).

The rate of increase in the fraction of recombinant SNP pairs with distance observed when comparing haplotypes from two different individuals was similar irrespective of which set of reads was used to assemble haplotypes and of the stringency of filtering of phased blocks (Supplementary Figs. 17 and 18). This shows that the signal of LD decay assessed through the modified four-gamete test persists irrespective of whether haplotypes are phased using HiSeq, MiSeq or PacBio reads and regardless of the filtering stringency of the phased data.

Conceivably, some genomic regions, e.g. repeats or low-complexity regions, can be more prone to erroneous phasing independently of the sequencing technology and filtering, and this could result in recurrent erroneous phasing of the same block from different sets of reads. In such cases, comparison of phased blocks assembled for the same individual from different reads would not reveal phasing inconsistencies, as an erroneously assembled haplotype would be present in both datasets. Therefore, it is likely that our approach does not detect some phasing errors. Still, phasing errors are more likely to be shared by the datasets assembled from HiSeq and MiSeq Illumina

reads than by Illumina- and PacBio-phased datasets. Indeed, testing of L1 haplotype phases recovered from HiSeq reads against those from PacBio reads reveals a higher rate of putative phasing errors than in a comparison of HiSeq-based phased sets against MiSeq-phased sets (Supplementary Data 3). However, although the estimates of phasing error rate for L1 retrieved through comparison of filtered HiSeq-phased datasets against the PacBio-phased dataset are larger than those obtained from comparison of L1 HiSeq- and MiSeq-phased datasets subjected to analogous filtering, estimates of the fraction of haploid contigs (among those harboring phased haplotype blocks) with putative switch errors as assessed for these filtered L1 datasets from PacBio reads remain below 1% (Supplementary Data 3). Moreover, the results of the modified four-gamete test obtained using PacBio-based phased blocks for L1 are almost indistinguishable from those obtained using HiSeq- or MiSeq-phased blocks (Supplementary Figs. 17 and 18).

Taken together, these analyses show that the observed decay of LD in *A. vaga* could not be attributed to phasing errors.

Supplementary Note 3: Distinguishing signatures of gene conversion and other types of recombination

To disentangle signatures of gene conversion from those of other types of recombination (further referred to in this Note as recombination), we devised a modified implementation of the Hudson's four-gamete test[46]. In the original Hudson's four-gamete test, the presence of all four possible haplotypes for a pair of biallelic polymorphic loci within a population is interpreted as evidence for recombination, because recurrent mutations are unlikely. However, a mutation followed by gene conversion would suffice to explain the presence of all four haplotypes without assuming genetic exchanges between individuals (Fig. 3a). Nevertheless, allelic gene conversion can only produce a homozygous genotype from the heterozygous one, but not *vice versa*. Therefore, it cannot produce a pair of individuals, each heterozygous at two loci, carrying all four haplotypes (Fig. 3b); while such a pair can obviously arise through homologous recombination during conventional meiosis or transformation. We use this feature of gene conversion to distinguish it from other types of recombination.

For this purpose, for each pair of the sequenced *A. vaga* individuals, we consider only those pairs of sites at which both individuals are simultaneously heterozygous. Next, among all such pairs of heterozygous sites for a given pair of individuals, we look for those that are represented by all four possible haplotypes in these two individuals (Supplementary Fig. 16). In the absence of recurrent mutations, presence of such pairs of sites is indicative of recombination. Note that reciprocal mitotic recombination can also in principle give rise to such pairs of sites. We refer to such pairs of sites in the text of the paper as to ‘recombinant’ pairs of sites, or pairs of sites passing the modified four-gamete test.

To obtain a statistic that could be applied to all individuals simultaneously, we compute the fraction of SNP pairs passing the modified four-gamete test among all SNP pairs that are simultaneously heterozygous in at least one pair of the considered individuals.

To see if the fraction of recombinant SNP pairs increases with distance, heterozygous SNP pairs meeting the requirements of the modified four-gamete test were subdivided into 4 distance bins with approximately equal numbers of cases using the *cut_number* function from the ggplot2 package (version 3.2.1) in R.

Fractions of recombinant SNP pairs were calculated for each bin, and significance of the difference in these fractions for all pairs of bins was assessed by permuting SNP pairs between the two compared bins 10,000 times (i.e. randomly reassigning pairs of SNPs to one or the other bin; Fig. 3c). For each pair of bins, the two-sided *P* value was computed based on 10,000 permutations and adjusted for multiple testing using the Bonferroni method. Comparisons between all pairs of bins were found to be significant (in all cases $P < 6\times10^{-4}$). To complement this analysis, we compared distributions of distances between SNPs in recombinant and non-recombinant pairs (among the SNP pairs meeting the conditions of the modified four-gamete test) showing that recombinant pairs of SNPs tend to reside farther apart from each other than non-recombinant ones (Fig. 3d; two-sided $P < 1\times10^{-4}$, permutation test).

The observed increase in the fraction of recombinant SNP pairs with increasing physical distance (Fig. 3c, d) is equivalent to LD decay that could not be ascribed solely to the action of gene conversion. Note that recurrent mutations can give rise to pairs of recombinant sites passing the modified four-gamete test, however the fraction of such pairs resulting from recurrent mutations is not expected to increase with physical distance. The results reported in the paper are for pairwise comparisons among the individuals L4-L11 with the cut-off threshold for a minor allele count of 4.

Supplementary Note 4: Characterizing relationship between recombination and GC-content in *A. vaga*

We sought to investigate whether the probability of a recombination event is associated with the GC-content of a genomic region.

As a proxy for recombination rate of a region we used two measures:

1. Normalized minimum number of recombination events (R_{min})[46] inferred for individual phased segments with LDhat (version 2.2)[33]. Estimation of the minimum number of recombination events was performed for the same set of phased genomic segments (n = 245) which was utilized to obtain Wakeley's estimates of the population-scaled recombination rate (see section «Estimation of the population-scaled recombination rate» of Supplementary Note 10). Estimates of R_{min} according to Hudson and Kaplan[46] for individual phased segments were inferred with LDhat[33]. We normalized the obtained values dividing them by the total number of SNP pairs residing within the phased segment.
2. Fraction of SNP pairs passing the modified four-gamete test. Unlike R_{min}, which was calculated for individual phased genomic segments, this analysis was done in a contig-wise manner. For this purpose, we first retained only those contigs from the haploid sub-assembly that harbor at least 20 pairs of heterozygous SNPs (minor allele count ≥ 4) meeting conditions of the modified four-gamete test, which resulted in a set of 666 haploid contigs. For each haploid contig from the resulting set, we calculated the fraction of SNP pairs passing the modified four-gamete test.

We observed a weak negative correlation between the normalized $R_{\min}$ and the GC-content of a genomic segment (Supplementary Fig. 19a): the corresponding Pearson's $r = -0.28$ (95% confidence interval [CI]: −0.39 to −0.16), P value of the two-sided t-test = 1.072×10^{-5}. By performing a linear regression of normalized $R_{\min}$ on the segment GC-content, we obtained the slope estimate of −0.052 (standard error [SE] of the slope = 0.012). R-squared of this model = 0.077; $F = 20.22$ with 1 and 243 degrees of freedom, P value = 1.072×10^{-5}. To rule out the size of the GC-poor segments as a confounder for the negative correlation, we fitted a multiple linear regression model with GC-content and the size of the segment as explanatory variables and the normalized $R_{\min}$ as the response variable. The P value for the partial regression coefficient associated with GC-content remained significant (partial regression coefficient = −0.052 with SE = 0.011; t-value = −4.61, P value = 6.52×10^{-6}).

In line with this observation, fractions of SNP pairs passing the modified four-gamete test confirmed the same pattern demonstrating a negative correlation with the GC-content of a haploid contig (Supplementary Fig. 19b): Pearson's $r = -0.16$ (95% CI: −0.23 to −0.08), P value of the two-sided t-test = 4.221×10^{-5}. We fitted a linear regression model of the fraction of recombinant SNP pairs on the haploid contig GC-content and obtained the slope estimate = −1.78 (SE = 0.43). R-squared of this model = 0.025; $F = 17$ with 1 and 664 degrees of freedom, P value = 4.221×10^{-5}.

To confirm that the observed negative relationship is not due to larger sizes of GC-poor contigs, we uncoupled the effects of GC-content and contig size by comparing fractions of SNP pairs passing the modified four-gamete test among the SNP pairs falling within the same distance bin for haploid contigs of different GC-content (Supplementary Fig. 19c). For this purpose, we subdivided haploid contigs carrying heterozygous pairs of SNPs meeting the conditions of the modified four-gamete test (n = 1,740) into 3 bins of approximately equal size according to their GC-content. Next, each considered pair of heterozygous SNPs was assigned to a group based on its distance bin and GC-content bin of the corresponding haploid contig. This analysis showed that for the same distance bin, recombinant SNP pairs tend to be found in GC-depleted contigs (Supplementary Fig. 19c).

To see if individual recombination events tend to happen in regions with skewed GC-content, we focused on genomic intervals likely overlapping sites of recombination events. Even if the contig identity was controlled for, recombination events showed tendency to occur in the GC-depleted regions of the contig. This has been demonstrated in the following way. For each contig of the haploid sub-assembly carrying pairs of SNPs passing the modified four-gamete test (MAC ≥ 4 among L4-L11), we selected a pair of SNPs separated by the smallest distance among those recombinant SNP pairs that were located at a distance of at least 100 bp from each other (if no such pairs were found, the contig was excluded). We treated intervals separating such sites (n = 1,014) as a proxy for the locations of recombination events (further referred to as 'recombinant intervals'). To see if the GC-content of such intervals is lower than would be expected by chance, we randomly sampled genomic intervals preserving the number, haploid contig identity and the distribution of sizes of the actual recombinant intervals. This sampling procedure was repeated 1,000 times and the mean GC-content of the recombinant intervals was compared to the mean values of GC-content for 1,000 random samples.

The reduction in GC-content of the recombinant intervals relative to random expectation was found to be significant (P = 0.02). Here, the one-sided P value was computed as the fraction of 1,000 random interval sets with mean GC-content

(rounded to three decimal places) lower or equal to the mean GC-content of the recombinant intervals (Supplementary Fig. 19d).

Therefore, it appears that recombination is more likely to occur in GC-poor regions of the *A. vag*a genome. While a similar pattern has been described in some organisms[47] (e.g. in *Arabidopsis thaliana*), the opposite trend attributed to GC-biased gene conversion is more common[48]. This has interesting implications for future studies of recombination and gene conversion in *A. vaga* and in bdelloid rotifers in general. A question of whether gene conversion in bdelloids is not GC-biased (as it appears to be in *Drosophila melanogaster*[49]) is of special interest.

Supplementary Note 5: Gene conversion and deviations from Hardy-Weinberg equilibrium

Here, we ask whether gene conversion combined with mutation can lead to Hardy-Weinberg equilibrium (HWE) in the absence of sex.

Consider two values: the inbreeding coefficient $F,$ defined as the probability that two alleles sampled from the same individual are identical by descent, and $\theta,$ defined as the probability that two alleles sampled from different individuals are identical by descent.

General equations describing the dynamics of identities by descent under arbitrary probability of clonal reproduction, selfing and migration in a subdivided population (in the absence of gene conversion) are given by Balloux *et al.* 2003 (ref. [50]). In this Note and in the legends of related figures, to keep variable names consistent with those used by Balloux *et al.*, the mutation rate is denoted by u (instead of μ as in the main text and the rest of supplementary information).

Here, we consider an idealized Wright-Fischer population[51], therefore, the effective population size is equal to the census population size ($N_{\mathrm{e}} = N$).

Let F_t and θ_t denote the probabilities of two alleles being identical by descent at generation t. Assume that prior to reproduction, a heterozygote turns into a homozygote with probability α due to a gene conversion event. Then under strict clonality and assuming a single unstructured population,

$$F_{t+1} = (1-u)^2 F_t + \alpha(1 - F_t)$$

$$\theta_{t+1} = (1-u)^2 \left(\frac{1}{N}\left(\frac{1+F_t}{2}\right) + \left(1 - \frac{1}{N}\right)\theta_t\right). \qquad (1)$$

In the absence of gene conversion ($\alpha = 0$), these equations are an instance of eq. (5) in (Balloux *et al.* 2003) under strict clonality[50]. The only effect of conversion is increasing F, and it can only act on a former heterozygote (which has frequency $1 - F$).

At equilibrium,

$$F = \frac{\alpha}{2u - u^2 + \alpha}$$

$$\theta = \frac{(1-u)^2(1+F)}{2Nu(2-u) + 2(1-u)^2}. \qquad (2)$$

Without mutation ($u = 0$), $F = 1$ and $\theta = 1$; this is because conversion rids the population of any differences within individuals, while genetic drift rids the population of any differences between individuals.

In the absence of conversion ($\alpha = 0$), the differences between alleles within an individual will accumulate indefinitely, unchecked by any forces; while the differences between alleles in different individuals will be in mutation-drift balance:

$F = 0$

$$\theta = \frac{(1-u)^2}{2Nu(2-u) + 2(1-u)^2}; \quad (3)$$

if the mutation rate is small ($u^2 \approx 0$), the equation for θ reduces to:

$$\theta = \frac{1}{2 + 4Nu}. \quad (4)$$

If $4Nu$ is large, θ approaches 0, while if it is small, θ approaches 0.5.

The extent of the deviation from HWE can be described by the F_{IS} statistic,

$$F_{\mathrm{IS}} = \frac{F - \theta}{1 - \theta}, \quad (5)$$

which equals 0 under the HWE, is positive if there is an excess of homozygotes, and negative if there is an excess of heterozygotes. Plugging the values of (2) into (5) allows to calculate the equilibrium values of the F_{IS} statistic.

F_{IS} statistic is also commonly computed as $F_{\mathrm{IS}} = 1 - \frac{H_{\mathrm{o}}}{H_{\mathrm{e}}}$, where H_{o} and H_{e} stand for the observed and expected heterozygosity respectively. Note that these two definitions give equivalent results[52]:

$$F_{\mathrm{IS}} = 1 - \frac{H_{\mathrm{o}}}{H_{\mathrm{e}}} \cong \frac{F - \theta}{1 - \theta}. \quad (6)$$

In the absence of conversion ($\alpha = 0$), clonal reproduction leads to indefinite accumulation of mutations between the two haploid genotypes within a single individual, leading to a strong excess of heterozygotes. If drift is strong *(*$4Nu$ is very small), this will lead to $F_{\mathrm{IS}} = -1$, although under higher $4Nu$*,* the excess of heterozygotes will not be so radical and the values of F_{IS} will be above -1 (Balloux *et al.* 2003, ref. [50]).

Conversion can restore homozygotes, increasing F_{IS}, and under some parameter values can make the equilibrium within-individual differences equal to those between individuals ($F = \theta$, $F_{\mathrm{IS}} = 0$). However, the conditions for that are extremely restrictive. From equation (2), the value of α corresponding to this equilibrium is:

$$\alpha = \frac{(1-u)^2}{2N}. \quad (7)$$

For realistic mutation rates, this is very close to:

$$\alpha = \frac{1}{2N}. \qquad (8)$$

In other words, for conversion to recreate the HWE, it needs to reduce F at the same rate as drift reduces θ, which is $1/2N$.

Even slight deviations of α from this equilibrium value will radically deviate the population from the HWE. To illustrate this, we plot F_{IS} as the function of conversion rate α for three pairs of parameters: $N = 10^4$, $u = 10^{-7}$ (Supplementary Fig. 21); $N = 10^5$, $u = 10^{-8}$ (Supplementary Fig. 22); and $N = 10^6$, $u = 10^{-9}$ (Supplementary Fig. 23). In all three cases, $4Nu = 0.004$, which is close to the value observed in the L4-L11 cluster. However, the values of α leading to $F_{\mathrm{IS}} \approx 0$ differ by an order of magnitude between the three cases (respectively $\alpha = 5\times10^{-5}$, 5×10^{-6} and 5×10^{-7}). A small deviation of α from the required value in either direction leads to a substantial deviation from the HWE; for example, under the parameters of Supplementary Fig. 21, $F_{\mathrm{IS}} = -0.11$ if $\alpha = 4\times10^{-5}$, and $F_{\mathrm{IS}} = 0.09$ if $\alpha = 6\times10^{-5}$. It is unclear why mutation, effective population size and conversion rate should conspire to give a good match to the HWE.

Supplementary Note 6: Analysis of triallelic sites

Identification of sites harboring three heterozygous genotypes

To estimate the observed to expected ratio of the numbers of triallelic sites carrying all three heterozygous genotypes, we used only those sites of the *A. vaga* genome (belonging to the stringent SNP dataset II) that were simultaneously called in all the individuals (L1-L11) and applied additional strict filters on the SNP quality. Prior to the analysis, we excluded all sites for which there were more than two nucleotides simultaneously present in the aligned reads in any individual genome. We subdivided the resulting set of sites according to the number of alleles they carried within the large cluster (L4-L11).

The probability of a mutation recurrently affecting the same site could be estimated from the fraction of triallelic sites among all sites with two or three alleles. Hence, we calculated the fraction of triallelic sites (P3) among all sites represented by two or three alleles. This fraction could be viewed as an estimate of a probability of a mutation recurrently affecting the same site in the history of the sample of genotypes. Therefore, the expected number of triallelic sites simultaneously harboring all three possible heterozygous genotypes due to recurrent or back mutations could be estimated as N3 × P3, where N3 is the observed number of the triallelic sites. The significance of the difference between the observed and expected fractions of triallelic sites carrying all three heterozygous genotypes was assessed with one-sample *Z*-test for proportions (function *prop.test* from the stats R package [version 3.6.3] employed without continuity correction, two-sided test).

Among high-quality 1,136,041 sites variable among the individuals L4-L11, 9,738 sites (0.008572) were found to be triallelic, thus we would expect 83.5 (9,738 × 0.008572) triallelic sites to carry all the three possible heterozygous combinations of alleles due to recurrent mutations. However, the observed number of such sites is 1,839 (0.189 among all triallelic sites; Supplementary Fig. 24). To explain this observation under the hypothesis of obligate asexuality, one would have

to allow the rate of recurrent mutations to be ~22 times higher than it apparently is ($P < 2.2\times10^{-16}$, one-sample Z-test for proportions; Supplementary Data 4).

Besides, recurrent mutations affecting triallelic sites should give rise to the similar numbers of tetraallelic sites and sites carrying all three heterozygous genotypes. Therefore, the observed number of tetraallelic sites could be used to obtain an independent estimate of the expected number of sites represented by three heterozygous genotypes due to recurrent mutations. Among the whole-genome calls for L4-L11, only 1 high-quality tetraallelic site was identified (versus 1,839 triallelic sites with three heterozygotes; Supplementary Data 4), this argues against recurrent mutations as the main source of sites harboring three heterozygous genotypes.

Importantly, if only the regions of the genome with high-confidence ploidy (allelic regions or allelic genes) were considered, even a greater enrichment with triallelic sites carrying three heterozygous genotypes was observed (Supplementary Data 4), making erroneous read mappings an unlikely explanation for the phenomenon.

To check that our estimates of the proportion of triallelic sites among sites with two or three alleles and consequently the estimates of the expected numbers of triallelic sites with three heterozygotes were not significantly biased due to applied filtering, we repeated the analysis on less stringently filtered sets of sites. Specifically, we repeated the analysis (i) using sites from the stringent SNP dataset II without removing sites for which there were more than two nucleotides simultaneously present in the aligned reads from individual genomes, (ii) using sites from the SNP dataset III (see section «Variant calling and filtering» of Supplementary Methods). Using these two datasets produced virtually the same results (Supplementary Data 4).

Analysis of sites harboring three heterozygous genotypes

It is conceivable that sites with all three possible heterozygous genotypes could be a result of cross-sample contamination. However, if it were the case, we would expect those samples that originated from contamination to harbor the majority of rare heterozygous genotypes. To confirm that the sites carrying all three possible heterozygous genotypes are not likely to be due to cross-sample contamination, we separately considered those sites carrying all three heterozygotes among the individuals L4-L11 that harbor only one private heterozygous genotype (n = 607). That is, we retained a site for the analysis if the least frequent of the three heterozygous genotypes was present in a single individual with the next frequent genotype present at least in two individuals.

Such private heterozygous genotypes possessed by a single individual are most likely to stem from contamination. Moreover, should contamination be the case, we would expect to see a skewed distribution of per individual numbers of such private heterozygous sites with the samples resulting from contamination carrying disproportionally more private heterozygotes.

Following this logic, we analyzed how the 607 private heterozygous genotypes are distributed among different individuals. For this purpose, for each individual, we tabulated the total number of sites with the least frequent heterozygous genotype private to this individual.

Contrary to what would be expected under contamination, we observed that the resulting numbers of private heterozygous sites were similar across different individuals (average number of private heterozygous sites per individual was 75.9, with the minimum and maximum values of 64 and 88 sites respectively; Supplementary Table 12). Thus, the distribution of unique heterozygous genotypes

among the sequenced individuals argues against contamination being the source of sites harboring all three heterozygotes.

Supplementary Note 7: Analysis of mitochondrial variation in L1-L11

COX1 phylogenies did not support the same subdivision of individuals L1-L11 into two genetic clusters (L1-L3 and L4-L11) that was inferred from their nuclear genomes. Notably, in the *COX1* tree, individuals L2-L11 were intermingled, while L1 had a longer branch and even grouped with isolates belonging to other *A. vaga* cryptic species rather than with L2-L11 (Supplementary Note 1 and Supplementary Figs. 2-3). To see whether this grouping may be due to technical artifacts, we undertook further analysis. As we sequenced cultures established from distinct individuals rather than individual rotifers, in principle, it is possible that a divergent mitochondrial haplotype of L1 could stem from contamination. However, in this case, we would expect to detect more than one frequent mitochondrial haplotype in Illumina reads derived from L1 culture. This logic is also applicable to testing for contamination in other sequenced cultures: although some mitochondrial variation can be expected even in a culture derived from a single individual, all copies of the mitochondrial genome present in it should be very similar to each other.

To look for signatures of potential contaminations, we first extracted sequences of mitochondrial contigs from the L1 diploid assembly and from highly fragmented assemblies obtained for the rest of individuals L2-L11 from the available Illumina HiSeq reads (these assemblies were also used to extract *COX1* sequences, see Supplementary Note 1). For this, we used a simple approach similar to that used to extract *COX1*-containing regions: namely, we used the sequence of the mitochondrial contig from the first published *A. vaga* genome[1] as the query for blastn search against individual genomes of L1-L11.

To identify the mitochondrial contig from the first published *A. vaga* genome assembly[1], we performed a blastn search against this assembly using the two sequences of reference bdelloid mitochondrial genomes from other species available in GenBank as queries: *Philodina citrina* (GenBank accession number FR856884.1; 14,003 bp in length) and *Rotaria rotatoria* (GenBank accession number GQ304898.1; 15,319 bp in length). The best hit for both of these queries was to contig 2917 of the 2013 *A. vaga* genome assembly (CAWI020038741.1). This contig covers a large fraction of the reference mitochondrial genomes: 81% for *Philodina* (80.07% identity) and 74% for *Rotaria* (79.49% identity). Reciprocally, the two best hits of contig 2917 in nt database were the mitochondrial genomes of *Philodina* and *Rotaria*. The third best scoring hit was the *COX1* gene of *A. vaga* (JX184001.1)[39]; the corresponding hit covered only 6% of the contig, but had 100% sequence identity. We further used contig 2917 as the reference sequence of the *A. vaga* mitochondrial genome.

Analysis of blastn hits of this reference *A. vaga* mitochondrial contig in L1-L11 genomes revealed that the bulk of the mitochondrial genome was usually present in a small number of contigs (ranging from 1 to 3). Specifically, in the L1 diploid assembly, the bulk of the mitochondrial genome was split between three contigs: contig8072 (length = 7,124 bp), contig11064 (length = 3,631 bp) and contig12085 (length = 2,836 bp) corresponding to three fragments of the reference *A. vaga* contig. L1 contig12085 contained a large number of short tandem repeats (with period size ranging from 13 to 36 bp as identified with the Tandem repeats finder[53]) and was not used in the majority of downstream analyses; however,

inclusion of this contig did not change the results. In assemblies obtained for L2, L3, L4, L7, L10 and L11, most of the mitochondrial genome assembled into a single contig of about 14,000 bp in length (ranging from 13,781 bp for L3 to 14,052 bp for L4). Assemblies for L5 and L9 contained a large part of the mitochondrial genome in two contigs, and L6 and L8 in three contigs (with the overall length ranging from 9,360 bp for L6 to 13,967 bp for L5). Extracted mitochondrial contigs[54] for individuals L1-L11 in the FASTA format are available at https://doi.org/10.6084/m9.figshare.12008790.v2.

In line with the analysis of *COX1* phylogenies, mitochondrial haplotypes of individuals L2-L11 were all very similar: the average identity of the best blastn hit of the L4 mitochondrial contig against mitochondrial contigs of individuals L2-L3 and L5-L11 was 99.66% (ranging from 99.33% for L4 versus L11 to 99.88% for L4 versus L2). Also similarly to *COX1*, the L1 mitochondrial haplotype was significantly more divergent from the L2-L11 haplotypes than the L2-L11 haplotypes from one another. The average identity of the best blastn hit of L1 contig8072 against mitochondrial genomes of individuals L2-L11 was only 91.41% (ranging from 90.43% to 92.75% for blastn search against mitochondrial contigs of different individuals). The corresponding value for L1 contig11064 was 91.07% (ranging from 90.97% to 91.18%). The nucleotide identity between contig12085 and mitochondrial genomes of L2-L11 was even lower (average identity of the best blastn hit in L2-L11 mitochondrial contigs for contig12085 was 84.27%, ranging from 83.14% to 84.58%) reflecting, in part, a substantial number of indels, likely associated with the presence of tandem repeats in the corresponding region of the mitochondrial genome.

Blastn searches with L1 mitochondrial contigs as queries against complete assemblies of L2-L11 did not reveal 'alternative' mitochondrial haplotypes more closely related to the L1 haplotype. Similarly, blastn searches with L2-L11 mitochondrial contigs as queries against the L1 assembly also did not reveal the presence of another variant of mitochondrial haplotype that would be more similar to L2-L11 haplotypes among the L1 contigs. This confirms that the placement of L1 in the *COX1* tree is indeed due to a divergent mitochondrial haplotype of L1 and not due to contamination of the L1 culture.

As discussed in the main text, patterns of nuclear haplotype phylogenies observed in L1-L11 point to hybrid origin of L1-L3, with L1-L3 possibly representing an offspring of a cross from a population close to that of the large cluster and a relatively distant population (see the main text for details). Given that L1 and L2-L3 carry diverged mitochondrial haplotypes, a single event of hybridization would not suffice to produce these three individuals. However, the data could be explained by assuming at least two reciprocal hybridization events: one resulting in retention of the mitochondrial genome from the population of the large cluster (L2 and L3) and one leading to retention of the mitochondrial genome from the second unknown population (L1). This hypothesis is impossible to test directly in the absence of data on mitochondrial haplotypes of individuals from this second population.

Still, the data on the extent of within- and between-individual divergence indirectly support reciprocal hybridization events. If we assume that L1-L3 are indeed hybrids, then the divergence level between the two haplotypes of L1-L3 would reflect the genetic distance between the nuclear genomes of the two populations involved in the hybridization event. These divergence levels estimated as proportions of heterozygous sites within individual genomes of L1-L3 are ~2% (Supplementary Data 2). Meanwhile, if L2-L3 carry the mitochondrial haplotypes inherited from the 'population of the large cluster' and L1 carries a mitochondrial haplotype inherited

from the second unknown population involved in hybridization, then the extent of mitochondrial divergence between these two populations is ~9% (as estimated from the best blastn hits of L1 mitochondrial contigs contig8072 and contig11064 against L2-L11, see above). These estimates of nuclear and mitochondrial divergences can only be compared cautiously because while nuclear estimates are based on genotype calls made against the reference genome, mitochondrial estimates employ *de novo* assembled contigs. Still, it is clear that the mitochondrial haplotype of L1 is more divergent from mitochondrial haplotypes of L2-L11 than the two nuclear haplotypes of L1-L3 from each other. This is consistent with what would be expected as a result of reciprocal hybridization events. It is well known that the mutation and divergence rates in mitochondria for most species are at least several times higher than those in the nucleus[55]. Therefore, populations with a ~2% difference between nuclear genomes are expected to exhibit a much higher mitochondrial difference, consistent with our findings. This further supports the reciprocal hybridization scenario.

To gain more insight into the relationships between mitochondrial haplotypes of the sequenced individuals, we used mitochondrial genotype calls obtained for 10 individuals L2-L11 carrying similar mitochondrial haplotypes (see Supplementary Note 8) to build a phylogenetic tree. For this, for each individual L2-L11, we reconstructed its mitochondrial haplotype based on the genotype calls produced against the L4 mitochondrial contig as reference (total length 14,052 bp; see Supplementary Note 8). Prior to being used for reconstructing haplotypes, genotype calls were subjected to stringent filtration (this filtering procedure is also described in Supplementary Note 8): we filtered out sites with heterozygous calls in any of the individuals L2-L11 (3 sites), SNPs within 10 bp of an indel, indels, sites with missing genotypes or coverage DP < 50 in any of the individuals L2-L11 and sites with low-quality calls (QUAL < 15). This stringent filtering resulted in 13,765 high-confidence sites retaining 98% of the total mitochondrial reference contig. We then used the "consensus" command of BCFtools to obtain haplotype sequences for each of the individuals L2-L11. The resulting sequences were used to infer the mitochondrial phylogeny of L2-L11 in RAxML (version 8.2.12)[42]. This phylogenetic inference based on the almost complete mitochondrial sequences was performed under the GTR+G model with 1,000 bootstrap replicates. The resulting tree was visualized in Dendroscope (version 3.5.10)[43] and manually rooted at the longest branch (Supplementary Fig. 27).

Additionally, we also inferred mitochondrial phylogeny for all 11 individuals, L1-L11. The L1 mitochondrial haplotype is too divergent from mitochondrial haplotypes of L2-L11 to allow simultaneous genotype calling of mitochondrial variants for L1 and L2-L11 (see Supplementary Note 8). Therefore, to obtain an alignment of a reasonably long mitochondrial fragment for all 11 individuals, we aligned the sequence of the longest L1 mitochondrial contig (contig8072, length = 7,124 bp; see above) with the mitochondrial haplotypes of L2-L11 obtained in the previous step using MUSCLE (version 3.8.31)[41]. To infer the L1-L11 mitochondrial phylogeny, we used a segment of the alignment corresponding to the region present in the L1 contig8072 (total alignment length = 7,126 bp). Flanking alignment segments not covered by L1 contig8072 were trimmed prior to phylogenetic reconstruction. The mitochondrial phylogenetic tree for L1-L11 was constructed analogously to the mitochondrial tree for L2-L11 (see above). Due to the presence of a long L1 branch in the resulting tree, to highlight topology, we show this tree with branches not to scale (Supplementary Fig. 28). We also show the part of this tree remaining after removing the L1 branch (Supplementary Fig. 29). This allows to

draw the L2-L11 branches to scale, while preserving the order of the branches as in the complete L1-L11 tree rooted using the L1 mitochondrial haplotype as an outgroup.

Interestingly, in the inferred mitochondrial phylogenies, two individuals from the small cluster (L2 and L3) do not form a monophyletic group: L2 is most closely related not to L3 but to L10 (Supplementary Figs. 27-29). This suggests that L2 and L3 may have originated from two separate hybridization events, making the total number of hybridization events required to produce the small cluster equal to three.

Supplementary Note 8: Looking for patterns in mitochondrial variation suggestive of cross-culture contamination

We asked whether the data on between- and within-individual mitochondrial variation is suggestive of cross-sample contamination. For this, we identified the mitochondrial single-nucleotide variants carried by the sequenced *A. vaga* individuals. As a reference sequence for this analysis, we selected the mitochondrial contig of L4 (assigned to the large cluster based on nuclear variants; Fig. 1c and Supplementary Fig. 8), as L4 harbored the longest mitochondrial contig (14,052 bp) among all sequenced individuals (see Supplementary Note 7). In addition, we repeated this analysis using a mitochondrial contig from an individual assigned to another genetic cluster based on nuclear variants (L3, small cluster) as reference.

We aligned the Illumina HiSeq reads for each sequenced individual to the L4 (L3) mitochondrial contig with Bowtie 2 (version 2.3.2) with the parameters "--no-mixed --no-discordant" specifying the maximum insert size of 800 bp with only a single best alignment of the pair of reads reported. Filtering of read alignments to the mitochondrial contigs was carried out similarly to that employed in the analysis of nuclear variants: we removed reads for which more than one alignment was found (those with XS tag set) and, among the remaining reads, retained only properly-paired reads with MAPQ ≥ 20. The resulting alignments[56] were used to call mitochondrial single-nucleotide variants. These filtered alignments in the BAM format are available at http://doi.org/10.6084/m9.figshare.11396955.v2.

As previously, genotype calls were generated using the SAMtools[13] mpileup utility (v.1.4.1) with the parameters "-aa -u -t DP,AD,ADF,ADR" followed by the command "bcftools call" with the "-m" option. To detect mitochondrial sites possibly affected by contamination and heteroplasmic sites, we performed genotype calling in the default diploid mode. If only a single mitochondrial haplotype is present in a clonal culture, all genotypes for this culture would be expected to be called as 'homozygous'. Conversely, substantial contamination with a different mitochondrial haplotype would be expected to produce 'heterozygous' calls. 'Heterozygous' mitochondrial calls can also arise from mitochondrial heteroplasmy (presence of different variants of mitochondrial genome within the cell)[57,58] or differences accumulated between individuals of a clonal culture.

First, we performed joint mitochondrial genotype calling for all 11 individuals, L1-L11, together. However, unlike the nuclear genome, where a lower level of inter-individual divergence (with an average of 1.22% between the large and the small cluster) allowed aligning reads and simultaneous variant calling in all 11 individuals against L1 contigs, the mitochondrial haplotype of L1 turned out to be too divergent from L2-L11 to allow simultaneous mitochondrial variant calling in 11 individuals using standard approaches. The L1 Illumina reads aligned to L4 (L3) mitochondrial contigs very unevenly, probably due to differences in conservation

level, with well-covered regions alternating with regions exhibiting zero or near-zero coverage. As a result, out of the 14,033 assessed sites of the L4 mitochondrial contig, 7,484 were covered by fewer than 5 reads in L1, and at 5,526 sites, the genotype of L1 was not determined. To avoid dealing with a large number of missing genotypes, we excluded L1 from simultaneous variant calling with L2-L11. Instead, we generated genotype calls for 10 individuals (L2-L11) harboring similar mitochondrial haplotypes. After genotype filtering (SNPs within 10 bp of an indel removed, indels removed, sites with missing genotypes or DP < 50 in any of the individuals L2-L11 removed, sites with QUAL < 15 removed), we were left with 13,768 sites available for simultaneous analysis in L2-L11. If the L3 mitochondrial contig was used as reference, the corresponding number of available sites was 13,644. That is, a substantial portion of the mitochondrial genome could be assessed in all of the individuals L2-L11 relative to both 'reference' mitochondrial contigs.

First, for each individual L2-L11, we tabulated the numbers of mitochondrial sites called with SAMtools/BCFtools as homozygous or heterozygous. Nearly all genotyped sites (13,765 out of 13,768) were called as homozygous in all 10 individuals. This is consistent with the expectation of a single frequent mitochondrial haplotype present in each clonal culture. Only 3 sites were called as 'heterozygous', showing evidence of two variants present within the single culture. These three 'heterozygous' sites were identified in three different individuals (L5, L6 and L9), each carrying a single 'heterozygous' site.

We sought to roughly estimate how many sites genotyped as heterozygous we would expect to observe under contamination between individuals L2-L11. For this, we computed the inter-individual pairwise distances between the mitochondrial haplotypes of L2-L11 using only those sites that were called as homozygous in each individual. The proportion of mitochondrial sites at which individuals L2-L11 differed from one another was low: only 214 out of the 13,764 sites (excluding the 3 sites with heterozygous calls and one multi-allelic site) were variable. Still, each individual among L2-L11 carried a number of sites at which it was different from the rest of the L2-L11 individuals; the number of such sites ranged from 5 for L2 to 57 for L11 (Supplementary Table 13). The minimal pairwise distance computed as the number of mitochondrial sites (out of the 13,764 assessed) at which two individuals were different (i.e. one was genotyped as '0/0' and the other as '1/1') was 11 (for individuals L2-L10), and most pairs of individuals were different at ≥ 20 sites (Supplementary Table 15). Using the L3 mitochondrial contig as the reference sequence produced nearly identical results (Supplementary Tables 14 and 16). Therefore, cross-sample contamination between L2-L11 would be expected to result in a sample with at least 11 'heterozygous' sites, and a mean of 57.9 such sites (Supplementary Table 15). However, among the three samples with detected mitochondrial heterogeneity (L5, L6 and L9, see above), each sample carried only one heterogeneous site. Therefore, mitochondrial heteroplasmy, accumulated mutations between individuals in a clonal culture, or technical artifacts (such as errors in base calling, e.g. due to nuclear mitochondrial pseudogenes[59]) appear to be more likely explanations for these three cases than contamination[57,58].

We also checked for the presence of heterogeneous mitochondrial sites in L1. For this, we aligned HiSeq reads for L1 to L1 mitochondrial contigs (extracted from the L1 assembly based on MiSeq reads, see Supplementary Note 7). Note that HiSeq and MiSeq reads for L1 were obtained by sequencing two independent libraries. Visual inspection of alignments of L1 HiSeq reads confirmed the accuracy of MiSeq-based assembly of L1 mitochondrial contigs. Among the 10,716 genotyped sites of L1

contig8072 and contig11064 retained after filtering (13,531 sites if contig12085 was included), according to SAMtools/BCFtools variant calling, none displayed evidence for the presence of the second variant, and in all cases the L1 mitochondrial variant recovered from HiSeq reads was consistent with that present in the corresponding L1 contig. The BAM file with alignments of L1 HiSeq reads to L1 mitochondrial contigs is also available at https://doi.org/10.6084/m9.figshare.11396955.v2.

Still, it could be that the low levels of mitochondrial heterogeneity detected within individual clonal cultures are due to inability of the SAMtools/BCFtools pipeline to identify variants present in Illumina reads at medium and low frequencies. Although we were not specifically interested in low allele fraction mitochondrial variants most likely associated with mitochondrial heteroplasmy, large numbers of medium allele fraction variants could point to potential cross-culture contamination. Therefore, to more thoroughly test for the presence of heterogeneous mitochondrial sites, we also carried out calling of mitochondrial variants using a somatic SNP caller, Mutect2, tuned to detect somatic mutations including those present at low allele fractions[60]. Mutect2 does not allow joint genotype calling of different individuals and does not report sites at which the individual matches the reference sequence. Therefore, Mutect2 does not suit the purpose of comparing genotypes across multiple individuals. The power of Mutect2 lies in identification of sites where the presence of a non-reference variant is only supported by a few reads. Variant calling with Mutect2 (GATK version 4.1.2.0)[18,60] was run for each individual L1-L11 in the mitochondrial mode ("--mitochondria-mode true" option). For individuals L2-L11, variant calls were generated relative to the L4 mitochondrial contig, and for L1, relative to the L1 mitochondrial contigs. As previously, we did not consider indels, variants within 10 bp of an indel, or sites with DP < 50. Variants were filtered using the FilterMutectCalls tool (GATK version 4.1.2.0) also using mitochondrial filters ("--mitochondria-mode true" option). Variants with 'PASS' or 'weak_evidence' values in the FILTER column were retained for further analysis. We did not discard variants marked as 'weak_evidence' because this filter treats alternative and reference alleles asymmetrically: it usually removes heterogeneous sites with an alternative (non-reference) variant supported by few reads, but does not remove heterogeneous sites where the reference variant is present at low read counts. Next, we used the resulting data to look for mitochondrial heterogeneity within each sequenced culture in more detail. Specifically, for each individual, L1-L11, we tabulated the number of sites where Mutect2 identified two variants present in the aligned reads from this culture, such that the minor allele fraction variant was supported by no less than 3 reads aligned at the corresponding position. As expected, Mutect2 identified a larger number of potentially heterogeneous mitochondrial sites compared to SAMtools/BCFtools. Each individual carried from 1 (L7 and L10) to 11 (L6) such sites (Supplementary Table 17). However, in most cases the minor allele fraction variant was present only in a small proportion of reads (Supplementary Tables 17 and 18). For example, at 6 heterogeneous sites found in L2, an average of only 2.4% of reads (median 0.7%) supported the minor allele fraction variant (Supplementary Table 18). If we required that the minor allele fraction variant was supported by ≥ 1% of reads, per-individual numbers of heterogeneous sites significantly dropped: no individual carried more than 3 such sites (Supplementary Table 17). Consistent with the SAMtools/BCFtools results, well-supported heterogeneous sites defined as sites with the minor allele fraction variant supported by ≥ 10% of reads were found only in three individuals (Supplementary Table 17). These were the same three individuals

identified with SAMtools/BCFtools (L5, L6 and L9), the only difference being that Mutect2 detected two instead of one heterogeneous site in L9.

In summary, the results of this analysis are not suggestive of cross-sample contamination. Instead, they are consistent with what we would expect if the mitochondrial genome of each culture was largely homogeneous, with a few sites showing some heterogeneity due to heteroplasmy, differences accumulated between individuals of a clonal culture or technical artifacts.

Supplementary Note 9: Characterization of phased segments inferred to be incongruent in L4-L11

A large fraction of phased segments is expected to overlap with protein-coding genes as over 50% (101,294,782 out of 197,096,676) of genomic bases of the L1 diploid assembly are contained within the predicted exons or introns. We checked whether the segments inferred to be incongruent in L4-L11 were more likely than the genomic background to overlap with protein-coding regions and therefore more likely to carry functional variation.

The phased genomic segments used in the current study were reconstructed based on the haploid sub-assembly. As only those gene models predicted in the L1 diploid assembly that were fully contained within the boundaries of haploid contigs were transferred to the haploid sub-assembly, it is possible that some segments annotated as non-coding relative to the haploid sub-assembly could in fact overlap with protein-coding regions. To account for this, we first performed a BLAST search of all phased segments from the set A (n = 303) subjected to analysis of incongruence in L4-L11 as well as of 52 segments inferred to be incongruent in this set against the protein-coding transcripts (n = 61,531) predicted in the L1 diploid assembly. For the BLAST search, we used sequences of the phased segments reconstructed for L4. The analyzed segments were on average much shorter than the transcripts: the average size of the phased segment from the set A is 803.48 bp (median = 715 bp), while a protein-coding transcript spans an average of 1,646.24 bp (median = 1,203 bp). 52 segments identified as incongruent in L4-L11 were similar in size to the whole set of analyzed segments with the average size of 857.69 bp (median = 818.5 bp). Given this, we computed the number of segments (L4) with a high-identity BLAST hit (≥95%) to a protein-coding transcript (L1), such that a hit covered at least 30% of the considered segment. We did not detect the difference in the fractions of such segments between all segments from the set A (0.77; 233 out of 303) and segments inferred as incongruent (0.77; 40 out of 52), P = 0.9968, two-sample Z-test for proportions (two-sided). We also did not detect the difference in the fractions of such segments between segments from the set A remaining after exclusion of incongruent ones (0.77; 193 out of 251) and segments inferred as incongruent (0.77; 40 out of 52), P = 0.9962, two-sample Z-test for proportions (two-sided). Accordingly, P values for the one-sided test (testing against the alternative that the fraction of incongruent segments overlapping protein-coding regions is greater than that among all 303 segments from the set A or among those remaining after exclusion of incongruent ones) were also non-significant (P = 0.4984 and P = 0.4981 respectively). As such, incongruent segments do not appear to more frequently overlap with protein-coding regions of the genome than the whole set of analyzed segments or the segments remaining after exclusion of incongruent ones.

Additionally, we carried out annotation of SNPs residing within the 52 phased segments inferred to be incongruent in L4-L11. SNPs were annotated with VEP (version 96.3)[61] relative to the variant present in the haploid sub-assembly. If VEP reported multiple consequences for a SNP, only a single consequence was retained. For this, the consequences were ranked in the following order: splice site variants, stop gained variants, stop or start lost variants, missense variants, synonymous variants, intron variants, intergenic variants. Start and stop retained variants were regarded as synonymous variants. As our annotation was restricted to protein-coding regions of the *A. vaga* genome, all variants annotated as upstream or downstream gene variants were regarded as intergenic. The summary statistics on the numbers of SNPs falling in different functional categories for each incongruent segment are presented in Supplementary Data 8.

Supplementary Note 10: Estimation of the population-scaled mutation and recombination rates

Estimation of the population-scaled mutation rate

We estimated the population-scaled mutation rate, $4N_{\mathrm{e}}\mu$, where N_{e} is the effective population size, and μ is the mutation rate per nucleotide per generation, using the maximum likelihood approach implemented in the program mlRho (version 2.9)[28]. Estimates were obtained independently for each individual and are based on sites covered by no less than 20 reads in the considered individual. Estimates of $4N_{\mathrm{e}}\mu$ for the individuals belonging to the large cluster (L4-L11) ranged from 0.0072 to 0.0094 (Supplementary Table 8) with the average value equal to 0.0086. Estimates for the individuals from the small cluster L1-L3 were higher, reflecting a higher level of within-individual heterozygosity, and ranged from 0.0221 to 0.0226 (Supplementary Table 8). 95% confidence intervals (CIs) for the $4N_{\mathrm{e}}\mu$ estimates are provided in Supplementary Table 8.

Estimation of the population-scaled recombination rate

We sought to infer the population-scaled recombination rate, $4N_{\mathrm{e}}c$, where c is the recombination rate per nucleotide per generation, in *A. vaga* from the rate of LD decay among the individuals of the large cluster (L4-L11). For this, we estimated the rate of short-range decay of r^2 with physical distance among L4-L11 by applying nonlinear regression based on the equation[62] for the expected value of r^2 under mutation-recombination-drift model (assuming low mutation rate) with an adjustment for sample size (n):

$$E(r^2) = \left[\frac{(10 + C)}{(2 + C)(11 + C)}\right] \times \left[1 + \frac{(3 + C)(12 + 12C + C^2)}{n(2 + C)(11 + C)}\right], \quad (9)$$

where $C = 4N_{\mathrm{e}}c_{\mathrm{sites}}$, and c_{sites} is the recombination fraction between sites.

To estimate the rate of LD decay, we used pairs of SNPs residing within the maximal distance of 500 bp from each other, with at least 4 copies of the minor allele among the individuals L4-L11. We fit nonlinear regression in R using the script by Marroni *et al*[63]. The estimated values of $4N_{\mathrm{e}}c$ based on the SNPs from the phased dataset 1 and based on the SNPs belonging to the more rigorously filtered phased dataset 2 were 0.0160 and 0.0147 respectively. Corresponding 95% bootstrap

confidence intervals (CIs) for $4N_{\mathrm{e}}c$ based on 1,000 bootstrap replicates were 0.0157–0.0164 and 0.0141–0.0152.

We also used the simpler formula for the expected value of r^2 under the basic model of recombination-drift equilibrium[64] (without taking mutation into account and without adjusting for sample size). Under this model, the expectation of r^2 is given by:

$$E(r^2) = \frac{1}{1+C}. \quad (10)$$

The estimates of $4N_{\mathrm{e}}c$ produced with the nonlinear regression based on this formula were 0.0263 (95% bootstrap CI: 0.0259–0.0267) and 0.0262 (95% bootstrap CI: 0.0254–0.0271) for the phased dataset 1 and 2 respectively.

Additionally, we estimated the population-scaled recombination rate with the Wakeley's moment method[65], as implemented in LDhat (version 2.2)[33]. For this purpose, we first obtained Wakeley's estimates of the population-scaled recombination rate for all segments of the *A. vaga* genome harboring at least 5 non-singleton SNPs simultaneously phased in all individuals L4-L11 (n = 1,962). The Wakeley's estimate of the population-scaled recombination rate (computed for the whole analyzed region) was obtained separately for each genomic segment and normalized by the segment size. Those regions that were identified as outliers for the normalized Wakeley's estimate of the population-scaled recombination rate using the interquartile range method were excluded from the further analyses (n = 242).

We further removed from consideration regions with less than 20 phased non-singleton variants, which left us with 245 genomic segments. The median length of the segments belonging to the resulting dataset is 901 base pairs and the median number of non-singleton phased variants harbored by these segments is 26. These 245 segments belonged to 222 haploid contigs. For those haploid contigs that were represented by more than one segment, we computed the mean value of the normalized Wakeley's estimates across the corresponding segments, obtaining a single estimate for each of 222 haploid contigs. The median Wakeley's estimate of the population-scaled recombination rate across these 222 haploid contigs is 0.0499, which is largely consistent with the estimates based on the rate of LD decay.

Supplementary Note 11: Estimating hypothetical frequency of meiosis or HGT in the *A. vaga* population

Estimating hypothetical frequency of meiosis

To address the question of what incidence of meiosis would be required to explain the observed rate of LD decay in the absence of other types of recombination, we need to know c, the recombination rate per nucleotide per generation. c can be inferred from the ratio of the population-scaled recombination rate $4N_{\mathrm{e}}c$ to the population-scaled mutation rate $4N_{\mathrm{e}}\mu$, if μ, the mutation rate per nucleotide per generation, is known.

The estimates of the population-scaled recombination rate ($4N_{\mathrm{e}}c$) in individuals L4-L11 inferred from LD (r^2) decay were found to be of the order of 10^{-2} (ranging from 0.0147 to 0.0263; see section «Estimation of the population-scaled recombination rate» of Supplementary Note 10). The level of genetic variation suggests that $4N_{\mathrm{e}}\mu$ is also ~10^{-2}: estimates of the population-scaled mutation rate

$4N_e\mu$ obtained for the individuals belonging to the large cluster (L4-L11) ranged from 0.0072 to 0.0094 with the average value equal to 0.0086 (individual estimates with 95% confidence intervals are provided in Supplementary Table 8; see section «Estimation of the population-scaled mutation rate» of Supplementary Note 10).

Thus, $c \sim \mu$. Accurately estimating c requires knowledge of the exact mutation rate in *A. vaga*, on which there are unfortunately no data. If μ is within the range of 10^{-9}–10^{-8}, typical for multicellular eukaryotes[30,66], c is also ~10^{-9}–10^{-8}.

Finally, we can estimate what incidence of meiosis is needed, to obtain such values of c. Let us denote by G the total genome size in nucleotides and by n the number of chromosomes in a haploid set. If all chromosomes are of the same size and one crossover occurs per chromosome pair per meiotic event, the probability of meiosis per generation can be estimated as:

$$\frac{G \times c}{n}. \quad (11)$$

The number of nucleotides in the haploid *A. vaga* genome is ~10^8 and the diploid number of *A. vaga* chromosomes[67] $2n = 12$ ($n = 6$), therefore, the incidence of meiosis can be calculated as $\frac{G \times c}{n} = \frac{10^8 \times c}{6}$.

Hence, to obtain c within the range of 10^{-9}–10^{-8}, we need 1 meiosis in ~10–100 generations.

Therefore, to alone produce the observed LD decay, meiotic sex has to be rather common, which would be difficult to reconcile with the reported failure to detect males among several hundred thousands of bdelloid individuals[68]. Conceivably, our estimates of the required prevalence of meiosis could be inflated. There are several possible causes of this. First, if the true mutation rate in *A. vaga* is substantially below the assumed range of 10^{-9}–10^{-8}, the estimates of c and of meiosis frequency would be corrected downwards. However, in eukaryotes reports of such low mutation rates are restricted to unicellular species (e.g. *Saccharomyces cerevisiae* or *Chlamydomonas reinhardtii*)[69]. Second, reciprocal mitotic recombination and gene conversion can also contribute to LD decay[70,71]. This could affect our calculations based on the assumption that the only source of LD decay is reciprocal meiotic recombination and consequently bias upwards estimates of the prevalence of meiosis. Finally, it is possible that both meiotic recombination and horizontal gene transfer (HGT) contribute to LD decay.

Estimating hypothetical frequency of HGT

In addition, we estimated what approximate rate of HGT (transformation) would be required to explain the observed rate of LD decay in the absence of other forms of recombination. Under the transformation scenario, the population-scaled recombination rate $4N_e c$ (where c is the probability of recombination between adjacent sites) would depend on the transformation frequency.

Therefore, we first derived an expression for c, assuming that genetic exchange in *A. vaga* occurs by transformation.

Let us denote by $p(x)$ the distribution of lengths of DNA segments which are transferred between genomes in the course of transformation. Then, the probability that any given genomic site would experience transformation in a single generation is given by:

$$a \int_{1}^{\infty} x\, p(x)\, dx \qquad (12)$$

where a is the per nucleotide probability of a transformation event per generation, defined as T/G, where T is the expected number of transformation events per genome per generation and G is the genome size in nucleotides.

Next, let us consider a pair of sites A and B residing k nucleotides apart from each other. Since a transfer of a DNA segment simultaneously spanning sites A and B would not lead to recombination between them, a recombination event between sites A and B would require a transformation event affecting only one of these two sites.

If sites A and B are separated by more than x nucleotides ($x < k$), there could be no segments of the length x simultaneously spanning both sites. Consequently, in this case transformation with a segment of the length x affecting site A would always result in its recombination with site B. However, if the distance between A and B is less or equal to x ($x \geq k$), then the probability that a segment of the length x does not cover B given that it covers A (and, thus, that recombination takes place) is $\frac{k}{x}$.

Therefore, the probability of recombination between two sites given that one of the sites underwent transformation with the segment of the size x is:

$$\begin{cases} 1, & \text{if } x < k \\ \frac{k}{x}, & \text{if } x \geq k \end{cases} \qquad (13)$$

From equations (12) and (13) we can calculate the per generation probability of recombination between a pair of sites k nucleotides apart, $R(k)$.

$$R(k) = 2a \int_{1}^{k} x\, p(x)\, dx \; + \; 2ak \int_{k}^{\infty} p(x)\, dx \qquad (14)$$

Equation (14) could be used to obtain the probability of recombination between two adjacent sites $c = R(1)$:

$$c = R(1) = 2a \qquad (15)$$

In other words, with transformation the probability of recombination between adjacent sites is simply twice the per nucleotide per generation rate of transformation. Hence, the rate of recombination between adjacent sites can be used to infer the per generation rate of transformation.

The estimates of the population-scaled recombination rate ($4N_{e}c$) in the *A. vaga* population (L4-L11) inferred from LD (r^2) decay were on the order of 10^{-2} (ranging from 0.0147 to 0.0263; see section «Estimation of the population-scaled recombination rate» of Supplementary Note 10). Assuming μ, the mutation rate per nucleotide per generation, ~10^{-9}–10^{-8} (see section «Estimating hypothetical

frequency of meiosis» of this Note), this corresponds to c, as well as to a (from eq. (15)), also of the order of 10^{-9}–10^{-8}. Given that the number of nucleotides in the haploid *A. vaga* genome is ~10^8, this translates to 1 transformation event per ~1–10 generations. If true μ is below the assumed range (see section «Estimating hypothetical frequency of meiosis» of this Note), the estimate of the required transformation frequency would be adjusted downwards.

Assuming that transformation is a means of interindividual genetic exchanges in *A. vaga*, the probability of recombination between a pair of sites is expected to increase with increasing distance between the sites, k, only while k stays below the maximal length of the transferred segment, L_{M}. This is due to the fact that for sufficiently large values of k ($k > L_{\mathrm{M}}$), the probability of recombination between the pair of sites is the same, irrespective of the exact value of k, as there are no more segments that can simultaneously span both sites.

Supplementary Discussion

On inferring negative and positive selection in *A. vaga*

As discussed in the main text, individuals from the small (L1-L3) and the large (L4-L11) clusters exhibit notable difference in the levels of intraindividual heterozygosity: the average genome-wide fraction of heterozygous sites per individual is 1.98% for the small cluster but only 0.63% for the large cluster (Supplementary Data 2 and Supplementary Table 8; Supplementary Fig. 9; Supplementary Methods). The corresponding values for silent (four-fold synonymous) sites of the protein-coding regions are 3.75% and 1.21% respectively (Supplementary Data 2). The average ratios of heterozygosity at replacement (zero-fold synonymous) relative to silent (four-fold synonymous) sites for the individuals from the two clusters are similar (0.30 for the small and 0.28 for the large cluster); these values are close to those observed in human[72] but higher than those in *Drosophila*[73] and those observed in some other multicellular eukaryotes[74], possibly indicating relaxation of natural selection against deleterious mutations in *A. vaga*. Overall, a detailed study of negative and positive selection in this species remains an important avenue for future research. Here, to reduce the potential effect of population structure, we focused most of the subsequent analysis on the 8 individuals (L4-L11) forming the large cluster.

On inferring congruent and incongruent groupings of haplotypes in L4-L11

Our analysis of haplotype groupings in *A. vaga* individuals L4-L11 is based on the subset of phased genomic segments for which we were able to identify reciprocal closest counterparts for both haplotypes of at least one individual. Specifically, among 303 phased genomic segments initially selected for the analysis of haplotype groupings, reciprocal closest counterparts for both haplotypes of at least one individual were identified in 90 segments (see Methods). This subset of 303 segments was further used to look for congruent and incongruent haplotype groupings.

Among these 90 segments, only in 12 segments we found a pair of individuals such that their haplotypes represented reciprocal closest counterparts (congruent grouping). By contrast, in 79 segments, we found at least one individual such that its two haplotypes had reciprocal closest counterparts in two different individuals (incongruent grouping; for one segment, both a congruent and an incongruent grouping were observed for different individuals). In 52 of the 79 ‘incongruent’ segments, and 10 of the 12 ‘congruent’ segments, the corresponding groupings received decent bootstrap support (≥70%). Importantly, our ability to identify incongruence using the outlined approach is limited as haplotypes can still be involved in incongruent groupings, even though it is not possible to assign haplotypes to pairs of most closely related neighbors. Therefore, 52 out of 303 phased regions exhibiting clear incongruent groupings of haplotypes provide a lower bound for a proportion of ‘incongruent’ segments.

Even those segments showing congruent groupings of the two haplotypes of the same individual exhibited different patterns of such haplotype groupings across segments, suggesting that there was no dominant underlying topology among phased genomic regions (Supplementary Data 5). For example, among the 4 segments with congruent groupings for L4, in the first segment, both haplotypes of L4 were clustered with haplotypes of L5; in the second segment, with haplotypes of L8; in the third segment, with haplotypes of L9; and in the fourth segment, with haplotypes of L10 (Supplementary Data 5). That is, congruence was preserved only within the segment, but not among segments, in line with other observations arguing against clonality.

These findings also argue against *Oenothera*-like meiosis in *A. vaga*. While we cannot rule out the possibility that the phylogenetic incongruence observed in our data resulted from *Oenothera*-like meiosis accompanied by conversion and/or reciprocal mitotic recombination[75,76] (see below and Supplementary Fig. 26), it appears to be a non-parsimonious explanation for our data. Both HGT and conventional meiosis can explain the data more easily.

Gene conversion and patterns of incongruence in haplotype phylogenies

Here, we describe the rationale used to distinguish signatures of genetic exchange from those of gene conversion in haplotype phylogenies.

Although gene conversion can introduce incongruence to phylogenies of haplotypes by increasing the similarity of the two haplotypes of a single individual to each other, it cannot increase the similarity between haplotypes harbored by different individuals[38].

Still, gene conversion can create spurious clustering of haplotypes from a single individual with the two haplotypes from different individuals[75,76]. To see this, consider two closely related individuals A and B (carrying at a given genomic segment haplotypes hapA.1/hapA.2 and hapB.1/hapB.2 respectively; Supplementary Fig. 25), and the third individual C (with haplotypes hapC.1/hapC.2 at this genomic segment) which is more distantly related to A and B. Under obligate asexual reproduction in the absence of gene conversion, we would expect the haplotypes of individuals A and B to form two pairs of similar haplotypes[77] hapA.1-hapB.1 and hapA.2-hapB.2 (the existence of such clustering in asexuals has been suggested by M. Meselson and is commonly referred to as the 'Meselson effect'[77]; Supplementary Fig. 25). However, if gene conversion replaced the sequence of one of the haplotypes in the lineage leading to individual B with the sequence of the other (converting hapB.1 to hapB.2), there would no longer exist a haplotypic counterpart of hapA.1 in individual B (Supplementary Fig. 25). In this case, one haplotype of individual A would be most similar to a haplotype from individual B, while the other, to a haplotype from individual C, as the corresponding haplotype in the lineage leading to individual B had been replaced by the sequence of its allelic counterpart.

However, the resulting phylogenetic signal of gene conversion can be distinguished from the signal of genetic exchange. In contrast to genetic exchange between individuals (we do not consider inbreeding here), gene conversion would make the distance separating the two haplotypes of individual B (hapB.1-hapB.2) shorter than the distances separating each of these haplotypes from its closest haplotypic neighbor in another individual (Supplementary Fig. 25). The cases in which this condition does not hold cannot arise from gene conversion, and by exclusion, have to be due to genetic exchange.

We employed this logic to identify cases of putative genetic exchange (see Methods; Table 1; Fig. 5) in the phased haplotype data for L4-L11 and found a signature of genetic exchange in 52 out of the 303 phased segments used for the analysis (only cases with bootstrap support ≥ 70% were considered).

The analyzed phased segments cover a small portion of the *A. vaga* genome (243,455 bp out of 76,679,421 bp included in the haploid sub-assembly). This is due to a high cut-off set on the number of non-singleton SNPs simultaneously phased in all individuals L4-L11 (we required a minimum of 15 such SNPs for a segment to be included in this analysis; see Methods) and further filtering of the segments. Still, ~17% of the analyzed segments exhibit a signature of genetic exchange inferred from incongruent groupings of the two haplotypes of a single individual. This proportion is

apparently a lower estimate, as we detect only those cases of incongruence where both haplotypes of an individual have unambiguous reciprocal best matches in different individuals and these groupings are well supported (bootstrap support ≥ 70%). That means that those cases when a haplotype is equally closely related to haplotypes from two or more different individuals are not included, as in such situation there are no unambiguous reciprocal best matches. We also do not infer incongruence in those cases when a haplotype has an unambiguous reciprocal best match in terms of genetic distance in another individual (H1-H1´), but a handful of highly similar haplotypes exist, making grouping of H1-H1´ on the phylogeny poorly supported.

***Oenothera*-like meiosis and signatures of recombination**

Here, we discuss different evolutionary scenarios that could give rise to the observed signatures of recombination.

Unlike conventional meiosis and HGT (transformation), *Oenothera*-like meiosis alone is not expected to cause a decline in LD with distance and create other signatures of recombination as it involves no crossing over at the majority of the genome[38]. However, it is conceivable, that gene conversion between allelic regions would suffice to explain a decay of LD irrespective of the mode of reproduction. Furthermore, pairs of sites passing the modified four-gamete test in principle could also arise under *Oenothera*-like meiosis as well as under conventional meiosis or HGT. Obviously, reciprocal meiotic recombination or incorporation of external DNA segments occurring during transformation can produce a pair of individuals, each heterozygous at two loci, carrying all four haplotypes (Fig. 3b; Supplementary Fig. 26a). On the contrary, gene conversion alone cannot give rise to such a pair. This is the basis of the modified four-gamete test employed in this study to distinguish gene conversion from other types of recombination (Supplementary Note 3). Nevertheless, homologous recombination during conventional meiosis or transformation is not a prerequisite for the existence of pairs of sites passing the modified four-gamete test. First, gene conversion in conjunction with sexual reproduction involving *Oenothera*-like meiosis would suffice to explain the existence of such pairs of sites (Supplementary Fig. 26b). Second, reciprocal mitotic recombination can also in principle give rise to such pairs of sites.

Although we cannot rule out the possibility that signatures of recombination as well as incongruence observed in the current study stem from gene conversion and/or reciprocal mitotic recombination[70,71,75,76] accompanied by *Oenothera*-like meiosis, it is not a parsimonious explanation, as it is sufficient to assume the presence of a single mechanism of genetic exchange involving recombination and introducing different patterns of incongruence at different loci such as conventional meiosis or HGT to explain the data.

Supplementary Figures

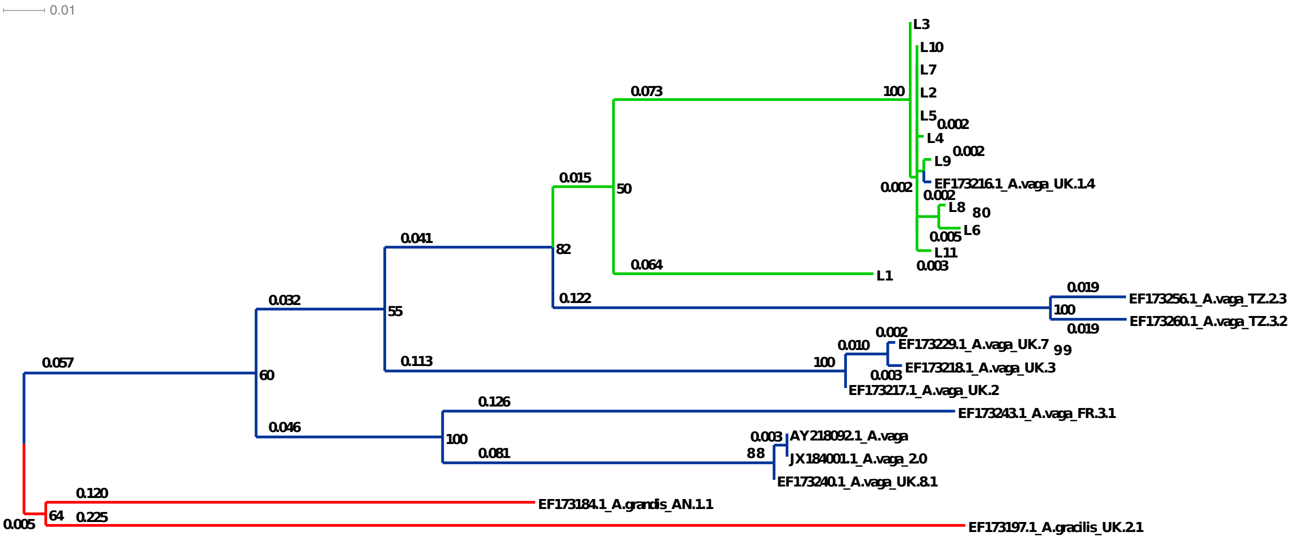

Supplementary Fig. 1 Phylogenetic tree for the individuals L1-L11 and reference *Adineta* isolates based on the *COX1* marker. The phylogenetic tree was constructed based on partial *COX1* sequences using the maximum likelihood method in RAxML[42] under the GTR+G model with 1,000 bootstrap replicates and visualized in Dendroscope[43]. Branches leading to individuals L1-L11 sequenced in the current study are shown in green, branches leading to reference *A. vaga* isolates from previous works[1,37] are in blue, branches leading to other species from the genus *Adineta* are in red. The reference *A. vaga* strain sequenced to produce the first *A. vaga* genome assembly[1] is designated as JX184001.1_A.vaga_2.0. The bootstrap support values and branch lengths are not shown on very short branches. Source data are provided as a Source Data file.

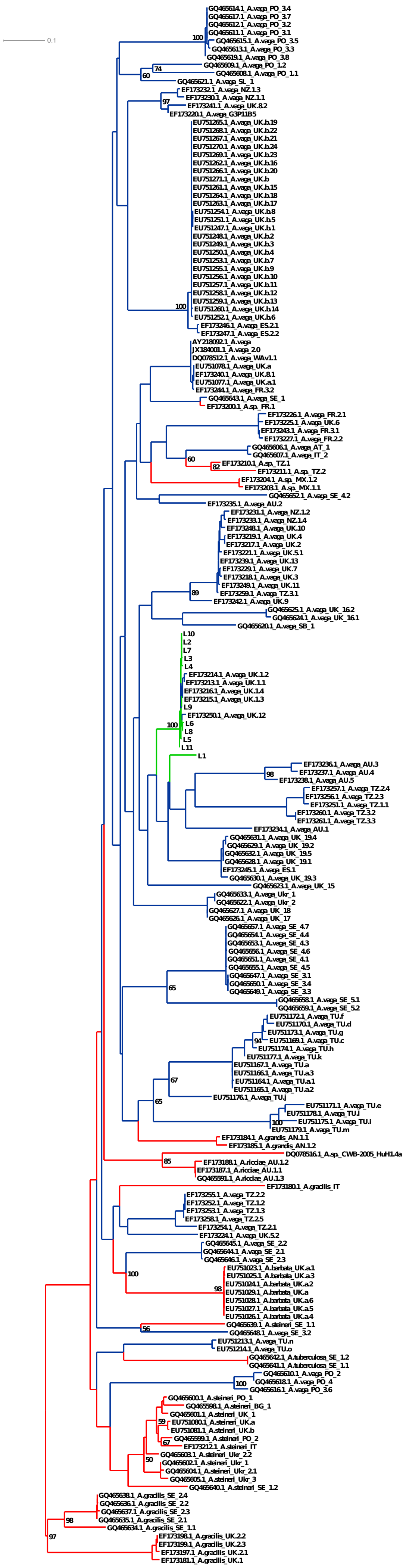
0.1
GQ465614.1_A.vaga_PO_3.4
GQ465617.1_A.vaga_PO_3.7
GQ465612.1_A.vaga_PO_3.2
GQ465611.1_A.vaga_PO_3.1
GQ465615.1_A.vaga_PO_3.5
GQ465613.1_A.vaga_PO_3.3
GQ465619.1_A.vaga_PO_3.8
GQ465609.1_A.vaga_PO_1.2
GQ465608.1_A.vaga_PO_1.1
GQ465621.1_A.vaga_SL_1
EF173232.1_A.vaga_NZ.1.3
EF173230.1_A.vaga_NZ.1.1
EF173241.1_A.vaga_UK.8.2
EF173220.1_A.vaga_G3P11B5
EU751265.1_A.vaga_UK.b.19
EU751268.1_A.vaga_UK.b.22
EU751267.1_A.vaga_UK.b.21
EU751270.1_A.vaga_UK.b.24
EU751269.1_A.vaga_UK.b.23
EU751262.1_A.vaga_UK.b.16
EU751266.1_A.vaga_UK.b.20
EU751271.1_A.vaga_UK.b
EU751261.1_A.vaga_UK.b.15
EU751264.1_A.vaga_UK.b.18
EU751263.1_A.vaga_UK.b.17
EU751254.1_A.vaga_UK.b.8
EU751251.1_A.vaga_UK.b.5
EU751247.1_A.vaga_UK.b.1
EU751248.1_A.vaga_UK.b.2
EU751249.1_A.vaga_UK.b.3
EU751250.1_A.vaga_UK.b.4
EU751253.1_A.vaga_UK.b.7
EU751255.1_A.vaga_UK.b.9
EU751256.1_A.vaga_UK.b.10
EU751257.1_A.vaga_UK.b.11
EU751258.1_A.vaga_UK.b.12
EU751259.1_A.vaga_UK.b.13
EU751260.1_A.vaga_UK.b.14
EU751252.1_A.vaga_UK.b.6
EF173246.1_A.vaga_ES.2.1
EF173247.1_A.vaga_ES.2.2
AY218092.1_A.vaga
JX184001.1_A.vaga_2.0
DQ078512.1_A.vaga_WAv1.1
EU751078.1_A.vaga_UK.a
EF173240.1_A.vaga_UK.8.1
EU751077.1_A.vaga_UK.a.1
EF173244.1_A.vaga_FR.3.2
GQ465643.1_A.vaga_SE_1
EF173200.1_A.sp._FR.1
EF173226.1_A.vaga_FR.2.1
EF173225.1_A.vaga_UK.6
EF173243.1_A.vaga_FR.3.1
EF173227.1_A.vaga_FR.2.2
GQ465606.1_A.vaga_AT_1
GQ465607.1_A.vaga_IT_2
EF173210.1_A.sp._TZ.1
EF173211.1_A.sp._TZ.2
EF173204.1_A.sp._MX.1.2
EF173203.1_A.sp._MX.1.1
GQ465652.1_A.vaga_SE_4.2
EF173235.1_A.vaga_AU.2
EF173231.1_A.vaga_NZ.1.2
EF173233.1_A.vaga_NZ.1.4
EF173248.1_A.vaga_UK.10
EF173219.1_A.vaga_UK.4
EF173217.1_A.vaga_UK.2
EF173221.1_A.vaga_UK.5.1
EF173239.1_A.vaga_UK.13
EF173229.1_A.vaga_UK.7
EF173218.1_A.vaga_UK.3
EF173249.1_A.vaga_UK.11
EF173259.1_A.vaga_TZ.3.1
EF173242.1_A.vaga_UK.9
GQ465625.1_A.vaga_UK_16.2
GQ465624.1_A.vaga_UK_16.1
GQ465620.1_A.vaga_SB_1
L10
L2
L7
L3
L4
EF173214.1_A.vaga_UK.1.2
EF173213.1_A.vaga_UK.1.1
EF173216.1_A.vaga_UK.1.4
EF173215.1_A.vaga_UK.1.3
L9
EF173250.1_A.vaga_UK.12
L6
L8
L5
L11
L1
EF173236.1_A.vaga_AU.3
EF173237.1_A.vaga_AU.4
EF173238.1_A.vaga_AU.5
EF173257.1_A.vaga_TZ.2.4
EF173256.1_A.vaga_TZ.2.3
EF173251.1_A.vaga_TZ.1.1
EF173260.1_A.vaga_TZ.3.2
EF173261.1_A.vaga_TZ.3.3
EF173234.1_A.vaga_AU.1
GQ465631.1_A.vaga_UK_19.4
GQ465629.1_A.vaga_UK_19.2
GQ465632.1_A.vaga_UK_19.5
GQ465628.1_A.vaga_UK_19.1
EF173245.1_A.vaga_ES.1
GQ465630.1_A.vaga_UK_19.3
GQ465623.1_A.vaga_UK_15
GQ465633.1_A.vaga_Ukr_1
GQ465622.1_A.vaga_Ukr_2
GQ465627.1_A.vaga_UK_18
GQ465626.1_A.vaga_UK_17
GQ465657.1_A.vaga_SE_4.7
GQ465654.1_A.vaga_SE_4.4
GQ465653.1_A.vaga_SE_4.3
GQ465656.1_A.vaga_SE_4.6
GQ465651.1_A.vaga_SE_4.1
GQ465655.1_A.vaga_SE_4.5
GQ465647.1_A.vaga_SE_3.1
GQ465650.1_A.vaga_SE_3.4
GQ465649.1_A.vaga_SE_3.3
GQ465658.1_A.vaga_SE_5.1
GQ465659.1_A.vaga_SE_5.2
EU751172.1_A.vaga_TU.f
EU751170.1_A.vaga_TU.d
EU751173.1_A.vaga_TU.g
EU751169.1_A.vaga_TU.c
EU751174.1_A.vaga_TU.h
EU751177.1_A.vaga_TU.k
EU751167.1_A.vaga_TU.a
EU751166.1_A.vaga_TU.a3
EU751164.1_A.vaga_TU.a1
EU751165.1_A.vaga_TU.a2
EU751176.1_A.vaga_TU.j
EU751171.1_A.vaga_TU.e
EU751178.1_A.vaga_TU.l
EU751175.1_A.vaga_TU.i
EU751179.1_A.vaga_TU.m
EF173184.1_A.grandis_AN.1.1
EF173185.1_A.grandis_AN.1.2
DQ078516.1_A.sp._CWB-2005_HuH1.4a
EF173188.1_A.ricciae_AU.1.2
EF173187.1_A.ricciae_AU.1.1
GQ465591.1_A.ricciae_AU.1.3
EF173180.1_A.gracilis_IT
EF173255.1_A.vaga_TZ.2.2
EF173252.1_A.vaga_TZ.1.2
EF173253.1_A.vaga_TZ.1.3
EF173258.1_A.vaga_TZ.2.5
EF173254.1_A.vaga_TZ.2.1
EF173224.1_A.vaga_UK.5.2
GQ465645.1_A.vaga_SE_2.2
GQ465644.1_A.vaga_SE_2.1
GQ465646.1_A.vaga_SE_2.3
EU751023.1_A.barbata_UK.a.1
EU751025.1_A.barbata_UK.a.3
EU751024.1_A.barbata_UK.a.2
EU751029.1_A.barbata_UK.a
EU751028.1_A.barbata_UK.a.6
EU751027.1_A.barbata_UK.a.5
EU751026.1_A.barbata_UK.a.4
GQ465639.1_A.steineri_SE_1.1
GQ465648.1_A.vaga_SE_3.2
EU751213.1_A.vaga_TU.n
EU751214.1_A.vaga_TU.o
GQ465642.1_A.tuberculosa_SE_1.2
GQ465641.1_A.tuberculosa_SE_1.1
GQ465610.1_A.vaga_PO_2
GQ465618.1_A.vaga_PO_4
GQ465616.1_A.vaga_PO_3.6
GQ465600.1_A.steineri_PO_1
GQ465598.1_A.steineri_BG_1
GQ465601.1_A.steineri_UK_1
EU751080.1_A.steineri_UK.a
EU751081.1_A.steineri_UK.b
GQ465599.1_A.steineri_PO_2
EF173212.1_A.steineri_IT
GQ465603.1_A.steineri_Ukr_2.2
GQ465602.1_A.steineri_Ukr_1
GQ465604.1_A.steineri_Ukr_2.1
GQ465605.1_A.steineri_Ukr_3
GQ465640.1_A.steineri_SE_1.2
GQ465638.1_A.gracilis_SE_2.4
GQ465636.1_A.gracilis_SE_2.2
GQ465637.1_A.gracilis_SE_2.3
GQ465635.1_A.gracilis_SE_2.1
GQ465634.1_A.gracilis_SE_1.1
EF173198.1_A.gracilis_UK.2.2
EF173199.1_A.gracilis_UK.2.3
EF173197.1_A.gracilis_UK.2.1
EF173181.1_A.gracilis_UK.1

Supplementary Fig. 2 Phylogenetic tree for the individuals L1-L11 and the extended set of reference *Adineta* isolates based on the *COX1* marker.
The phylogenetic tree was constructed based on partial *COX1* sequences using the maximum likelihood method in RAxML[42] under the GTR+G model with 1,000 bootstrap replicates and visualized in Dendroscope[43]. Branches leading to individuals L1-L11 sequenced in the current study are shown in green, branches leading to reference *A. vaga* isolates from previous works[1,37] are in blue, branches leading to other species from the genus *Adineta* are in red. The reference *A. vaga* strain sequenced to produce the first *A. vaga* genome assembly[1] is designated as JX184001.1_A.vaga_2.0. The bootstrap support values ≥ 50% are shown next to branches (some bootstrap support values ≥ 50% were hidden due to space limitations). Source data are provided as a Source Data file.

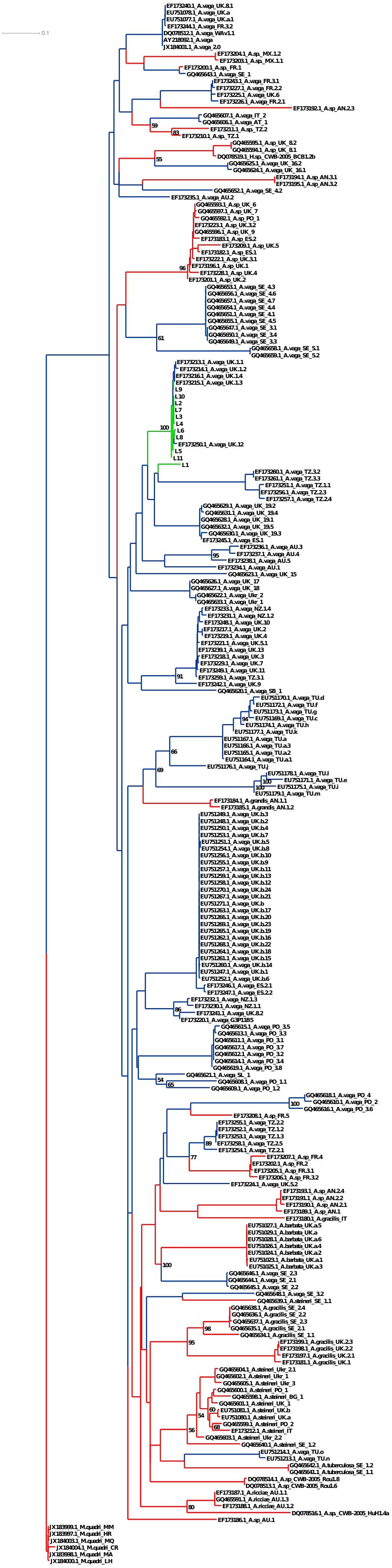

Supplementary Fig. 3 Phylogenetic tree for the individuals L1-L11 and the set of reference bdelloid isolates from the genera *Adineta* and *Macrotrachela* based on the *COX1* marker. The phylogenetic tree was constructed based on partial *COX1* sequences using the maximum likelihood method in RAxML[42] under the GTR+G model with 1,000 bootstrap replicates and visualized in Dendroscope[43]. Branches leading to individuals L1-L11 sequenced in the current study are shown in green, branches leading to reference *A. vaga* isolates from previous works[1,37] are in blue, branches leading to other bdelloid species (including unidentified *Adineta* species and *Macrotrachela*) are in red. The reference *A. vaga* strain sequenced to produce the first *A. vaga* genome assembly[1] is designated as JX184001.1_A.vaga_2.0. The bootstrap support values ≥ 50% are shown next to branches (some bootstrap support values ≥ 50% were hidden due to space limitations). Source data are provided as a Source Data file.

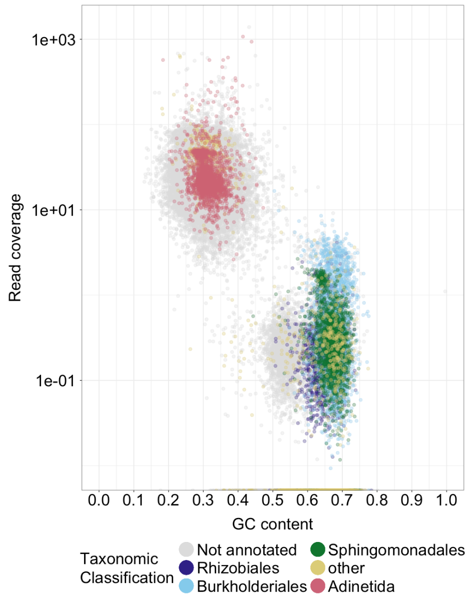

Supplementary Fig. 4 Taxonomic classification of contigs from the initial assembly of the *A. vaga* L1 genome. GC-content versus read coverage for the contigs from the initial genome assembly of lineage L1 from Illumina MiSeq reads. Read coverage was determined from Illumina HiSeq reads which were obtained from a separate library and not used for assembly (see Methods). Contigs are color coded based on taxonomic classification[78] of their BLAST hits to nt database with *E*-value cut-off set to 1×10^{-5}. Only taxonomic annotations ascribed to at least 6% of the contigs are displayed; unannotated contigs and contigs representing less abundant annotations are shown in grey and yellow respectively.

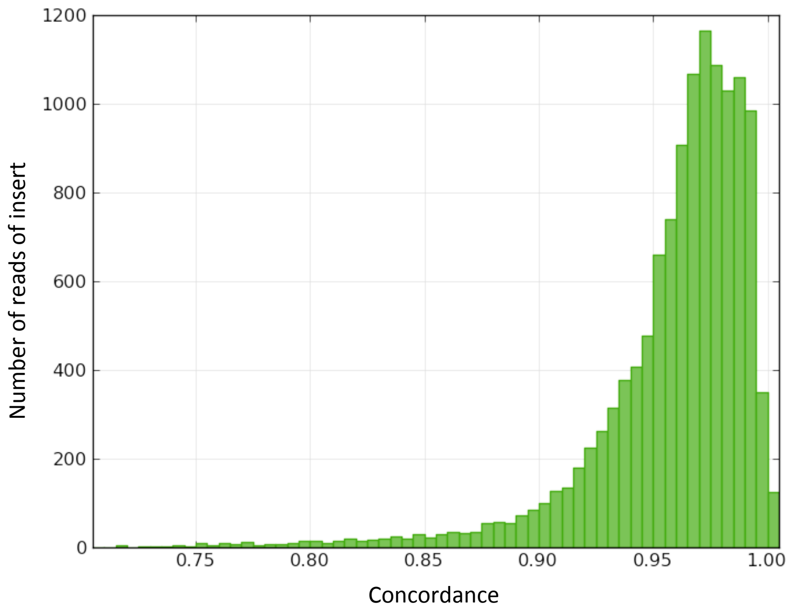

Supplementary Fig. 5 PacBio reads of insert concordance with the *A. vaga* L1 diploid genome assembly. Distribution of concordance for mapped PacBio reads of insert measured against the initial L1 diploid assembly.

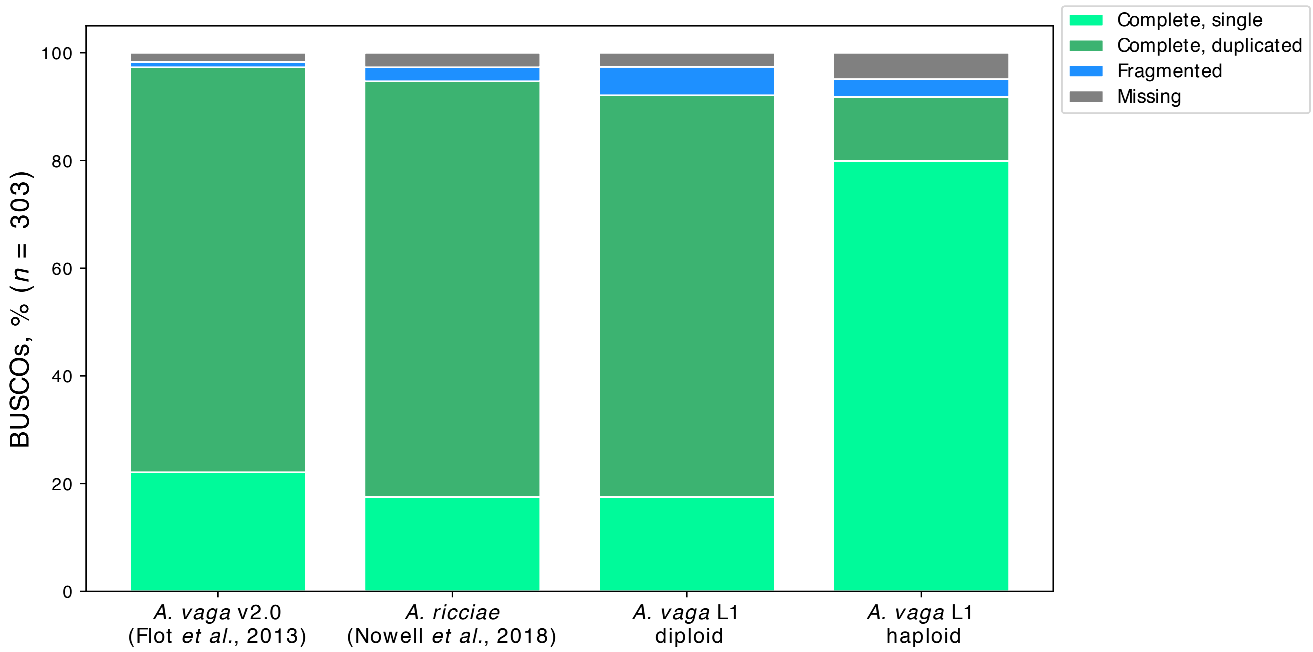

Supplementary Fig. 6 Results of BUSCO assessment (eukaryotic BUSCOs, *n* = 303) of the *A. vaga* L1 genome assembly as compared to the previously published bdelloid genomes.

Results of BUSCO assessment are shown for both L1 diploid assembly (*A. vaga* L1 diploid) and L1 haploid sub-assembly (*A. vaga* L1 haploid). For comparison, we used the first published genome assembly obtained for *A. vaga* by Flot *et al.*[1] (*A. vaga* v2.0) and the genome assembly for *A. ricciae* reported by Nowell *et al.*[2] (*A. ricciae*). Out of 303 eukaryotic BUSCO groups, 1.7% (*n* = 5), 2.6% (*n* = 8) and 2.6% (*n* = 8) were missing in *A. vaga* v2.0, *A. ricciae* and *A. vaga* L1 diploid assemblies respectively.

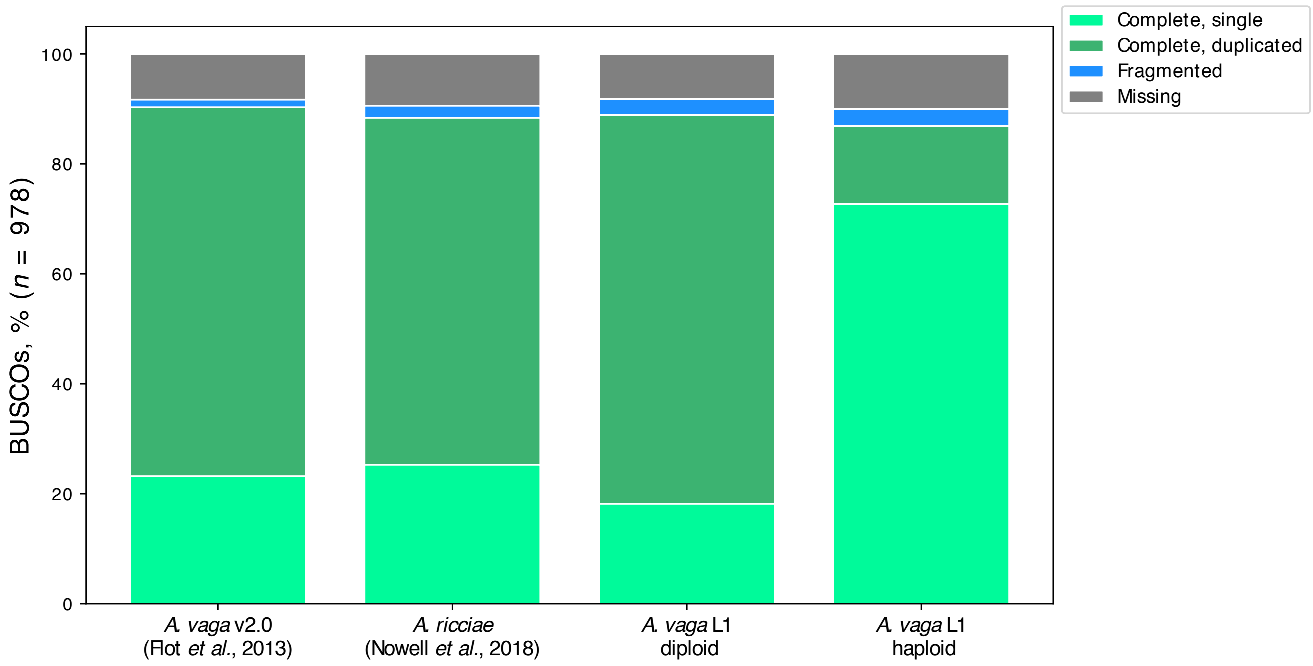

Supplementary Fig. 7 Results of BUSCO assessment (metazoan BUSCOs, *n* = 978) of the *A. vaga* L1 genome assembly as compared to the previously published bdelloid genomes.
Results of BUSCO assessment are shown for both L1 diploid assembly (*A. vaga* L1 diploid) and L1 haploid sub-assembly (*A. vaga* L1 haploid). For comparison, we used the first published genome assembly obtained for *A. vaga* by Flot *et al.*[1] (*A. vaga* v2.0) and the genome assembly for *A. ricciae* reported by Nowell *et al.*[2] (*A. ricciae*). Out of 978 metazoan BUSCO groups, 8.3% (*n* = 81), 9.4% (*n* = 92) and 8.3% (*n* = 81) were missing in *A. vaga* v2.0, *A. ricciae* and *A. vaga* L1 diploid assemblies respectively.

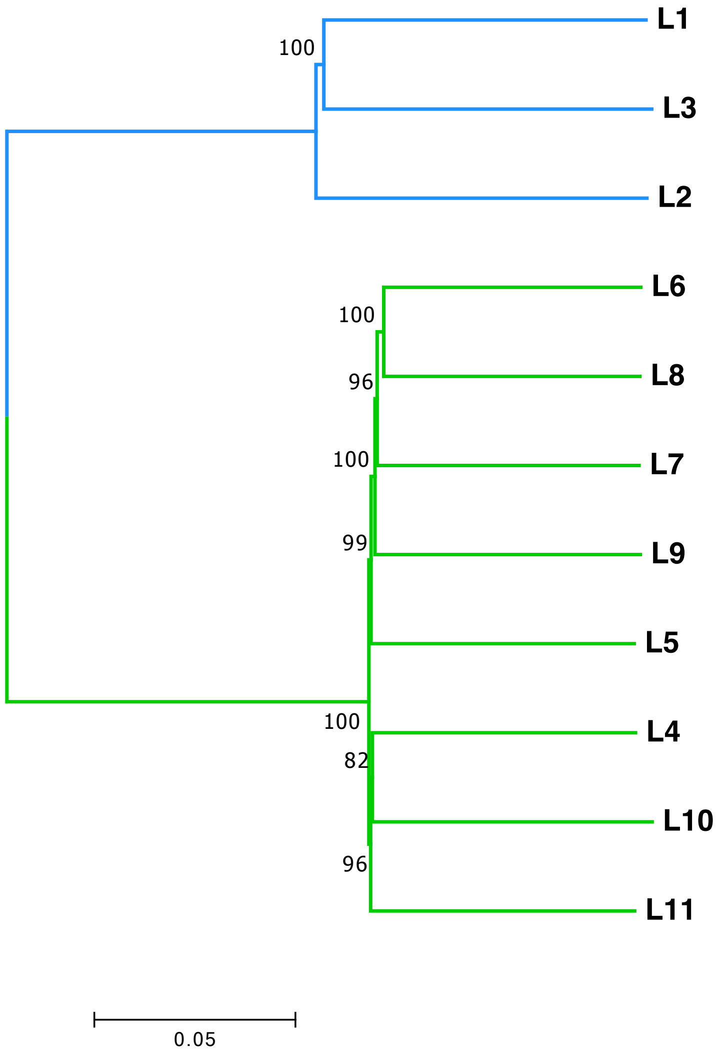

Supplementary Fig. 8 SNP-based neighbor-joining tree of individuals L1-L11. The tree is based on distances calculated from L1-L11 biallelic SNPs as fractions of alleles different between individuals. The tree was constructed using a thinned subset of L1-L11 biallelic SNPs from the stringent SNP dataset I (n = 449,218; see Supplementary Methods). Unrooted phylogenetic tree was obtained using the *aboot* function from the *poppr*[22,23] R package and rooted at the midpoint in MEGA7[79]. Bootstrap support values from 1,000 replicates (rounded to the nearest integer) are shown adjacent to nodes. Note that here fractions of different alleles are computed for biallelic sites (invariant sites are not included), therefore the distances are by construction significantly larger than those computed taking invariant sites into consideration (Supplementary Table 7). Branches leading to individuals of the small and the large cluster are shown in blue and green respectively. Source data are provided as a Source Data file.

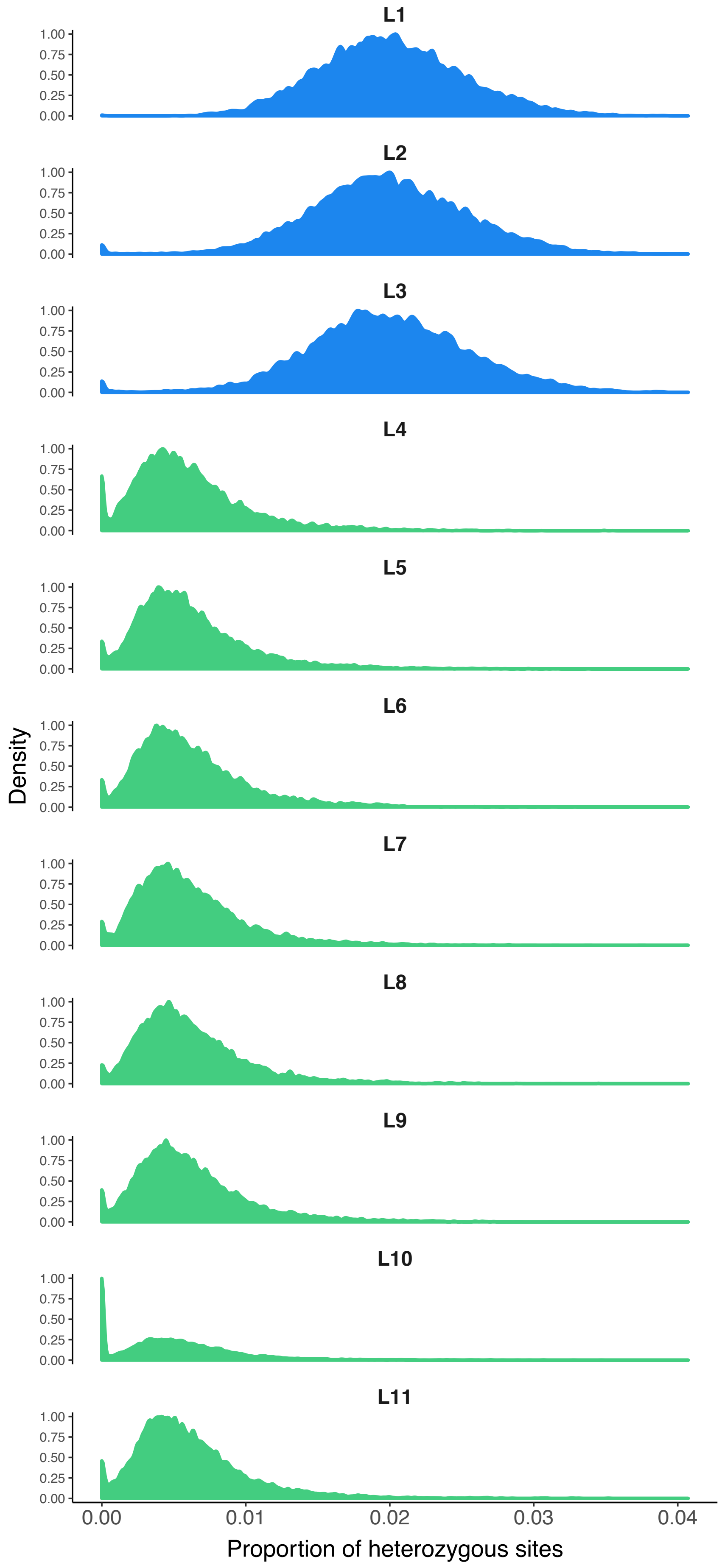
L1
L2
L3
L4
L5
L6
L7
L8
L9
L10
L11
1.00
0.75
0.50
0.25
0.00
Density
0.00
0.01
0.02
0.03
0.04
Proportion of heterozygous sites

Supplementary Fig. 9 Density distribution of heterozygosity in 5 kb windows in genomes of the sequenced *A. vaga* individuals. Normalized density distributions for proportions of heterozygous sites are shown for all 11 individuals of the small cluster (blue) and the large cluster (green).

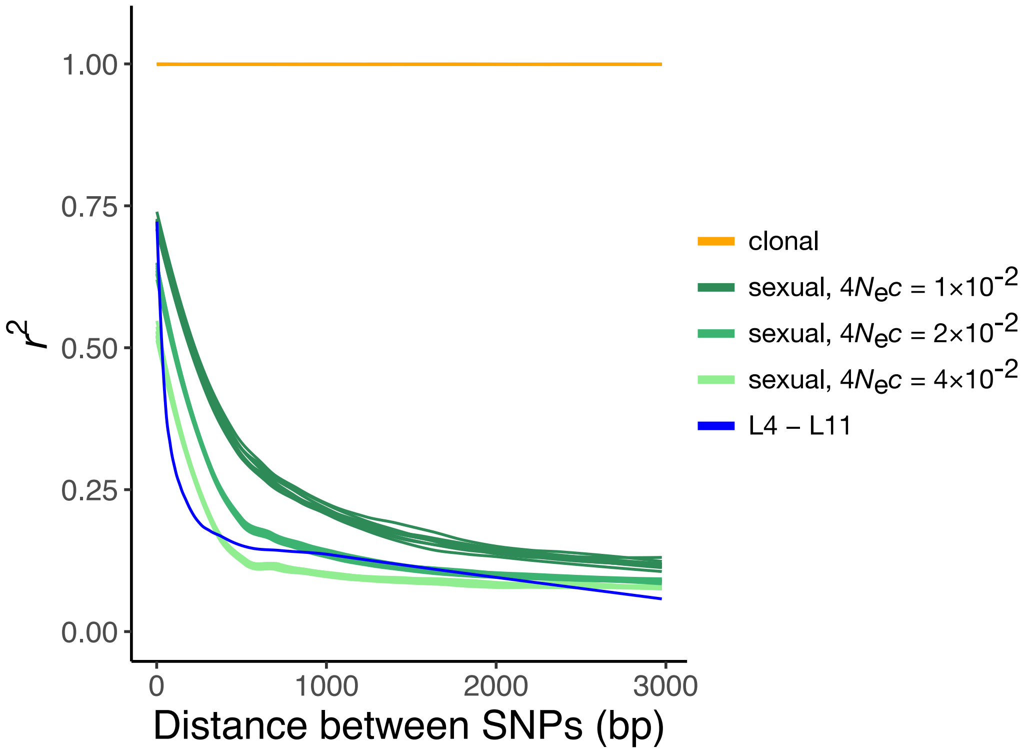

Supplementary Fig. 10 Dependence of LD on the physical distance in populations reproducing clonally or sexually. The figure shows second-degree LOESS regression curves of r^2 versus physical distance (smoothing parameter set to 0.4) in simulated populations and in individuals L4-L11. We simulated a strictly clonal population and strictly sexual populations in SLiM[80]. Parameters were chosen so that the population-scaled mutation rate $4N_e\mu$ was close to that estimated from the data (L4-L11; Supplementary Table 8): $N_e = 2{,}500$, $\mu = 10^{-6}$. Sexually reproducing populations were simulated with the population-scaled recombination rate $4N_ec$ equal to: 1×10^{-2}, 2×10^{-2} and 4×10^{-2}. All simulations were run for 200,000 generations and replicated 10 times. For each replicate of each simulation, we randomly chose 8 individuals (matching the number of the analyzed individuals L4-L11) and retained only those SNPs that had minor allele count ≥ 4. The data used for L4-L11 are the same as shown in Fig. 2a (biallelic SNPs with minor allele count ≥ 4). For each simulation, results for 10 replicates are shown. In the case of strictly clonal reproduction, differences between the replicates are not visible.

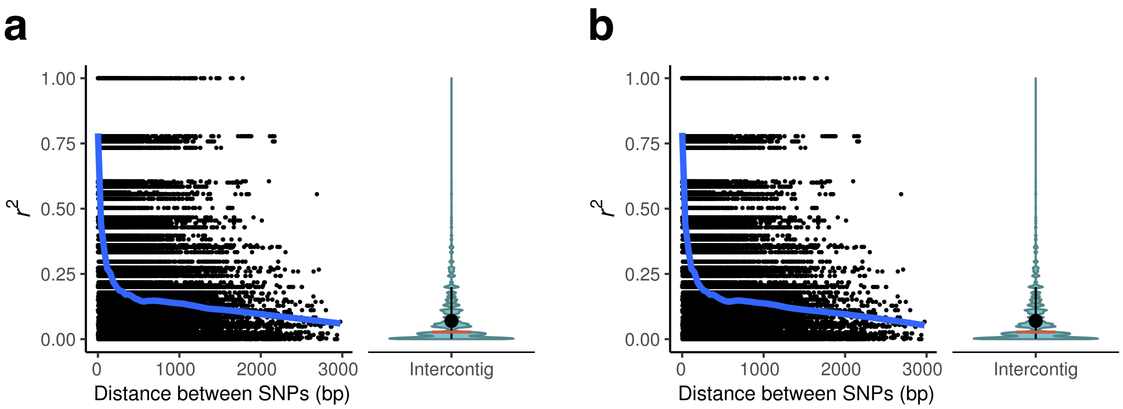

Supplementary Fig. 11 Decay of LD with physical distance among the individuals L4-L11 fitted by LOESS with the smoothing parameter selected according to the bias-corrected Akaike information criterion.

a, b, LD is measured as r^2. Decay of r^2 with physical distance is estimated using phased haplotype data (phased dataset 1). r^2 was calculated using biallelic sites residing within the segments of the reference genome where haplotypes had been reconstructed for all the individuals forming the large cluster (L4-L11, the data are the same as in Fig. 2a). First-degree (**a**) or second-degree (**b**) LOESS regression curves of r^2 versus physical distance are shown in blue. The smoothing parameter for LOESS curves was selected according to the bias-corrected Akaike information criterion using the *loess.as* function from the fANCOVA R package. The smoothing parameter is equal to 0.08198 for the first-degree LOESS (**a**) and 0.14138 for the second-degree LOESS (**b**). Violin plots show the distributions of r^2 values for the pairs of SNPs located on different contigs. Ends of the whiskers represent the 10th and 90th percentiles, with the mean and median values shown as a black dot and a red horizontal bar respectively.

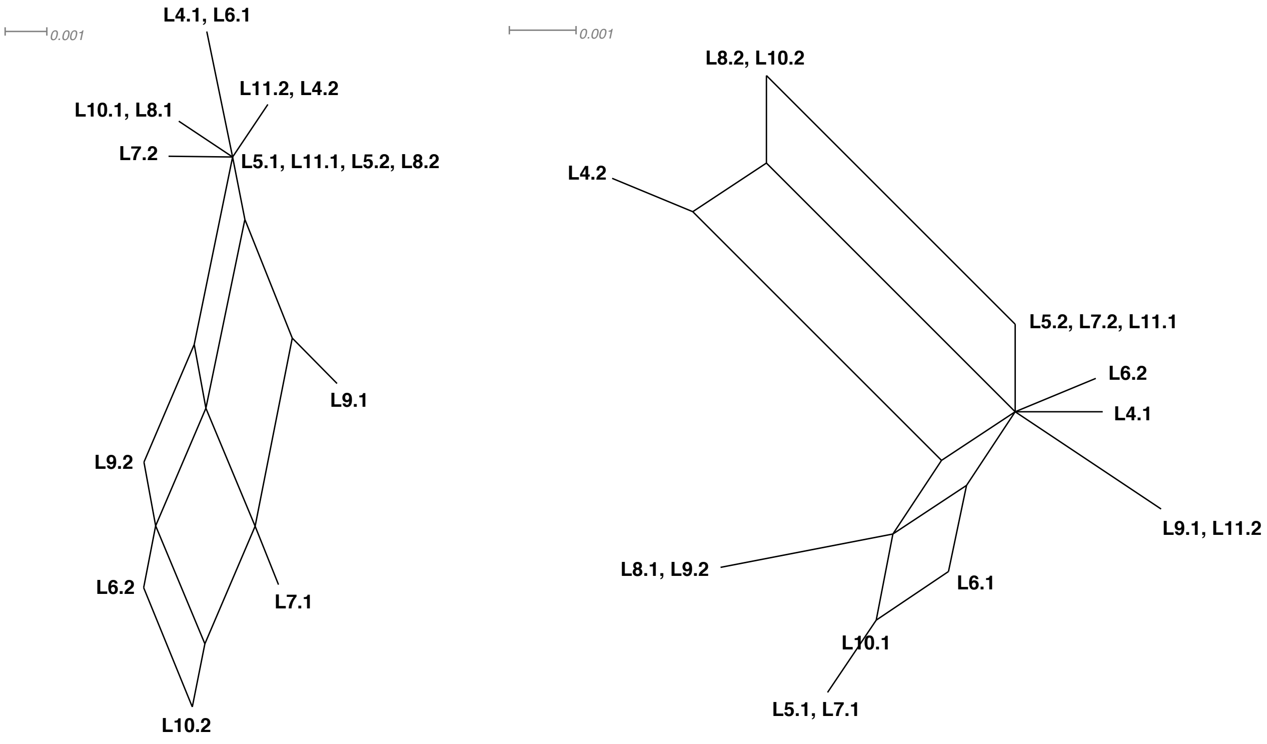

Supplementary Fig. 12 Examples of split decomposition networks for two phased segments showing significant evidence for recombination according to the PHI test. These two phased segments are located on contig1569 (left) and contig1342 (right) of the L1 diploid assembly. Split decomposition networks were constructed with SplitsTree[35]. Overall, according to the PHI test, the evidence for recombination was significant for 190 out of 434 segments (see Supplementary Methods). Indices 1 and 2 designate the two haplotypes of a single individual.

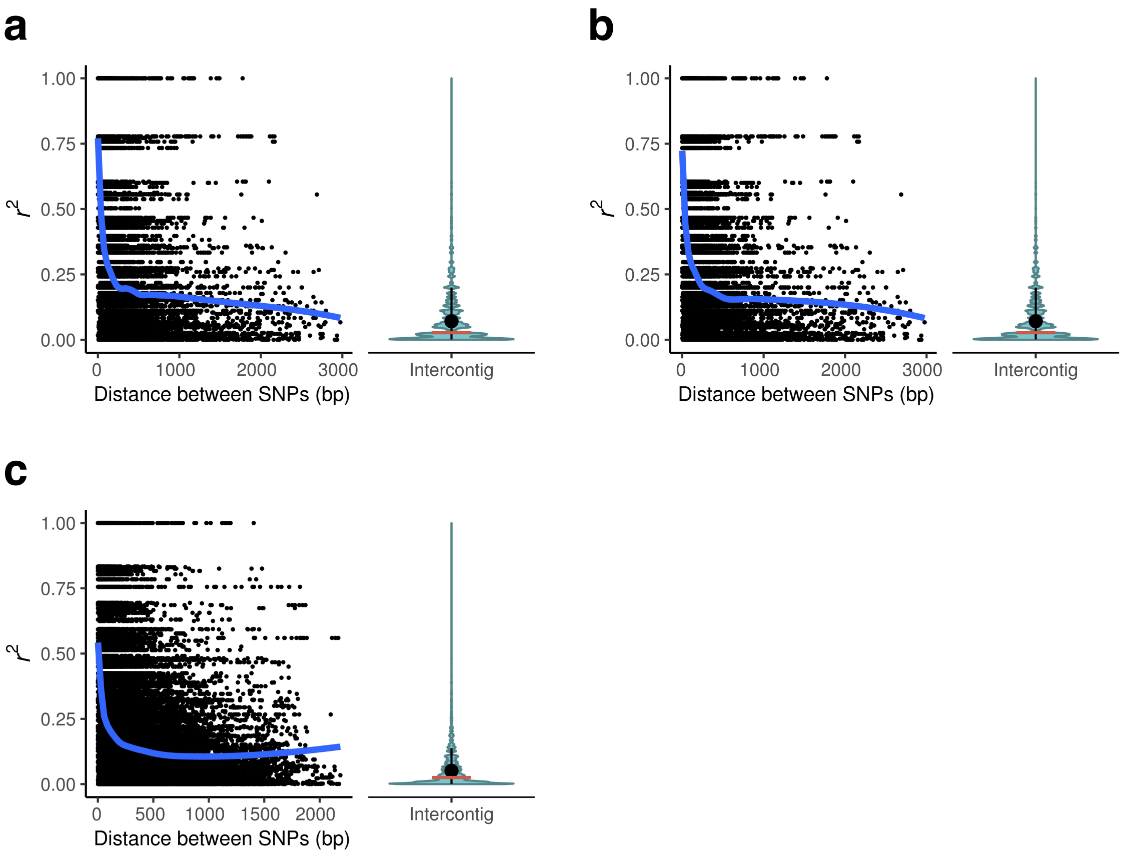

Supplementary Fig. 13 Decay of LD with physical distance among the sequenced *A. vaga* individuals is robust to erroneous read alignment, phasing errors and effects of population structure.

a, b, c, LD is measured as r^2. Decay of r^2 with physical distance is estimated using phased haplotype data. Second-degree LOESS regression curves of r^2 versus physical distance (smoothing parameter set to 0.4) are shown in blue. Violin plots show the distributions of r^2 values for the pairs of SNPs located on different contigs. Ends of the whiskers represent the 10th and 90th percentiles, with the mean and median values shown as a black dot and a red horizontal bar respectively.

a, b, Decay of LD with physical distance among the individuals L4-L11. r^2 was calculated using biallelic sites residing within the segments of the *A. vaga* genome where haplotypes had been reconstructed for all the individuals forming the large cluster (L4-L11). **a,** r^2 was calculated using biallelic sites from the phased dataset 1 residing within the allelic regions of the *A. vaga* genome (see Supplementary Methods). **b,** r^2 was calculated using biallelic sites from the stringently filtered phased dataset 2 (see Supplementary Methods). **c,** Decay of LD with physical distance among the individuals L1-L11. r^2 was calculated using biallelic sites from the phased dataset 1 residing within the segments of the *A. vaga* genome where haplotypes had been reconstructed for the complete set of individuals L1-L11.

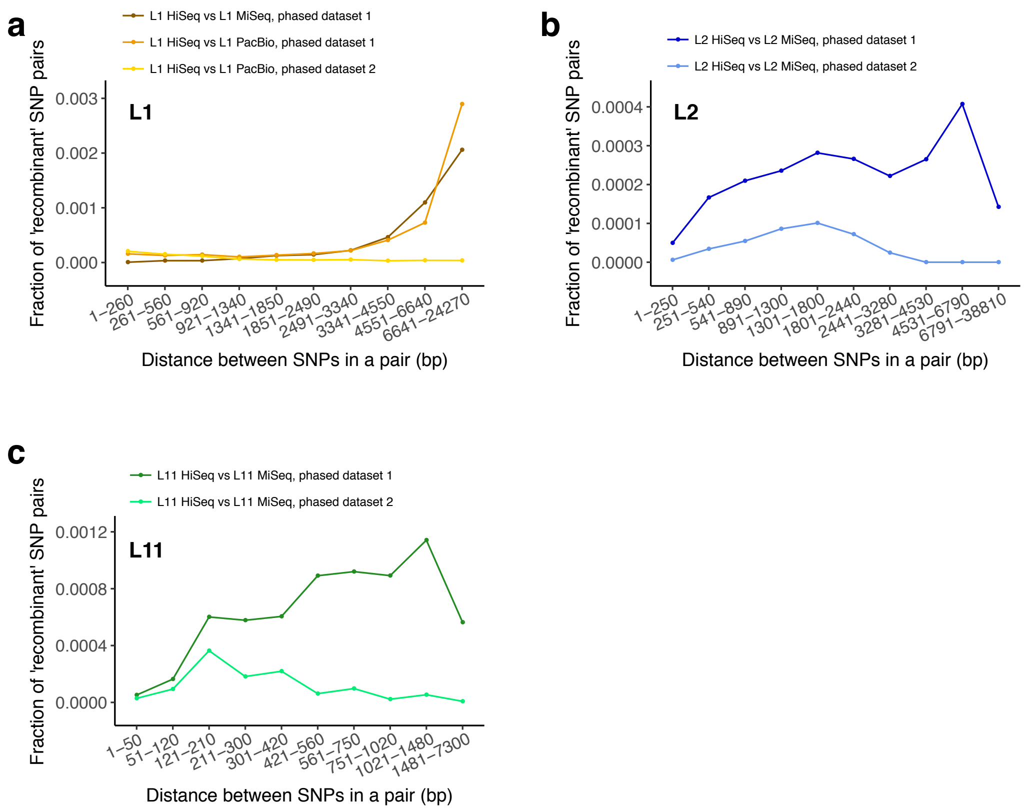

Supplementary Fig. 14 Phasing error rate as inferred from comparison of phased blocks assembled for the same individual from different sets of reads. The presented data are for the three individuals (L1 – **a**, L2 – **b**, L11 – **c**) for which more than one set of reads was available. To assess phasing error rate for different bins of distance, we applied the modified four-gamete test to the phased blocks reconstructed for the same individual from Illumina HiSeq and Illumina MiSeq reads (L1, L2 and L11) or from Illumina HiSeq and PacBio reads (L1). In this analysis, 'recombinant' SNP pairs correspond to SNP pairs exhibiting discordant phasing between the two considered phased datasets obtained for the same individual. For each individual, the distance bins are chosen in such a way that each bin contains a similar number of heterozygous SNP pairs for the pair of datasets with the fewest simultaneously phased heterozygous SNP pairs.

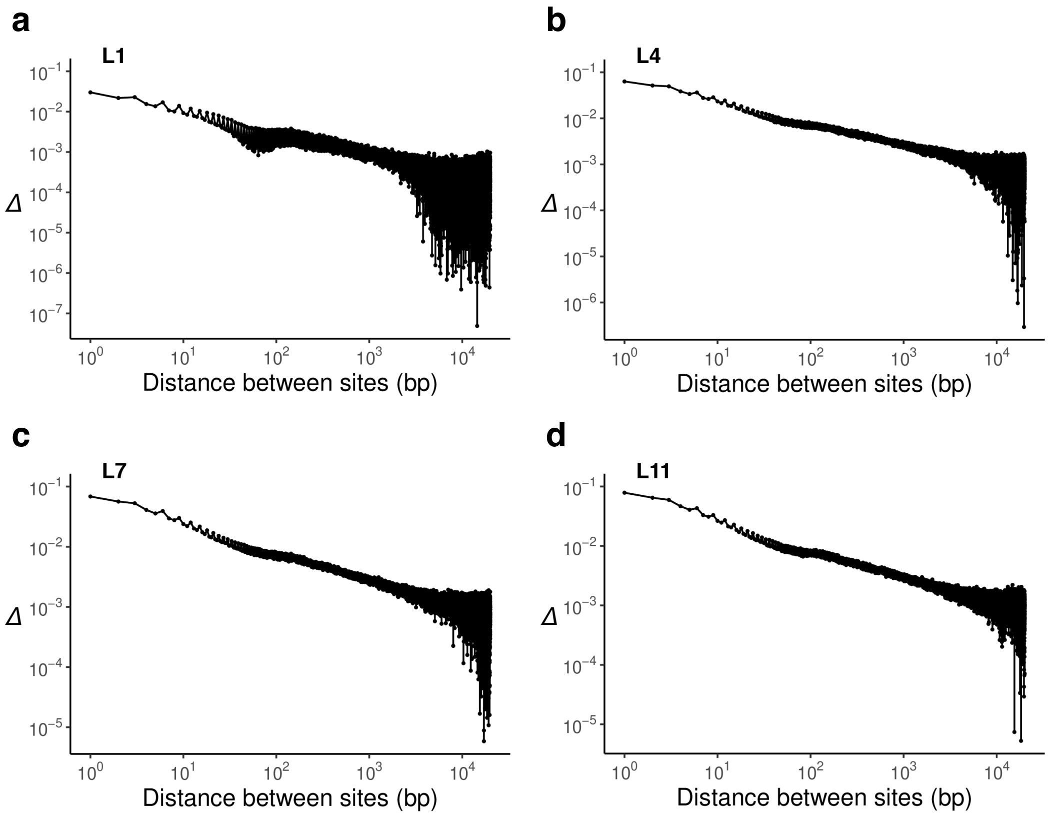

Supplementary Fig. 15 Decay of correlation of zygosity (Δ) in individual genomes with physical distance. Different panels show dependence of Δ on physical distance in genomes of four different individuals (L1 – **a**, L4 – **b**, L7 – **c**, L11 – **d**). Maximum likelihood estimates of Δ were obtained with mlRho[28] for each individual independently.

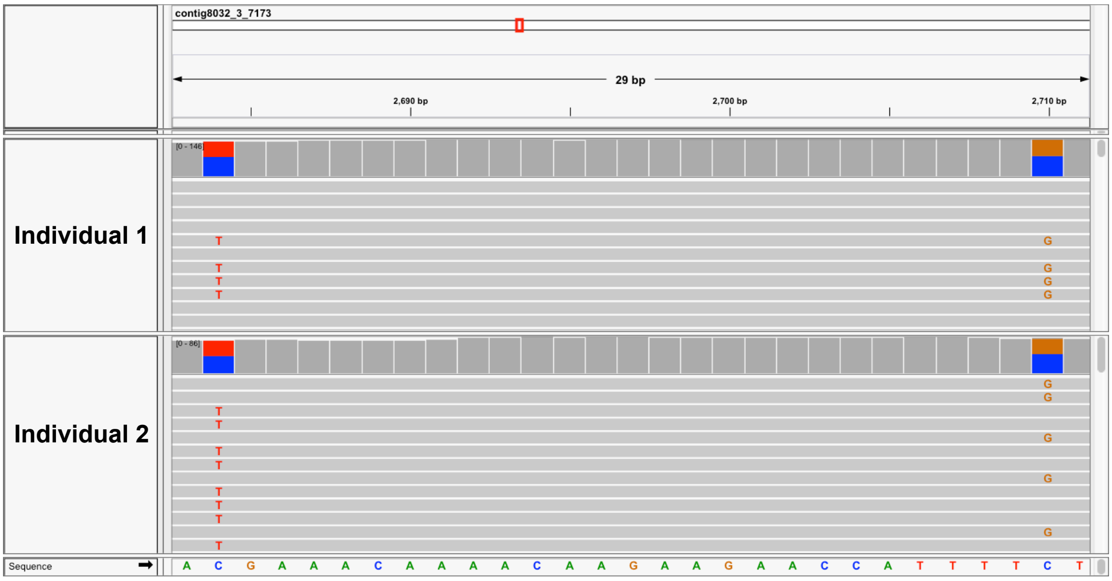

Supplementary Fig. 16 Screenshot from IGV genome browser showing an example of a pair of biallelic sites passing the modified four-gamete test. For a pair of SNPs, all four haplotypes are present in two individuals. Individual 1 carries haplotypes C-C and T-G, while Individual 2 carries haplotypes C-G and T-C. The displayed region is located on contig8032 of the L1 diploid assembly.

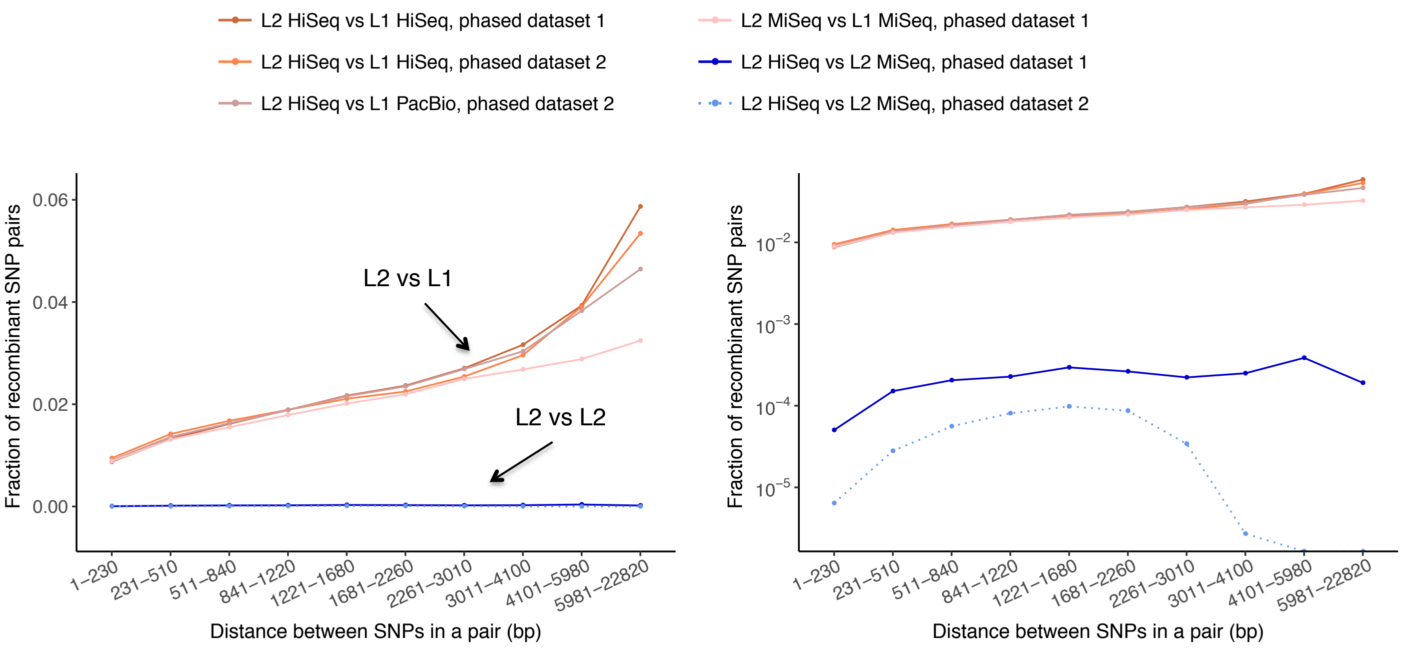

Supplementary Fig. 17 Results of the modified four-gamete test applied to phased SNPs from two different individuals (L2 and L1) and to SNPs of the same individual (L2) phased from different sets of reads. Recombinant pairs of SNPs detected when comparing different phased datasets obtained for the same individual are expected to result from phasing errors, while recombinant pairs of SNPs detected in different individuals are expected, in addition, to comprise those stemming from true recombination events. The plot in the right panel shows the same data as the one on the left, but uses a base 10 log scale for the y-axis.

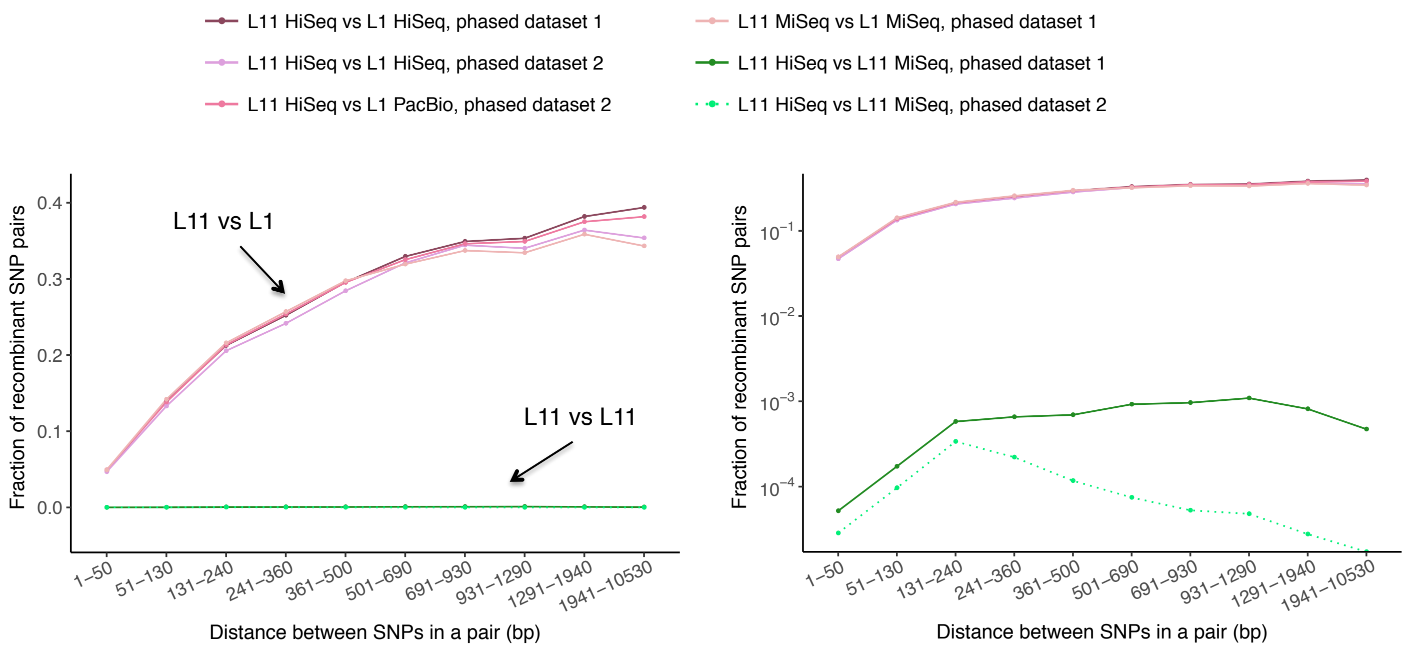

Supplementary Fig. 18 Results of the modified four-gamete test applied to phased SNPs from two different individuals (L11 and L1) and to SNPs of the same individual (L11) phased from different sets of reads. Recombinant pairs of SNPs detected when comparing different phased datasets obtained for the same individual are expected to result from phasing errors, while recombinant pairs of SNPs detected in different individuals are expected, in addition, to comprise those stemming from true recombination events. The plot in the right panel shows the same data as the one on the left, but uses a base 10 log scale for the y-axis.

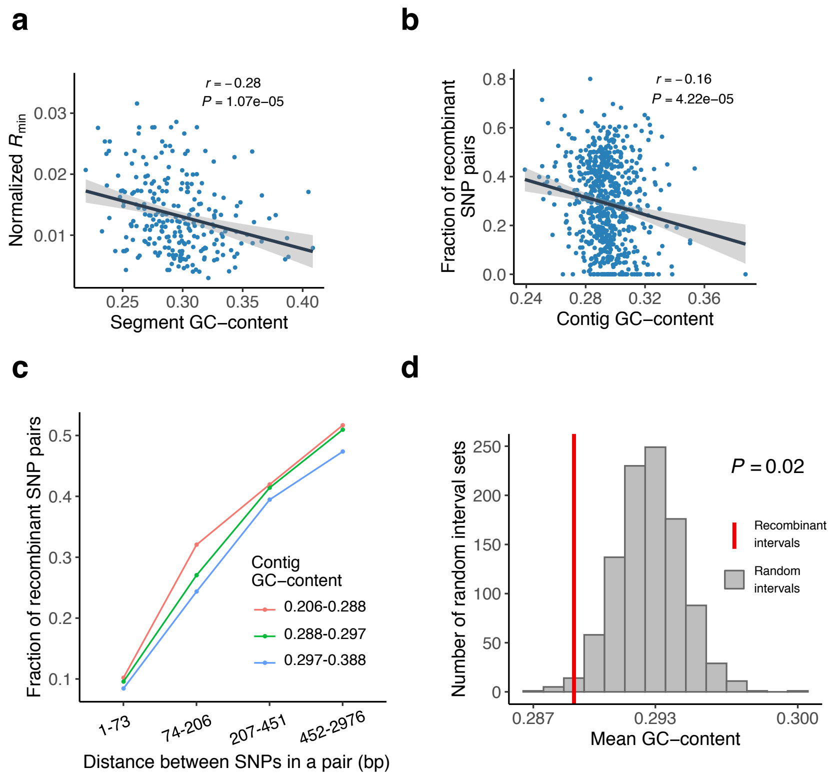

Supplementary Fig. 19 Recombination is skewed towards GC-poor regions of the *A. vaga* genome. a, Normalized minimum number of recombination events (R_{min}) in a genomic segment vs the GC-content of that genomic segment. The Pearson's $r = -0.28$ (95% CI: −0.39 to −0.16) and the two-sided P value (1.072×10^{-5}) for the correlation between normalized R_{min} and GC-content of genomic segments are reported. The P value is from the t-test (t-value = −4.50, 243 degrees of freedom). The line and the shaded area represent the best fit line from a linear regression of normalized R_{min} on the segment GC-content and the 95% confidence interval of alternative fits, respectively. Minimum numbers of recombination events were estimated for individual phased genomic segments harboring no less than 20 non-singleton SNPs phased in L4-L11 ($n = 245$; see Supplementary Note 4).
b, Fraction of recombinant SNP pairs in a haploid contig vs the GC-content of that contig. The Pearson's $r = -0.16$ (95% CI: −0.23 to −0.08) and the two-sided P value (4.221×10^{-5}) for the correlation between the fraction of recombinant SNP pairs and GC-content of haploid contigs are reported. The P value is from the t-test (t-value = −4.12, 664 degrees of freedom). The line and the shaded area represent the best fit line from a linear regression of the fraction of recombinant SNP pairs on the haploid contig GC-content and the 95% confidence interval of alternative fits, respectively. **c,** Fraction of SNP pairs passing the modified four-gamete test as a function of distance between SNPs in a pair and haploid contig GC-content. Colored lines correspond to 3 different bins of GC-content. Boundaries of GC-content bins are rounded to three decimal places. The data are the same as in Fig. 3c-d. **d,** Mean GC-content of genomic intervals residing between recombinant pairs of sites (recombinant intervals) compared to that of random genomic intervals. The vertical red line shows the mean GC-content across recombinant intervals (0.289) and the histogram represents the null expectation. The distribution was obtained by sampling 1,000 times random genomic intervals matched for the number (n = 1,014), length

and haploid contig identity to the recombinant intervals. The one-sided *P* value was calculated as the fraction, among 1,000 sets of random intervals, of those with mean GC-content lower or equal to that of the recombinant intervals (see Supplementary Note 4). The bin intersected by the red line corresponds to sets of random intervals with mean GC-content equal to that of the recombinant intervals (when rounded to three decimal places). Source data are provided as a Source Data file (**d**).

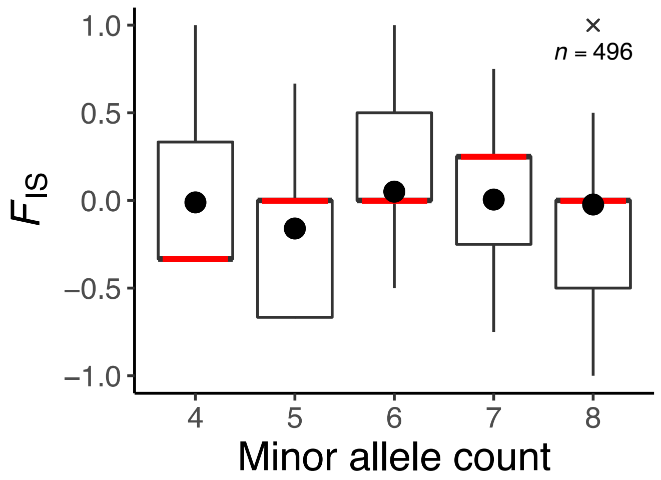

Supplementary Fig. 20 Boxplot showing the distribution of values of the inbreeding coefficient (F_{IS}) for SNPs with different minor allele counts. Only those sites biallelic in individuals L4-L11 with minor allele count ≥ 4 (n = 440,564) were considered. Numbers of sites at minor allele count 4, 5, 6, 7 and 8 equaled to 113,866, 100,501, 92,682, 89,419 and 44,096 respectively. The lower and upper hinges of boxes represent the first and the third quartiles with whiskers extending to the lowest/highest value within 1.5 × IQR from the corresponding hinge, where IQR stands for the interquartile range. Outliers were detected only at minor allele count of 8 and are shown separately with a single cross symbol (all outliers have the same value of F_{IS} equal to 1). The total number of variants corresponding to outliers (496) is indicated beneath the cross. Mean and median values are shown as a black dot and a red horizontal bar respectively. Source data are the same as for Fig. 4a of the main text and are provided as a Source Data file.

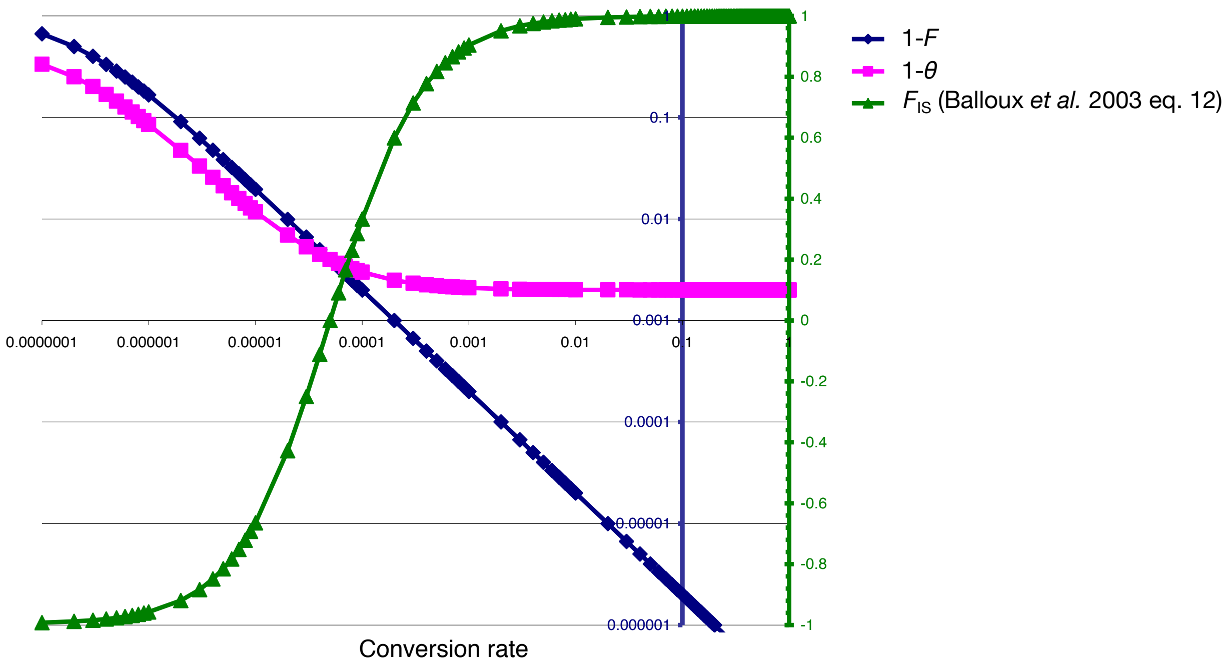

Supplementary Fig. 21 F_{IS}, $1-F$ and $1-\theta$ statistics as the function of gene conversion rate for $N = 10^4$, $u = 10^{-7}$, $4Nu = 0.004$.

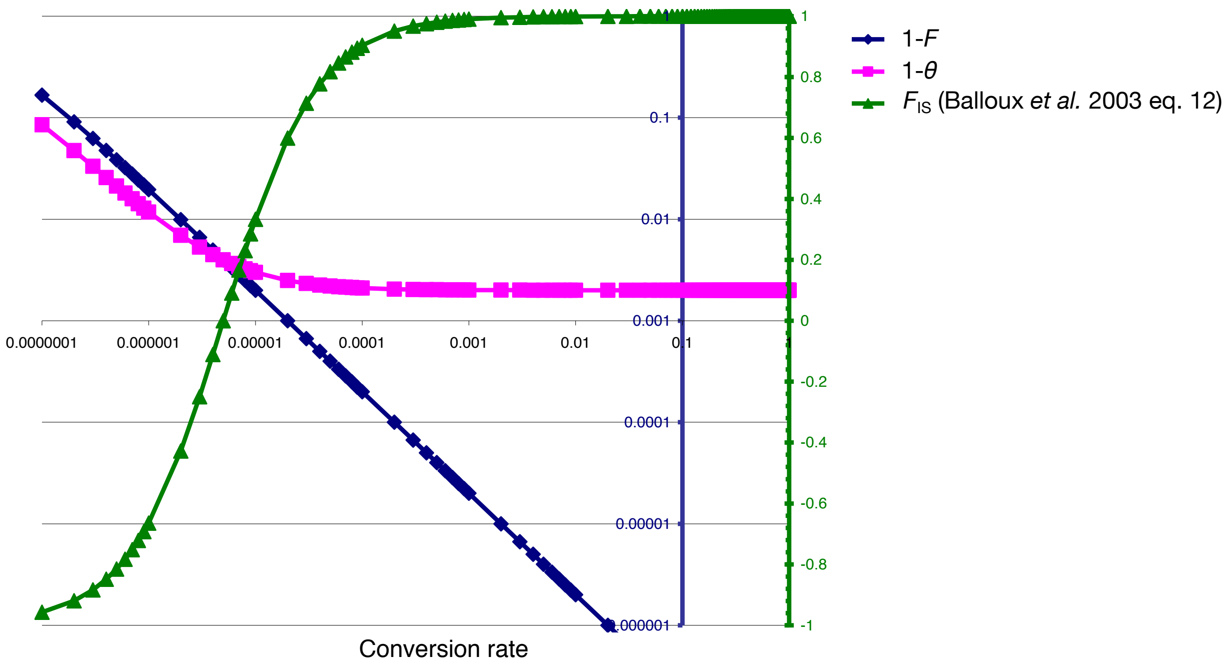

Supplementary Fig. 22 F_{IS}, $1-F$ and $1-\theta$ statistics as the function of gene conversion rate for $N = 10^5$, $u = 10^{-8}$, $4Nu = 0.004$.

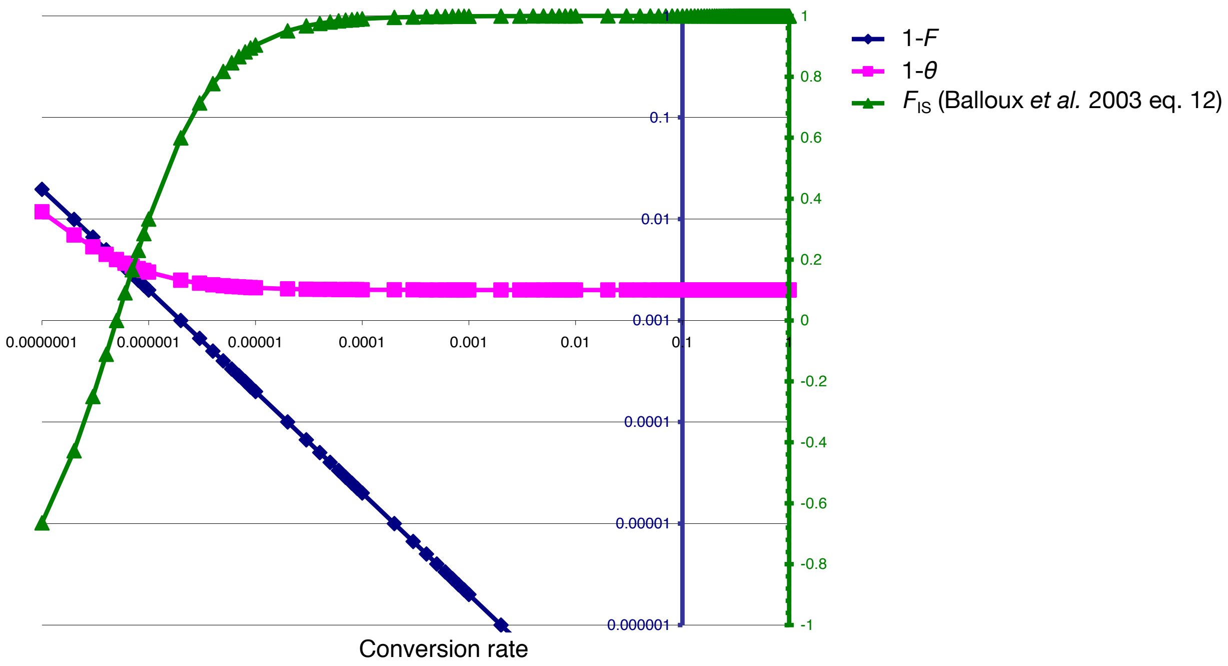

Supplementary Fig. 23 F_{IS}, $1-F$ and $1-\theta$ statistics as the function of gene conversion rate for $N = 10^6$, $u = 10^{-9}$, $4Nu = 0.004$.

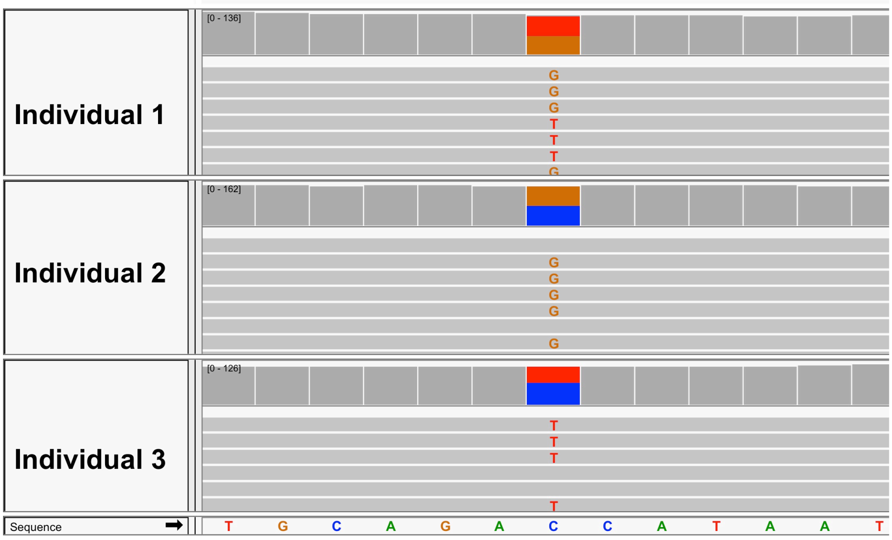

Supplementary Fig. 24 Screenshot from IGV genome browser showing an example of a triallelic site with alleles C, T and G harboring all three heterozygous genotypes among the sequenced individuals. The displayed region is located on contig353 of the L1 diploid assembly.

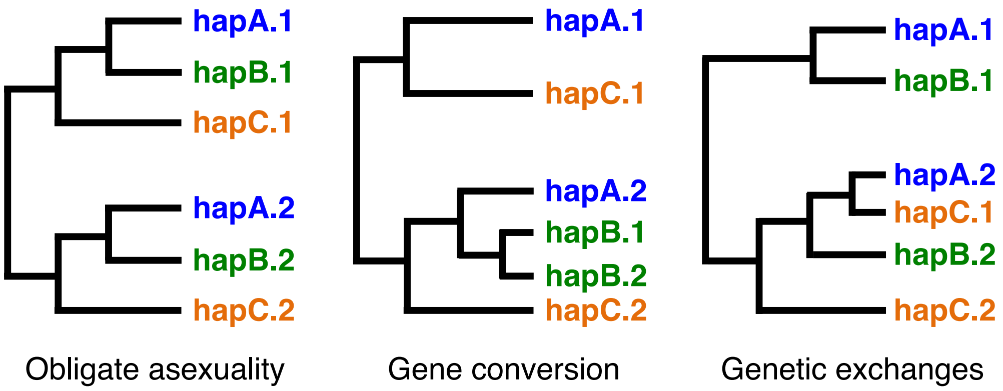

Supplementary Fig. 25 Patterns of relationships between haplotypes of different individuals expected under or consistent with different evolutionary scenarios[38,77]. Haplotypes of three different individuals (designated in Supplementary Discussion as A, B and C) are shown in different colors. Indices 1 and 2 designate the two haplotypes of a single individual.

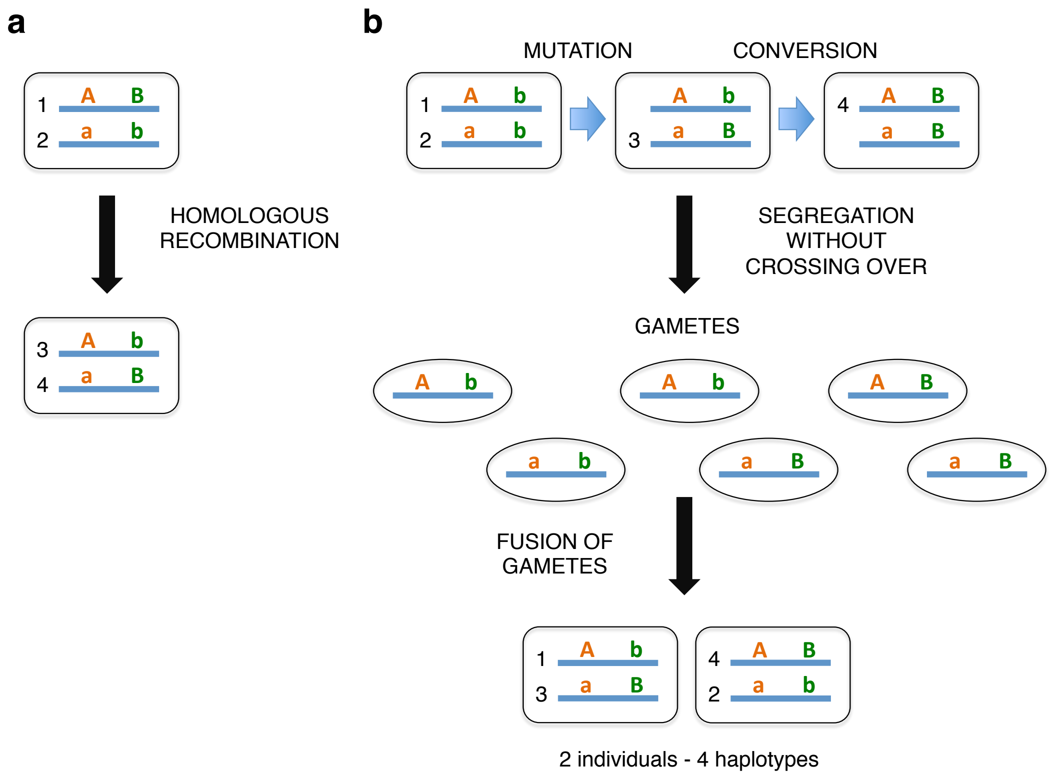

Supplementary Fig. 26 Emergence of four haplotypes for a pair of heterozygous sites in two individuals due to reciprocal recombination or transformation (a) or due to gene conversion in conjunction with sexual reproduction involving *Oenothera*-like meiosis (b).

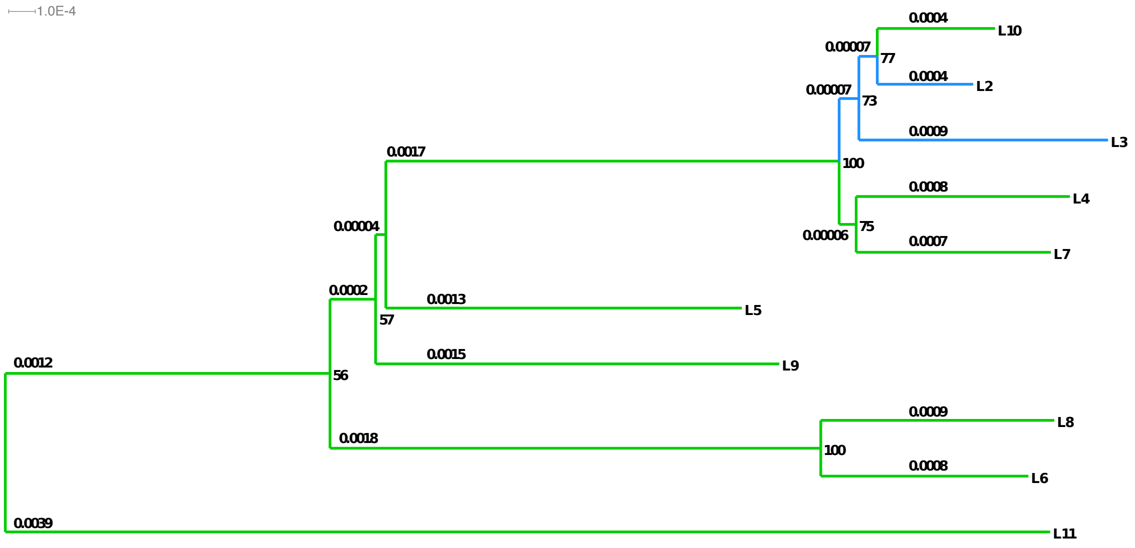

Supplementary Fig. 27 Maximum likelihood mitochondrial phylogeny for individuals L2-L11. The phylogenetic tree was built for the mitochondrial haplotypes of L2-L11 reconstructed based on the genotype calls produced against the L4 mitochondrial contig as reference (total length 14,052 bp; see Supplementary Notes 7 and 8). The unrooted tree was inferred using the maximum likelihood method in RAxML[42] under the GTR+G model with 1,000 bootstrap replicates and manually rooted at the longest branch (L11). The tree was visualized in Dendroscope[43]. Bootstrap support values are shown next to nodes and branch lengths adjacent to branches. Branches leading to individuals of the small and the large cluster are shown in blue and green respectively. Source data are provided as a Source Data file.

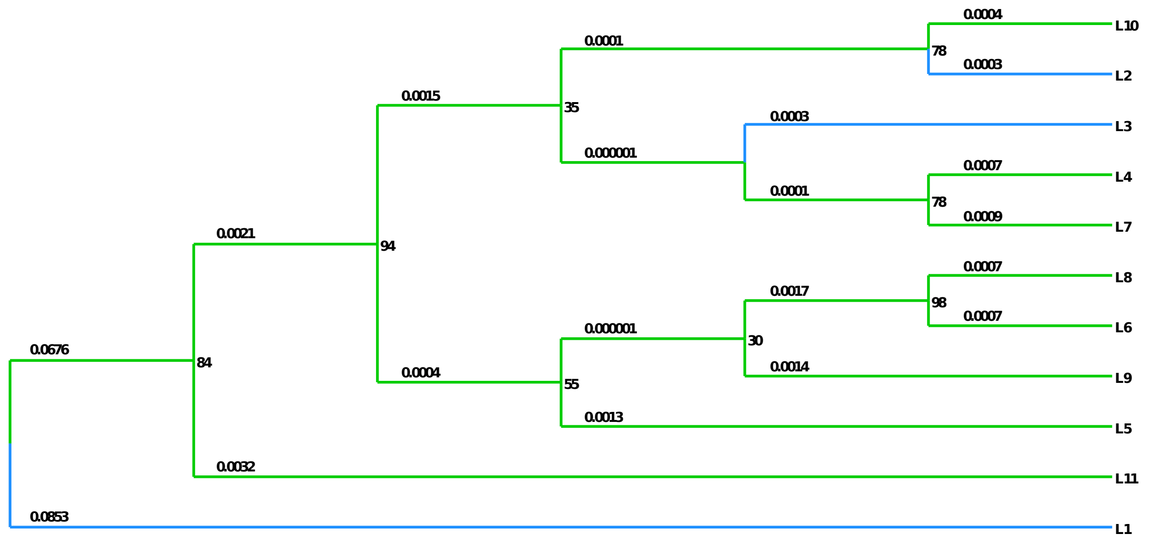

Supplementary Fig. 28 Maximum likelihood mitochondrial phylogeny for individuals L1-L11. The phylogenetic tree was constructed based on the mitochondrial genome region corresponding to the longest L1 mitochondrial contig (contig8072, length = 7,124 bp). The tree was built using the alignment of L1 contig8072 with mitochondrial haplotypes of L2-L11 reconstructed based on the genotype calls produced against the L4 mitochondrial contig as reference (total alignment length = 7,126 bp; see Supplementary Notes 7 and 8). The unrooted tree was inferred using the maximum likelihood method in RAxML[42] under the GTR+G model with 1,000 bootstrap replicates and manually rooted at the longest branch (L1). The tree was visualized in Dendroscope[43]. To highlight topology, branches are not to scale (see Supplementary Fig. 29 for a tree with the scaled branch lengths). Bootstrap support values are shown next to nodes and branch lengths adjacent to branches. Branches leading to individuals of the small and the large cluster are shown in blue and green respectively. Source data are provided as a Source Data file.

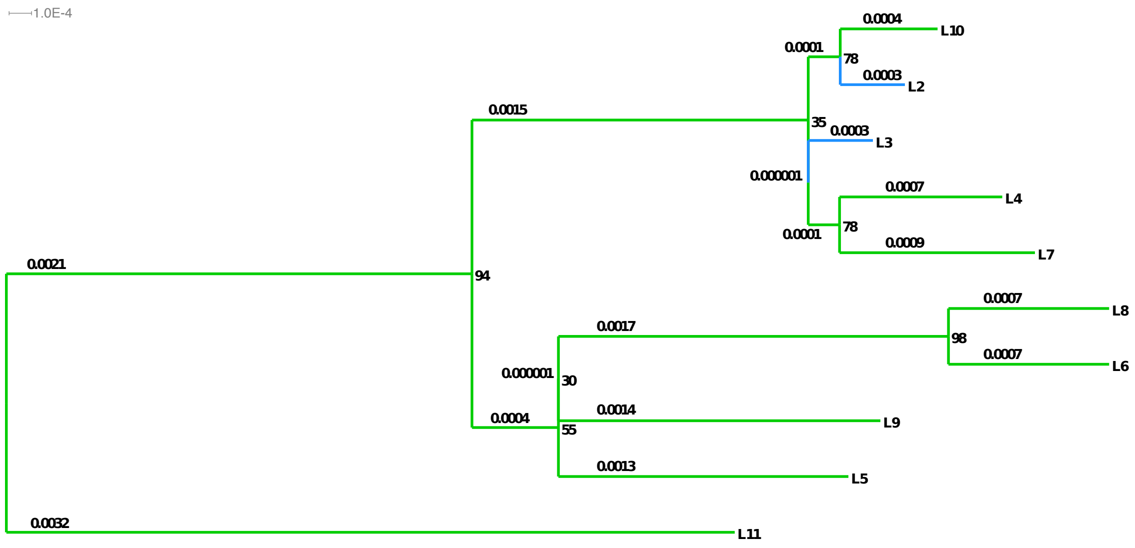

Supplementary Fig. 29 Maximum likelihood mitochondrial phylogeny for individuals L2-L11 with the order of branches determined by rooting with the L1 mitochondrial sequence. This is the subset of the mitochondrial tree for L1-L11 shown in Supplementary Fig. 28 remaining after removing the L1 branch. The phylogenetic tree was constructed based on the mitochondrial genome region corresponding to the longest L1 mitochondrial contig (contig8072, length = 7,124 bp). The tree was built using the alignment of L1 contig8072 with mitochondrial haplotypes of L2-L11 reconstructed based on the genotype calls produced against the L4 mitochondrial contig as reference (total alignment length = 7,126 bp; see Supplementary Notes 7 and 8). The unrooted tree was inferred using the maximum likelihood method in RAxML[42] under the GTR+G model with 1,000 bootstrap replicates and manually rooted at the longest branch (L1) which was then removed from the tree. The tree was visualized in Dendroscope[43]. Bootstrap support values are shown next to nodes and branch lengths adjacent to branches. Branches leading to individuals of the small and the large cluster are shown in blue and green respectively.

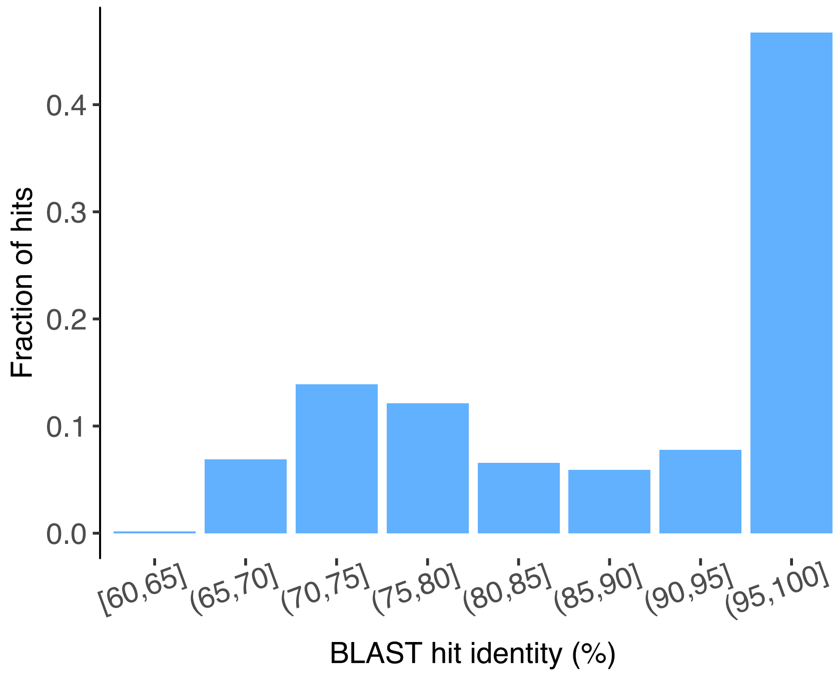

Supplementary Fig. 30 Distribution of nucleotide identities of BLAST hits in L1 genome for L4 alleles from the set of phased segments used to detect incongruence. Both L4 alleles for each out of 434 phased segments harboring at least 15 non-singleton SNPs simultaneously phased in L4-L11 were used to perform the BLAST search against the L1 diploid assembly.

Supplementary Tables

Supplementary Table 1. Sampling locations for the sequenced *A. vaga* individuals L1-L11. Sampling coordinates are approximate. All sequenced individuals collected in the same area were sampled from different trees at least 20 m apart.

Sample/Samples	Sampling coordinates		Sampling location
	Latitude	Longitude	
**L1, L4**	55.752° N	36.513° E	Ruza district, Moscow region, Russia
**L2**	55.752° N	36.512° E	Ruza district, Moscow region, Russia
**L3**	55.74° N	36.52° E	Ruza district, Moscow region, Russia
**L5, L11**	58.166° N	44.403° E	Manturovo district, Kostroma region, Russia
**L6, L7**	55.74° N	36.50° E	Ruza district, Moscow region, Russia
**L8**	55.73° N	36.53° E	Ruza district, Moscow region, Russia
**L9, L10**	55.73° N	36.54° E	Ruza district, Moscow region, Russia

Supplementary Table 2. Estimates of percent identity between the genomes of *A. vaga* individuals L1-L11 and the first published *A. vaga* genome. For each sequenced individual, 1,000,000 Illumina HiSeq reads were randomly drawn and used for the blastn search against the first published *A. vaga* genome assembly[1] (Flot *et al.*, 2013). The maximum number of target sequences to report per query was set to 1 and only a single alignment per hit was considered. Then, the average (or median) nucleotide identity was computed across the read hits to the 2013 *A. vaga* assembly based on reads for which a hit with a minimum alignment length of 70 bp was found.

Individual	Average identity, %	Median identity, %
**L1**	87.35	87.76
**L2**	87.38	87.76
**L3**	87.52	87.76
**L4**	87.43	87.76
**L5**	87.50	87.76
**L6**	87.47	87.76
**L7**	87.49	87.76
**L8**	87.40	87.76
**L9**	87.17	87.36
**L10**	87.45	87.76
**L11**	87.33	87.64

Supplementary Table 3. Assembly statistics for the obtained *A. vaga* reference genome (L1). The statistics were generated with QUAST (v5.0.0)[81] and, unless noted otherwise, are based on contigs (or haploid segments in the case of haploid sub-assembly) with a minimum length of 500 bp.

	**Initial assembly**	**Assembly after removal of contaminant contigs and contigs without match in the published *A. vaga* assembly**	**Haploid sub-assembly**
Number of contigs* (> 0 bp)	78,825	19,202	12,034
Number of contigs (≥ 500 bp)	51,852	19,068	8,999
Number of contigs (≥ 1,000 bp)	25,404	15,929	7,771
Number of contigs (≥ 10,000 bp)	6,530	6,383	2,373
Number of contigs (≥ 100,000 bp)	27	27	1
Total span (> 0 bp)	243,756,304	197,096,676	76,679,421
Total span (≥ 500 bp)	233,752,219	197,031,160	76,098,573
Total span (≥ 1,000 bp)	215,896,885	194,878,077	75,214,106
Total span (≥ 10,000 bp)	158,370,356	154,705,106	54,102,273
Total span (≥ 100,000 bp)	3,101,138	3,101,138	103,250
Number of contigs	51,852	19,068	8,999
Largest contig	167,368	167,368	103,250
GC (%)	33.47	29.92	29.52
N50	18,125	22,073	18,007
N75	6,789	11,331	8,568
L50	3,473	2,620	1,182
L75	8,572	5,731	2,695
Number of N's per 100 kbp	0	0	0

*Contigs or haploid segments in the case of haploid sub-assembly.

Supplementary Table 4. Annotation metrics for the set of gene predictions generated for the *A. vaga* L1 diploid genome assembly. For comparison with the first published *A. vaga* genome[1] (Flot *et al.*, 2013), see Table 1 from the paper by Nowell *et al.*, 2018, reporting reannotation of the 2013 *A. vaga* assembly[2].

Mean (median) CDS length, bp	1,278.3 (990)
Mean (median) intron length, bp	93.3 (55)
Mean number of introns per gene	4.1
Transcript GC, %	31.9

Supplementary Table 5. Numbers of genomic sites in the raw SNP datasets, and numbers of sites retained after applying various quality filtering steps. Numbers of sites included in the final filtered datasets (stringent SNP datasets I and II) are shown in bold.

	**SNP dataset**	
	**Dataset I (variable sites only)**	**Dataset II (variable and invariant sites)**
**Prior to filtration**	3,318,352	76,306,143
**Filter:**		
**SNPs within 10 bp of an indel removed**	2,979,193	75,968,700
**Sites with missing genotypes and sites with QUAL < 50 removed**	2,655,917	49,222,726
**Sites on haploid segments shorter than 1,000 bp removed**	2,634,341	48,730,324
**Sites residing within repetitive regions removed**	2,596,490	47,531,499
**Sites covered by < 10 reads in any of the samples removed**	2,409,323	44,541,675
**Sites with extremely high or low coverage removed**	2,391,710	43,924,505
**Sites within the windows outliers for SNP density removed**	**2,282,099**	**42,850,155**

Supplementary Table 6. Validation of SNP calls included in the SNP dataset I with GATK HaplotypeCaller. The table presents statistics on the numbers of SNP calls obtained with SAMtools/BCFtools and identically recovered with GATK HaplotypeCaller for the raw and stringently filtered SNP dataset I.
*Out of 3,318,352 SNP sites of the raw SNP dataset I, for each individual, only those sites with called (non-missing) genotypes for the given individual are considered.
In the stringent SNP dataset I, the same number of sites is assessed for all individuals, as sites with missing genotypes in any of the individuals were removed from this dataset.

	raw SNP dataset I (SAMtools)			stringent SNP dataset I (SAMtools)		
	Total assessed SNPs*	Identically called with GATK		Total assessed SNPs	Identically called with GATK	
**Individual**		**Total**	**%**		**Total**	**%**
**L1**	3,269,617	2,848,378	87.1%	2,282,099	2,148,593	94.1%
**L2**	3,183,086	2,845,505	89.4%		2,171,466	95.2%
**L3**	3,202,457	2,858,438	89.3%		2,171,573	95.2%
**L4**	3,192,537	2,854,936	89.4%		2,172,377	95.2%
**L5**	3,301,107	2,864,509	86.8%		2,136,972	93.6%
**L6**	3,216,248	2,868,368	89.2%		2,172,356	95.2%
**L7**	3,203,839	2,860,042	89.3%		2,171,935	95.2%
**L8**	3,205,121	2,861,219	89.3%		2,171,595	95.2%
**L9**	3,275,387	2,845,563	86.9%		2,145,689	94.0%
**L10**	3,204,304	2,859,660	89.2%		2,171,224	95.1%
**L11**	3,219,783	2,870,057	89.1%		2,171,381	95.1%

Supplementary Table 7. Pairwise genotypic distances between the sequenced *A. vaga* individuals. Genotypic distances were computed based on the sites of the haploid sub-assembly simultaneously called in all sequenced individuals L1-L11. Only monomorphic and biallelic sites from the SNP dataset III were used in the analysis (n = 58,118,767). For a pair of *A. vaga* individuals, the genotypic distance was calculated in the following way: the distance at each assessed genomic site was computed as the difference in the number of non-reference variants (0, 1 or 2), then the resulting values were summed over all analyzed sites and divided by $2n$. Distances between the individuals belonging to different clusters are highlighted in orange, and distances within the small and the large cluster are shown in blue and green respectively.

	L1	L2	L3	L4	L5	L6	L7	L8	L9	L10	L11
**L1**	0%										
**L2**	0.657%	0%									
**L3**	0.650%	0.675%	0%								
**L4**	1.212%	1.217%	1.221%	0%							
**L5**	1.215%	1.217%	1.222%	0.529%	0%						
**L6**	1.214%	1.217%	1.220%	0.538%	0.528%	0%					
**L7**	1.213%	1.219%	1.220%	0.538%	0.530%	0.526%	0%				
**L8**	1.215%	1.219%	1.222%	0.538%	0.529%	0.511%	0.521%	0%			
**L9**	1.217%	1.222%	1.221%	0.535%	0.533%	0.530%	0.528%	0.531%	0%		
**L10**	1.229%	1.232%	1.238%	0.542%	0.547%	0.552%	0.553%	0.555%	0.556%	0%	
**L11**	1.214%	1.215%	1.220%	0.531%	0.532%	0.536%	0.537%	0.535%	0.536%	0.544%	0%

Supplementary Table 8. Maximum likelihood estimates of $4N_e\mu$ and sequencing error rate for 11 sequenced *A. vaga* individuals. Estimates of $4N_e\mu$ and sequencing error rate were obtained independently for each individual using the maximum likelihood approach implemented in the program mlRho. Estimates are based on sites covered by no less than 20 reads in the considered individual.
Individuals belonging to the small and the large cluster are highlighted in blue and green respectively.

Individual	Number of sites (≥20X)	$4N_e\mu$	95% CI for $4N_e\mu$		Sequencing error rate
**L1**	71,024,512	2.21E-02	2.21E-02	2.21E-02	4.40E-04
**L2**	72,189,265	2.26E-02	2.26E-02	2.27E-02	5.44E-04
**L3**	70,758,489	2.23E-02	2.23E-02	2.24E-02	4.19E-04
**L4**	67,943,878	8.15E-03	8.12E-03	8.17E-03	8.30E-04
**L5**	70,805,730	9.40E-03	9.37E-03	9.42E-03	4.53E-04
**L6**	69,056,642	8.63E-03	8.61E-03	8.65E-03	5.17E-04
**L7**	69,092,259	8.72E-03	8.70E-03	8.75E-03	4.81E-04
**L8**	69,160,624	8.77E-03	8.74E-03	8.79E-03	5.58E-04
**L9**	69,951,042	8.80E-03	8.78E-03	8.82E-03	5.86E-04
**L10**	68,708,316	7.22E-03	7.20E-03	7.24E-03	4.79E-04
**L11**	69,943,870	8.93E-03	8.91E-03	8.95E-03	5.48E-04

Supplementary Table 9. Statistics on the span of phased blocks assembled with HapCUT2 and included in the phased dataset 1. Homozygous SNPs were appended to phased blocks based on the block assignment of closest flanking heterozygous SNPs.

Individual	Median number of heterozygous SNPs per phased block	Average number of heterozygous SNPs per phased block	Median number of SNPs per phased block (homozygous SNPs appended)	Average number of SNPs per phased block (homozygous SNPs appended)	Median span of phased blocks (bp)	Average span of phased blocks (bp)
L1	26	47.9	50	90.5	1,720	3,196
L2	25	45.4	49	86.4	1,691	3,087
L3	32	59.8	65	114.7	2,205	4,143
L4	5	7.9	15	24.3	461	697
L5	5	8.5	17	27.5	541	813
L6	6	9.9	21	34.4	694	1,060
L7	6	9.5	19	32.2	639	980
L8	5	8.9	17	29.1	562	865
L9	6	9.8	20	33.5	680	1,027
L10	5	9.0	18	30.1	600	917
L11	6	9.1	18	30.1	596	906

Supplementary Table 10. Whole-genome numbers of phased SNPs belonging to phased blocks assembled with HapCUT2 and included in the phased dataset 1. Total numbers of phased SNPs were determined as the numbers of SNPs belonging to any phased block encompassing at least 2 heterozygous SNPs irrespective of its length. Homozygous SNPs were appended to phased blocks based on the block assignment of closest flanking heterozygous SNPs.

Individual	Total number of phased blocks	Total number of SNPs subjected to phasing	Total number of phased SNPs			Total number of unphased SNPs		
			SNPs in phased blocks	Heterozygous phased SNPs	Homozygous phased SNPs	Unphased SNPs	Heterozygous unphased SNPs	Homozygous unphased SNPs
L1	15,281	1,774,991	1,382,306	731,887	650,419	392,685	164,023	228,662
L2	11,503	1,774,991	994,252	522,769	471,483	780,739	359,381	421,358
L3	9,337	1,774,991	1,070,549	558,405	512,144	704,442	321,524	382,918
L4	31,777	1,774,991	771,705	251,366	520,339	1,003,286	33,706	969,580
L5	30,092	1,774,991	826,656	255,968	570,688	948,335	37,929	910,406
L6	26,488	1,774,991	911,315	262,482	648,833	863,676	31,445	832,231
L7	27,856	1,774,991	896,656	263,385	633,271	878,335	29,862	848,473
L8	29,752	1,774,991	865,236	264,824	600,412	909,755	30,324	879,431
L9	26,933	1,774,991	901,716	263,112	638,604	873,275	31,158	842,117
L10	24,135	1,774,991	727,669	217,007	510,662	1,047,322	23,005	1,024,317
L11	28,312	1,774,991	852,563	257,000	595,563	922,428	32,589	889,839

Supplementary Table 11. Statistics on F_{IS} values for individuals L4-L11. F_{IS} values were computed for biallelic SNPs from the stringent SNP dataset II common (MAC ≥ 4) among the individuals of the large cluster, L4-L11.

SNP Dataset	Total number of biallelic SNPs	Total number of common (MAC≥4) biallelic SNPs	F_{IS}				
			Mean	Median	Standard deviation	Q1	Q3
Whole genome, large cluster, L4-L11	1,106,582	440,564	−0.03	0	0.39	−0.33	0.25
Allelic regions, large cluster, L4-L11	285,043	112,236	−0.03	0	0.38	−0.33	0.25
Allelic genes, large cluster, L4-L11	194,384	77,543	−0.04	0	0.38	−0.33	0.25

Supplementary Table 12. Per individual numbers of sites with a unique heterozygous genotype private to the given individual among triallelic sites carrying all three heterozygous genotypes. Only those triallelic sites harboring all three heterozygous genotypes among L4-L11 for which exactly one private heterozygous genotype exists (n = 607) were considered.

Individual	Number of sites with a unique heterozygous genotype private to the given individual among the sites harboring three heterozygotes
L4	77
L5	77
L6	88
L7	76
L8	79
L9	74
L10	72
L11	64

Supplementary Table 13. Per individual numbers of unique single nucleotide mitochondrial variants private to an individual (identified using L4 haplotype as a reference sequence). For each individual L2-L11, the table shows the number of sites at which this individual is different from the rest of individuals L2-L11. The presented numbers are based on sites simultaneously called in all individuals L2-L11 (n = 13,764) using the L4 mitochondrial contig as reference.

Individual	Total assessed mitochondrial sites	Sites with a single nucleotide variant carried only by this individual
L2	13,764	5
L3	13,764	10
L4	13,764	8
L5	13,764	17
L6	13,764	9
L7	13,764	10
L8	13,764	12
L9	13,764	18
L10	13,764	6
L11	13,764	57

Supplementary Table 14. Per individual numbers of unique single nucleotide mitochondrial variants private to an individual (identified using L3 haplotype as a reference sequence). For each individual L2-L11, the table shows the number of sites at which this individual is different from the rest of individuals L2-L11. The presented numbers are based on sites simultaneously called in all individuals L2-L11 (n = 13,640) using the L3 mitochondrial contig as reference.

Individual	Total assessed mitochondrial sites	Sites with a single nucleotide variant carried only by this individual
L2	13,640	5
L3	13,640	10
L4	13,640	8
L5	13,640	18
L6	13,640	10
L7	13,640	10
L8	13,640	12
L9	13,640	17
L10	13,640	6
L11	13,640	56

Supplementary Table 15. Pairwise distances between mitochondrial haplotypes of *A. vaga* individuals L2-L11 inferred using L4 haplotype as a reference sequence. For each pair of individuals L2-L11, the table shows the absolute number of single nucleotide differences between mitochondrial haplotypes of these two individuals. Numbers of nucleotide differences were inferred by calling single nucleotide variants in individuals L2-L11 against the L4 mitochondrial contig (length = 14,052 bp). Presented numbers are based on sites simultaneously called in all individuals L2-L11 (*n* = 13,764).

	**L2**	**L3**	**L4**	**L5**	**L6**	**L7**	**L8**	**L9**	**L10**
**L3**	19								
**L4**	19	26							
**L5**	48	53	53						
**L6**	69	76	70	57					
**L7**	18	25	21	52	73				
**L8**	70	77	71	58	23	74			
**L9**	50	57	55	40	59	54	60		
**L10**	11	20	20	49	70	19	71	51	
**L11**	94	99	99	90	99	100	104	88	95

Supplementary Table 16. Pairwise distances between mitochondrial haplotypes of *A. vaga* individuals L2-L11 inferred using L3 haplotype as a reference sequence. For each pair of individuals L2-L11, the table shows the absolute number of single nucleotide differences between mitochondrial haplotypes of these two individuals. Numbers of nucleotide differences were inferred by calling single nucleotide variants in individuals L2-L11 against the L3 mitochondrial contig (length = 13,781 bp). Presented numbers are based on sites simultaneously called in all individuals L2-L11 (*n* = 13,640).

	**L2**	**L3**	**L4**	**L5**	**L6**	**L7**	**L8**	**L9**	**L10**
**L3**	20								
**L4**	19	27							
**L5**	48	54	53						
**L6**	69	77	70	59					
**L7**	18	26	21	52	73				
**L8**	69	77	70	59	24	73			
**L9**	48	56	53	40	59	52	59		
**L10**	11	21	20	49	70	19	70	49	
**L11**	93	97	98	91	100	99	104	87	94

Supplementary Table 17. Per individual numbers of mitochondrial sites with reads supporting presence of two nucleotide variants in a single individual as identified with Mutect2. For individuals L2-L11, Mutect2 variant calls were generated relative to the L4 mitochondrial contig, and for L1 relative to the L1 mitochondrial contigs. Only those heterogeneous mitochondrial sites with minor allele fraction variant supported by no fewer than three reads were considered. For each individual, the table shows the total number of such sites and numbers of heterogeneous sites where the minor allele fraction variant is supported by ≥ 1% and ≥ 10% of reads aligned at the corresponding position.

Individual	Mitochondrial sites with two variants detected in Illumina reads from a single individual		
	Total	Minor allele supported by ≥ 1% of reads	Minor allele supported by ≥ 10% of reads
**L1**	2	0	0
**L2**	6	2	0
**L3**	2	1	0
**L4**	8	1	0
**L5**	7	3	1
**L6**	11	2	1
**L7**	1	0	0
**L8**	5	1	0
**L9**	7	2	2
**L10**	1	1	0
**L11**	7	0	0

Supplementary Table 18. Percentage and numbers of reads supporting minor allele fraction variants for heterogeneous mitochondrial sites identified in L1-L11 with Mutect2. For individuals L2-L11, Mutect2 variant calls were generated relative to the L4 mitochondrial contig and for L1 relative to the L1 mitochondrial contigs. Only those heterogeneous mitochondrial sites with minor allele fraction variant supported by no fewer than three reads aligned at the corresponding position were considered. In the case of L7 and L10 for which only a single such site was found (denoted with an asterisk), the exact numbers/percentage of reads at such site are shown.

Individual	Mitochondrial sites with two variants detected in Illumina reads from a single individual						
	Total	Average per site coverage	Median per site coverage	Average number of reads supporting the minor allele fraction variant	Median number of reads supporting the minor allele fraction variant	Average percentage of reads supporting the minor allele fraction variant	Median percentage of reads supporting the minor allele fraction variant
**L1**	2	634.5	634.5	4.5	4.5	0.7%	0.7%
**L2**	6	736.5	760.5	16.2	4.5	2.4%	0.7%
**L3**	2	1,727.0	1,727	21.5	21.5	1.3%	1.3%
**L4**	8	1,046.0	1,157	4.8	3.5	0.5%	0.3%
**L5**	7	1,578.9	1,409	48.1	11	5.8%	0.8%
**L6**	11	2,121.3	2,448	26.4	4	1.8%	0.2%
**L7***	1	1,223	NA	3	NA	0.2%	NA
**L8**	5	1,718.8	1,559	6.0	5	0.5%	0.3%
**L9**	7	1,112.3	1,167	61.7	4	5.8%	0.3%
**L10***	1	100	NA	7	NA	7.0%	NA
**L11**	7	2,314.1	2,375	5.9	3	0.2%	0.2%

Supplementary Table 19. Patterns of incongruence observed for different phased segments of the *A. vaga* genome. For each pair of individuals L4-L11, we computed the number of phased segments (out of 303 analyzed segments from the set A) when there existed a third individual most similar to the first individual from the pair with respect to one haplotype and most similar to the second individual from the pair with respect to the other haplotype (only cases with bootstrap support values ≥ 70% were considered, see Methods and Table 1 of the main text). Some segments exhibited the above-described pattern for more than one pair of individuals.

	L4	L5	L6	L7	L8	L9	L10
**L5**	1						
**L6**	0	2					
**L7**	3	1	3				
**L8**	2	4	2	3			
**L9**	2	5	3	1	1		
**L10**	3	2	2	2	1	2	
**L11**	2	2	7	4	3	1	1

Supplementary Table 20. Incongruent groupings of the two haplotypes in individuals L4-L11 for the phased segments from the set B.
This table is analogous to Table 1 of the main text, but statistics are based on phased segments from the set B. Segments included in the set B were additionally filtered for SNPs possibly affected by inaccuracies in SNP identification including those potentially introduced by index hopping and heterozygote undercalling (see Methods). In total, out of the 190 analyzed phased genomic segments from the set B, 25 exhibited incongruent groupings of the two haplotypes at least in one individual with strong bootstrap support (≥70%).

	Number of			
Individual	Analyzed phased segments	Incongruent phased segments	Different patterns of incongruence	Observed patterns of incongruence
**L4**	190	2	2	L5-L11 (1), L6-L11 (1)
**L5**	190	2	2	L4-L10 (1), L6-L11 (1)
**L6**	190	6	5	L5-L8 (2), L5-L9 (1), L8-L10 (1), L8-L11 (1), L9-L11 (1)
**L7**	190	3	3	L4-L8 (1), L5-L9 (1), L10-L11 (1)
**L8**	190	8	6	L4-L5 (1), L5-L7 (1), L5-L10 (1), L6-L9 (2), L6-L10 (1), L6-L11 (2)
**L9**	190	2	2	L5-L10 (1), L6-L8 (1)
**L10**	190	6	5	L4-L11 (1), L6-L7 (1), L7-L8 (1), L7-L11 (2), L8-L9 (1)
**L11**	190	4	4	L4-L7 (1), L6-L10 (1), L7-L8 (1), L7-L9 (1)

Supplementary Table 21. Incongruent groupings of the two haplotypes in individuals L1-L11 for the phased segments from the set C.
This table is analogous to Table 1 of the main text, but statistics are based on segments from the set C phased in all individuals L1-L11 and carrying at least 15 non-singleton SNPs among L1-L11. Segments were additionally filtered for SNPs possibly affected by inaccuracies in SNP identification including those potentially introduced by index hopping and heterozygote undercalling (see Methods). In total, out of the 152 analyzed phased genomic segments from the set C, 16 exhibited incongruent groupings of the two haplotypes at least in one individual with strong bootstrap support (≥70%).

Individual	Number of			Observed patterns of incongruence
	Analyzed phased segments	Incongruent phased segments	Different patterns of incongruence	
**L1**	152	5	4	L2-L5 (1), L3-L4 (1), L3-L5 (1), L3-L10 (2)
**L2**	152	2	2	L3-L8 (1), L3-L11 (1)
**L3**	152	4	2	L1-L4 (2), L2-L9 (2)
**L4**	152	1	1	L5-L11 (1)
**L5**	152	0	0	
**L6**	152	2	2	L1-L8 (1), L5-L8 (1)
**L7**	152	0	0	
**L8**	152	2	2	L6-L9 (1), L6-L11 (1)
**L9**	152	1	1	L3-L6 (1)
**L10**	152	1	1	L3-L4 (1)
**L11**	152	2	2	L1-L5 (1), L7-L9 (1)

Supplementary Table 22. Accession numbers for Illumina HiSeq reads deposited in the NCBI short read archive for each sequenced *A. vaga* individual.

**Individual**	**SRA accession numbers**
**L1**	SRR8134454
**L2**	SRR8134453
**L3**	SRR8134452
**L4**	SRR8135136
**L5**	SRR8135135
**L6**	SRR8135137, SRR8135138
**L7**	SRR8135133, SRR8135134
**L8**	SRR8136358, SRR8136359
**L9**	SRR8136356
**L10**	SRR8136357, SRR8136361
**L11**	SRR8136360, SRR8136362

Supplementary References

1. Flot, J.-F. *et al.* Genomic evidence for ameiotic evolution in the bdelloid rotifer Adineta vaga. *Nature* **500**, 453–457 (2013).
2. Nowell, R. W. *et al.* Comparative genomics of bdelloid rotifers: Insights from desiccating and nondesiccating species. *PLOS Biol.* **16**, e2004830 (2018).
3. Simão, F. A., Waterhouse, R. M., Ioannidis, P., Kriventseva, E. V. & Zdobnov, E. M. BUSCO: assessing genome assembly and annotation completeness with single-copy orthologs. *Bioinforma. Oxf. Engl.* **31**, 3210–3212 (2015).
4. Vakhrusheva, O. *et al.* Assembly and annotation of the Adineta vaga L1 genome. Figshare https://doi.org/10.6084/m9.figshare.11620518.v2 (2020).
5. Altschul, S. F., Gish, W., Miller, W., Myers, E. W. & Lipman, D. J. Basic local alignment search tool. *J. Mol. Biol.* **215**, 403–410 (1990).
6. Blanchette, M. *et al.* Aligning multiple genomic sequences with the threaded blockset aligner. *Genome Res.* **14**, 708–715 (2004).
7. Schwartz, S. *et al.* Human-mouse alignments with BLASTZ. *Genome Res.* **13**, 103–107 (2003).
8. Stanke, M. & Waack, S. Gene prediction with a hidden Markov model and a new intron submodel. *Bioinforma. Oxf. Engl.* **19 Suppl 2**, ii215-225 (2003).
9. Lomsadze, A., Ter-Hovhannisyan, V., Chernoff, Y. O. & Borodovsky, M. Gene identification in novel eukaryotic genomes by self-training algorithm. *Nucleic Acids Res.* **33**, 6494–6506 (2005).
10. Dobin, A. *et al.* STAR: ultrafast universal RNA-seq aligner. *Bioinforma. Oxf. Engl.* **29**, 15–21 (2013).
11. Wang, Y. *et al.* MCScanX: a toolkit for detection and evolutionary analysis of gene synteny and collinearity. *Nucleic Acids Res.* **40**, e49 (2012).

12. Langmead, B. & Salzberg, S. L. Fast gapped-read alignment with Bowtie 2. *Nat. Methods* **9**, 357–359 (2012).

13. Li, H. *et al.* The Sequence Alignment/Map format and SAMtools. *Bioinforma. Oxf. Engl.* **25**, 2078–2079 (2009).

14. Danecek, P. *et al.* The variant call format and VCFtools. *Bioinformatics* **27**, 2156–2158 (2011).

15. Quinlan, A. R. BEDTools: The Swiss-Army Tool for Genome Feature Analysis. *Curr. Protoc. Bioinforma.* **47**, 11.12.1-34 (2014).

16. Cingolani, P. *et al.* Using Drosophila melanogaster as a Model for Genotoxic Chemical Mutational Studies with a New Program, SnpSift. *Front. Genet.* **3**, 35 (2012).

17. Vakhrusheva, O. *et al.* SNPs identified in Adineta vaga individuals L1-L11. Figshare https://doi.org/10.6084/m9.figshare.11625780.v2 (2020).

18. McKenna, A. *et al.* The Genome Analysis Toolkit: A MapReduce framework for analyzing next-generation DNA sequencing data. *Genome Res.* **20**, 1297–1303 (2010).

19. O'Rawe, J. *et al.* Low concordance of multiple variant-calling pipelines: practical implications for exome and genome sequencing. *Genome Med.* **5**, 28 (2013).

20. Hwang, S., Kim, E., Lee, I. & Marcotte, E. M. Systematic comparison of variant calling pipelines using gold standard personal exome variants. *Sci. Rep.* **5**, 17875 (2015).

21. Purcell, S. *et al.* PLINK: a tool set for whole-genome association and population-based linkage analyses. *Am. J. Hum. Genet.* **81**, 559–575 (2007).

22. Kamvar, Z. N., Tabima, J. F. & Grünwald, N. J. Poppr: an R package for genetic analysis of populations with clonal, partially clonal, and/or sexual reproduction. *PeerJ* **2**, e281 (2014).
23. Kamvar, Z. N., Brooks, J. C. & Grünwald, N. J. Novel R tools for analysis of genome-wide population genetic data with emphasis on clonality. *Front. Genet.* **6**, (2015).
24. Edge, P., Bafna, V. & Bansal, V. HapCUT2: robust and accurate haplotype assembly for diverse sequencing technologies. *Genome Res.* **27**, 801–812 (2017).
25. Kebschull, J. M. & Zador, A. M. Sources of PCR-induced distortions in high-throughput sequencing data sets. *Nucleic Acids Res.* **43**, e143 (2015).
26. Auton, A. & Marcketta, A. VCFtools. A set of tools written in Perl and C++ for working with VCF files. https://vcftools.github.io/man_latest.html (2015).
27. Lynch, M. Estimation of nucleotide diversity, disequilibrium coefficients, and mutation rates from high-coverage genome-sequencing projects. *Mol. Biol. Evol.* **25**, 2409–2419 (2008).
28. Haubold, B., Pfaffelhuber, P. & Lynch, M. mlRho - a program for estimating the population mutation and recombination rates from shotgun-sequenced diploid genomes. *Mol. Ecol.* **19 Suppl 1**, 277–284 (2010).
29. Lynch, M. *et al.* Genome-Wide Linkage-Disequilibrium Profiles from Single Individuals. *Genetics* **198**, 269–281 (2014).
30. Lynch, M. *et al.* Population Genomics of Daphnia pulex. *Genetics* **206**, 315–332 (2017).
31. Awadalla, P., Eyre-Walker, A. & Smith, J. M. Linkage Disequilibrium and Recombination in Hominid Mitochondrial DNA. *Science* **286**, 2524–2525 (1999).

32. Meunier, J. & Eyre-Walker, A. The Correlation Between Linkage Disequilibrium and Distance: Implications for Recombination in Hominid Mitochondria. *Mol. Biol. Evol.* **18**, 2132–2135 (2001).
33. McVean, G., Awadalla, P. & Fearnhead, P. A coalescent-based method for detecting and estimating recombination from gene sequences. *Genetics* **160**, 1231–1241 (2002).
34. Bruen, T. C., Philippe, H. & Bryant, D. A simple and robust statistical test for detecting the presence of recombination. *Genetics* **172**, 2665–2681 (2006).
35. Huson, D. H. & Bryant, D. Application of Phylogenetic Networks in Evolutionary Studies. *Mol. Biol. Evol.* **23**, 254–267 (2006).
36. Bankevich, A. *et al.* SPAdes: a new genome assembly algorithm and its applications to single-cell sequencing. *J. Comput. Biol.* **19**, 455–477 (2012).
37. Fontaneto, D. *et al.* Cryptic diversity in the genus Adineta Hudson & Gosse, 1886 (Rotifera: Bdelloidea: Adinetidae): A DNA taxonomy approach. *Hydrobiologia* **662**, 27–33 (2011).
38. Signorovitch, A., Hur, J., Gladyshev, E. & Meselson, M. Allele Sharing and Evidence for Sexuality in a Mitochondrial Clade of Bdelloid Rotifers. *Genetics* **200**, 581–590 (2015).
39. Lasek-Nesselquist, E. A Mitogenomic Re-Evaluation of the Bdelloid Phylogeny and Relationships among the Syndermata. *PLOS ONE* **7**, e43554 (2012).
40. Kans, J. *Entrez Direct: E-utilities on the UNIX Command Line*. (National Center for Biotechnology Information (US), 2019).
41. Edgar, R. C. MUSCLE: a multiple sequence alignment method with reduced time and space complexity. *BMC Bioinformatics* **5**, 113 (2004).

42. Stamatakis, A. RAxML version 8: a tool for phylogenetic analysis and post-analysis of large phylogenies. *Bioinformatics* **30**, 1312–1313 (2014).
43. Huson, D. H. *et al.* Dendroscope: An interactive viewer for large phylogenetic trees. *BMC Bioinformatics* **8**, 460 (2007).
44. Li, H. Minimap2: pairwise alignment for nucleotide sequences. *Bioinformatics* **34**, 3094–3100 (2018).
45. Patterson, M. *et al.* WhatsHap: Weighted Haplotype Assembly for Future-Generation Sequencing Reads. *J. Comput. Biol. J. Comput. Mol. Cell Biol.* **22**, 498–509 (2015).
46. Hudson, R. R. & Kaplan, N. L. Statistical properties of the number of recombination events in the history of a sample of DNA sequences. *Genetics* **111**, 147–164 (1985).
47. Wijnker, E. *et al.* The genomic landscape of meiotic crossovers and gene conversions in Arabidopsis thaliana. *eLife* **2**, e01426 (2013).
48. Duret, L. & Galtier, N. Biased gene conversion and the evolution of mammalian genomic landscapes. *Annu. Rev. Genomics Hum. Genet.* **10**, 285–311 (2009).
49. Robinson, M. C., Stone, E. A. & Singh, N. D. Population genomic analysis reveals no evidence for GC-biased gene conversion in Drosophila melanogaster. *Mol. Biol. Evol.* **31**, 425–433 (2014).
50. Balloux, F., Lehmann, L. & de Meeûs, T. The population genetics of clonal and partially clonal diploids. *Genetics* **164**, 1635–1644 (2003).
51. Wright, S. Evolution in Mendelian Populations. *Genetics* **16**, 97–159 (1931).
52. Reichel, K., Masson, J.-P., Malrieu, F., Arnaud-Haond, S. & Stoeckel, S. Rare sex or out of reach equilibrium? The dynamics of F_{IS} in partially clonal organisms. *BMC Genet.* **17**, 1–16 (2016).

53. Benson, G. Tandem repeats finder: a program to analyze DNA sequences. *Nucleic Acids Res.* **27**, 573–580 (1999).
54. Vakhrusheva, O. *et al.* Sequences of mitochondrial contigs for Adineta vaga individuals L1-L11. Figshare https://doi.org/10.6084/m9.figshare.12008790.v2 (2020).
55. Allio, R., Donega, S., Galtier, N. & Nabholz, B. Large Variation in the Ratio of Mitochondrial to Nuclear Mutation Rate across Animals: Implications for Genetic Diversity and the Use of Mitochondrial DNA as a Molecular Marker. *Mol. Biol. Evol.* **34**, 2762–2772 (2017).
56. Vakhrusheva, O. *et al.* Alignments of HiSeq reads for Adineta vaga individuals L1-L11 to A. vaga mitochondrial contigs. Figshare https://doi.org/10.6084/m9.figshare.11396955.v2 (2020).
57. Wilton, P. R., Zaidi, A., Makova, K. & Nielsen, R. A Population Phylogenetic View of Mitochondrial Heteroplasmy. *Genetics* **208**, 1261–1274 (2018).
58. Stewart, J. B. & Chinnery, P. F. The dynamics of mitochondrial DNA heteroplasmy: implications for human health and disease. *Nat. Rev. Genet.* **16**, 530–542 (2015).
59. Yuan, J. D., Shi, J. X., Meng, G. X., An, L. G. & Hu, G. X. Nuclear pseudogenes of mitochondrial DNA as a variable part of the human genome. *Cell Res.* **9**, 281–290 (1999).
60. Cibulskis, K. *et al.* Sensitive detection of somatic point mutations in impure and heterogeneous cancer samples. *Nat. Biotechnol.* **31**, 213–219 (2013).
61. McLaren, W. *et al.* The Ensembl Variant Effect Predictor. *Genome Biol.* **17**, 122 (2016).

62. Hill, W. G. & Weir, B. S. Variances and covariances of squared linkage disequilibria in finite populations. *Theor. Popul. Biol.* **33**, 54–78 (1988).

63. Marroni, F. *et al.* Nucleotide diversity and linkage disequilibrium in Populus nigra cinnamyl alcohol dehydrogenase (CAD4) gene. *Tree Genet. Genomes* **7**, 1011–1023 (2011).

64. Sved, J. A. Linkage disequilibrium and homozygosity of chromosome segments in finite populations. *Theor. Popul. Biol.* **2**, 125–141 (1971).

65. Wakeley, J. Using the variance of pairwise differences to estimate the recombination rate. *Genet. Res.* **69**, 45–48 (1997).

66. Shendure, J. & Akey, J. M. The origins, determinants, and consequences of human mutations. *Science* **349**, 1478–1483 (2015).

67. Mark Welch, J. L. & Meselson, M. Karyotypes of bdelloid rotifers from three families. *Hydrobiologia* **387**, 403–407 (1998).

68. Birky, C. W. Positively negative evidence for asexuality. *J. Hered.* **101 Suppl 1**, S42-45 (2010).

69. Lynch, M. *et al.* Genetic drift, selection and the evolution of the mutation rate. *Nat. Rev. Genet.* **17**, 704–714 (2016).

70. Ardlie, K. *et al.* Lower-Than-Expected Linkage Disequilibrium between Tightly Linked Markers in Humans Suggests a Role for Gene Conversion. *Am. J. Hum. Genet.* **69**, 582–589 (2001).

71. Przeworski, M. & Wall, J. D. Why is there so little intragenic linkage disequilibrium in humans? *Genet. Res.* **77**, 143–151 (2001).

72. Bustamante, C. D. *et al.* Natural selection on protein-coding genes in the human genome. *Nature* **437**, 1153–1157 (2005).

73. Begun, D. J. *et al.* Population Genomics: Whole-Genome Analysis of Polymorphism and Divergence in Drosophila simulans. *PLOS Biol.* **5**, e310 (2007).

74. Gayral, P. *et al.* Reference-Free Population Genomics from Next-Generation Transcriptome Data and the Vertebrate–Invertebrate Gap. *PLOS Genet.* **9**, e1003457 (2013).

75. Signorovitch, A., Hur, J., Gladyshev, E. & Meselson, M. Evidence for meiotic sex in bdelloid rotifers. *Curr. Biol.* **26**, R754–R755 (2016).

76. Judson, O. P. & Normark, B. B. Ancient asexual scandals. *Trends Ecol. Evol.* **11**, 41–46 (1996).

77. Mark Welch, D. B. & Meselson, M. Evidence for the Evolution of Bdelloid Rotifers Without Sexual Reproduction or Genetic Exchange. *Science* **288**, 1211–1215 (2000).

78. Kumar, S., Jones, M., Koutsovoulos, G., Clarke, M. & Blaxter, M. Blobology: exploring raw genome data for contaminants, symbionts and parasites using taxon-annotated GC-coverage plots. *Front. Genet.* **4**, 237 (2013).

79. Kumar, S., Stecher, G. & Tamura, K. MEGA7: Molecular Evolutionary Genetics Analysis Version 7.0 for Bigger Datasets. *Mol. Biol. Evol.* **33**, 1870–1874 (2016).

80. Haller, B. C. & Messer, P. W. SLiM 3: Forward Genetic Simulations Beyond the Wright-Fisher Model. *Mol. Biol. Evol.* **36**, 632–637 (2019).

81. Gurevich, A., Saveliev, V., Vyahhi, N. & Tesler, G. QUAST: quality assessment tool for genome assemblies. *Bioinforma. Oxf. Engl.* **29**, 1072–1075 (2013).
