# Supplementary material for: Genomic signatures of recombination in a natural population of the bdelloid rotifer Adineta vaga
Source: Nat Commun. 2020 Dec 18;11:6421. doi: 10.1038/s41467-020-19614-y (PMC7749112; doi:10.1038/s41467-020-19614-y)
Supplement: Supplementary file 3 — Reporting summary [file 41467_2020_19614_MOESM3_ESM.pdf]

nature research

Corresponding author(s): Olga A. Vakhrusheva

Last updated by author(s): Sep 25, 2020

Reporting Summary

Nature Research wishes to improve the reproducibility of the work that we publish. This form provides structure for consistency and transparency in reporting. For further information on Nature Research policies, see Authors & Referees and the Editorial Policy Checklist .

Statistics

For all statistical analyses, confirm that the following items are present in the figure legend, table legend, main text, or Methods section.

n/a	Confirmed	
☐	☒	The exact sample size (*n*) for each experimental group/condition, given as a discrete number and unit of measurement
☒	☐	A statement on whether measurements were taken from distinct samples or whether the same sample was measured repeatedly
☐	☒	The statistical test(s) used AND whether they are one- or two-sided<br>*Only common tests should be described solely by name; describe more complex techniques in the Methods section.*
☐	☒	A description of all covariates tested
☐	☒	A description of any assumptions or corrections, such as tests of normality and adjustment for multiple comparisons
☐	☒	A full description of the statistical parameters including central tendency (e.g. means) or other basic estimates (e.g. regression coefficient) AND variation (e.g. standard deviation) or associated estimates of uncertainty (e.g. confidence intervals)
☐	☒	For null hypothesis testing, the test statistic (e.g. *F*, *t*, *r*) with confidence intervals, effect sizes, degrees of freedom and *P* value noted<br>*Give P values as exact values whenever suitable.*
☒	☐	For Bayesian analysis, information on the choice of priors and Markov chain Monte Carlo settings
☒	☐	For hierarchical and complex designs, identification of the appropriate level for tests and full reporting of outcomes
☐	☒	Estimates of effect sizes (e.g. Cohen's *d*, Pearson's *r*), indicating how they were calculated

Our web collection on statistics for biologists contains articles on many of the points above.

Software and code

Policy information about availability of computer code

Data collection	Trimmomatic (V0.33), FastQC (v0.11.3), SPAdes (version 3.6.0), QUAST (v5.0.0), SMRT Analysis Portal (v2.3.0), scripts from the Blobology pipeline (revision bc2300c, https://github.com/blaxterlab/blobology), BLAST+ (version 2.2.31), all_bz (v.15), AUGUSTUS (v.2.7), GeneMark.ES Suite (version 4.32), Bowtie 2 (v.2.3.2), SAMtools (v.1.4.1), STAR aligner (v. 2.4.2a), minimap2 (version 2.16-r922), BCFtools (v.1.4.1), GATK (version 4.1.2.0), bedtools (v2.26.0), SnpSift (v.4.3s), RepeatMasker (version open-4.0.7), MUSCLE (version 3.8.31), HapCutToVcf utility from fgbio (version 0.2.0-SNAPSHOT).
Data analysis	BLAST+ (version 2.2.31), BUSCO (v. 3.1.0), VEP (version 96.3), MCScanX (available at http://chibba.pgml.uga.edu/mcscan2/MCScanX.zip; accessed August 28, 2017), PLINK (v1.90b5.4), VCFtools (v. 0.1.15), populations program from the Stacks pipeline (version 2.4), HapCUT2 (revision bd1a739, https://github.com/vibansal/HapCUT2), WhatsHap (version 0.14.1), PhiPack (available at http://www.maths.otago.ac.nz/~dbryant/software/PhiPack.tar.gz; accessed July 1, 2018), LDhat (version 2.2), SplitsTree (version 4.14.6), RDP4 (v.4.97), mlRho (version 2.9), PhyML (version 3.1), MEGA7 (version 7.0.26), RAxML (version 8.2.12), Dendroscope (version 3.5.10), SOWHAT (revision 907c289, https://github.com/josephryan/sowhat), SLiM (version 3.2), awk (version 3.1.7), R (versions 3.3.2 and 3.6.3), ggplot2 R package (version 3.2.1), fANCOVA R package (version 0.5-1), poppr R package (v2.8.6), stats R package (version 3.6.3), boot R package (version 1.3.24). Custom scripts available at https://github.com/vakh57/bdelloid_scripts.

For manuscripts utilizing custom algorithms or software that are central to the research but not yet described in published literature, software must be made available to editors/reviewers. We strongly encourage code deposition in a community repository (e.g. GitHub). See the Nature Research guidelines for submitting code & software for further information.

Data

Policy information about availability of data

All manuscripts must include a data availability statement. This statement should provide the following information, where applicable:

- Accession codes, unique identifiers, or web links for publicly available datasets
- A list of figures that have associated raw data
- A description of any restrictions on data availability

The Illumina sequencing data have been deposited at NCBI under BioProject ID PRJNA498886. Individual SRA accession numbers for deposited HiSeq reads are provided in Supplementary Table 22. MiSeq reads included in the obtained assembly of the A. vaga genome (L1) have been deposited with SRA accession numbers SRR8133179, SRR8133180, and SRR8133181. PacBio reads used to assess accuracy of phasing for L1 have been deposited at NCBI under BioProject ID PRJNA558051.
The assembled (diploid) contigs for A. vaga (L1) are available at NCBI: the Whole Genome Shotgun project has been deposited at DDBJ/ENA/GenBank under the accession WJQV00000000. The version described in this paper is version WJQV01000000.
The L1 diploid contigs are also available at https://doi.org/10.6084/m9.figshare.11620518.v2. The dataset accessible through this link also includes files containing haploid sub-assembly of the L1 genome, annotation of protein-coding genes in the GTF format produced for the L1 diploid contigs, and coordinates of gene models transferred to the haploid sub-assembly. Raw and filtered SNPs identified in L1-L11 (SNP dataset I) are available at https://doi.org/10.6084/m9.figshare.11625780.v2.
The data used in the analysis of mitochondrial variation are available at https://doi.org/10.6084/m9.figshare.12008790.v2 and http://doi.org/10.6084/m9.figshare.11396955.v2. This analysis also involved publicly available sequences of Philodina citrina and Rotaria rotatoria mitochondrial genomes (the respective GenBank accession numbers: FR856884.1 and GQ304898.1). For annotation, we used a publicly available RNA-seq dataset (generated for the A. vaga genome published in 2013) which can be downloaded at http://www.genoscope.cns.fr/adineta/data/Avaga_rnaseq_sort.bam. BUSCO analysis involved publicly available assemblies of A. vaga genome downloaded from http://www.genoscope.cns.fr/adineta/data/Adineta_vaga_v2.0.scaffolds.fa.gz and of A. ricciae genome available at GenBank under the accession GCA_900240375.1. GenBank accession numbers for reference COX1 sequences used in Supplementary Figs. 1-3 are given in Supplementary Data 9.
The source data underlying Fig. 2c, 3c-d, 4a-b, 5, 7, Supplementary Figs. 1-3, 8, 19d, and 27-28 are provided as a Source Data file. Haplotype sequences reconstructed for L6-L9 in the three segments used to produce Fig. 6 are provided as Supplementary Data 7. All other data supporting the findings of this study are available from the corresponding author upon request.

Field-specific reporting

Please select the one below that is the best fit for your research. If you are not sure, read the appropriate sections before making your selection.

☐ Life sciences ☐ Behavioural & social sciences ☒ Ecological, evolutionary & environmental sciences

For a reference copy of the document with all sections, see nature.com/documents/nr-reporting-summary-flat.pdf

Ecological, evolutionary & environmental sciences study design

All studies must disclose on these points even when the disclosure is negative.

Study description	To address the question of whether bdelloid rotifers engage in genetic exchanges, we obtained whole-genome variation data in a bdelloid rotifer Adineta vaga. For this, we established 11 clonal cultures, L1-L11, each started from a single wild-caught rotifer matching the morphological criteria of A. vaga. The genomic DNA of 11 A. vaga cultures was sequenced to the coverage of ~40-100X with Illumina paired-end libraries on the Illumina HiSeq platform. Additionally, we sequenced one of the lineages, L1, on the MiSeq platform to produce a de novo genome assembly which was used as a reference genome in the subsequent analyses. The obtained sequencing data were used to search for signatures of recombination and genetic exchange among A. vaga individuals.
Research sample	A group of 11 A. vaga individuals including 9 individuals sampled from the Moscow region of Russia and 2 individuals sampled from the Kostroma region of Russia, 550 km to the NE. We chose to perform population genetic analyses in A. vaga as it was the first bdelloid species for which a complete genome was sequenced.
Sampling strategy	We sequenced all A. vaga cultures that we were able to establish in the laboratory over the course of ~2.5 years.
Data collection	We extracted individual rotifers from clumps of moss which grew on trunks of aspen Populus tremula at height 120-170 cm. Moss was collected and the corresponding sampling locations were recorded by Yan R. Galimov, Elena A. Mnatsakanova, and Alexey S. Kondrashov.
Timing and spatial scale	Out of the 11 A. vaga individuals used in the study, nine (L1-L4 and L6-L10) were sampled from the Moscow region and two, L5 and L11, sampled from the Kostroma region, 550 km to the NE. Individuals L1, L2, and L4 were extracted from clumps of moss collected in October 2011. Individual L3 was extracted from a clump of moss collected in October 2012. The rest of individuals were extracted from moss samples collected in 2013.<br>We did not predetermine collection periods. Genome sequencing technology used in the current study requires a considerable amount of DNA (~100 ng) which could only be obtained from an A. vaga clonal culture. We were able to establish only 3 clonal cultures from rotifers collected in 2011. A necessity to increase the sample size motivated sample collection carried out in 2012 and 2013.
Data exclusions	Analysis of SNPs revealed that three of the sequenced individuals (L1-L3) form a separate group (Fig. 1c and Supplementary Fig. 8). To reduce the potential effect of population structure, we mainly focused the subsequent analyses on individuals L4-L11 excluding

individuals L1-L3 from consideration. If not indicated otherwise, the reported results are based on the analysis of individuals L4-L11.

Reproducibility	Our study is based on clonal cultures of A. vaga started from 11 individuals isolated from moss samples collected in two different geographical locations in 2011-2013. As such it would be impossible to exactly repeat the entire experiment. It would be of great interest to reproduce our findings in different populations of bdelloid rotifers. However, examining other populations would require a separate research effort.
Randomization	No allocation to groups was undertaken.
Blinding	Not relevant to the study as there was no allocation to groups.

Did the study involve field work? ☒ Yes ☐ No

Field work, collection and transport

Field conditions	We did not record field conditions as the study is based on sequences of genomes, and genomic DNA is not expected to be affected by field conditions.
Location	We collected rotifers from clumps of moss which grew on trunks of aspen Populus tremula at height 120-170 cm near the Hydrobiological Station "Lake Glubokoe" in Ruza district of the Moscow region, Russia, and in the vicinity of village Shilovo in the Manturovo district of the Kostroma region, Russia, 550 km to the NE. Out of the 11 individuals used in the study, nine (L1-L4 and L6-L10) were sampled from the Moscow region and two, L5 and L11, sampled from the Kostroma region. Approximate sampling coordinates are provided in Supplementary Table 1.
Access and import/export	All rotifer individuals used in the current study were extracted from patches of moss collected in the Russian Federation. As we established rotifer cultures, extracted DNA and performed sequencing within the borders of Russian Federation, no import/export restrictions were applicable.
Disturbance	We believe any disturbance caused by our study could be considered negligible, as we collected a limited number of tiny moss patches.

Reporting for specific materials, systems and methods

We require information from authors about some types of materials, experimental systems and methods used in many studies. Here, indicate whether each material, system or method listed is relevant to your study. If you are not sure if a list item applies to your research, read the appropriate section before selecting a response.

Materials & experimental systems

n/a	Involved in the study
☒	☐ Antibodies
☒	☐ Eukaryotic cell lines
☒	☐ Palaeontology
☐	☒ Animals and other organisms
☒	☐ Human research participants
☒	☐ Clinical data

Methods

n/a	Involved in the study
☒	☐ ChIP-seq
☒	☐ Flow cytometry
☒	☐ MRI-based neuroimaging

Animals and other organisms

Policy information about studies involving animals; ARRIVE guidelines recommended for reporting animal research

Laboratory animals	This study did not involve laboratory animals.
Wild animals	This study did not involve wild vertebrate animals.
Field-collected samples	Individual rotifers were isolated from moss and identified to the species level on the basis of morphological criteria. Clonal cultures were started from individuals identified as A. vaga, which were rinsed in Milli-Q water and transferred to 96-well cell-culture plates. To confirm that just a single individual was transferred to each well, plates were visually inspected daily for the next 3 days after inoculation. When a culture reached the size of ~30 individuals, it was transferred to a separate Petri dish containing Milli-Q water. Cultures were kept at 15-20 °C and fed E. coli (DH5-Alpha strain) grown overnight at 37°C in LB medium. When the total number of rotifers in the dish reached ~1,000 or more, they were harvested for DNA extraction.
Ethics oversight	No ethical approval was required as the study did not involve human participants or vertebrate animals.

Note that full information on the approval of the study protocol must also be provided in the manuscript.
