# Supplementary material for: Genomic signatures of recombination in a natural population of the bdelloid rotifer Adineta vaga
Source: Nat Commun. 2020 Dec 18;11:6421. doi: 10.1038/s41467-020-19614-y (PMC7749112; doi:10.1038/s41467-020-19614-y)
Supplement: Supplementary file 4 — Description of Additional Supplementary Files [file 41467_2020_19614_MOESM4_ESM.pdf]

Description of Additional Supplementary Files

Supplementary Data 1: Summary statistics on whole-genome sequencing of 11 wild-caught *A. vaga* individuals. Coverage for each individual was determined based on alignments of Illumina HiSeq paired-end reads against the *A. vaga* L1 diploid genome assembly and against the haploid sub-assembly. Coverage statistics are based on contigs with a minimum length of 1,000 bp. Alignments were performed with Bowtie 2. Coverage statistics for the diploid assembly were computed using the best alignments of paired-end reads reported by Bowtie 2. Paired-end reads mapping to no more than two positions in the diploid assembly were aligned against the haploid sub-assembly. The resulting alignments were additionally filtered (see Supplementary Methods), and only reads uniquely mapped to the haploid sub-assembly were retained. Statistics of coverage for the haploid sub-assembly are based on the final filtered alignments.

Supplementary Data 2: Proportions of homozygous and heterozygous sites within genomes of 11 sequenced *A. vaga* individuals. Presented numbers are based on sites of the haploid sub-assembly simultaneously called in all sequenced individuals L1-L11 and included in the SNP dataset III (n = 58,158,930). Whole-genome statistics as well as numbers for silent and replacement sites are shown. Individuals belonging to the small and the large cluster are highlighted in blue and green respectively.

Supplementary Data 3: Estimates of phasing error rates for phased SNP datasets. Estimates of phasing error rates were obtained for three individuals which were sequenced more than once (L1, L2 and L11). Three independent libraries for individual L1 were sequenced using the Illumina HiSeq, Illumina MiSeq platform and PacBio technology. L11 was sequenced from two independent libraries on the Illumina HiSeq and the Illumina MiSeq platforms. L2 was also sequenced on the Illumina HiSeq and the Illumina MiSeq platforms, but the same library was used in both cases. Estimates of switch error rates are based on comparison of phased blocks recovered from HiSeq and MiSeq reads (L1, L2, L11) or HiSeq and PacBio reads (L1). Detected putative switch errors could reflect PCR template switches as well as erroneous mapping of reads to paralogous regions and other artifacts. In the case of HiSeq-phased data, one-step filtering (removing blocks with conflicting pairs of SNPs) corresponds to the phased dataset 1, while two-step filtering (involving additional quality control based on switch and mismatch quality values) corresponds to the phased dataset 2. Estimates involving phased blocks from the phased dataset 1 used for the majority of the analyses are shown in bold.

Supplementary Data 4: Observed and expected numbers of triallelic sites harboring all three possible heterozygous genotypes among the individuals of the large cluster, L4-L11.

Supplementary Data 5: Incongruent and congruent groupings of the two haplotypes in individuals L4-L11. This is an extended version of Table 1 of the main text. In addition to the data on the numbers of incongruent segments identified in L4-L11, the table shows data on the numbers of congruent segments found in these individuals. For each individual, we computed the number of incongruent phased segments (those where the reciprocal closest counterparts of the two haplotypes were found in two different individuals) and congruent phased segments (those where the reciprocal closest counterparts of the two haplotypes were found in the same individual; see Methods). The table shows the raw numbers of such segments (prior to checking for the presence of the corresponding groupings in the phylogenetic trees and examining the corresponding bootstrap support values) as well as the numbers of the segments remaining after leaving only incongruent/congruent groupings with strong bootstrap support (≥70%). The numbers of identified incongruent/congruent segments along with the numbers of different patterns of haplotype groupings observed for each individual, L4-L11, are shown. The patterns of haplotype groupings observed for each individual are listed, with the number of segments for which each pattern was observed given in parentheses. In the case of incongruent segments, for each individual, each unique pair of other individuals harboring reciprocal closest counterparts of its two haplotypes at least at one locus constitutes a separate pattern of incongruence. For this analysis, we used the segments of the *A. vaga* genome harboring at least 15 non-singleton SNPs simultaneously phased in all individuals L4-L11. Segments with more than two highly similar hits (≥90% identity) in the L1 genome as well as segments harboring multiple paralogous regions were removed from this analysis (see Methods). In total, out of the 303 analyzed phased genomic segments, 79 exhibited incongruent groupings of the two haplotypes at least in one individual (including 52 with strong bootstrap support) and 12 exhibited congruent groupings of the two haplotypes of the same individual (including 10 with strong bootstrap support). Each of the 10 segments with congruent groupings passing the bootstrap filter is identified as congruent simultaneously in two individuals. This is because the employed definition of congruence implies symmetric relationship between haplotypes of a pair of individuals: cases where reciprocal closest counterparts of the two haplotypes of individual 1 are found in individual 2 automatically imply that the reciprocal closest counterparts of the two haplotypes of individual 2 are found in individual 1.

Supplementary Data 6: Recombination events inferred with RDP4 for the three genomic regions in Fig. 6. For each of the three phased genomic regions shown in Fig. 6, the table shows information on recombination events inferred with RDP4 (including *P* values). Those recombination events depicted in Fig. 6 are marked in bold and highlighted in red. The table also provides information on coordinates of the three analyzed segments in the L1 diploid assembly and subregions of the segments used to construct the phylogenetic trees shown in Fig. 6. The table key was generated with RDP4. The presented *P* values were subjected to the default RDP4 Bonferroni correction adjusting for the number of sequence triplets examined for the segment; however, no further correction for the number of analyzed segments was applied. Different methods implemented in RDP4 use different approaches to compute *P* values. RDP and BootScan *P* values are computed from the binomial distribution. GENECONV provides Karlin-Altschul-like *P* values. Roughly, the *P* values for these three methods correspond to the probability to observe sequence similarity at least as

extreme as observed in the putative recombinant region, if there was no recombination (and are essentially one-sided). MaxChi and Chimaera *P* values are based on the 2×2 χ^2 statistic, assessing the significance of the difference in the proportions of polymorphic sites to the left and to the right of the putative recombination breakpoint (the way how polymorphic sites are defined is different for MaxChi and Chimaera). SiScan *P* value estimation employs randomization of sequences and the *Z*-test (two-sided). For details on how different *P* values are computed in RDP4, see the RDP4 manual (available at http://web.cbio.uct.ac.za/~darren/RDP4Manual.pdf; accessed March 2020).

Supplementary Data 7: Haplotype sequences reconstructed for L6-L9 in the three genomic regions in Fig. 6. Sequences are in FASTA format.

Supplementary Data 8: Summary statistics on annotation for SNPs residing within 52 phased genomic segments inferred to be incongruent in L4-L11. SNPs were annotated with VEP relative to the L1 haploid sub-assembly and only a single consequence per SNP was retained (see Supplementary Note 9). Only those SNPs that satisfied all filtering criteria and were simultaneously phased in L4-L11 were considered.

Supplementary Data 9: GenBank accession numbers for *COX1* sequences of reference bdelloid isolates used in Supplementary Figs. 1-3.
