# Supplementary material for: Genomic signatures of recombination in a natural population of the bdelloid rotifer Adineta vaga
Source: Nat Commun. 2020 Dec 18;11:6421. doi: 10.1038/s41467-020-19614-y (PMC7749112; doi:10.1038/s41467-020-19614-y)
Supplement: Supplementary file 2 — Peer Review [file 41467_2020_19614_MOESM2_ESM.pdf]

Reviewers' comments:

Reviewer #1 (Remarks to the Author):

In this paper, Vakhrusheva et al have sequenced 11 genomes, including one as de novo sequenced, to address the population structure of a wild bdelloid rotifer population (<i>Adineta vaga</i>). Based on several lines of genome-wide evidence, they claim ongoing recombination between individuals of this putatively asexual species. The bdelloid rotifer was believed to have persisted and diversified in the absence of sex for millions of years. With molecular data increasing, whether the bdelloid is obligately asexual or of unknown forms of non-canonical sex has become a subject of much debate recently (e.g., Genetics 200: 581; Curr Biol 26: 723; Curr Biol 26: R754; Curr Biol 28: 2436). For these reasons, this manuscript has the potential to attract general interest and indeed makes important advance in this field, if its main finding stands. Although novel evidence and views are always welcome, any claims and conclusions should be addressed very carefully. In general, some methods are either not applied sophisticatedly or explained unclearly to assess whether the corresponding conclusions are reliable. It is a bit difficult to judge the evidence supporting the statements in the paper. More details are below.

1. Sampling
Authors should provide detailed information for each sequenced sample, such as the latitude and longitude location. Thus, readers could judge how representative the 11 samples used in this study. If some individuals were sampled from the same trunk, they possibly shared a very recent common ancestor; thus, they actually represent a single clone. Also, I was wondering whether the separation between samples (Fig. 1c) is related to their geographic locations.

2. De novo assembly of L1
Authors generated a de novo assembly for one of their sequenced individuals, L1, and used this assembly for following analyses. As explained by authors, the sequence identity between their sequenced genomes and the published A. vaga genome is ~88%. If such a high divergence is common within species, I was wondering how diverged inside the sequenced individuals of this study. At least, Fig. 1c suggests that these samples were from two distinct populations. If the sequence identity is quite low, improper mapping and saturated variations would affect the following analyses. Authors could just list the sequence identity between any pair of sequenced individuals.

Additional technical concerns about this part as follows:
(1) Based on the presented N50 size (~20Kb), it is unfair to claim an assembly of high quality. Moreover, author did not present any statistics regarding to the completeness of this assembly (e.g. BUSCO and many other quality control methods). Thus, the statement on line 78 is not justified.
(2) SPAdes seems more common for assembling small genomes such as bacterial. If the Miseq reads of long read length are bridged, I would recommend methods such as Discovar and Allpath-LG for assembling such type of reads.
(3) Authors generated a gene set of 61,531 gene models; the number is much higher than the previously predicted set of A. vaga (49,300; Nature 500: 453). It would be helpful to discuss how the gap occurs: technical bias or duplicated(redundant)/missed/contaminated contents?

3. SNP calling
The methods to call SNPs are not ideal. Authors used “mpileup” of samtools to call SNPs, but this module has been deprecated by the author of samtools for many years. A number of up-to-date algorithms exist, such as GATK. Given the complexity of the rotifer genome, using better methods to call variations would be quite useful. Another issue would be if involved pipelines have been optimized for such a tetraploid genome. Otherwise, alternative quality controls would be helpful to convince us of the accuracy of SNP matrix.

4. LD decay

The authors do not make any attempt to assess the quality of the phasing of their data. Accurate phasing may not be possible with the conditions of this study, such as a small sample size, fragmented contigs, and the polyploidy of genomes. Although a number of hard filters were made, it cannot demonstrate that the phased data is accurate.

[Optional]
It would be helpful if a simulated data set of clonal population (or even additional scenarios) was generated for contrast in the analyses and presentations of LD and HWE, respectively.

5. Evidence of recombination
All presented lines of evidence supporting the existence of recombination are genomic signatures. Alternatively, I think the most intuitive way is to provide local examples to show us how recombination occurred (such as Fig.2 of Genetics 2015 and Fig. 5 of Curr Biol 2016). Also, authors should plot out the global places of recombination sites along the genomes (perhaps, several long contigs), which is useful to compare the global pattern of recombination sites across individuals.

6. H score
Authors defined H score to represent the likelihood of recombination. If recombination indeed occurs between individuals, the frequency should be more or less correlated with the geographic distances. I'm not sure if the difference between H scores can match with the geographic pattern of these samples.

7. Presentation
Overall, the manuscript is not presented well. Some places of the manuscript is jumping, e.g. the paragraph on lines 108-111 interrupts the LD part, introducing several panels of Fig. 3 prior to the first indication of Fig. 2b. Importantly, I assume the publication of a Nature Communications paper should reach the widest possible audience of scientists. However, the manuscript uses too many specialized words, terms, and rationales. I have to frequently switch back and forth between main text and supplementary (even references); otherwise, many places are hardly to be understood without expertise (e.g. Fig. 3e). On the other hand, it would be highly helpful to provide schematic pattern of controls (Oenothera-like meiosis) in Fig. 4 and 5.

Reviewer #2 (Remarks to the Author):

This paper takes a broad view of the issue of whether there is recombination (sex of some sort) in bdelloid rotifers, using a population-genomics sequencing sample of Adineta vaga. The subject material is notable in that many have viewed bdelloids as an enigma – i.e., an apparent violation of the idea that some sort of reasonably frequent recombination is essential to avoiding long-term deleterious mutation accumulation. The authors do not identify a source of recombination, but their analyses of linkage-disequilibrium decay with physical distance on chromosomes does support the existence of such activity. This would seem to close the door on the scandalous bdelloids with respect to recombination, although a number of questions remain open.

Main points:

1. Something more explicit needs to be stated about the source material, as it was unclear whether the isolates were taken from a single tree or multiple trees taken over a large area. Given the presumed typical asexual mode of reproduction, it is surprising that none of the sequenced isolates were clone-mates.

2. Related to this point, it is also surprising that the "population" seems largely consistent with Hardy-Weinberg expectations. This is not really the expectation for a population undergoing long phases of asexual reproduction, unless there was somehow a recent bout of sex, and the authors then happened

to look at the immediate products of random mating.

One issue here is that the very small sample size may have yielded very little power to detect HW violations. A better approach would be to summarize the mean (and distribution) of F\sb{is} over all informative sites, which should have adequate power to determine whether there is general heterozygote excess or deficit.

3. It would be useful to present some basic population-genomic statistics, particularly given that this is a population-genomic data set. For example, what is the basic level of heterozygosity at silent and replacement sites in protein-coding genes? Is there anything unusual going on with respect to the potential strength of purifying selection?

4. Much of the discussion about gene conversion is quite dense and hard to follow. In addition, it is not entirely clear how the authors are viewing gene conversion – I don't think it should be really viewed as being something separate from recombination, as all recombination events are accompanied by gene conversions involving the invading heteroduplex (and most evidence suggests that the majority of recombination events are unaccompanied by crossovers). Most of the profiles in this paper on the decline of LD with physical distance only go out to a few hundred to ~3000 bp, but many estimates of gene-conversion tract lengths suggest a few hundred to several thousand bp on average, so the declines observed may be largely determined by conversion events (whether or not accompanied by crossing over). It ought to be possible to go out to a couple of orders of magnitude distance beyond what is shown, unless the scaffolds are too fragmented.

In addition, to put things in a broader perspective, the authors should put things in context by discussing how these profiles scale in other systems such as flies and vertebrates. One issue with respect to r^2 is that the values have upper bounds that depend on allele frequencies, and can only attain 1.0 when the frequencies at both sites are the same, so this is presumably a source of the enormous noise in the figures.

In addition, the authors may wish to apply Bernhard Haubold's program mlRho, which calculates the correlation of zygosity in an ML framework, and simply requires the quartets of reads at all sites and requires no phasing. This typically leads to very clean recombination-distance profiles, even when taken out to very large distances, say 100 kb if pooled over windows.

Reviewer #3 (Remarks to the Author):

This is a very interesting and timely manuscript performing an important job to test whether bdelloid rotifers really lack genetic exchange, using population data of whole genomes. Bdelloids are hard animals to work with and there is a huge amount of work in the paper, and a very solid and comprehensive set of analyses are performed.

At face value the results do seem to reject a strictly clonal population structure. I do have several major concerns, however, for the manuscript as currently presented. First, the manuscript is very dense and important material is relegated to supplementary info – see detailed suggestions below. Second, the authors assume that bdelloids undergo a transformation-style method of recombination among individuals – rather than sexual reproduction. I do not think this assumption is supported by other papers or by the analyses here and would urge a much more neutral/agnostic approach, or even better to attempt to test normal meiosis as an additional alternative. Third, I'm left with a nagging worry that there are assembly/phasing or other artefacts behind some of the patterns.

There have been a few prominent red herrings in the bdelloid literature, and I really want this manuscript to avoid such issues and help to clarify the field.

My main comments in more detail are:

(1) The manuscript assumes that bdelloids do not undergo meiosis, quoting the lack of proper homology of chromosomes described in line 17. There is no longer positive evidence to support this assumption. The lack of homology was reported in the Flot et al. Nature paper, but it has since been shown to be absent from a second species (A. ricciae), could not be recreated in A. vaga by other assembly methods, and I understand from the original authors that they now believe that result was an assembly artefact.
While the introduction proper does refer to this issue, the abstract makes a strong claim that the results cannot be interpreted as due to meiosis, which then pervades the whole manuscript. An alternative mechanism based on transformation is favoured by the authors throughout, but no definitive analysis is presented here to discriminate meiosis and sexual reproduction from transformation. I think you should step back from ascribing a particular mechanism unless you can strongly discriminate it from alternatives with your analyses and data here. This is especially true since the proposal of a transformation-mechanism is, as the authors state, "unheard of in eukaryotes", and would imply a number of rather extraordinary processes to get kb fragments of DNA into the germline.

(2) The results are dense and it is hard to extract the critical evidence provided by each part. The manuscript is set up to present the decay of LD with distance as the first key result, and then to step through different possible explanations for that pattern. This is a good approach, but it isn't fully executed.
The alternative explanations are: artefact of the assembly process, gene conversion within individuals, mitotic recombination within individuals, some form of meiosis, transformation between individuals. A figure illustrating the main alternatives would be very useful. It makes sense to rule out assembly artefacts first, then proceed in this kind of order. In fact, the text goes from LD result, to a discussion of gene trees (lines 108-111, not fully explaining what hypothesis this addresses), to assembly artefacts, then to gene conversion, then within-individual recombination (although it is not made clear in the main text how this section addresses that alternative), then back to gene trees.
I think the results would be more persuasive and digestible if presented in a more logical way, with hypothesis, prediction, evidence for each possible explanation. Indeed there is an attempt to do this in a supplementary note – I recommend revising to combine the best of both parts and just explain it clearly in main text – no need for a second explanation then.

(3) Assembly/phasing artefacts, lines 112-126. It seems inevitable that any errors in assembly or phasing will increase with increasing distance between SNPs, and hence potentially tend to introduce a signal of LD decay. Furthermore, the steep decline in LD that you observe (e.g. fig 2a) seems to be at roughly the length of your Illumina reads. The L1 genome is not especially well assembled, with N50 stats below other published bdelloid genomes. So I remain concerned that this result is somehow affected by assembly/phasing artefacts. The main text is not fully convincing to exclude this possibility, largely because key details on how you assembled and phased your data are restricted to the supplementary material, and in both the main text and supplementary, the information is presented in a very dense way.
(i) "the decay persists in subsets of polymorphic loci covered by long blocks of collinear genes" – that doesn't reassure me, why should phasing be more reliable for those cases?
(ii) "we filtered the phased haplotypes aggressively" – not enough information on this part to persuade me either way, I don't have a strong prior notion of whether aggressive filtering should reduce possibility for the relevant artefacts here or not.
(iii) Your first unphased method seems the neatest part to get at this – I'd suggest to make this higher profile and cut the weaker alternatives – but even then, with the number of SNPs meeting those criteria, it seems plausible that alternative genotypes of 0/0 and 1/1 at more distant SNPs could reflect assembly artefacts, e.g. alternative pieces of similar DNA being alternatively assembled into that position in different individuals. Gene conversion is another possible contributor to this pattern, as

seems to be acknowledged on line 127.
(iv) I don't understand your second unphased method, having read the supplementary methods a few times. Do you really need this even? For all the methods you use, I suggest adding a figure to the supplementary illustrating the rationale for each method – very hard to unpack all of this from the words.
(v) It might be reassuring if the same phasing were consistently recovered from multiple independent replicates of the same genome. Were any of the "clonal" populations sequenced more than once, and if so, how consistent was the phasing? Alternatively, is it possible to test any of the phasing by long-range amplification and cloning methods?

(4) Gene conversion. The argument in fig 2d and e and lines 137-138 assumes that recurrent mutations are rare. If recurrent mutations are not rare, the 4 genotypes in fig. 2e can be produced from an ancestor by just 2 mutation events – especially if most mutations are transitions, the plausibility of repeat mutations does not seem so unlikely. How the fraction of "recombinant" pairs changes with distance is the pertinent part, because there is no reason for the multiple mutation mechanism to vary with distance. I eventually found this explained clearly in the supplementary (top page 11) – would be useful to bring this reasoning to the main text, and tone down/reword the part about recurrent mutations. Given the range of exotic mechanisms being considered here – e.g. an entirely new mechanism of transformational recombination for animals – assuming a lack of recurrent mutations seems like a weak argument and you don't need it.

(5) Hardy-Weinberg test. This is implied by the flow from line 150 to be a test of mitotic recombination within individuals – please spell out to the reader exactly what would be expected under that mechanism versus between-individual recombination. What is your statistical power to reject the null hypothesis at each locus with 8 individuals sampled? Line 164-166, please expand on this explanation – how precise would this matching need to be and of what kind? How "unlikely" is it that the relevant parameters are constrained in the manner required, by some genetic or population-genetic feedback mechanism? Plausibility arguments are not so relevant when your preferred explanation involves an entirely new transformation mechanism for animals. Are there any conceivable assembly or sequencing artefacts to explain the HW results? These are important arguments for evaluating your alternatives.
Mitotic recombination (i.e. crossing over between homologs within an individual) seems incompletely explored. Is it possible that recombination between homologs within clonal lineages plus gene conversion could alone explain the LD results and four-gamete test? If so, make it clear that the claim of between-individual recombination rests heavily on the interpretation of the H-W results and draws no direct support from LD decay. How far can we get in explaining the data if we posit reciprocal intragenomic crossing over between homologs with preservation of heterozygosity? This seems more parsimonious than either males or transformation, because it would use the same mechanisms as gene conversion, which we know occurs.

(6) The triallelic SNPs part would benefit from a clearer explanation: important detail is relegated to supplementary or not stated. Something like: "a SNP with 3 alleles requires at least 1 mutation in above a 2 allele SNP, hence we estimate the probability of an additional mutation during the history of the sample as $p = N3/(N2+N3)$. Under a mutational mechanism alone, a triallelic SNP with all 3 heterozygotes present requires yet another additional mutation. Hence, based on the probability p and the number of triallelic SNPs, we expect to observe $p*N3 = 83.5$ SNPs with all 3 heterozygotes, but in fact we observe 1839". A diagram might also help. At present, line 176 in the main text reads like an assertion that recurrent mutation is rare, rather than a calculation, which is a much stronger case.
It might help to place the results on triallelic SNPs in context by comparing them with other animals (e.g. Drosophila, humans). I think the incidence of triallelic SNPs reported for rotifers here (0.0086) is several orders of magnitude lower than equivalent rates estimated in clearly sexual species. Perhaps there is some methodological reason for this, but if not it seems to reduce the force of the argument that the incidence of triallelic SNPs is so much higher than expected that it cannot arise from recurrent mutations.

(7) Gene trees (lines 198-220, figure 4). This part is least convincing. The gene trees look very odd in a few ways and it is not obvious they fit the transformation mechanism any better than the Oenothera mechanism (I agree they do not fit that):
(i) There is no signal of an underlying clonal structure to these populations, which seems very surprising for a transformation-type mechanism. It would have to occur at an exceptionally high rate. You estimated 1 transformation event per generation – is this consistent with observing no clonal structure in the gene trees? Even in bacterial species with readily measurable rates of natural competence and homologous recombination, there is typically substantial genome-wide linkage disequilibrium, as expected for organisms whose reproduction is strictly clonal even genetic transmission is not. I note a recent interesting preprint (https://www.biorxiv.org/content/10.1101/385336v1), which concludes that bacterial panmixia is rare and unstable, and evidence of clonal structure is seldom absent.
(ii) Another odd feature is that quite often there are two alleles from different individuals that are not just similar but identical. In many cases: Fig. 4 (a) (b), (c), Table 1, these putatively allele-sharing individuals were collected 550km apart. . What is the probability of observing so many cases of identity for this small sample of individuals, given the considerable phylogenetic "scatter" we see among homologs within each locality and within each individual? It would seem to be rather low by the proposed mechanisms. Some thoughts of things to check: index-hopping or contamination between libraries, were relevant clones multiplexed together; are there any alleles present in L1 also found in the L4 'species', which might indicate index hopping or contamination?
(iii) In contrast, no cases of two identical alleles from the same individual are observed, indeed the 2 alleles within an individual look – by eye – to be over-dispersed in the gene trees, i.e. never particularly close. Again, this does not seem so likely based on the proposed mechanism and our prior information about rates of gene conversion. This made me worried about paralogy. It would be worth blasting each allele back into all genomes just to check they are not present as additional paralogs lost somehow in the construction of your haplotype blocks and filtering.
(iv) It would be useful to have more information on the phased genomic sequences that emerged from filtering used for this analysis – do they contain genes, introns, are the SNPs in coding regions? If the variation is functional, certain combinations could represent selectively favoured genotypes that would attract multiple clones independently without implying genetic exchange.
(v) It could be made clearer in the caption to Figure 4 and elsewhere that these are not a typical sample of trees for randomly selected nuclear markers, but the result of an intensive filtering process designed to locate and enrich for cases of apparent incongruence (lines 784-812). It would be good to estimate what the 'expected' rate might be for detecting incongruence as extreme as this, given the number of regions and permutations examined.

(8) In the final parts, you only compare 2 models: transformation and Oenothera-type meiosis. It is clear from the LD analysis that the present results cannot be explained by the Oenothera model; that point could be made more succinctly. The bigger challenge (perhaps not just for the authors here) is to explain how the results of Signorovitch et al. (2015) can be reconciled with any mechanism proposed in the present manuscript.
What about the 3rd possibility of regular meiosis – are your results explicable by that mechanism or not? What frequency of meiosis relative to apomixis (e.g. 1 sexual generation per 10000 asexual) would be consistent with observed levels of LD decay? I think you have relevant calculations in the supplementary in terms of population-scaled recombination rate.

(9) For repeatability, you need to be more precise with your taxonomy (line 91 and Methods). You talk about Adineta vaga, but Adineta vaga covers a wide array of diversity and different cryptic species. If used in a strict sense, it ought to refer to the original type described by Davis (https://www.researchgate.net/publication/318711223_WHAT_IS_ADINETA_VAGA_DAVIS_1873), or for molecular purposes it might be defined based on the reference strain sequenced by Flot et al. 2013, which is clearly distinct from the individuals here. In either case, I think your animals are better designated as Adineta sp. (perhaps Adineta sp. 'L1-3' and sp. 'L4-11', depending how distinct the two

are by formal analysis).
I'm happy that you took diversity into account for your population analyses and focused on one main cluster. But a tree of your isolates for the cox1 marker (the standard marker for bdelloid DNA taxonomy), with reference sequences from the literature (including the A. vaga 2013 genome clone), would be very useful – more useful than figure 1c or Table S5. That is important information for people to be able to repeat your work and compare the relationships among your clones with those among clones in other salient datasets (e.g. Signorovitch et al. 2015 Genetics; Fontaneto et al. 2011 Hydrobiologia 662:27). You should also report more specifically which collection locality (i.e. tree and patch) each clone came from (with GPS coordinates), and particularly the distance between trees.

Additional comments:

Line 13 – both darwinulids and Timema have documented males, and so are maybe not such reliable examples of long-lived asexuals.

Line 15 – more like 60Mya? Tang et al. 2014. Evolution.

Line 17 – Remove reference to palindromes in abstract, or if refer to, needs to say something like "prior evidence from genomes not confirmed by subsequent work", to illustrate that there is no real evidence of lack of meiosis from current genome evidence.

Line 22 and 66 – saying one of the studies is controversial or has been questioned does not give an accurate impression here. Clear evidence was presented in a refereed paper that the original results were "artifacts of experimental error" arising from accidental contamination between tubes. The editors and reviewers of the journal in question accepted and published this result as "clear evidence that the data and findings of Debortoli et al. (2016) are unreliable". No further debate on the matter has appeared in other peer-reviewed outlets since. That's clear evidence against the original interpretation, not really any controversy. The description is worth clarifying both in the introduction and in the abstract too, as many people only read this.

Line 82- Important to report whether you find any evidence for lack of homology and palindromes or not – I presume not from what is stated, but it needs emphasizing.

Line 127. Why "finally" here – there are still other alternatives to consider after here.

Line 202: Have the authors read in detail the piece of work cited as reference 36? Having previously done so myself at some length, I do not think it adds any substance to the point and does not merit mention in the same sentence as references 17, 34 or 35. It contains no evidence that reported phenotypic effects were caused by DNA uptake or transformation, as opposed to other consequences of the experimental manipulation.

Line 203: Reference 9 is not the most appropriate citation for the hypothesis that intra-specific genetic exchanges might occur in bdelloids. That hypothesis was first put forward clearly and succinctly in Reference 34, which has the further advantage that its data and findings are reliable. Reference 9 has been found to supply "no credible evidence to address that question" (c.f. Reference 10).

Line 249 to 256 on GC content seems speculative and not to add much – I would move that to supplementary and make more space for important parts. The same arguments would apply if the recombination were intragenomic or linked to gene conversion, so this is not a powerful prediction.

Line 257. "Despite near certain lack of meiosis". What is the evidence for this? Apart from lack of males, I don't think you have any – unless you can tease this out more from the analysis. Same again in the Supplement (p.24): "reported lack of homologous chromosomes in A. vaga genome virtually

ruled out conventional sexual reproduction in bdelloid rotifers".

Line 259. Worth clarifying what you mean by 'panmixis'. This doesn't mean equivalent to a fully outcrossing sexual population, but that any individual in your cluster L4-L11 can transfer genes to any other member of that cluster at the calculated rate? Be careful with terminology – if there is no meiosis, then the "mode of reproduction" is clonal/asexual, but with transformation at some unspecified life-history phase (oogenesis? desiccation?). If so, A. vaga is not amphimictic, as that term is commonly understood in animals. Overall, you need to be more critical at how the rate of recombination compares to what you'd see in a fully outcrossing population, which is how people will interpret this section and the word "panmixis".
To further address this, it might be worth adding a brief comparison. How does this rate of recombination/LD decay compare to better-studied models such as Drosophila?

Line 461: The method of rearing a clonal lineage in the lab is partly at odds with the goal of determining bdelloids' mode of reproduction. If the LD and HW results imply high rates of inter-individual recombination, might the single individual transferred into the initial dish have been a fertilised female, in which case she would have produced a shuffled mixture of outcrossed recombinant F1 siblings, each of which would start a different clonal lineage in the same lab dish, where males apparently are no longer produced. That would make reliable phasing impossible and could produce downstream results that might look like evidence of transformation, perhaps including some of the patterns that were excluded from the analysed data as likely artefacts of "PCR template switches" (e.g. repeatedly finding four different haplotypes in a single "individual" where only two are possible, Supplemental, p.7).

Line 470: "the species identity of cultures L1-L11 was additionally confirmed..." Based on Table S5, it is not clear that L1-3 and L4-11 actually share the same species identity; ideally you would conduct a formal analysis to determine whether they ought to be designated as different species, perhaps using a larger dataset to generate trees and conspecificity matrices (e.g. mtCO1 or the nuclear markers reported by Debortoli et al. 2016). If the two are different species, then it is not strictly accurate to talk about 'population structure' when differentiating them. There might also be some questions about using a reference genome from L1 to map the reads from L4-11, although the methods appear to have been carefully implemented and checked to show the results are robust to this.

Supplement, pages 17-18. How did you estimate the effective size of "the A. vaga population" as 10^6? This could be rather low. First, see Section 9 above- what exactly is "the bdelloid rotifer A. vaga" (Line 268) and how do we delineate it into species and populations? More specifically, I have seen unpublished data suggesting that the number of individual rotifers in the genus Adineta on a single moss-covered tree at a single timepoint is between 10^4 and 10^5. The total number of bdelloid rotifers annually washed by rain from a single tree into a single square meter of soil was recently estimated at 1.2 x 10^6 (https://doi.org/10.1186/s12898-018-0198-4). What happens to the calculations in Sections XIV and XV if Ne is increased by several orders of magnitude? Returning to Figure 4, with these sorts of population sizes, what rates of transformation would be required to give a measurable probability of observing any alleles that are identical by transformation between two individuals from a sample of eight, collected 550km apart (e.g. Fig. 4a L4.2/11.2; Fig. 4b L4.2/L5.1; Fig. 4c L5.1/L7.2, L5.2/L8.1)?

Another thought: Did you run your tests on the mitochondrial genome? If so, does that show similar evidence for the decline in LD observed here, or not – that might help further address meiosis versus ameiotic mechanisms (which arguably should apply to mtDNA as well).

Supplementary note – this seems to restate the manuscript, perhaps with clearer explanations of some of the logic employed – I suggest reworking so the main text makes these points clearly enough that a re-explanation in supplementary is not required.

There appears to be no evidence that the two individuals (L5, L11) collected 550km from Moscow show different patterns of incongruence or putative allele sharing than the six individuals collected from Moscow. This seems surprising even given the potentially rapid dispersal abilities of bdelloid rotifers; recent transfer or sex ought to be more evident among individuals collected closer together versus 550km apart. What explanations for the apparent incongruence can we turn to that would take no respect of physical geography? Incidentally, I cannot reconcile the data in Table 1 with the summary in Table S12. How can it be that L4 shares zero patterns of incongruence with L6 in Table S12, when they share three different patterns of incongruence across 6 different segments in the first line of Table 1 (with respect to L7 x 2, L8 x 1 and L11 x 3)?

Tim Barraclough

We would like to thank the three reviewers for careful reading of our manuscript and their thoughtful comments and suggestions. Accordingly, we incorporate many changes in the revised version of the text. These include important new analyses addressing the questions raised. We also hope that the presentation of the manuscript has now been improved. Our point-by-point responses to the reviewers' comments (normal font) are presented below (bold font).

While revising the manuscript based on comments of the reviewers, we obtained new results, which prompted us to re-adjust our initial interpretations and to revise the discussion section of the manuscript accordingly. We are truly grateful to the reviewers who inspired us to obtain these new important data.

Reviewers' comments:

Response to Reviewer 1:

Reviewer #1 (Remarks to the Author):

In this paper, Vakhrusheva et al have sequenced 11 genomes, including one as de novo sequenced, to address the population structure of a wild bdelloid rotifer population (*Adineta vaga*). Based on several lines of genome-wide evidence, they claim ongoing recombination between individuals of this putatively asexual species. The bdelloid rotifer was believed to have persisted and diversified in the absence of sex for millions of years. With molecular data increasing, whether the bdelloid is obligately asexual or of unknown forms of non-canonical sex has become a subject of much debate recently (e.g., Genetics 200: 581; Curr Biol 26: 723; Curr Biol 26: R754; Curr Biol 28: 2436). For these reasons, this manuscript has the potential to attract general interest and indeed makes important advance in this field, if its main finding stands. Although novel evidence and views are always welcome, any claims and conclusions should be addressed very carefully. In general, some methods are either not applied sophisticatedly or explained unclearly to assess whether the corresponding conclusions are reliable. It is a bit difficult to judge the evidence supporting the statements in the paper. More details are below.

1. Sampling

Authors should provide detailed information for each sequenced sample, such as the latitude and longitude location. Thus, readers could judge how representative the 11 samples used in this study. If some individuals were sampled from the same trunk, they possibly shared a very recent common ancestor; thus, they actually represent a single clone. Also, I was wondering whether the separation between samples (Fig. 1c) is related to their geographic locations.

We thank the Reviewer for this suggestion. Detailed information on sampling locations is indeed important to assess analyses presented in the paper. We contemplated the possibility that individual rotifers sampled from the same trunk or from trunks located in a close proximity to one another are likely to share a very recent common ancestor. We devised our sampling strategy bearing this in mind.

First, we did not collect clumps of moss from the same tree or from closely located trees. For our sampling, we chose trees at least 20 m apart. Second, to avoid sequencing clone mates, we only sequenced one isolate among those obtained from a single clump of moss. Therefore, all clonal lineages sequenced in our study were started from individuals collected from separate trunks located at a distance of 20 m apart or more. (In general, establishing *A. vaga* cultures in the laboratory was very difficult; in particular, the vast majority of initially isolated single individuals died without reproducing.) We now include this information in the Methods section and in the caption for Fig. 1. According to the Reviewer's suggestion, we have added a table with sampling locations for each clonal culture, L1-L11, to Supplementary Information (Supplementary Table 1).

We did not detect any obvious association between genetic relatedness and geographic locations. Our analyses revealed the presence of two genetic clusters (Fig. 1c); however, this clustering did not reflect the geography of sampling locations. Out of the 11 individuals used in the study, nine (L1-L4 and L6-L10) were sampled from the Moscow region, and two, L5 and L11, sampled from the Kostroma region, 550 km to the NE. Despite this distance between the two sampling locations, L5 and L11 clearly belong to the large cluster, together with individuals L4 and L6-L10. This is in line with the findings of prior studies (e.g. Fontaneto et al., 2011, https://link.springer.com/article/10.1007/s10750-010-0481-7) reporting wide geographic dispersal of genetically similar 'lineages' of bdelloid rotifers. The lack of geographic clustering between our samples was discussed in the Supplementary Methods of the first version of the manuscript, but we agree that this is an important point and it should be given more attention. Accordingly, we have added the corresponding information to the caption for Fig. 1 and Methods. It is also discussed in the section VIII "MDS analysis and identification of the population outliers" of the Supplementary Methods.

2. De novo assembly of L1

Authors generated a de novo assembly for one of their sequenced individuals, L1, and used this assembly for following analyses. As explained by authors, the sequence identity between their sequenced genomes and the published A. vaga genome is ~88%. If such a high divergence is common within species, I was wondering how diverged

inside the sequenced individuals of this study. At least, Fig. 1c suggests that these samples were from two distinct populations. If the sequence identity is quite low, improper mapping and saturated variations would affect the following analyses. Authors could just list the sequence identity between any pair of sequenced individuals.

The information on genomic divergence between the individuals sequenced in our study was present in the first version of the manuscript as a part of the Supplementary Methods: "The average pairwise genotypic distance was 1.56% for the individuals belonging to different clusters, 0.85% for the 3 individuals belonging to the small cluster, and 0.67% for the 8 individuals belonging to the large cluster." We now report this information in the main text. Sequence distances for all pairwise comparisons among the individuals L1-L11 are provided as Supplementary Table 8. Of note, although sequenced individuals can be clearly assigned to two distinct clusters, absolute genomic divergence between the individuals from different clusters is quite low (1.56%).

Additional technical concerns about this part as follows:
(1) Based on the presented N50 size (~20Kb), it is unfair to claim an assembly of high quality. Moreover, author did not present any statistics regarding to the completeness of this assembly (e.g. BUSCO and many other quality control methods). Thus, the statement on line 78 is not justified.

Assembling a genome for a bdelloid rotifer is a tricky task and we tried out several approaches to obtain an assembly with adequate levels of haplotype resolution and continuity. Still, we agree that this assembly is not "high quality" by most standards, and no longer refer to it as such.

To more thoroughly characterize the L1 genome assembly, we have followed the suggestion of the Reviewer and have added results of BUSCO analysis, contrasting our assembly to previously published bdelloid genomes (Supplementary Methods II, Supplementary Figs. 6 and 7). This analysis showed that from the point of view of completeness, L1 assembly is fairly good. Moreover, in terms of completeness it is very similar to the previously published bdelloid genomes (*A. vaga* from Flot et al., 2013 and *A. ricciae* from Nowell et al., 2018; please see Supplementary Figs. 6 and 7).

(2) SPAdes seems more common for assembling small genomes such as bacterial. If the Miseq reads of long read length are bridged, I would recommend methods such as Discovar and Allpath-LG for assembling such type of reads.

The choice of the assembler (SPAdes) was motivated by its ability to be run in the 'diploid' mode aimed at separation of haplotypes during the assembly. We sought to obtain an assembly with a high fraction of loci assembled to the level of separate haplotypes due to the following reasons. Our analysis is based on calling SNPs in multiple genomes from reads mapped to a haploid sub-assembly of the single genome (L1). This allows us to call diploid variants, because homologous sites of both haplotypes from each individual are aligned to the same site of the sub-assembly. However, this procedure assumes that only truly diploid loci are included in the haploid sub-assembly. Therefore, we can utilize only those loci that are present as two haplotypes in the original assembly, as we cannot be sure of the ploidy of loci with haplotypes possibly collapsed into a single contig. For these reasons, it is desirable to maximize separation of haplotypes.

We tried out several assemblers before settling on SPAdes, which performed significantly better than other algorithms. For example, we tried to assemble L1 genome from merged overlapping MiSeq reads using Newbler – an approach which proved to be efficient in the assembly of a plant genome with divergent subgenomes (Kasianov et al. 2017; The plant journal). However, in the case of the *A. vaga* L1 genome, this technique resulted in a very fragmented assembly (N50~2,500 bp vs 18,000 bp obtained with SPAdes).

We carefully considered the Reviewer's recommendations on using other methods to assemble L1 genome. However, some data requirements of DISCOVAR and Allpath-LG make their application in case of L1 genome assembly unfeasible. In particular, Allpath-LG is not suited to assemble reads from a single short-insert library (all MiSeq reads for L1 were obtained by sequencing the same library). While the other suggested assembler, DISCOVAR, does not require multiple Illumina libraries, its authors recommend using a PCR-free protocol for library construction, which was not applicable in our case due to low DNA content of our samples.

(3) Authors generated a gene set of 61,531 gene models; the number is much higher than the previously predicted set of A. vaga (49,300; Nature 500: 453). It would be helpful to discuss how the gap occurs: technical bias or duplicated(redundant)/missed/contaminated contents?

The number of gene models predicted in bdelloid genomes is highly dependent on the technicalities of the gene prediction software and filtering criteria employed. The procedure in (Nature 500: 453) initially yielded 94,395 gene models (Supplementary text C3 of that paper https://media.nature.com/original/nature-assets/nature/journal/v500/n7463/extref/nature12326-s1.pdf), which was subsequently reduced to 49,300 as a result of filtering.

By contrast, we started from a lower number of raw gene models (75,877), but ended up with a higher final number (61,531). A recent reannotation of the 2013 *A. vaga* genome assembly (Nowell et al., 2018) yielded 67,364 genes (57,431 high quality CDSs), which is close to our numbers. Nowell et al. (2018) also annotated the genome of a closely related bdelloid species *A. ricciae*, predicting 55,801 genes. In summary, the exact number of genes identified in bdelloid genomes heavily depends on the gene predicting and filtering strategies. This is now discussed in Supplementary Methods IV.

3. SNP calling

The methods to call SNPs are not ideal. Authors used “mpileup” of samtools to call SNPs, but this module has been deprecated by the author of samtools for many years. A number of up-to-date algorithms exist, such as GATK. Given the complexity of the rotifer genome, using better methods to call variations would be quite useful. Another issue would be if involved pipelines have been optimized for such a tetraploid genome. Otherwise, alternative quality controls would be helpful to convince us of the accuracy of SNP matrix.

**We thank the Reviewer for this criticism. Regarding “samtools mpileup”: first, the original “samtools mpileup” command in fact is not deprecated but rather included in the up-to-date version of bcftools package. According to the current bcftools manual, “the mpileup command was transferred to bcftools in order to avoid errors resulting from use of incompatible versions of samtools and bcftools”.
Second, “mpileup” was only utilized to produce pileup output from BAM files. The actual SNP calling was performed with “bcftools call” command, which is unarguably supported by the authors.**

**We agree that newer methods to call SNPs exist, although none are ideal (e.g. Hwang et al., 2015; https://www.nature.com/articles/srep17875). There are several considerations supporting our choice. While GATK is indeed considered the gold standard for SNP identification in humans, SAMtools/BCFtools are still widely used to call SNPs in non-model organisms. Many recent high-profile papers employ SAMtools/BCFtools for SNP detection, including those published in Nature Communications (Park et al., 2018) https://www.nature.com/articles/s41467-018-07370-z,
(Milanese et al., 2019) https://www.nature.com/articles/s41467-019-08844-4,
(Kearns et al., 2018) https://www.nature.com/articles/s41467-018-03294-w,
PNAS (Guellil et al., 2018)
https://www.pnas.org/content/115/41/10422,**

PLOS Biology (Gilabert et al., 2018)
https://journals.plos.org/plosbiology/article?id=10.1371/journal.pbio.2006035 and
Nature Ecology and Evolution (Gaither et al., 2018)
https://www.nature.com/articles/s41559-018-0482-x.

A strength of GATK is its ability to assess and adjust the quality of raw SNP calls using the set of known validated variants. Such datasets of high-quality variants are available for several model species, most notably, *H. sapiens*. Unfortunately, there is no such dataset for *A. vaga,* as this study is the first attempt to obtain whole-genome polymorphism data in it (or any bdelloid rotifer). In the absence of a 'reference' set of SNP calls, we cannot exploit this feature of GATK.

GATK is also more accurate than other algorithms in detection of insertions and deletions. However, even in the case of human genomes, the rate of false positive indel calls made with GATK as well as with other methods is quite high (e.g. Cornish and Guda, 2015 https://www.ncbi.nlm.nih.gov/pmc/articles/PMC4619817/). We were concerned that for the complex *A. vaga* genomes, indel calls would be especially prone to errors. For this reason, we chose to exclude indels from our analyses, and filtered out SNPs in close proximity of an indel.

Given that GATK has been originally developed for use with human genomes and that we would not be able to fully utilize its capabilities, we opted to apply SAMtools/BCFtools, a pipeline widely used in non-human genomics.

That said, the complex structure of *A. vaga* genome harbouring divergent haplotypes along with remnants of whole-genome duplication can indeed make it difficult to obtain high-confidence SNPs. To gain more confidence in our SNP calls, we have now performed SNP calling with GATK as suggested by the Reviewer, and compared it with the original set of SNPs generated with SAMtools/BCFtools and used in the analyses. We have now added the relevant discussion to the Methods section.

We showed that ~88% of raw SNPs called with SAMtools for L1-L11 were identically called with GATK (Methods, Supplementary Table 7). This rate of concordance is similar to that reported for different variant callers on human data (57%-92%; O'Rawe et al., 2013; https://genomemedicine.biomedcentral.com/articles/10.1186/gm432; Hwang et al., 2015; https://www.nature.com/articles/srep17875; Cornish et al. 2015).

Moreover, after the filtering applied in our study, the proportion of SNPs identically called with SAMtools and GATK among those in the SAMtools dataset increased to ~95% (Supplementary Table 7). This indicates that the employed

filtering indeed resulted in exclusion of the majority of low-confidence SNP calls. This concordance rate is comparable to the upper estimates of concordance reported for different variant callers on human datasets (92%, Hwang et al., 2015), which are arguably expected to be less problematic in terms of SNP calling.

4. LD decay

The authors do not make any attempt to assess the quality of the phasing of their data. Accurate phasing may not be possible with the conditions of this study, such as a small sample size, fragmented contigs, and the polyploidy of genomes. Although a number of hard filters were made, it cannot demonstrate that the phased data is accurate.

We agree that the quality of phasing is crucial. To address this concern, we now validate phasing using two independent technologies: PacBio (for individual L1) and Illumina MiSeq (for individuals L1, L2 and L11). We compare the phasing results obtained using these approaches to those in the original manuscript based on Illumina HiSeq. In the new text, we show that the concordance between approaches is high, and that the observed LD decay is not likely to be explained by phasing errors. The details are provided in Supplementary Note 2 and in the main text of the manuscript.

[Optional]
It would be helpful if a simulated data set of clonal population (or even additional scenarios) was generated for contrast in the analyses and presentations of LD and HWE, respectively.

We thank the Reviewer for this important suggestion. We have now implemented it, contrasting the patterns of HWE observed in the actual data to those in simulations. This analysis allowed us to show that the observed low absolute values of inbreeding coefficient F_{IS} (~0) are not compatible with strict clonality and extremely rare rates (<<1%) of sexual reproduction (Fig. 3b).

We have also added a plot showing patterns of LD in simulated clonal and sexual populations (Supplementary Fig. 9).

5. Evidence of recombination

All presented lines of evidence supporting the existence of recombination are genomic signatures. Alternatively, I think the most intuitive way is to provide local examples to show us how recombination occurred (such as Fig.2 of Genetics 2015 and Fig. 5 of Curr Biol 2016). Also, authors should plot out the global places of recombination sites along the genomes (perhaps, several long contigs), which is useful to compare the global pattern of recombination

sites across individuals.

We agree that providing local examples of recombination might be a more intuitive way, however, our study in contrast to prior works addressing putative genetic exchange in bdelloids is focused on genome-wide signatures of recombination. While Signorovitch et al. (Genetics 2015) and Debortoli et al. (Curr Biol 2016) analyzed in detail 4 and 3 nuclear loci respectively, our analysis involves a substantial fraction of the *A. vaga* genome. Here, we should note that simultaneous analysis of LD in 8 individuals requires that all considered SNPs must be phased in all 8 individuals. Due to this requirement and limits on the phasing span imposed by short Illumina reads we deal with a large number of fragmented genomic regions. As such we think that plotting places of recombination for relatively short loci we analyze would not add much to the conclusions of the manuscript and would probably deflect the reader's attention away from the whole-genome signatures which we believe are one of the strongest aspects of our study.

6. H score

Authors defined H score to represent the likelihood of recombination. If recombination indeed occurs between individuals, the frequency should be more or less correlated with the geographic distances. I'm not sure if the difference between H scores can match with the geographic pattern of these samples.

We see no association between H scores and sampling locations. This is perhaps surprising, but matches the results obtained in prior works as well as the lack of geographic structure observed here, and could probably be explained by very efficient dispersal of bdelloids.

First, prior publications have reported that genetic relatedness between *Adineta* individuals is often not associated with distances between sampling locations. For example, Fig. 1 from the paper (Fontaneto et al., 2011) https://link.springer.com/article/10.1007/s10750-010-0481-7 shows that some *A. vaga* individuals from the UK are more closely related to Tanzania individuals sampled from similar habitats than to other UK individuals. Our study recapitulated a similar pattern – although on a smaller geographical scale – with several individuals collected in the Moscow region (L4 and L6-L10) being more closely related to individuals L5 and L11 collected in the Kostroma region, 550 km to the NE, than to the three other individuals L1-L3 sampled from the Moscow region (Fig. 1c).

Second, an even more peculiar feature has been reported by Signorovitch et al. (Genetics 2015) who observed an individual of a bdelloid rotifer *Macrotrachela quadricornifera* sharing alleles with individuals collected from sites located more than 240 km away. Moreover, they found a second-order clustering of two US

individuals with an individual from Milan, Italy (more than 6,000 km away).

Of note, the small sample size (8 individuals) is likely to limit our ability to detect fine-scale differences in probability of genetic exchange between individuals from remote locations. Perhaps increasing the sample size by a factor of 10 or more could reveal subtle differences in likelihoods of recombination that should exist between individuals collected from different sites. This is an interesting subject for further study.

7. Presentation

Overall, the manuscript is not presented well. Some places of the manuscript is jumping, e.g. the paragraph on lines 108-111 interrupts the LD part, introducing several panels of Fig. 3 prior to the first indication of Fig. 2b. Importantly, I assume the publication of a Nature Communications paper should reach the widest possible audience of scientists. However, the manuscript uses too many specialized words, terms, and rationales. I have to frequently switch back and forth between main text and supplementary (even references); otherwise, many places are hardly to be understood without expertise (e.g. Fig. 3e). On the other hand, it would be highly helpful to provide schematic pattern of controls (Oenothera-like meiosis) in Fig. 4 and 5.

We agree that the first version of the manuscript did not read especially smoothly. We have substantially expanded and rewritten the text of the manuscript and hope that it became far more readable. We also added a schematic figure (Fig. 7) illustrating main hypotheses and their predictions.

Response to Reviewer 2:

Reviewer #2 (Remarks to the Author):

This paper takes a broad view of the issue of whether there is recombination (sex of some sort) in bdelloid rotifers, using a population-genomics sequencing sample of Adineta vaga. The subject material is notable in that many have viewed bdelloids as an enigma – i.e., an apparent violation of the idea that some sort of reasonably frequent recombination is essential to avoiding long-term deleterious mutation accumulation. The authors do not identify a source of recombination, but their analyses of linkage-disequilibrium decay with physical distance on chromosomes does support the existence of such activity. This would seem to close the door on the scandalous bdelloids with respect to recombination, although a number of questions remain open.

We would like to thank the Reviewer for the interest in our work and for the general positive evaluation. Most importantly, we do agree that the main result of our study is unambiguous evidence for recombination in this system.

Main points:

1. Something more explicit needs to be stated about the source material, as it was unclear whether the isolates were taken from a single tree or multiple trees taken over a large area. Given the presumed typical asexual mode of reproduction, it is surprising that none of the sequenced isolates were clone-mates.

We thank the Reviewer for this comment, which has also been raised by Reviewer 1. We have now added the required information on sampling procedure to the Methods section and to the caption for Fig. 1 of the main text. To avoid sequencing clone-mates, we did not sequence isolates from the same tree, and isolates collected in the same area were sampled from trees at least 20 m apart. We have also added a table with sampling locations for each clonal culture, L1-L11, to Supplementary Information (Supplementary Table 1).

2. Related to this point, it is also surprising that the "population" seems largely consistent with Hardy-Weinberg expectations. This is not really the expectation for a population undergoing long phases of asexual reproduction, unless there was somehow a recent bout of sex, and the authors then happened to look at the immediate products of random mating.

One issue here is that the very small sample size may have yielded

very little power to detect HW violations. A better approach would be to summarize the mean (and distribution) of F\sb{is} over all informative sites, which should have adequate power to determine whether there is general heterozygote excess or deficit.

Counterintuitively, it takes very little sex to approach the Hardy-Weinberg equilibrium (HW), and severe HW violations are expected only in populations reproducing exclusively asexually or undergoing extremely rare sexual reproduction; this has been demonstrated in a paper by (Balloux et al., 2003) http://www.genetics.org/content/164/4/1635. The Figure 1 from that paper illustrates that populations with low proportions of sexual reproduction behave similarly to strictly sexual populations in terms of F_{IS}.

Thank you very much for the suggestion regarding the F_{IS}. Indeed, the exact test that we employed to detect HW violations in the first version of the manuscript has little power with the sample size of 8 individuals. As suggested, we now provide F_{IS} statistics evaluated from the data (Fig. 3a, Supplementary Table 14) and compare it to F_{IS} obtained for simulated populations reproducing clonally or sexually (Fig. 3b). Our rejection of strict asexuality holds.

3. It would be useful to present some basic population-genomic statistics, particularly given that this is a population-genomic data set. For example, what is the basic level of heterozygosity at silent and replacement sites in protein-coding genes? Is there anything unusual going on with respect to the potential strength of purifying selection?

Thank you for this important suggestion. We have now added a table presenting whole-genome levels of heterozygosity as well as levels of heterozygosity at silent and replacement sites for all 11 individuals (please see Supplementary Table 9 and Supplementary Methods IX), and discuss it in the main text. We have also estimated the Watterson's theta for 11 individuals using the maximum-likelihood method implemented in mlRho (Supplementary Table 10).

Interestingly, individuals from the small and large clusters exhibit notable differences in levels of intraindividual heterozygosity: the mean genomic fraction of heterozygous sites per individual is 2.46% for the small cluster and only 0.76% for the large cluster (see Supplementary Table 9). The corresponding values for silent (four-fold synonymous) sites are 5.01% and 1.54%. Analysis of haplotype phylogenies for all 11 individuals revealed that this difference in heterozygosity is likely to be explained by the hybrid origin of the small cluster; this is now discussed in the main text.

The ratios of heterozygosity at replacement relative to silent sites for the sequenced individuals are around 0.3 for both

clusters. This value is somewhat higher than the ratio usually observed in eukaryotes with strictly sexual reproduction, possibly indicating relaxation of natural selection against deleterious mutations in *A. vaga*. This is now discussed.

We believe that a thorough analysis of the strength of purifying selection in *A. vaga* is needed, but is hard to accomplish in this study. The complex nature of bdelloid rotifers' genomes must be carefully accounted for while carrying out pN/pS and other similar calculations. One issue is that our SNPs are called against one of the two haplotypes. We actually plan to perform an in-depth analysis of the strength of purifying selection in *A. vaga* as a future development of the current study.

4. Much of the discussion about gene conversion is quite dense and hard to follow. In addition, it is not entirely clear how the authors are viewing gene conversion – I don't think it should be really viewed as being something separate from recombination, as all recombination events are accompanied by gene conversions involving the invading heteroduplex (and most evidence suggests that the majority of recombination events are unaccompanied by crossovers). Most of the profiles in this paper on the decline of LD with physical distance only go out to a few hundred to ~3000 bp, but many estimates of gene-conversion tract lengths suggest a few hundred to several thousand bp on average, so the declines observed may be largely determined by conversion events (whether or not accompanied by crossing over). It ought to be possible to go out to a couple of orders of magnitude distance beyond what is shown, unless the scaffolds are too fragmented.

According to the Reviewer's suggestion, we have expanded the discussion related to gene conversion, and revised it for clarity. Regarding the relatively short scale at which we were able to assess the decay of LD: our analysis of LD patterns is based on the phased haplotype data and the phasing has been performed from short Illumina reads. Length of reads imposes limits on the attainable phasing span. Indeed, mean span of phased blocks for different individuals from the large cluster (L4-L11) ranged from 697 to 1,060 bp. Please see Supplementary Tables 11 and 12 for more detailed statistics. In addition simultaneous analysis of LD in 8 individuals requires that all considered SNPs must be phased in all 8 individuals, and this requirement further reduces the maximal distance at which we can assess LD.

We agree that short-scale LD decay measured in a conventional way as r^2 could largely be caused by gene conversion events. To show that LD decay cannot be attributed to gene conversion alone, we carried out the modified four-gamete test considering for a pair of individuals only those pairs of SNPs that are simultaneously heterozygous in these two individuals. Our analysis revealed that among the pairs of SNPs each heterozygous in two individuals, the fraction of those giving rise

to all four possible haplotypes (Supplementary Fig. 14) in these individuals increases rapidly with the physical distance between SNPs (Fig. 2f, g). As gene conversion alone cannot produce a pair of heterozygous sites, represented by all four haplotypes, this pattern is indicative of LD decay not attributable solely to gene conversion.

Nevertheless, we agree with the general criticism over small physical scale at which we assessed LD decay. To go out to a larger distance, we have now added a LD-related plot focusing on two individuals from the small cluster, L1 and L2, which were sequenced using different instruments. Due to higher level of intra-individual heterozygosity, haplotypes for individuals from the small cluster (L1-L3) show improved assembly statistics compared to individuals from the large cluster. This allows to compare fractions of heterozygous SNP pairs harboring all four haplotypes in L1 and L2 going up to ~20,000 bp. Please see Supplementary Fig. 15 for the corresponding plot. This plot also demonstrates that the increase in the fraction of SNP pairs passing the modified four-gamete test is largely unaffected by the type of reads (MiSeq, HiSeq or PacBio) used to carry out phasing and by the stringency of filtering.

According to the Reviewer's suggestion, we also added a plot showing LD decline with distance assessed as decay in correlation of zygosity inferred for separate individuals with mlRho (Supplementary Fig. 13). In this analysis it was also possible to go to 20,000 bp (maximum likelihood estimates of zygosity correlation at larger distances were too noisy).

In addition, to put things in a broader perspective, the authors should put things in context by discussing how these profiles scale in other systems such as flies and vertebrates. One issue with respect to r^2 is that the values have upper bounds that depend on allele frequencies, and can only attain 1.0 when the frequencies at both sites are the same, so this is presumably a source of the enormous noise in the figures.

We agree that it is essential to discuss patterns of LD decay observed in *A. vaga* in context of other species engaging in normal sexual reproduction. We have added the relevant comparison with *D. melanogaster* and *H. sapiens*.

In addition, the authors may wish to apply Bernhard Haubold's program mlRho, which calculates the correlation of zygosity in an ML framework, and simply requires the quartets of reads at all sites and requires no phasing. This typically leads to very clean recombination-distance profiles, even when taken out to very large distances, say 100 kb if pooled over windows.

We thank the Reviewer for the suggestion to use mlRho. We applied mlRho to the filtered BAM files generated for several individuals. The resulting recombination-distance profiles are

shown in Supplementary Fig. 13. We showed estimates of correlation in zygosity for distances up to 20,000 bp, as estimates at larger distances are very noisy (most likely due to reduced number of observations).

Response to Reviewer 3:

Reviewer #3 (Remarks to the Author):

This is a very interesting and timely manuscript performing an important job to test whether bdelloid rotifers really lack genetic exchange, using population data of whole genomes. Bdelloids are hard animals to work with and there is a huge amount of work in the paper, and a very solid and comprehensive set of analyses are performed.

We thank the Reviewer for a high esteem of our work!

At face value the results do seem to reject a strictly clonal population structure. I do have several major concerns, however, for the manuscript as currently presented. First, the manuscript is very dense and important material is relegated to supplementary info – see detailed suggestions below.

Indeed, the first version of our manuscript was dense. Now this is remedied, and hopefully, our manuscript became significantly more readable.

Second, the authors assume that bdelloids undergo a transformation-style method of recombination among individuals – rather than sexual reproduction. I do not think this assumption is supported by other papers or by the analyses here and would urge a much more neutral/agnostic approach, or even better to attempt to test normal meiosis as an additional alternative.

Thank you for this insightful comment. Initially, we believed that males in bdelloids are exceedingly unlikely, but this followed from general considerations and not from our data. The data presented in the first version of the manuscript could not discriminate between the two feasible mechanisms of genetic exchanges between our rotifers: transformation and meiotic sex. We have now updated the manuscript expanding the analysis of haplotype phylogenies to all 11 individuals, L1-L11, from both genetic clusters. This new analysis revealed patterns that appear to be incompatible with transformation, but are consistent with conventional meiosis. Namely, at the majority of the assessed loci, the individuals of the small cluster, L1-L3, carry one haplotype similar to those present in L4-L11 (group 1) and one haplotype more distantly related to haplotypes of L4-L11 (group 2). In other words, L1, L2 and L3 are usually clustered by one of the two haplotypes, but not by the other one.

These findings would be hard to explain under the transformation scenario, but they could be reconciled with the

rest of our data if we assume that the three individuals of the small cluster represent a hybrid between individuals from the population of the large cluster and some more distantly related population. As such, we now believe that conventional meiosis seems to be the more likely explanation for the observed patterns, and no longer promote transformation as the explanation of choice. Still, of course, the exact mode of genetic exchanges in bdelloids merits further investigation. Please see below and the main text for details.

Third, I'm left with a nagging worry that there are assembly/phasing or other artefacts behind some of the patterns.

Indeed, it could be that some of our analyses are not 100% water-tight. However, we believe that together they prove, beyond reasonable doubt, that our rotifers regularly engage in genetic exchanges, by whatever means. See also comments on assembly/ phasing validation above and below.

There have been a few prominent red herrings in the bdelloid literature, and I really want this manuscript to avoid such issues and help to clarify the field.

We tried!

My main comments in more detail are:

(1) The manuscript assumes that bdelloids do not undergo meiosis, quoting the lack of proper homology of chromosomes described in line 17. There is no longer positive evidence to support this assumption. The lack of homology was reported in the Flot et al. Nature paper, but it has since been shown to be absent from a second species (A. ricciae), could not be recreated in A. vaga by other assembly methods, and I understand from the original authors that they now believe that result was an assembly artefact.
While the introduction proper does refer to this issue, the abstract makes a strong claim that the results cannot be interpreted as due to meiosis, which then pervades the whole manuscript. An alternative mechanism based on transformation is favoured by the authors throughout, but no definitive analysis is presented here to discriminate meiosis and sexual reproduction from transformation. I think you should step back from ascribing a particular mechanism unless you can strongly discriminate it from alternatives with your analyses and data here. This is especially true since the proposal of a transformation-mechanism is, as the authors state, "unheard of in eukaryotes", and would imply a number of rather extraordinary processes to get kb fragments of DNA into the germline.

We agree that currently is seems more plausible that the assembly of Flot et al. was incorrect and, therefore, the gross structure of *A. vaga* genome is consistent with meiotic recombination. We changed the text accordingly. As a side note, the intensive acquisition of DNA from foreign species by rotifer germline is supported by a lot of evidence, which has not been questioned, and the within-species transformation could rely on similar means.

However, notably, the new analysis of haplotype phylogenies extended to all 11 individuals L1-L11 (from both genetic clusters) and included in the revised version of the manuscript revealed patterns suggestive of conventional meiosis.

(2) The results are dense and it is hard to extract the critical evidence provided by each part. The manuscript is set up to present the decay of LD with distance as the first key result, and then to step through different possible explanations for that pattern. This is a good approach, but it isn't fully executed.
The alternative explanations are: artefact of the assembly process, gene conversion within individuals, mitotic recombination within individuals, some form of meiosis, transformation between individuals. A figure illustrating the main alternatives would be very useful. It makes sense to rule out assembly artefacts first, then proceed in this kind of order. In fact, the text goes from LD result, to a discussion of gene trees (lines 108-111, not fully explaining what hypothesis this addresses), to assembly artefacts, then to gene conversion, then within-individual recombination (although it is not made clear in the main text how this section addresses that alternative), then back to gene trees.
I think the results would be more persuasive and digestible if presented in a more logical way, with hypothesis, prediction, evidence for each possible explanation. Indeed there is an attempt to do this in a supplementary note – I recommend revising to combine the best of both parts and just explain it clearly in main text – no need for a second explanation then.

Expanded.

(3) Assembly/phasing artefacts, lines 112-126. It seems inevitable that any errors in assembly or phasing will increase with increasing distance between SNPs, and hence potentially tend to introduce a signal of LD decay. Furthermore, the steep decline in LD that you observe (e.g. fig 2a) seems to be at roughly the length of your Illumina reads. The L1 genome is not especially well assembled, with N50 stats below other published bdelloid genomes. So I remain concerned that this result is somehow affected by assembly/phasing artefacts. The main text is not fully convincing to exclude this possibility, largely because key details on how you assembled and phased your data are

restricted to the supplementary material, and in both the main text and supplementary, the information is presented in a very dense way.

Thank you for this criticism. We agree that phasing errors could affect our results and introduce a signal indistinguishable of that of LD decay. Assessing to what extent phasing errors could affect our analysis is therefore crucial to judge the conclusions of our study. We have now added analyses aimed at estimating the phasing error rate in our data (Supplementary Note 2). Following your suggestion, we compared results of phasing recovered from different sets of reads for 3 individuals which were sequenced more than once (these are L1, L2 and L11). L1 was sequenced three times from separate libraries on the Illumina Hiseq, Illumina MiSeq and PacBio platform. For L11, two independent libraries were sequenced using Illumina Hiseq and Ilumina MiSeq. In case of L2, a single library was sequenced both on the Illumina Hiseq and Illumina MiSeq.

We showed that switch error rate as assessed from testing HiSeq-based phased blocks against MiSeq-based phased blocks or against PacBio-based phased blocks (in case of L1) is low: estimates of average per contig switch error rate are on the order of 10^{-3} prior to filtering and on the order 10^{-5}-10^{-4} after filtering out phased blocks with conflicting pairs of SNPs (please see Supplementary Table 13 and Supplementary Note 2).

Moreover, applying the four-gamete test to pairs of individuals L2-L1 and L11-L1 for which more than one phased dataset is available, shows that the increase in the fraction of recombinant SNP pairs with distance is virtually independent of which type of data is used to assemble haplotypes and of severity of filtering (please see Supplementary Figs. 15 and 16).

To show that LD decay cannot be attributed to phasing errors, we contrasted fractions of recombinant SNP pairs detected when comparing haplotypes of two different individuals (L2-L1 or L11-L1) to the corresponding fractions observed when comparing haplotypes of the same individual reconstructed from different sets of reads. In both cases pairs of sites affected by phasing errors could contribute to pools of pairs inferred to be recombinant. Conversely, if there is recombination, we would expect it to contribute to the pool of recombinant SNP pairs inferred from comparison of different individuals, but not to the pool of SNP pairs inferred to be 'recombinant' when testing different phased datasets of the same individual. As expected under recombination scenario, we observed that fractions of SNP pairs passing the four-gamete test are from one to four orders of magnitude larger when testing haplotypes of different individuals than the corresponding fractions detected when comparing phased haplotypes recovered for the same individual from different sets of reads (Supplementary Figs. 15 and 16).

Please see Supplementary Figures 12, 15, 16 and Supplementary Note 2 for details.

(i) “the decay persists in subsets of polymorphic loci covered by long blocks of collinear genes” – that doesn’t reassure me, why should phasing be more reliable for those cases?

Because regions involved in long blocks of collinear genes are less likely to be misassembled paralogous regions. Therefore, the chance of confusing haplotypes with paralogous loci in such regions is reduced and alignment of reads is accordingly expected to be more reliable. We have expanded the relevant part of the text. Now it reads as follows: “the decay persists in subsets of polymorphic loci covered by long blocks of collinear genes, making this explanation unlikely. Existence of two haplotypes in the L1 genome in these subsets of loci is additionally confirmed by the presence of highly similar genes collinear between the two putative haplotypes.**”**

(ii) “we filtered the phased haplotypes aggressively” – not enough information on this part to persuade me either way, I don’t have a strong prior notion of whether aggressive filtering should reduce possibility for the relevant artefacts here or not.

We now make use of the newly obtained multiple haplotype assemblies (HiSeq-, MiSeq- and PacBio-based) to validate this approach. We now measure the phasing error rate as the fraction of SNP pairs exhibiting inconsistent haplotype phasing recovered from different data types for the same individual (HiSeq vs. MiSeq and HiSeq vs. PacBio). We show that this error rate is indeed reduced by filtering. For example, the fraction of haploid contigs with inconsistent phasing between HiSeq and MiSeq data for L1 was 0.023 for raw phased data (Supplementary Table 13). This fraction was reduced to 0.00073 by exclusion of phased blocks encompassing ‘conflicting’ pairs of SNP (the filtering applied to the main phased dataset 1), and further dropped to 0 after additional filtering based on switch and mismatch quality scores (applied to the phased dataset 2).

Please see Supplementary Table 13 and Supplementary Note 2 for details.

(iii) Your first unphased method seems the neatest part to get at this – I’d suggest to make this higher profile and cut the weaker alternatives – but even then, with the number of SNPs meeting those criteria, it seems plausible that alternative genotypes of 0/0 and 1/1 at more distant SNPs could reflect assembly artefacts, e.g. alternative pieces of similar DNA being alternatively assembled into that position in different individuals. Gene conversion is another possible contributor to this pattern, as seems to be acknowledged on line 127.

Thank you for the positive evaluation of this method. We agree that although its results are less likely to be affected by phasing artifacts, the LD decay observed in this analysis could be particularly sensitive to gene conversion, as it only considers sites homozygous in all 8 individuals. In contrast to this unphased method, the four-gamete test dealing with pairs of heterozygous sites is expected to be less affected by conversion events. Therefore, we need both methods. Moreover, we now show that the phasing in the phased datasets is accurate, and that the results of the four-gamete test stay almost unaffected by the type of reads used for phasing and by the stringency of filtering (see Supplementary Figs. 15-16, Supplementary Note 2 and replies to the previous comments). Therefore, we think that the four-gamete test deserves more emphasis than the unphased methods.

(iv) I don't understand your second unphased method, having read the supplementary methods a few times. Do you really need this even? For all the methods you use, I suggest adding a figure to the supplementary illustrating the rationale for each method – very hard to unpack all of this from the words.

Unlike our first unphased method (which we, as far as we know, invented), the second method is rather standard. It is a part of PLINK, a set of tools commonly used in population genomics studies. We now describe it in more detail in the corresponding section of the supplementary methods: "The second approach to inferring the rate of LD decay from the unphased genotypic SNP data relies on calculation of squared correlation coefficients between genotypes using VCFtools[13] command --geno-r2 (https://vcftools.github.io/man_latest.html). This command computes the same unphased LD measure as PLINK[16]. Namely, for each pair of SNPs it gives the squared correlation coefficient between numbers of non-reference variants (which could be 0, 1 or 2) at two corresponding sites in the considered individuals. Note that each genotyped genomic site could be represented by a vector of length n, where n is equal to the number of individuals and the i-th element of a vector represents a genotype (0, 1 or 2) of the i-th individual. Therefore, correlation coefficients could be computed for a pair of sites, each encoded as a vector of genotypes." This analysis has been updated, now as in the rest of LD related analyses only SNPs with minor allele count of at least 4 are utilized.

(v) It might be reassuring if the same phasing were consistently recovered from multiple independent replicates of the same genome. Were any of the "clonal" populations sequenced more than once, and if so, how consistent was the phasing? Alternatively, is it possible to test any of the phasing by long-range amplification and cloning methods?

Following this suggestion, we now estimate phasing accuracy – please see our responses above and Supplementary Note 2.

(4) Gene conversion. The argument in fig 2d and e and lines 137-138 assumes that recurrent mutations are rare. If recurrent mutations are not rare, the 4 genotypes in fig. 2e can be produced from an ancestor by just 2 mutation events – especially if most mutations are transitions, the plausibility of repeat mutations does not seem so unlikely. How the fraction of "recombinant" pairs changes with distance is the pertinent part, because there is no reason for the multiple mutation mechanism to vary with distance. I eventually found this explained clearly in the supplementary (top page 11) – would be useful to bring this reasoning to the main text, and tone down/reword the part about recurrent mutations. Given the range of exotic mechanisms being considered here – e.g. an entirely new mechanism of transformational recombination for animals – assuming a lack of recurrent mutations seems like a weak argument and you don't need it.

As suggested, we now tone down the part of the argument pertinent to recurrent mutations, and stress that our claim for recombination rests on the increase in the fraction of recombinant SNP pairs with distance.

(5) Hardy-Weinberg test. This is implied by the flow from line 150 to be a test of mitotic recombination within individuals – please spell out to the reader exactly what would be expected under that mechanism versus between-individual recombination. What is your statistical power to reject the null hypothesis at each locus with 8 individuals sampled? Line 164-166, please expand on this explanation – how precise would this matching need to be and of what kind? How "unlikely" is it that the relevant parameters are constrained in the manner required, by some genetic or population-genetic feedback mechanism? Plausibility arguments are not so relevant when your preferred explanation involves an entirely new transformation mechanism for animals. Are there any conceivable assembly or sequencing artefacts to explain the HW results? These are important arguments for evaluating your alternatives.

We now expanded the section on deviation from the Hardy-Weinberg equilibrium significantly. Most importantly, using simulations, we now show that the low absolute values of inbreeding coefficient F_{IS} that we observe (F_{IS}~0) are statistically incompatible with strict clonality, and set the lower limit on the fraction of reproduction events involving genetic exchange (please see main text and Fig. 3). Furthermore, as requested by the Reviewer, we now develop a theory for the interaction of mutation and conversion under strict clonality. We show that even with conversion, it is impossible to obtain F_{IS}~0 under any

realistic parameters, again rejecting strict clonality (please see Supplementary Note 5).

Mitotic recombination (i.e. crossing over between homologs within an individual) seems incompletely explored. Is it possible that recombination between homologs within clonal lineages plus gene conversion could alone explain the LD results and four-gamete test? If so, make it clear that the claim of between-individual recombination rests heavily on the interpretation of the H-W results and draws no direct support from LD decay. How far can we get in explaining the data if we posit reciprocal intragenomic crossing over between homologs with preservation of heterozygosity? This seems more parsimonious than either males or transformation, because it would use the same mechanisms as gene conversion, which we know occurs.

Indeed, the LD results and the four-gamete test results can be explained by mitotic recombination, as acknowledged in the original submission. We now clarify this. The claim for between-individual recombination rests on the Hardy-Weinberg results, the triallelic SNPs, and the haplotype trees. We now show our logic schematically in Fig. 7.

(6) The triallelic SNPs part would benefit from a clearer explanation: important detail is relegated to supplementary or not stated. Something like: “a SNP with 3 alleles requires at least 1 mutation in above a 2 allele SNP, hence we estimate the probability of an additional mutation during the history of the sample as p = N3/(N2+N3). Under a mutational mechanism alone, a triallelic SNP with all 3 heterozygotes present requires yet another additional mutation. Hence, based on the probability p and the number of triallelic SNPs, we expect to observe p*N3 = 83.5 SNPs with all 3 heterozygotes, but in fact we observe 1839”. A diagram might also help. At present, line 176 in the main text reads like an assertion that recurrent mutation is rare, rather than a calculation, which is a much stronger case.
It might help to place the results on triallelic SNPs in context by comparing them with other animals (e.g. Drosophila, humans). I think the incidence of triallelic SNPs reported for rotifers here (0.0086) is several orders of magnitude lower than equivalent rates estimated in clearly sexual species. Perhaps there is some methodological reason for this, but if not it seems to reduce the force of the argument that the incidence of triallelic SNPs is so much higher than expected that it cannot arise from recurrent mutations.

Thank you for you suggestion for clarifying the text, which we gratefully employ.
Regarding the incidence of triallelic SNPs: it is dependent on many things, including (of course) the number of sampled individuals as well the mutation rates, effective population sizes,

allele frequency spectra, and the variability in mutation and selection between genomic regions. All these factors differ between *Drosophila*, humans and rotifer, so differences between datasets are perhaps unsurprising. Still, the fraction of triallelic sites among all variable sites in individuals L4-L11 (0.0086) is comparable to that in *Drosophila* (0.0186; Table 1 of Seplyarskiy et al., 2012, https://academic.oup.com/mbe/article/29/8/1943/1043688).

We stress that we do not use the incidence of triallelic sites as an argument in favour of genetic exchange in *A. vaga.* It is clear that triallelic sites *per se* could emerge under asexuality as well as under normal sexual reproduction. Rather, it is the high incidence of triallelic sites represented by all three heterozygous genotypes that lends support to genetic exchange.

(7) Gene trees (lines 198-220, figure 4). This part is least convincing. The gene trees look very odd in a few ways and it is not obvious they fit the transformation mechanism any better than the Oenothera mechanism (I agree they do not fit that):
(i) There is no signal of an underlying clonal structure to these populations, which seems very surprising for a transformation-type mechanism. It would have to occur at an exceptionally high rate. You estimated 1 transformation event per generation – is this consistent with observing no clonal structure in the gene trees? Even in bacterial species with readily measurable rates of natural competence and homologous recombination, there is typically substantial genome-wide linkage disequilibrium, as expected for organisms whose reproduction is strictly clonal even genetic transmission is not. I note a recent interesting preprint (https://www.biorxiv.org/content/10.1101/385336v1), which concludes that bacterial panmixia is rare and unstable, and evidence of clonal structure is seldom absent.
(ii) Another odd feature is that quite often there are two alleles from different individuals that are not just similar but identical. In many cases: Fig. 4 (a) (b), (c), Table 1, these putatively allele-sharing individuals were collected 550km apart. . What is the probability of observing so many cases of identity for this small sample of individuals, given the considerable phylogenetic “scatter” we see among homologs within each locality and within each individual? It would seem to be rather low by the proposed mechanisms. Some thoughts of things to check: index-hopping or contamination between libraries, were relevant clones multiplexed together; are there any alleles present in L1 also found in the L4 ‘species’, which might indicate index hopping or contamination?

In the updated version of the main text and below, we no longer regard transformation as the most likely mechanism of genetic exchange in bdelloids.

Regarding index hopping, indeed, some of the relevant clones were demultiplexed together. However, there are several

lines of evidence against index-hopping as an explanation for the observed incongruence.

1. As stated on the illumina website, the Illumina sequencers of the type used in the current study (Illumina Illumina HiSeq 2000 and 2500) typically "have rates of index hopping ≤ 1%", which is lower than that of more recent models (e.g. HiSeq 3000/HiSeq 4000) (https://www.illumina.com/content/dam/illumina-marketing/documents/products/whitepapers/index-hopping-white-paper-770-2017-004.pdf, https://emea.illumina.com/science/education/minimizing-index-hopping.html). Hence, the instances of index hopping are expected to result in the presence of a haplotype supported by just a small fraction of reads. For most individuals, the genomic coverage of the haploid sub-assembly is ~100X; therefore, 1% of erroneously demultiplexed reads would translate to ~1 read supporting the 'minor' haplotype. We visually inspected the read alignments in many of the regions identified as incongruent, and observed no cases when the signal of incongruence was associated with the presence of such poorly supported haplotypes; in all cases, it was supported by many more reads.

2. To formally show that the signal of incongruence is not explained by index hopping, we now tested for incongruence after applying an additional very strict filtering step. The spurious heterozygous sites arising from index hopping are expected to have very weak support from the reads for one of the alleles. Therefore, prior to looking for the segments exhibiting incongruence, we excluded sites called as heterozygous in any individual but with one of the two alleles supported by fewer than 30% of the aligned reads in this individual. (As an additional precaution, we also excluded sites that were called as homozygous in an individual despite some reads supporting an alternative nucleotide, as such a pattern may be indicative of index hopping). The remaining sites cannot be affected by index hopping. The number of genomic regions that survived this stringent filtering was low, but the patterns observed in the main analysis persisted, with (i) multiple segments displaying haplotype incongruence, and (ii) at least two different patterns of incongruence for each individual (Supplementary Table 18 and Methods).

3. Finally, we have now expanded the analysis of incongruence to all individuals L1-L11. The total number of genomic segments phased in all individuals from both clusters and carrying at least 15 non-singleton SNPs is rather low (n=152). However, even in this small dataset, we detected several cases of incongruent grouping of haplotypes for individuals from the small cluster (L1-L3, Supplementary Table 21). Intriguingly, in all such cases when the two haplotypes (H1 and H2) of an individual from the small cluster had unambiguous closest counterparts in different individuals, one haplotype (H1) was always clustered with a haplotype from another individual from the small cluster,

and the other haplotype (H2), with a haplotype from the large cluster. This pattern was observed for all three individuals of the small cluster (L1, L2 and L3), although the total numbers of segments with detected incongruence for L1, L2 and L3 were low (5, 2 and 4 respectively). This result cannot be explained by index-hopping, as (i) neither L1 nor L2 were demultiplexed with the individuals from the large cluster; and (ii) although L3 was demultiplexed with L6, L7 and L10, neither of the two patterns of incongruence detected for L3 (L1-L4 and L2-L9) involved these individuals (Supplementary Table 21). Similarly, for several individuals from the large cluster (L6, L9, L10 and L11), we detected cases when their haplotypes clustered with individuals from different clusters (Supplementary Table 21). Out of these 4 individuals, just one (L10) was demultiplexed with the relevant clone from the small cluster. In summary, our results could not be explained by index hopping. The patterns of haplotype phylogenies observed for 3 individuals of the small cluster appear to be explained by hybrid origin of this cluster (please see main text for details).

Our results are also unlikely to be associated with contamination based on the analysis of triallelic SNPs with three heterozygous genotypes. This is discussed in the main text and Supplementary Note 6: "To confirm that the sites carrying all three possible heterozygous genotypes are not likely to be due to cross-sample contamination, we separately considered those sites carrying all three heterozygotes among the individuals L4-L11 that harbor only one private heterozygous genotype (n = 607). That is, we retained a site for the analysis if the least frequent of the three heterozygous genotypes was present in a single individual with the next frequent genotype present at least in two individuals.
Such private heterozygous genotypes possessed by a single individual are most likely to stem from contamination. Moreover, should contamination be the case, we would expect to see a skewed distribution of per individual numbers of such private heterozygous sites with the samples resulting from contamination carrying disproportionally more private heterozygotes.
Following this logic, we analyzed how the 607 private heterozygous genotypes are distributed among different individuals. For this purpose, for each individual, we tabulated the total number of sites with the least frequent heterozygous genotype private to this individual.
Contrary to what would be expected under contamination, we observed that the resulting numbers of private heterozygous sites were very similar across different individuals".

(iii) In contrast, no cases of two identical alleles from the same individual are observed, indeed the 2 alleles within an individual look – by eye – to be over-dispersed in the gene trees, i.e. never particularly close. Again, this does not seem so likely based on the proposed

mechanism and our prior information about rates of gene conversion. This made me worried about paralogy. It would be worth blasting each allele back into all genomes just to check they are not present as additional paralogs lost somehow in the construction of your haplotype blocks and filtering.

To formally address the issue of heterozygosity within individuals, we have now conducted a sliding windows analysis of heterozygosity in different individuals (Supplementary Fig. 8). This plot shows that in individuals L4-L11 there is a minor peak at very high similarity (~0% heterozygosity) consistent with the presence of regions having undergone gene conversion (or inbreeding). The reason why such regions are not found among phased regions subjected to the analysis of incongruence is that the phasing algorithm which we employed assigns to haplotypes only those SNPs at which the considered individual is heterozygous. As described in the Methods section, for regions with some heterozygous SNPs, we overcome this by complementing the phased haplotype blocks with the data on homozygous SNPs embedded within the phased blocks. However, regions of extended homozygosity would not be considered by HapCUT2 in the first place, as there are no heterozygous SNPs which could be subjected to phasing. As we require a region to be simultaneously phased in all 8 individuals L4-L11 to be included in the analysis of incongruence, regions devoid of heterozygous SNPs in any individual would be automatically omitted. This is OK for the purposes of our analysis: as we focused on detection of incongruence associated with genetic exchange, we were not interested in cases when a haplotype had the closest counterpart in the same individual. The procedure used to detect cases of potential incongruence is detailed in the Methods section.

To show that the analysis of incongruence is not affected by the presence of paralogs, we now blasted each considered segment back into the diploid assembly of L1, and removed segments for which alleles could be potentially confused with paralogs (assemblies obtained from available HiSeq reads for individuals L4-L11 were very fragmented with N50 in the range 1,600-3,400 bp and as such did not suit the purposes of paralogs identification).

For each allele of each considered segment (n=434), we tabulated the number of unique hits in the L1 diploid assembly. The median number of hits per allele was equal to 4: usually there were 2 very similar hits (≥90% nucleotide identity) corresponding to two haplotypes and 2 hits with lower identity (~65-85%) likely representing paralogs. However, some segments had hits to a substantially larger number of contigs. In most such cases there were two highly similar full-length hits corresponding to haplotypes and a number of short low-identity hits covering a small portion of a segment. We note that these cases are not likely to cause false inference of incongruence. First, reads from

diverged regions are not normally aligned with bowtie2. Second, paralogous alignments usually have low mapping quality scores and we filtered out alignments with MAPQ < 20 prior to SNP calling. Third, within-individual interallelic distances as assessed from reconstructed haplotypes were low (within 2.5%) which is what is expected for the distances separating the two haplotypes but not paralogs.

To ensure that genomic segments containing sequences with a large number of diverged copies in the *A. vaga* genome do not introduce a spurious signal of incongruence, we have now excluded segments with more than 10 hits in the diploid assembly regardless of the hit identity level. In the remaining set, an allele had an average of 2.07 high-identity (≥90%) hits and an average of 3.02 low-identity (<90%) hits. To get rid of regions with highly similar paralogs that could be mistaken for haplotypes, we further excluded segments with alleles harboring more than 2 high-identity hits. After these steps we were left with 303 out of original 434 phased segments. The results of incongruence analysis in L4-L11 included in the revised version of the manuscript are based on these 303 filtered segments (segments used to infer incongruence in L1-L11 (n=152) were also subjected to the analogous filtering). Although some of the regions reported as incongruent in L4-L11 in the first version of the manuscript were filtered out as a result of the above-described procedure, the conclusions of this analysis remained unchanged.

(iv) It would be useful to have more information on the phased genomic sequences that emerged from filtering used for this analysis – do they contain genes, introns, are the SNPs in coding regions? If the variation is functional, certain combinations could represent selectively favoured genotypes that would attract multiple clones independently without implying genetic exchange.

Incongruence implies different individuals carrying near-identical haplotypes. In the absence of genetic exchange, this would imply that (i) there were multiple independent mutation events affecting exactly the same sites in different lineages in exactly the same way and (ii) there were few mutations affecting the remaining sites. It is hard to quantify the exact probability of this, but this appears to be very unlikely, and the high bootstrap support values show that the observed incongruence is not likely due to chance. More generally, parallel adaptation is quite rarely achieved by independent origin or identical mutations (one of the most well-known examples in humans is the genetic adaptation conferring the ability to digest lactose in adults, e.g. Jones et al., https://www.cell.com/ajhg/fulltext/S0002-9297(13)00326-1).

As suggested, we have now annotated the SNPs residing within the segments identified as incongruent in L4-L11 (Supplementary Table 19). The SNPs belonging to the incongruent

segments fell into many different categories including synonymous, missense, intronic, intergenic and stop gain variants. Usually a segment harbored SNPs belonging to several functional categories. This argues against selectively favoured homoplasies, as we would not expect SNPs in introns or synonymous sites to be under strong selection and to attract multiple clones.

We have also added a new analysis aimed at characterization of phased segments inferred as incongruent (Supplementary Note 8). If regions inferred as incongruent emerged due to homoplasies affecting functional sites, we would expect such regions to be enriched in protein-coding genes. Contrary to this expectation, we now show that segments identified as incongruent among L4-L11 (n=52) do not display an increased probability to overlap with protein-coding genes compared to the genomic background (Supplementary Note 8 and main text). Therefore, the signal of incongruence does not appear to be driven by functional variants.

(v) It could be made clearer in the caption to Figure 4 and elsewhere that these are not a typical sample of trees for randomly selected nuclear markers, but the result of an intensive filtering process designed to locate and enrich for cases of apparent incongruence (lines 784-812). It would be good to estimate what the 'expected' rate might be for detecting incongruence as extreme as this, given the number of regions and permutations examined.

This is now clarified. It is not straightforward to estimate the 'expected' rate of incongruence, given the many factors that may contribute to this value. Still, 79 out of 303 (unfiltered!) regions demonstrating incongruence, including 52 with high bootstrap support for incongruence, seems way too high for errors in phylogenetic reconstruction.

(8) In the final parts, you only compare 2 models: transformation and Oenothera-type meiosis. It is clear from the LD analysis that the present results cannot be explained by the Oenothera model; that point could be made more succinctly. The bigger challenge (perhaps not just for the authors here) is to explain how the results of Signorovitch et al. (2015) can be reconciled with any mechanism proposed in the present manuscript.

What about the 3rd possibility of regular meiosis – are your results explicable by that mechanism or not? What frequency of meiosis relative to apomixis (e.g. 1 sexual generation per 10000 asexual) would be consistent with observed levels of LD decay? I think you have relevant calculations in the supplementary in terms of population-scaled recombination rate.

We have now substantially revised the discussion of potential evolutionary scenarios underlying the observed patterns. The updated analysis of incongruence expanded to all 11 individuals L1-L11 revealed patterns that seem to be incompatible with transformation alone. Instead, taken together, signatures of recombination and observed incongruence could be most parsimoniously explained by conventional meiosis. The frequency of meiosis needed to alone explain the LD decay is now estimated (please see main text).

(9) For repeatability, you need to be more precise with your taxonomy (line 91 and Methods). You talk about Adineta vaga, but Adineta vaga covers a wide array of diversity and different cryptic species. If used in a strict sense, it ought to refer to the original type described by Davis (https://www.researchgate.net/publication/318711223_WHAT_IS_ADINETA_VAGA_DAVIS_1873), or for molecular purposes it might be defined based on the reference strain sequenced by Flot et al. 2013, which is clearly distinct from the individuals here. In either case, I think your animals are better designated as Adineta sp. (perhaps Adineta sp. 'L1-3' and sp. 'L4-11', depending how distinct the two are by formal analysis).
I'm happy that you took diversity into account for your population analyses and focused on one main cluster. But a tree of your isolates for the cox1 marker (the standard marker for bdelloid DNA taxonomy), with reference sequences from the literature (including the A. vaga 2013 genome clone), would be very useful – more useful than figure 1c or Table S5. That is important information for people to be able to repeat your work and compare the relationships among your clones with those among clones in other salient datasets (e.g. Signorovitch et al. 2015 Genetics; Fontaneto et al. 2011 Hydrobiologia 662:27). You should also report more specifically which collection locality (i.e. tree and patch) each clone came from (with GPS coordinates), and particularly the distance between trees.

We now add the tree of our isolates based on the cox1 marker. In this tree, our isolates L1-L11 are clustered with reference isolates identified as *A. vaga* by other groups and not with non-*A. vaga Adineta* species (Supplementary Figs. 1-3 and Supplementary Note 1). Specifically, based on cox1 phylogeny, our isolates are very similar to some UK and Tanzania *A. vaga* isolates. As such, our taxonomy originally based on morphological criteria is justified.

We have also added a supplementary table with sampling locations (Supplementary Table 1).

Additional comments:

Line 13 – both darwinulids and Timema have documented males, and so are maybe not such reliable examples of long-lived asexuals.

We agree, and have removed the corresponding sentence from the abstract. Darwinulids and Timema are still included in the list of putatively ancient asexual groups listed in the introduction. It is indeed hard to come up with an example of an anciently asexual lineage whose asexual status has not been questioned. Notably, there is a debate with regard to whether several males detected in darwinulids are sufficient to refute obligate asexuality for this group. For example, Martens and Schon, 2008 (https://www.nature.com/articles/453587b) argue that: "Sex in darwinulids has not been conclusively demonstrated. The three males in a single species of the darwinulid genus *Vestalenula*, found among thousands of females, did not have identifiable sperm (nor did any of the investigated females), suggesting that these specimens are non-functional atavisms." We believe that it is out of the scope of our manuscript to delve into the details of this and other relevant discussions, which are indeed very interesting to follow.

Line 15 – more like 60Mya? Tang et al. 2014. Evolution.

Thank you, we now have cited this reference and corrected the age estimate.

Line 17 – Remove reference to palindromes in abstract, or if refer to, needs to say something like "prior evidence from genomes not confirmed by subsequent work", to illustrate that there is no real evidence of lack of meiosis from current genome evidence.

Corrected.

Line 22 and 66 – saying one of the studies is controversial or has been questioned does not give an accurate impression here. Clear evidence was presented in a refereed paper that the original results were "artifacts of experimental error" arising from accidental contamination between tubes. The editors and reviewers of the journal in question accepted and published this result as "clear evidence that the data and findings of Debortoli et al. (2016) are unreliable". No further debate on the matter has appeared in other peer-reviewed outlets since. That's clear evidence against the original interpretation, not really any controversy. The description is worth clarifying both in the introduction and in the abstract too, as many people only read this.

We have now clarified the corresponding part of the manuscript.

Line 82- Important to report whether you find any evidence for lack of homology and palindromes or not – I presume not from what is stated, but it needs emphasizing.

We now revise the corresponding section of the manuscript as follows: "Intriguingly, in contrast with the first published genome of *A. vaga* where multiple instances of collinear regions residing on the same contig and organised as palindromes were detected, we identified only a single palindrome. Although our relatively low N50 value does not allow a detailed analysis, this finding is in line with the results of Nowell *et al*. who have not detected palindromes in the genome assembly of another *Adineta* species, *Adineta riccae*.".

Line 127. Why "finally" here – there are still other alternatives to consider after here.

Corrected.

Line 202: Have the authors read in detail the piece of work cited as reference 36? Having previously done so myself at some length, I do not think it adds any substance to the point and does not merit mention in the same sentence as references 17, 34 or 35. It contains no evidence that reported phenotypic effects were caused by DNA uptake or transformation, as opposed to other consequences of the experimental manipulation.

We no longer cite this reference.

Line 203: Reference 9 is not the most appropriate citation for the hypothesis that intra-specific genetic exchanges might occur in bdelloids. That hypothesis was first put forward clearly and succinctly in Reference 34, which has the further advantage that its data and findings are reliable. Reference 9 has been found to supply "no credible evidence to address that question" (c.f. Reference 10).

Now Gladyshev et al. (previously, ref. 34) is cited at this place.

Line 249 to 256 on GC content seems speculative and not to add much – I would move that to supplementary and make more space for important parts. The same arguments would apply if the recombination were intragenomic or linked to gene conversion, so this is not a powerful prediction.

We have now moved the relevant sentences to supplementary information, as suggested.

Line 257. "Despite near certain lack of meiosis". What is the evidence for this? Apart from lack of males, I don't think you have any – unless you can tease this out more from the analysis. Same again in the

Supplement (p.24): “reported lack of homologous chromosomes in A. vaga genome virtually ruled out conventional sexual reproduction in bdelloid rotifers”.

We agree that there is now not much evidence against conventional meiosis left. Moreover, our new analyses on haplotype trees in 11 individuals appear to be consistent with conventional meiosis, but not with transformation as a sole mechanism of genetic exchange. Therefore, we no longer claim lack of meiosis.

Line 259. Worth clarifying what you mean by ‘panmixis’. This doesn’t mean equivalent to a fully outcrossing sexual population, but that any individual in your cluster L4-L11 can transfer genes to any other member of that cluster at the calculated rate? Be careful with terminology – if there is no meiosis, then the “mode of reproduction” is clonal/asexual, but with transformation at some unspecified life-history phase (oogenesis? desiccation?). If so, A. vaga is not amphimictic, as that term is commonly understood in animals. Overall, you need to be more critical at how the rate of recombination compares to what you’d see in a fully outcrossing population, which is how people will interpret this section and the word “panmixis”.
To further address this, it might be worth adding a brief comparison. How does this rate of recombination/LD decay compare to better-studied models such as Drosophila?

We agree that “panmixis” is an ambiguous term in the context of transformation, although it has been used in this context in previous works (e.g. Maynard Smith et al., 1993 https://www.pnas.org/content/90/10/4384). We no longer propose transformation as the mechanism of choice for genetic exchange. We now mention that the rapid LD decay is similar to that observed in *Drosophila melanogaster.* Note that it does not imply that *A. vaga* and *D. melanogaster* experience the same rate of recombination per base pair per generation. As now discussed in the main text, rate of LD decay depends not only on the per generation rate of recombination (c) but also on the effective population size (N_e). Therefore, the same rate of LD decay can arise in populations with different per generation rates of recombination (and meiosis) but with the similar parameter $4N_ec$ (commonly referred to as population-scaled recombination rate).

Line 461: The method of rearing a clonal lineage in the lab is partly at odds with the goal of determining bdelloids’ mode of reproduction. If the LD and HW results imply high rates of inter-individual recombination, might the single individual transferred into the initial dish have been a fertilised female, in which case she would have produced a shuffled mixture of outcrossed recombinant F1 siblings, each of which would start a different clonal lineage in the same lab

dish, where males apparently are no longer produced. That would make reliable phasing impossible and could produce downstream results that might look like evidence of transformation, perhaps including some of the patterns that were excluded from the analysed data as likely artefacts of “PCR template switches” (e.g. repeatedly finding four different haplotypes in a single “individual” where only two are possible, Supplemental, p.7).

If our cultures were in fact started from fertilized females, we would expect many regions for which 4, rather than 2, well-supported distinct haplotypes would be present. However, we very rarely see more than 2 well-supported distinct haplotypes. For example, for L1 out of total assessed 5,882,563 pairs of heterozygous sites simultaneously covered by paired-end reads only 82 pairs were represented by four different haplotypes each supported by no less than two reads. For L4 the corresponding numbers are 167 out of 1,021,641; for L6 244 out of 1,263,625 etc. This is in contrast to what we observe in comparison of two different individuals where we find many pairs of heterozygous sites represented by all four possible combinations.

Line 470: “the species identity of cultures L1-L11 was additionally confirmed...” Based on Table S5, it is not clear that L1-3 and L4-11 actually share the same species identity; ideally you would conduct a formal analysis to determine whether they ought to be designated as different species, perhaps using a larger dataset to generate trees and conspecificity matrices (e.g. mtCO1 or the nuclear markers reported by Debortoli et al. 2016). If the two are different species, then it is not strictly accurate to talk about ‘population structure’ when differentiating them. There might also be some questions about using a reference genome from L1 to map the reads from L4-11, although the methods appear to have been carefully implemented and checked to show the results are robust to this.

We have now complemented the manuscript with the tree of our isolates for the cox1 marker (Supplementary Figs. 1-3 and Supplementary Note 1). On this tree, our isolates L1-L11 are clustered with reference isolates identified as *A. vaga* by other groups and not with non-*A. vaga Adineta* species. Moreover, cox1 phylogeny does not support the presence of two genetic clusters among our isolates inferred from the nuclear SNPs, rejecting the hypothesis that the two clusters correspond to two isolated species. The analysis of haplotypes from all 11 individuals (L1-L11) provides an explanation for this observation: the three individuals of the small cluster (L1-L3) are likely to be of hybrid origin (please see the main text of the manuscript).

Supplement, pages 17-18. How did you estimate the effective size of “the A. vaga population” as 10^6? This could be rather low. First, see

Section 9 above- what exactly is “the bdelloid rotifer A. vaga” (Line 268) and how do we delineate it into species and populations? More specifically, I have seen unpublished data suggesting that the number of individual rotifers in the genus Adineta on a single moss-covered tree at a single timepoint is between 10^4 and 10^5. The total number of bdelloid rotifers annually washed by rain from a single tree into a single square meter of soil was recently estimated at 1.2 x 10^6 (https://doi.org/10.1186/s12898-018-0198-4). What happens to the calculations in Sections XIV and XV if Ne is increased by several orders of magnitude? Returning to Figure 4, with these sorts of population sizes, what rates of transformation would be required to give a measurable probability of observing any alleles that are identical by transformation between
two individuals from a sample of eight, collected 550km apart (e.g. Fig. 4a L4.2/11.2; Fig. 4b L4.2/L5.1; Fig. 4c L5.1/L7.2, L5.2/L8.1)?

Indeed, if N_e is increased by several orders of magnitude, estimates of transformation rate would become several orders of magnitude smaller.

Estimates of N_e could be derived from the levels of heterozygosity based on equation $\theta=4N_e\mu$, where θ stands for the expected level of heterozygosity and μ for the mutation rate. We obtained maximum-likelihood estimates of the expected heterozygosity (θ) for each individual independently using the approach proposed by M. Lynch (https://www.ncbi.nlm.nih.gov/pubmed/18725384) and implemented in the program mlRho (https://www.ncbi.nlm.nih.gov/pubmed/20331786).

Estimates of θ for the individuals belonging to the large cluster (L4-L11) were on the order of 10^{-2} (ranging from 0.00722 to 0.0094, with the average value equal to 0.00858, Supplementary Table 10).

This roughly gives us $\theta=10^{-2}=4N_e\mu$.

Estimates of mutation rates in different species are usually in range 10^{-9}-10^{-8}. E.g. two studies employing different methods estimate an average mutation rate in human germline of 1.28×10^{-8} and 2.2×10^{-8} per bp per generation respectively https://science.sciencemag.org/content/349/6255/1478.long. Recent estimates of mutation rate in *Daphnia pulex* and *Drosophila melanogaster* are 5.69×10^{-9} and 5.17×10^{-9} per bp per generation respectively http://www.genetics.org/content/206/1/315.

Assuming μ in *Adineta vaga* is 10^{-8}, gives us $\theta = 10^{-2} = 4N_e \times 10^{-8}$ which translates to N_e = ~250,000. If μ is 10^{-9}, estimate of N_e would become ~2,500,000. Even if mutation rate in *A. vaga* is extremely low of the order 10^{-10}, this would correspond to N_e of ~25,000,000. However, such low rates of mutation have been reported previously only in some unicellular eukaryotes (*Saccharomyces cerevisiae* or *Chlamydomonas reinhardtii*), but never in multicellular species (for example, see Lynch et al. 2016, https://www.nature.com/articles/nrg.2016.104).

In summary, there is no reason to suspect that N_e of the studied population is several orders of magnitude larger than 10^6. It is important to note that our estimates of N_e do not contradict the observations of enormous numbers of individual bdelloid rotifers on single trees. This is because effective population size (N_e) in the majority of studied species is usually found to be significantly reduced compared to the census size (N) (for example, see https://www.nature.com/articles/nrg2526/tables/1). E.g. N_e of the human population is estimated to be around only 10,000.
N_e reflects the amount of genetic drift in population and does not have to reflect the actual number of the individuals in current populations. However, we note that accurate estimates of effective population size in *A. vaga* would require precise estimates of mutation rates in this species. We have expanded the corresponding section of the text accordingly.

Another thought: Did you run your tests on the mitochondrial genome? If so, does that show similar evidence for the decline in LD observed here, or not – that might help further address meiosis versus ameiotic mechanisms (which arguably should apply to mtDNA as well).

We did not run the tests on the mitochondrial genome. This is an interesting subject for further study. Unfortunately, the recombination or lack thereof in mtDNA tells little about the mode of reproduction in the nuclear genome: there are both species with and without mitochondrial recombination among, e.g., strictly sexual vertebrates (for example Tsaousis et al., https://www.ncbi.nlm.nih.gov/pubmed/15647518).

Supplementary note – this seems to restate the manuscript, perhaps with clearer explanations of some of the logic employed – I suggest reworking so the main text makes these points clearly enough that a re-explanation in supplementary is not required.

Thank you for this suggestion; we now move some of the text from the old Supplementary Note to the main text.

There appears to be no evidence that the two individuals (L5, L11)

collected 550km from Moscow show different patterns of incongruence or putative allele sharing than the six individuals collected from Moscow. This seems surprising even given the potentially rapid dispersal abilities of bdelloid rotifers; recent transfer or sex ought to be more evident among individuals collected closer together versus 550km apart. What explanations for the apparent incongruence can we turn to that would take no respect of physical geography?

Here we repeat our reply to the similar question raised by the first Reviewer:

"First, prior publications have reported that genetic relatedness between *Adineta* individuals is often not associated with distances between sampling locations. For example, Fig. 1 from the paper (Fontaneto et al., 2011) https://link.springer.com/article/10.1007/s10750-010-0481-7 shows that some *A. vaga* individuals from the UK are more closely related to Tanzania individuals sampled from similar habitats than to other UK individuals. Our study recapitulated a similar pattern – although on a smaller geographical scale – with several individuals collected in the Moscow region (L4 and L6-L10) being more closely related to individuals L5 and L11 collected in the Kostroma region, 550 km to the NE, than to the three other individuals L1-L3 sampled from the Moscow region (Fig. 1c).

Second, an even more peculiar feature has been reported by Signorovitch et al. (Genetics 2015) who observed an individual of a bdelloid rotifer *Macrotrachela quadricornifera* sharing alleles with individuals collected from sites located more than 240 km away. Moreover, they found a second-order clustering of two US individuals with an individual from Milan, Italy (more than 6,000 km away).

Of note, the small sample size (8 individuals) is likely to limit our ability to detect fine-scale differences in probability of genetic exchange between individuals from remote locations. Perhaps increasing the sample size by a factor of 10 or more could reveal subtle differences in likelihoods of recombination that should exist between individuals collected from different sites."

Incidentally, I cannot reconcile the data in Table 1 with the summary in Table S12. How can it be that L4 shares zero patterns of incongruence with L6 in Table S12, when they share three different patterns of incongruence across 6 different segments in the first line of Table 1 (with respect to L7 x 2, L8 x 1 and L11 x 3)?

Tim Barraclough

We admit that the reference to old Table S12 (revised Table S17) could be misleading, and we did our best to clarify the logic in the revision. It should be read as follows: for each pair of individuals (Ind1-Ind2) it shows the numbers of cases (segments) when there existed a third individual most closely related to Ind1 with respect to one haplotype and most closely related to Ind2 with respect to the other haplotype. (However, it does not mean that Ind1 is close to Ind2 in these segments.)

That is, for each pattern of incongruence listed in the rightmost column of the old Table 1, Table S12 gave the total number of occurrences in different individuals. For example, for the pair L6-L7, there were 4 segments where a third individual existed that clustered with L6 based on one haplotype and with L7 based on the other haplotype (2 segments in L4, 1 segment in L8 and 1 segment in L10). If we go back to the old Table 1 and look at individuals L4 and L6, we will see that there are indeed 6 cases when the unambiguous haplotypic counterpart of one of haplotypes in L4 was found in L6 (L6-L7 (2), L6-L8 (1), L6-L11 (3)). That is, L4 is clustered with L6 with respect to one of the two haplotypes in these segments. However, the pattern of incongruence L4-L6 given in the (old) table S12 would refer to a situation when L4 is closely related to some individual IndX based on one haplotype, and L6 is closely related to the same individual IndX based on the other haplotype, however L4 and L6 are not particularly close. As it were, there are no segments satisfying these conditions, so the corresponding number in Table S12 is zero.

There is no contradiction between old Tables 1 and 12 as we do not consider cases when we cannot identify unambiguous haplotypic counterparts (e.g. more than two haplotypes are clustered together). Because of this, cases when L4 shares a segment with L6 (6 cases listed in the first line of Table 1) do not contribute to the pattern of incongruence L4-L6 listed in Table S12. Numbers in revised Table S17 changed relative to old Table S12, as according to the Reviewer's suggestion some phased segments were filtered out in the revised analysis to exclude regions with close paralogs.

Reviewers' comments:

Reviewer #1 (Remarks to the Author):

In this revised manuscript, the authors have added a number of new analyses and quality controls, and well extended the discussion. I appreciate the care the authors have taken with their revisions. The manuscript is improved in many ways. The presentation of the revised manuscript helps clarify and strengthen the conclusions.

However, I am still not convinced by the explanation of lacking of local examples.I have previously requested that the authors should show schematic examples of how recombination and genetic exchanges occurred locally. In contrast to what the authors explained, I don't think presenting local examples would deflect the reader's attention away from the whole genome signatures; instead, it would demonstrate the power of genome-wide analyses and help convince the general audience. Actually, most genome-wide studies I've read do include local examples which reciprocally strengthen the finding of global signatures. It's unclear to me why the presentation of recombination places has to rely on phasing SNPs in all 8 individuals simultaneously. I further note that the title of this manuscript is "recombination in a nature population of the bdelloid rotifer". Such a topic means that the corresponding study should not only focus on the existence of recombination but also the signatures of recombination such as where it distributed across the genome and how it occurred (any hotspots?). Otherwise, I think a more specified title, such as "Genomic signatures reveal the existence of recombination in a natural population of the bdelloid rotifer", would be more appropriate.

Comments to the responses to referee#2
As requested by the editor, I have gone through the comments of the second referee and the associated responses. I acknowledge that the authors have carefully addressed all raised concerns by this referee and made most requested revisions. The only remained issue is the suggestion of adding population genomic statistics to address selection. Although I understand that the authors were inclined to confine to the existence of recombination, I agree with this referee that it would be useful to extend the scope of this study, such as the adaptive evolution of such a largely asexual species and the occurrence of recombination in the context of selection.

Reviewer #3 (Remarks to the Author):

The authors have worked extremely hard to address most comments from the first version with substantial new analyses: I have no further comments on phasing, assembly errors, index-hopping, these have been robustly dealt with. The clarity of the manuscript has also improved, especially the earlier sections, and the authors have now applied greatly improved rigour to evaluating alternative explanations for the population genetic signatures, laid out in a more logical fashion – with just a few parts that still lack clarity. The final summary figure/table is very useful. There are interesting new sections to evaluate meiosis versus other mechanisms. I had very much wished to avoid further recommendations after the considerable efforts the authors have already expended, but there are a few critical points that still need addressing, some relating to new material in this draft.

1) The gene tree comparisons in line 354-366 and supplementary note 9 are puzzling and seem to contradict the main conclusions of the manuscript. As I understand it, you pulled out cases where the relationships of haplotypes A and B in individuals 1 and 2 were ((A1,A2),(B1,B2)), and then found a positive correlation between A1 to A2 divergences and the B1 to B2 divergences. This is used to rule out Oenanthera-type meiosis.

But the same evidence rules out the new hypothesis of regular meiosis, doesn't it? The correlation implies joint inheritance of both haplotypes (as stated by the authors in their previous draft), which is not expected with independent assortment under the rates of meiosis proposed here. With meiosis there should be equal chance of observing ((A1,B1),(A2,B2)) and no tendency for there to even be 'counterparts' between individuals. Instead, a positive correlation between distances and the fact that so many alleles can even be paired across animals seems to be consistent with clonal inheritance. This evidence doesn't seem consistent with your estimates for the frequency of interindividual exchange.

2) There are also a few features that are odd and unexplained about the hybridisation story:
A) Why have L1 to L3 retained copies from both divergent ancestors at every locus? Does this imply they are F1 hybrids with no further meiosis since the hybridisation? In fact, this pattern is weirdly reminiscent of the Signorovitch et al. finding, where one haplotype set across loci is behaving separately from the other haplotype set (in their case, without any evidence or suggestion of hybridisation).
B) L1 has a very divergent mitochondrial haplotype from L2, L3 and L4-L11 – sufficiently divergent that it groups with separate cryptic species within A. vaga according to cox1 variation. Although this is not stated anywhere, it is apparent in Supplementary figures 1 to 3. These data are not explicable with a single hybridisation event, but would require at least 2 – e.g. species A and B hybridised at least twice, one time with mtDNA retained from species A and one time with mtDNA from species B. Some reference to the actual pattern of mtDNA variation (which is glossed over a bit) and how it relates to the hybridisation story would be useful.
C) Is hybridisation really the explanation for this? I wonder because the two copies in individuals L4-L11 also seem to be more divergent than expected by chance in a population with levels of meiosis proposed here – not quite as strikingly as L1 to L3, but still apparent. This seems at odds with the Hardy-Weinberg result based on SNPs (see point expanded below).

3) Lines 170 - Line 216 – the text lost clarity of flow here. I wondered if a section heading for this part would help, e.g. 'signatures of reciprocal recombination'. Some further suggestions for improving clarity of this section:
- line 171, just say without reciprocal recombination here – as that is the part focused on in coming section.
- The point that gene conversion can happen in both meiosis and mitosis is made in 3 different ways, which is confusing. Key points seem to be "Even in the absence of phasing artifacts, LD decay could potentially arise without reciprocal recombination through gene conversion. Gene conversion leads to copying of a DNA segment from one homologous chromosome onto the other; importantly, it can result from resolution of both crossover and non-crossover events35. Gene conversion has been previously proposed to inflate the rate of LD decay between tightly linked loci in humans38,39, and it has been suggested to act within diploid loci of a single A. vaga individual11."
- line 182 Suggest reversing the sentence to say something like "we tested for reciprocal recombination using the classical 4-gamete test".
- line 191. "while such a pair can obviously arise through recombination involving genetic exchanges between individuals" This is confusing here, because then in line 218 you say could still be within individual recombination. Better around line 191 to say such a pair can arise through reciprocal recombination between strands from either the same or different individuals. Otherwise reader gets lost what has been excluded by this point.
- line 216. Insert word "reciprocal" to emphasise finding of the preceding part of the results.

4) The analyses are so comprehensive in their attempts to filter out uncertain data and consider alternative scenarios that it is a little hard to visualise what we're left with. There's a nagging worry that some of the filtering has selected for particular patterns – as mentioned in the authors' response letter where it is stated the over-dispersion of intra-individual allele pairs might be because they require heterozygosity to distinguish haplotypes. For this reason, and other concerns expressed here, I strongly recommend more cautious language for your grand conclusions. This caution seems justified by the fact that your conclusions have shifted considerably from the first draft, despite relying on the

same data!

Minor comments:

Line 16: I would recommend replacing the word 'show' with 'present evidence', given the construction of the arguments, nature of the inferences and puzzling/inconsistent nature of some of the evidence presented, notably the gene tree comparisons.

Lines 21, 60: I am not sure about the term 'compelling evidence'. Perhaps so for intraindividual recombination, but the arguments for interindividual exchange are characterised more cautiously elsewhere (lines 258-260), and the strength of evidence for the two different elements of the manuscript should not be conflated. Perhaps just present the evidence and let readers judge how compelling they find it.

Line 25 "Sexual reproduction, which involves alternation of meiosis and syngamy, is the ancestral condition of extant eukaryotes" Add commas.

Line 68. According to your cox1 phylogenies, the L2-L11 group is a single cluster, whereas L1 is divergent and its mtDNA derives from a separate cryptic species (contrary to your claims in the response to reviewers' comments). This contrasts with the inference from nuclear data, which indicates L1-L3 belong to a separate cluster from L4-L11. Should mention this somewhere in the main text as it is highly relevant for your hybridisation part

Line 74. "through the presence of **% of nearly universal single-copy". Suggest adding summary of percentage into this sentence to help readers unfamiliar with other papers.

Line 79 "revealed the same patterns of genomic structure as…" not true, as no palindromes here, which was a highlight result on genome structure in the original A.vaga. The distinction is important as that was previously reported as evidence against meiosis.

Line 84 – collapse to previous paragraph?

Line 115 – suggest inserting in brackets brief half sentence to describe the nature of the filtering.

Line 225. As mentioned above, the Hardy-Weinberg results seem to be contradicted by the gene tree patterns in figures 5 and 6, where the two alleles within an individual seem to be divergent on the gene trees and never very closely related even in the L4-L11 individuals: alleles in different individuals are identical in 16 cases across the four gene trees shown, but there are no cases of identical alleles in the same individual and very few cases of alleles in the same individual belonging to the same subclade on the tree (the only exception 4.1 and 4.2 in fig 5a). I suspect that the degree of allelic divergence within individuals would come out as significantly higher than expected under a model with the degree of sexual reproduction estimated based on other signatures.

Line 295 – this is not a strong, direct test of contamination as it assumes that contamination only affected a few samples – what if all of the samples were contaminated? Stronger tests of contamination would be to search for reads of contrary mtDNA or nuclear haplotypes within samples, or to search for evidence of tri-allelic SNPs among reads within the same individual. Wording "rejects contamination" is too strong for what is reported here. I'd recommend more cautious wording.

Table 1. The number of incongruent segments seems quite a low fraction of the total (in total 52 cases on 303 phased regions), I wondered whether this was because there was insufficient bootstrap support to judge congruence/incongruence for most cases, or whether there were a large number of

cases that support congruence. What do most gene trees look like? Is there a dominant underlying topology across regions or is it shuffled?

Line 306: "Our data demonstrate that variation in A. vaga was shaped by genetic exchanges between individuals and recombination." Again, more cautious language is recommended to avoid conflating two different phenomena with different bases of evidence: "Our data demonstrate that variation in A. vaga was shaped by recombination, and is consistent with genetic exchanges between individuals".

Line 354-366 and supplementary note 9. The explanation of this analysis is repeated in several places but is difficult to follow each time. This could be resolved by adding a diagram showing the different nested entities that are being compared (pairs of animals, allele pairs within an animal, haplotype pairs across animals), and stating how many instances of each comparison comprise the dataset. I think the comparison of haplotype pairs might be better illustrated by sketching a four-allele tree and indicating which distances are contrasted.

Line 440. "males and meiosis must exist" – history makes me nervous of such strong statements in bdelloids based on analyses of genetic variation! At the very least, the possibility of hermaphrodites cannot be excluded, hence concluding that males exist seems to go well beyond the immediate data. The previous draft of this manuscript highlighted the "near-certain lack of conventional meiosis", whereas the new interpretation uses the same data to argue that "males and meiosis must exist". This is quite a reversal and illustrates the risks when assigning confidence to indirect inferences.

Line 459. "but the very fact that these processes regularly occur in this species has been firmly established". Again, I am nervous of such firm conclusions, having seen firm conclusions about bdelloid rotifers on several occasions turn out to be wrong. I think you would be safer saying that your analyses of genetic variation are consistent with regular inter-individual gene exchange. As a minimum, "firmly established" would seem to me to require direct observation of meiosis or its products, and that is still lacking at this point.

Supplementary Note 6:

"Intriguingly, the observed to expected ratios for the numbers of triallelic sites represented by three heterozygotes were higher when the individuals L4-L11 forming the large cluster were analyzed separately than when all the individuals L1-L11 were analyzed together (Supplementary Table 15). The observed to expected ratios ranged from 12.6 to 13.3 for the individuals L1-L11 and from 22.0 to 28.1 for the individuals L4-L11 (Supplementary Table 15). More frequent genetic exchanges between genetically more similar individuals could explain this difference."

How does this inference match with the interpretation of L1-L3 as hybrids? It does not seem informative to pool all 11 lineages in population genetic analyses when three of them are hypothetical hybrids with greatly elevated heterozygosity.

Tim Barraclough

We thank the two reviewers for the thoughtful reading and many insightful comments on the manuscript. We have now revised the manuscript accordingly, adding new analyses, and hope that it was improved.

Most importantly, as suggested by Reviewer 3, we have substantially expanded the analysis of mitochondrial variation. As suggested by Reviewer 1, we have added a figure with examples of recombination events. We have also introduced smaller refinements. In particular, we have reimplemented the simulation, obtaining more robust estimates of F_{IS} expected under different rates of clonal reproduction. We now also revise our genotype filtering for invariant sites, removing potential biases that could arise from the procedure used in the previous version. These refinements have not affected our conclusions.

Reviewers' comments:

Reviewer #1 (Remarks to the Author):

In this revised manuscript, the authors have added a number of new analyses and quality controls, and well extended the discussion. I appreciate the care the authors have taken with their revisions. The manuscript is improved in many ways. The presentation of the revised manuscript helps clarify and strengthen the conclusions.

However, I am still not convinced by the explanation of lacking of local examples.I have previously requested that the authors should show schematic examples of how recombination and genetic exchanges occurred locally. In contrast to what the authors explained, I don't think presenting local examples would deflect the reader's attention away from the whole genome signatures; instead, it would demonstrate the power of genome-wide analyses and help convince the general audience. Actually, most genome-wide studies I've read do include local examples which reciprocally strengthen the finding of global signatures. It's unclear to me why the presentation of recombination places has to rely on phasing SNPs in all 8 individuals simultaneously. I further note that the title of this manuscript is "recombination in a nature population of the bdelloid rotifer". Such a topic means that the corresponding study should not only focus on the existence of recombination but also the signatures of recombination such as where it distributed across the genome and how it occurred (any hotspots?). Otherwise, I think a more specified title, such as "Genomic signatures reveal the existence of recombination in a natural population of the bdelloid rotifer", would be more appropriate.

We thank the Reviewer for this comment. As suggested, we now added a figure (Fig. 2) showing three schematic examples of recombination. Also as suggested by the Reviewer, for this analysis we required SNPs to be phased just in 4 out of the 8 individuals (we picked L6, L7, L8 and L9, as they had slightly better phasing statistics than L4,

L5, L10 and L11). This allowed to slightly increase the span of phased blocks. To minimize the probability of recombination signal being driven by phasing errors, we based this analysis on the very stringently filtered phased dataset 2 where phasing error rate was assessed to be very low (Supplementary Note 2).

The new figure (Fig. 2) shows three local examples of phased segments demonstrating evidence of recombination. The three corresponding genomic regions all come from different contigs. Presence of recombination in these regions was inferred with RDP4 with different methods; only events supported by at least three out of six different methods were included. We visually checked the evidence for recombination in RDP4 and the alignments of reads to ensure that the signal of recombination is not due to alignment artifacts. Genomic coordinates and other related information (including RDP4 P values) for shown segments are provided in new Supplementary Table 11.

We agree that the original title of our manuscript probably would imply a more detailed survey of recombination features in bdelloids. Therefore, we now thankfully borrow the more specific title proposed by the Reviewer: "Genomic signatures reveal the existence of recombination in a natural population of the bdelloid rotifer *Adineta vaga*".

Still, we do include some analysis of characteristics of recombination. In particular, we test whether recombination tends to happen in regions with high GC-content (Supplementary Note 4). Contrary to our expectations, we found that recombination is more likely to occur in GC-poor regions of the *A. vaga* genome. While a similar pattern has been described in some organisms (e.g. in *Arabidopsis thaliana*; https://elifesciences.org/articles/01426), the opposite trend attributed to GC-biased gene conversion is more common. We now discuss this discrepancy (Supplementary Note 4).

Comments to the responses to referee#2
As requested by the editor, I have gone through the comments of the second referee and the associated responses. I acknowledge that the authors have carefully addressed all raised concerns by this referee and made most requested revisions. The only remained issue is the suggestion of adding population genomic statistics to address selection. Although I understand that the authors were inclined to confine to the existence of recombination, I agree with this referee that it would be useful to extend the scope of this study, such as the adaptive evolution of such a largely asexual species and the occurrence of recombination in the context of selection.

As mutually agreed upon in our correspondence with the Editor, a detailed study of selection goes beyond the scope of this manuscript and merits a separate paper as it would require analyses comparable in length to those presented here. Having said that, our manuscript does include basic whole-genome estimates of selection based on heterozygosity at four-fold nonsynonymous and synonymous sites. In the current revision, we have revised this analysis as follows. We

noticed that the original filtering we used based on the QUAL field (QUAL≥50) removed disproportionally more invariant sites compared to variable sites. While most of our analyses are concerned only with genotype calls at variable sites (SNPs) and are not affected by this effect, this bias could slightly inflate our estimates of heterozygosity; besides, it could also affect interindividual distances. To address this, we have now obtained an additional version of the SNP dataset, called dataset III, which was subjected to less stringent QUAL filtering and therefore had similar proportions of variable and invariant sites retained (Supplementary Methods VII). We have used this dataset in the two analyses involving genotype calls at invariant sites: estimating heterozygosity and computing interindividual distances (see Main Text, Supplementary Methods VII and Supplementary Tables 8-9). As our original filtering was not biased with respect to site synonymicity, the average ratios of heterozygosity at nonsynonymous and synonymous sites remained unchanged: 0.30 for the individuals from the small cluster, and 0.28 for the large cluster.

Reviewer #3 (Remarks to the Author):

The authors have worked extremely hard to address most comments from the first version with substantial new analyses: I have no further comments on phasing, assembly errors, index-hopping, these have been robustly dealt with. The clarity of the manuscript has also improved, especially the earlier sections, and the authors have now applied greatly improved rigour to evaluating alternative explanations for the population genetic signatures, laid out in a more logical fashion – with just a few parts that still lack clarity. The final summary figure/table is very useful. There are interesting new sections to evaluate meiosis versus other mechanisms. I had very much wished to avoid further recommendations after the considerable efforts the authors have already expended, but there are a few critical points that still need addressing, some relating to new material in this draft.

1) The gene tree comparisons in line 354-366 and supplementary note 9 are puzzling and seem to contradict the main conclusions of the manuscript. As I understand it, you pulled out cases where the relationships of haplotypes A and B in individuals 1 and 2 were ((A1,A2),(B1,B2)), and then found a positive correlation between A1 to A2 divergences and the B1 to B2 divergences. This is used to rule out Oenanthera-type meiosis.
But the same evidence rules out the new hypothesis of regular meiosis, doesn't it? The correlation implies joint inheritance of both haplotypes (as stated by the authors in their previous draft), which is not expected with independent assortment under the rates of meiosis proposed here. With meiosis there should be equal chance of observing ((A1,B1),(A2,B2)) and no tendency for there to even be 'counterparts' between individuals. Instead, a positive correlation between distances and the fact that so many alleles can even be paired across animals seems to be consistent with clonal inheritance. This evidence doesn't seem consistent with your estimates for the frequency of interindividual exchange.

Thank you for this insightful comment. It led us to reexamine this analysis in the context of evidence for meiosis provided by genomes L1-L3, which we haven't done in the first revision. The analysis in question was designed under the assumption of a predominantly clonal reproduction with occasional HGT events, which was our main hypothesis (most likely incorrect, as we now know) before we obtained evidence for putative hybridization in L1-L3. We have now reconsidered this analysis and believe that it has several caveats making its results difficult for interpretation. First, as you mention, this analysis indeed selected cases where we were able to identify haplotypic 'counterparts' in two individuals. However, as you correctly point out, with meiosis we would not expect to find haplotypic counterparts at the majority of loci. Indeed, for each pair of analyzed individuals, 'counterparts' were found only for a relatively small proportion of analyzed segments. E.g. out of the 457 phased segments used in this analysis, 'counterparts' were identified for 90 segments in L4-L8, 101 segments in L6-L10, 84 segments in L8-L11, etc. These numbers were provided in Supplementary Table 20 of the previous version of the manuscript. The analysis of the overall correlation involving 444 phased segments was carried out on the pooled set of segments: specifically, we used all segments with haplotypic 'counterparts' identified at least in one pair of individuals (this procedure was detailed in the Supplementary Note 9 of the previous version of the manuscript).

For any given pair of individuals, most loci do not have identifiable 'counterparts', and it is indeed possible that those cases where we are able to find 'counterparts' are just by mere chance. Moreover, in the analysis included in the previous version of the manuscript, when pairing haplotypes, we did not require a haplotype from individual 1 to be more closely related to its closest counterpart in the individual 2 than to the other haplotype of individual 1. In the revision, we repeated the analysis imposing this additional requirement. As a result, the numbers of segments where 'counterparts' can be identified for a pair of individuals dropped even further (e.g. for L4-L8, L6-L10 and L8-L11 the corresponding numbers became 75, 82 and 61 segment respectively). The fact that haplotypes cannot be paired at the majority of loci between two individuals is consistent with thorough randomization of the genome evident in other analyses. We admit that as our null hypothesis in this analysis was obligate asexuality, our inference here most likely was incorrect: while it is reasonable to expect 'counterparts' under asexuality, it does not make much sense to look for 'counterparts' if there is regular genetic exchange. Moreover, as we now realize, the observed correlation between distances can stem not only from joint inheritance of haplotypes, but also from variation in mutation rates or strength of selection between genomic segments. Therefore, the results of this analysis are difficult to interpret. As such, we no longer include this analysis in the manuscript.

2) There are also a few features that are odd and unexplained about the hybridisation story:
A) Why have L1 to L3 retained copies from both divergent ancestors at every locus? Does this imply they are F1 hybrids with no further meiosis since the hybridisation? In fact, this pattern is weirdly reminiscent of the Signorovitch et al. finding, where one haplotype set across loci is behaving separately from the other haplotype set (in their case, without any evidence or suggestion of hybridisation).

Indeed, it appears that the patterns observed in L1-L3 are consistent with them being F1 hybrids. An important caveat is that we were only able to reconstruct the haplotypes in all 11 individuals, L1-L11, for a small fraction of the genome (152 segments from 138 contigs spanning a total of 114,592 base pairs); nevertheless, when heterozygosity is assessed across the whole genome from unphased data, it is still significantly higher in L1-L3 than in L4-L11, and uniformly so across the genome (Supplementary Fig. 8), further supporting the status of L1-L3 as F1 hybrids. Also, observation of putative F1 hybrids without any evidence for subsequent meiosis in rarely-sexual species is not unheard of; a similar pattern has been observed in *Trichoplax* (https://www.nature.com/articles/s41598-018-29400-y). (As a side note, the observations of Fig. 6 and Supplementary Table 28 probably require more than one hybridization event, as suggested by the Reviewer and consistently with the results on mitochondria; see the next comment).

While our results may seem reminiscent of those of Signorovitch et al., there are two important differences. First, Signorovitch et al. described the same pattern of incongruence across all loci they analyzed. This could probably be explained by the small number of loci studied in that work (N=3), as the authors later suggested (personal communication). By contrast, we detect different patterns of incongruence between genomic regions, ruling out *Oenothera*-like meiosis.

Second, our argument for putative hybridization relies on the observation of two subpopulations with major difference in heterozygosity levels between the two clusters. By contrast, no such observation was made in Signorovitch et al. The magnitude of heterozygosity in L1-L3 is within the range previously reported for bdelloids. Therefore, without a joint analysis of L1-L3 and L4-L11, no signature of hybridization could be detected. If we had by chance only sampled individuals from the small cluster (L1-L3) or from the large cluster (L4-L11), we would not have had evidence for hybridization.

Still, we believe that our evidence for L1-L3 as F1 hybrids is not bullet-proof, and write about it cautiously. To support this inference more robustly, one would need to assemble haplotypes at more loci.

B) L1 has a very divergent mitochondrial haplotype from L2, L3 and L4-L11 – sufficiently divergent that it groups with separate cryptic species within A. vaga according to cox1 variation. Although this is not stated anywhere, it is

apparent in Supplementary figures 1 to 3. These data are not explicable with a single hybridisation event, but would require at least 2 – e.g. species A and B hybridised at least twice, one time with mtDNA retained from species A and one time with mtDNA from species B. Some reference to the actual pattern of mtDNA variation (which is glossed over a bit) and how it relates to the hybridisation story would be useful.

Thank you for this important suggestion, which motivated us to significantly expand the analysis and discussion of mitochondrial variation among L1-L11: see the newly added Supplementary Notes 7 and 8 and Supplementary Tables 18-23.

Specifically, we now add the analysis of complete mitochondrial genomes. To make this analysis robust, we implemented stringent quality control. First, we needed to ensure that the high divergence of the mitochondrial haplotype of L1 is not an artifact of genome assembly or contamination. To this end, we blasted the mitochondrial contigs of L2-L11 onto the L1 assembly. If the mitochondrial haplotype of L1 was in fact close to L2-L11, and the high divergence of L1 was artefactual, we would observe some high-similarity hits between L2-L11 and L1. However, in all cases, the best blast hit to the L2-L11 contig in L1 had much lower identity than between the L2-L11 haplotypes. Reciprocally, we also blasted the mitochondrial contigs of L1 onto the assemblies obtained for L2-L11 from HiSeq reads, and observed no high-identity hits.

Second, we checked that sequencing data for L1-L11 are consistent with the presence of a single dominant mitochondrial haplotype within each culture and do not reveal significant mitochondrial heterogeneity expected in the case of cross-culture contamination (this analysis was done using Mutect2). See the newly added Supplementary Notes 7 and 8 and Supplementary Tables 18-23 for details.

We observed that the L1 mitochondrial haplotype is highly divergent along the whole length of the mitochondrial genome. Therefore, this property of L1 is not restricted to the cox1 segment, rendering strong support to its distinct ancestry.

In total, we now believe that the presence of a divergent mitochondrial haplotype in L1 is robust enough to be mentioned among evidence for interindividual genetic exchange. The exact mechanism of this discrepancy is more dubious. While one of the possible interpretations is mitochondrial introgression, we agree with the Reviewer that two reciprocal hybridization events appear more plausible. The data on divergence between L1 and the remaining individuals seem to indirectly support this scenario. If L1-L3 are indeed F1 hybrids, then the extent of nuclear divergence between the two hybridizing populations can be estimated from the level of intraindividual heterozygosity in these individuals, which turned out to be ~2% (Supplementary Table 9). The mitochondrial mutation rate and divergence usually exceed those in the nucleus by a factor of 2 to 6 in invertebrates, and >20 in many vertebrates (e.g. https://academic.oup.com/mbe/article/34/11/2762/3976052). Therefore, if

L1 carried a mitochondrial genome inherited from the second population involved in the putative hybridization, we would expect its mitochondrial divergence from L2-L11 to be much higher than 2%. We found that the L1 mitochondrial haplotype is ~9% different from L2-L11, which is consistent with the reciprocal hybridization scenario. This is now discussed in Supplementary Note 7.

We now have added relevant discussion to the main text.

C) Is hybridisation really the explanation for this? I wonder because the two copies in individuals L4-L11 also seem to be more divergent than expected by chance in a population with levels of meiosis proposed here – not quite as strikingly as L1 to L3, but still apparent. This seems at odds with the Hardy-Weinberg result based on SNPs (see point expanded below).

Intra-individual allelic divergence is indeed higher in the phased segments compared to the baseline genome level. For example, while the whole-genome proportion of heterozygous sites among all called sites in L1 is 0.020 (Supplementary Table 9), the median proportion of heterozygous sites among the 152 phased segments used to analyze haplotype phylogenies in L1-L11 is 0.028. This is not surprising given that, as we discussed in the previous rebuttal letter, the method employed to phase haplotypes indeed requires high heterozygosity, leading to an unavoidable ascertainment bias. We believe that this explains the discrepancy with the Hardy-Weinberg results. Our argument for hybridization, however, relies mainly on the phylogenetic clustering of L1-L3 haplotypes rather than the genetic distances (Fig. 6).

3) Lines 170 - Line 216 – the text lost clarity of flow here. I wondered if a section heading for this part would help, e.g. 'signatures of reciprocal recombination'. Some further suggestions for improving clarity of this section:
- line 171, just say without reciprocal recombination here – as that is the part focused on in coming section.

Thank you, we have added the suggested section heading and removed reference to genetic exchanges at this point.

- The point that gene conversion can happen in both meiosis and mitosis is made in 3 different ways, which is confusing. Key points seem to be "Even in the absence of phasing artifacts, LD decay could potentially arise without reciprocal recombination through gene conversion. Gene conversion leads to copying of a DNA segment from one homologous chromosome onto the other; importantly, it can result from resolution of both crossover and non-crossover events35. Gene conversion has been previously proposed to inflate the rate of LD decay between tightly linked loci in humans38,39, and it has been suggested to act within diploid loci of a single A. vaga individual11."

We agree, and gratefully employ your suggestion for clarifying this section.

- line 182 Suggest reversing the sentence to say something like “we tested for reciprocal recombination using the classical 4-gamete test”.

In the light of other edits, we have added an introductory sentence to this section (“To systematically check whether the distribution of SNPs across haplotypes could be ascribed solely to the action of gene conversion, we employed a modified version of the Hudson's four-gamete test”).

- line 191. “while such a pair can obviously arise through recombination involving genetic exchanges between individuals” This is confusing here, because then in line 218 you say could still be within individual recombination. Better around line 191 to say such a pair can arise through reciprocal recombination between strands from either the same or different individuals. Otherwise reader gets lost what has been excluded by this point.

We removed the words ‘involving genetic exchanges between individuals’ and added ‘reciprocal’ in front of ‘recombination’. Now this phrase reads as ‘while such a pair can obviously arise through reciprocal recombination’. Technically, meiotic recombination also happens between strands from the same individual (which belonged to different individuals in the previous generation, when they resided in the genome of parents of the considered individual). So, probably, introducing such a distinction could also be misleading.

- line 216. Insert word “reciprocal” to emphasise finding of the preceding part of the results.

Done.

4) The analyses are so comprehensive in their attempts to filter out uncertain data and consider alternative scenarios that it is a little hard to visualise what we’re left with. There’s a nagging worry that some of the filtering has selected for particular patterns – as mentioned in the authors’ response letter where it is stated the over-dispersion of intra-individual allele pairs might be because they require heterozygosity to distinguish haplotypes. For this reason, and other concerns expressed here, I strongly recommend more cautious language for your grand conclusions. This caution seems justified by the fact that your conclusions have shifted considerably from the first draft, despite relying on the same data!

Thank you for this comment. As you suggest, we have edited the text implementing more cautious wording. We believe however that parts of our analysis are fully reliable, e.g. the LD decay; and that the data taken together is totally inconsistent with clonal reproduction, which is our main conclusion.

Minor comments:

Line 16: I would recommend replacing the word ‘show’ with ‘present evidence’, given the construction of the arguments, nature of the inferences and puzzling/inconsistent nature of some of the evidence presented, notably the gene tree comparisons.

Changed as suggested.

Lines 21, 60: I am not sure about the term ‘compelling evidence’. Perhaps so for intraindividual recombination, but the arguments for interindividual exchange are characterised more cautiously elsewhere (lines 258-260), and the strength of evidence for the two different elements of the manuscript should not be conflated. Perhaps just present the evidence and let readers judge how compelling they find it.

According to your suggestion, we have removed the word ‘compelling’ from the corresponding sentences.

Line 25 “Sexual reproduction, which involves alternation of meiosis and syngamy, is the ancestral condition of extant eukaryotes” Add commas.

Corrected.

Line 68. According to your cox1 phylogenies, the L2-L11 group is a single cluster, whereas L1 is divergent and its mtDNA derives from a separate cryptic species (contrary to your claims in the response to reviewers’ comments). This contrasts with the inference from nuclear data, which indicates L1-L3 belong to a separate cluster from L4-L11. Should mention this somewhere in the main text as it is highly relevant for your hybridisation part

We have thoroughly revised and expanded the analysis of mitochondrial variation/heterogeneity (this analysis is presented in newly added Supplementary Notes 7 and 8), and mention it in the section on hybridization as suggested.

Line 74. “through the presence of **% of nearly universal single-copy”. Suggest adding summary of percentage into this sentence to help readers unfamiliar with other papers.

Thank you, we have now added a summary of relevant data.

Line 79 “revealed the same patterns of genomic structure as…” not true, as no palindromes here, which was a highlight result on genome structure in the original A.vaga. The distinction is important as that was previously reported as evidence against meiosis.

At this point of the original text, we referred to signatures of tetraploidy evident through the distribution of Ks/percentage of collinear genes among homologous blocks. We agree that the original wording was misleading as it could create an impression that we detected palindromes as well. To clarify this part of the text, we have now replaced words “revealed the same patterns of genomic structure” with “revealed the same patterns of tetraploidy”.

Line 84 – collapse to previous paragraph?

Fixed as suggested.

Line 115 – suggest inserting in brackets brief half sentence to describe the nature of the filtering.

Thank you, we have added a short description of the filtering at this point.

Line 225. As mentioned above, the Hardy-Weinberg results seem to be contradicted by the gene tree patterns in figures 5 and 6, where the two alleles within an individual seem to be divergent on the gene trees and never very closely related even in the L4-L11 individuals: alleles in different individuals are identical in 16 cases across the four gene trees shown, but there are no cases of identical alleles in the same individual and very few cases of alleles in the same individual belonging to the same subclade on the tree (the only exception 4.1 and 4.2 in fig 5a). I suspect that the degree of allelic divergence within individuals would come out as significantly higher than expected under a model with the degree of sexual reproduction estimated based on other signatures.

As discussed above, we are reluctant to interpret the apparent elevated divergence in gene trees as they might be affected by ascertainment bias associated with phasing. This still allows us to search for signatures of genetic exchanges in haplotype phylogenies, as phasing biases cannot lead to clustering of haplotypes from different individuals.

Importantly, the HWE analysis is based on both phased and unphased segments, and the phased segments represent a tiny minority. (As an aside, we now have reimplemented the simulation for the HWE analysis, employing 100 instead of 10 replicates of each simulation. This allowed us to obtain more robust estimates of F_{IS} expected under different rates of clonal reproduction).

Line 295 – this is not a strong, direct test of contamination as it assumes that contamination only affected a few samples – what if all of the samples were contaminated? Stronger tests of contamination would be to search for reads of contrary mtDNA or nuclear haplotypes within samples, or to search for evidence of tri-allelic SNPs among reads within the same individual. Wording

“rejects contamination” is too strong for what is reported here. I’d recommend more cautious wording.

Thank you for this suggestion. Accordingly, we have replaced “rejects contamination” with “argues against contamination”. We agree that if all of the samples were contaminated, this test would not reveal this. However, in this case we would expect to observe significant levels of mitochondrial heterogeneity within individual sequenced samples. We have now carried out the analysis of mitochondrial variation by aligning HiSeq reads from L2-L11 to the mitochondrial contig of L4 and calling mitochondrial single-nucleotide variants with SAMtools/BCFtools and Mutect2 (the details are provided in new Supplementary Notes 7 and 8). Consistently with the expectation of a single dominant mitochondrial haplotype present within each culture, the overwhelming majority of the genotyped sites were found to be homogeneous within each culture L2-L11. Although individuals L2-L11 are very close with respect to mitochondrial haplotypes (nucleotide identity >99%), on average a pair of individuals L2-L11 is different at 57.9 mitochondrial sites, median = 59 sites (Supplementary Table 20). Therefore, if all of the samples were contaminated, we would expect to find a large number of ‘heterozygous’ mitochondrial sites. However, we found only a small number of mitochondrial sites showing evidence for some mitochondrial heterogeneity within a single culture (as detected with Mutect2). In most such cases, the minor-allele fraction variant at such heterogeneous sites was supported by only a small fraction of reads. In total, we found only four well-supported heterogeneous sites (defined as heterogeneous mitochondrial sites with the minor allele fraction variant supported by ≥10% reads) distributed across three individuals (Supplementary Table 22). These results are consistent with low levels of mitochondrial heteroplasmy and argue against contamination of all samples (for details, please see newly added Supplementary Notes 7 and 8).

Regarding the analysis of triallelic sites: for this analysis, we removed all sites with evidence for >2 nucleotides in the aligned reads from any single individual (Supplementary Note 6). Most such cases appear to stem from sequencing errors or index-hopping of very low incidence, as the third nucleotide is usually found only in a single read and normally does not affect genotype calling. Therefore, this filtering may be excessively stringent. In the revision, we recapitulated the result on SNPs with three heterozygotes, carrying out analysis without the above-mentioned filtering. An excess of the observed vs expected number of triallelic SNPs carrying three heterozygotes was virtually the same, whether of not sites with >2 nucleotides in the reads from individual genomes were removed (Supplementary Note 6 and Supplementary Table 16).

In addition, we have significantly expanded the H-score part of the analysis (Supplementary Note 6 and Supplementary Fig. 23). In the previous version of the manuscript, H-scores were computed only for the genotypes of the sequenced individuals L4-L11. Now, we compare the distribution of H-scores in the data to that obtained for datasets with

artificially introduced 'contamination'. Specifically, we have constructed sets of genotypes where genotypes for one of the individuals, L4-L11, at triallelic sites are replaced with genotypes constructed by randomly picking alleles from other individuals, imitating contamination. Such 'contaminated' sets of genotypes indeed exhibit an increase in the variance of H-scores relative to the actual data (Supplementary Note 6 and Supplementary Fig. 23). However, as this analysis also is not a direct test for contamination, we moved it to the Supplementary Information.

Table 1. The number of incongruent segments seems quite a low fraction of the total (in total 52 cases on 303 phased regions), I wondered whether this was because there was insufficient bootstrap support to judge congruence/incongruence for most cases, or whether there were a large number of cases that support congruence. What do most gene trees look like? Is there a dominant underlying topology across regions or is it shuffled?

We now explicitly spell out the breakdown of these 303 segments (see revised Methods). In brief, for 213 of these segments, we were unable to unambiguously identify the closest counterparts for any of the individuals (so we were unable to make a call on whether the grouping was congruent or not). Among the remaining 90 segments, 79 were found to be incongruent at least in one individual, and in 12 segments we identified congruent groupings of the two haplotypes of the same individual. In 52 of the 79 'incongruent' segments, and 10 of the 12 'congruent' segments, the corresponding groupings received decent bootstrap support (≥70%). Importantly, our ability to identify incongruence using the outlined approach is limited as haplotypes can still be involved in incongruent groupings, even though it is not possible to assign haplotypes to pairs of most closely related neighbors. Therefore, 52 out of the 303 phased regions exhibiting clear incongruent groupings of haplotypes provide a lower bound for a proportion of 'incongruent' segments.

Even those segments showing congruent groupings of the two haplotypes of the same individual exhibited different patterns of such haplotype groupings across segments, so there was no dominant underlying topology**. For example, among the 4 segments with congruent groupings for L4, in the first segment, both haplotypes of L4 were clustered with haplotypes of L5; in the second segment, with haplotypes of L8; in the third segment, with haplotypes of L9; and in the fourth segment, with haplotypes of L10 (Supplementary Table 24). That is, congruence was preserved only within the segment, but not among segments, again arguing against clonality (we now discuss this in newly added Supplementary Discussion 1).**

We have now added an extended version of the Table 1 (Supplementary Table 24), showing the raw as well as bootstrap-filtered numbers of congruent and incongruent segments identified for each individual, L4-L11.

Line 306: “Our data demonstrate that variation in A. vaga was shaped by genetic exchanges between individuals and recombination.” Again, more cautious language is recommended to avoid conflating two different phenomena with different bases of evidence: “Our data demonstrate that variation in A. vaga was shaped by recombination, and is consistent with genetic exchanges between individuals”.

Thank you, we have replaced the original version of this sentence with the version suggested by you.

Line 354-366 and supplementary note 9. The explanation of this analysis is repeated in several places but is difficult to follow each time. This could be resolved by adding a diagram showing the different nested entities that are being compared (pairs of animals, allele pairs within an animal, haplotype pairs across animals), and stating how many instances of each comparison comprise the dataset. I think the comparison of haplotype pairs might be better illustrated by sketching a four-allele tree and indicating which distances are contrasted.

This analysis is now removed (see above).

Line 440. “males and meiosis must exist” – history makes me nervous of such strong statements in bdelloids based on analyses of genetic variation! At the very least, the possibility of hermaphrodites cannot be excluded, hence concluding that males exist seems to go well beyond the immediate data. The previous draft of this manuscript highlighted the “near-certain lack of conventional meiosis”, whereas the new interpretation uses the same data to argue that “males and meiosis must exist”. This is quite a reversal and illustrates the risks when assigning confidence to indirect inferences.

We have replaced the words “males and meiosis must exist” with “this suggests the existence of sexual reproduction”. The next few sentences relate to the lack of evidence for males in bdelloids (“However, sex apparently must occur rather often, every ~10-100 generations, to alone produce the observed rate of LD decay – and it is difficult to imagine that hypothetical bdelloid males were overlooked by generations of zoologists if they are that common.”**).**

Line 459. “but the very fact that these processes regularly occur in this species has been firmly established”. Again, I am nervous of such firm conclusions, having seen firm conclusions about bdelloid rotifers on several occasions turn out to be wrong. I think you would be safer saying that your analyses of genetic variation are consistent with regular inter-individual gene exchange. As a minimum, “firmly established” would seem to me to require direct observation of meiosis or its products, and that is still lacking at this point.

We agree and we have replaced the corresponding sentence with the following one: "In summary, we have to conclude that the mechanisms of interindividual genetic exchanges and recombination in a bdelloid rotifer *A. vaga* remain obscure, but the data on genetic variation strongly suggest regular occurrence of these processes in *A. vaga*."

Supplementary Note 6:

"Intriguingly, the observed to expected ratios for the numbers of triallelic sites represented by three heterozygotes were higher when the individuals L4-L11 forming
the large cluster were analyzed separately than when all the individuals L1-L11 were
analyzed together (Supplementary Table 15). The observed to expected ratios ranged
from 12.6 to 13.3 for the individuals L1-L11 and from 22.0 to 28.1 for the individuals
L4-L11 (Supplementary Table 15). More frequent genetic exchanges between genetically more similar individuals could explain this difference."

How does this inference match with the interpretation of L1-L3 as hybrids? It does not seem informative to pool all 11 lineages in population genetic analyses when three of them are hypothetical hybrids with greatly elevated heterozygosity.

Tim Barraclough

Although an excess of sites harboring three heterozygotes evident in this pooled analysis is still indicative of genetic exchange, we agree that pooling 11 individuals together does not make much sense, given that L1-L3 appear to be hybrids. Indeed, the observed to expected ratios for the numbers of triallelic sites represented by three heterozygotes are not directly comparable between the 8 individuals of the large cluster and all 11 individuals pooled together. According to the suggestion of the Reviewer, we have removed this inference both from the main text and the Supplementary Note 6. Supplementary Table 16 (previously, Supplementary Table 15) showing observed and expected numbers of triallelic sites with three heterozygotes has also been updated: now we only present the numbers pertinent to the analysis of the 8 individuals of the large cluster, L4-L11.

REVIEWERS' COMMENTS:

Reviewer #1 (Remarks to the Author):

The authors have done a great job addressing my previous concerns. I only have a few minor suggestions to improve clarity in newly added Fig. 2. I acknowledge the great efforts to add these local examples that help convince audience with the existence of recombination in the bdelloid rotifer. This figure could be further improved in presentation. The authors might borrow the idea of a previous study (Debortoli et al. Curr Biol 26: 728; Fig. 5) to separate haplotypes along the contig by using colors. Also, please add contig IDs.

Reviewer #3 (Remarks to the Author):

The authors have completed another extensive round of revision. There is lots of work and mostly more careful wording now, which is good given the history in this field of definitive statements later being over-turned. The between-individual recombination part is the key part of interest to most people, but also remains the most uncertain part. I would like to see the manuscript published, with some final modifications addressing the points below, but I still have my doubts that this provides strong evidence for regular inter-individual genetic transfers. I can't provide a simple alternative explanation, but the results do not seem to be explicable simply through sexual reproduction either. With suitably careful wording, however, this manuscript does make an important contribution towards finally working out what's going on in these infuriating animals.

1) The hybrid story, referred to by the authors as the strongest evidence for sex, is rather complex, apparently requiring 2 or 3 hybridization events (see below) but no further interbreeding or HGT since them. I am still left wondering whether some alternative scenario aside from either sex or HGT could explain these differences. Certainly it has turned out to be more complex than simply looking in a population of rotifers and finding incompatible gene trees, and neither sex nor HGT seem like an easy explanation for these data. The authors acknowledge this complexity in their response letter and more so now in the manuscript – in just one or two places it could still be further underlined in the manuscript (listed below).

2) My earlier reviews asked for some overall phylogenetic analysis of nuclear relationships and mitochondrial relationships, to see whether the exemplar trees being shown for filtered regions are indicative of the general pattern. A table of mitochondrial divergences is now shown – I still think it would be easier for most readers to see this as a tree, and this information (with support values) would also confirm whether 2 or 3 hybridisation events are needed to explain the data (see below). An overall tree for the nuclear genome, for example as might be obtained from gene alignments by Orthofinder, is also still lacking. Supplementary table 8 provides some clues – for example L6 and L8 seem to be less divergent than the other lineages in green, and also share a very close relationship in mitochondrial haplotype – would this be expected in a sexually recombining population?

3) The recombination examples in figure 2 are helpful but look like weak evidence relative to the potential power of this approach to show clear cases. These are three cases hand-picked from the whole genome to show recombination, but they are very short fragments and have weak p values (supplementary table 11: case a, 3/6 tests significant with mean p=0.003; case b, 4/6 tests significant with mean p=0.01; case c, mean p=0.02 and 0.013). The figure seems to come too early in the manuscript, and the text is too definitive there ("often reveals visible signatures of recombination" on Line 110, versus "suggestive" on Line 1391). It would fit better where you talk about between-individual recombination and incongruent gene trees.

Specific comments:

Could delete “reveal the existence” from title and have a snappier title.

Abstract – line 18, 19. The wording here implies that all of the signatures mentioned reject clonality, but the manuscript now carefully outlines which ones are indicative of recombination (potentially including intra-individual) versus recombination between individuals. This sentence also contradicts your final sentence of the paper, which says that the mechanism of genetic exchanges remains obscure- i.e. could still be compatible with clonal reproduction + HGT. Please modify the abstract to be consistent with the manuscript.

The abstract doesn’t mention the hybridisation story, which you later state is the strongest evidence for sex in bdelloid rotifers. Suggest to add reference, e.g. something like “including evidence for hybridisation between divergent taxa” to final sentence.

Line 68. “we confirmed the species identity of these individuals using mitochondrial marker-based phylogeny”. Clarify here that this indicated 2 species – as this is important for the hybridisation story later.

The discussion implies there are “two options” now: sex or HGT. Based on the detail of your discussion, response letter, and conclusions, however it is clear that neither HGT nor sex provide a simple, easy explanation for your data. It might be prudent, especially given the history of this field, to add one sentence saying that there might be additional genetic mechanisms or scenarios not considered here that resolve some of these inconsistencies.

Trying to visualise a tree from your mitogenome distance matrix, it looks like L10, a non-hybrid, is nested within a clade containing L2 and L3, i.e. L2 and L3 are separated by a non-hybrid mtDNA haplotype. If so, your data would require 3 hybridisation events – one to generate L1 with the mitogenome of the divergent species; and two to generate L2 and L3, otherwise L10 would also have to be a hybrid (which it isn’t).

Line 103. Why would selection against deleterious mutations be relaxed in A.vaga, if it isn't clonal…?

Line 190: To complement the cited references, a new preprint from March 2020 describes the recombinational landscape of another primarily asexual freshwater invertebrate, Daphnia pulex. One of the key findings might be relevant to this manuscript:
“Contrary to expectations for models in which crossing-over is the primary mechanism of recombination, and consistent with data for other species, the gradient of linkage disequilibrium with increasing physical distance between sites is far too high at short distances and far too low at long distances, suggesting an important role for factors such as the nonindependent appearance of pairs of mutations on haplotypes and long-range gene-conversion-like processes.”

Tim Barraclough

We would like to cordially thank all the three Reviewers who have commented on this manuscript during the course of subsequent revisions for their hard work. We believe that implementing many insightful suggestions of the Reviewers and addressing their concerns has helped to greatly improve the manuscript and make its conclusions more robust. Our responses to the remaining comments are given below.

REVIEWERS' COMMENTS:

Reviewer #1 (Remarks to the Author):

The authors have done a great job addressing my previous concerns. I only have a few minor suggestions to improve clarity in newly added Fig. 2. I acknowledge the great efforts to add these local examples that help convince audience with the existence of recombination in the bdelloid rotifer. This figure could be further improved in presentation. The authors might borrow the idea of a previous study (Debortoli et al. Curr Biol 26: 728; Fig. 5) to separate haplotypes along the contig by using colors. Also, please add contig IDs.

We thank the Reviewer for the high esteem of our work.
We agree with the Reviewer that the idea of Debortoli et al. to separate haplotypes by using colors is a great approach to aid visualizing recombination events. According to the Reviewer's suggestion, in the course of this revision we tried to adopt this approach. However, unlike the relevant Figure from the study by Debortoli et al., which depicts only non-overlapping recombination events, panel c of our Fig. 6 (previously Fig. 2) involves two events that partially overlap. We realized that it is tricky to come up with a clear coloring scheme in such case, where the same genomic region was assigned by RDP4 to more than one recombination event. Because of this we would rather prefer to stick with the original way of presentation. However, we have slightly redesigned the figure and borrowed an element of design used by Debortoli et al. (a symbol of axis break) to show that a displayed fragment is a part of a larger DNA molecule. Following the suggestion of the Reviewer, we have also added contig IDs to Fig. 6.

Reviewer #3 (Remarks to the Author):

The authors have completed another extensive round of revision. There is lots of work and mostly more careful wording now, which is good given the history in this field of definitive statements later being over-turned. The between-individual recombination part is the key part of interest to most people, but also remains the most uncertain part. I would like to see the manuscript published, with some final modifications addressing the points below, but I still have my doubts that this provides strong evidence for regular inter-individual genetic transfers. I can't provide a simple alternative explanation, but the results do not seem to be explicable simply through sexual reproduction either. With suitably careful wording, however, this manuscript does make an

important contribution towards finally working out what's going on in these infuriating animals.

1) The hybrid story, referred to by the authors as the strongest evidence for sex, is rather complex, apparently requiring 2 or 3 hybridization events (see below) but no further interbreeding or HGT since them. I am still left wondering whether some alternative scenario aside from either sex or HGT could explain these differences. Certainly it has turned out to be more complex than simply looking in a population of rotifers and finding incompatible gene trees, and neither sex nor HGT seem like an easy explanation for these data. The authors acknowledge this complexity in their response letter and more so now in the manuscript – in just one or two places it could still be further underlined in the manuscript (listed below).

We now additionally state this in the end of Discussion.

2) My earlier reviews asked for some overall phylogenetic analysis of nuclear relationships and mitochondrial relationships, to see whether the exemplar trees being shown for filtered regions are indicative of the general pattern. A table of mitochondrial divergences is now shown – I still think it would be easier for most readers to see this as a tree, and this information (with support values) would also confirm whether 2 or 3 hybridisation events are needed to explain the data (see below). An overall tree for the nuclear genome, for example as might be obtained from gene alignments by Orthofinder, is also still lacking. Supplementary table 8 provides some clues – for example L6 and L8 seem to be less divergent than the other lineages in green, and also share a very close relationship in mitochondrial haplotype – would this be expected in a sexually recombining population?

Thank you, in addition to the table of mitochondrial divergences, we have now complemented the manuscript with a mitochondrial tree (Supplementary Figs. 27-29). For 10 individuals L2-L11 carrying very similar mitochondrial haplotypes, we were able to reconstruct almost complete mitochondrial sequences (~14 kb, Supplementary Notes 7 and 8) based on variant calls produced against the L4 mitochondrial contig. The presented mitochondrial phylogeny for L2-L11 (Supplementary Fig. 27), was inferred using these nearly complete mitochondrial haplotypes. The mitochondrial haplotype of L1 is too divergent to allow simultaneous variant calling of mitochondrial variants with L2-L11 (Supplementary Notes 7 and 8). As such, to build a mitochondrial tree including all 11 individuals, we took the longest L1 mitochondrial contig (~7 kb, contig8072) and aligned it with the reconstructed mitochondrial haplotypes of L2-L11. Next, we used a portion of the alignment corresponding to the region present in L1 contig8072 (total alignment length = 7,126 bp), to reconstruct mitochondrial phylogeny for all 11 individuals (Supplementary Fig. 28).

Thank you for the suggestion to use Orthofinder, however, in the case of our data, it does not appear to be applicable for purposes of obtaining gene alignments. Orthofinder requires a set of protein sequence files, one file per analyzed species (or individual in our case). This would imply obtaining genome assemblies for each sequenced

individual and annotating genomes of each individual independently. However, we were able to obtain a genome assembly of reasonable quality only for L1 (which is used as reference). The reads for remaining individuals were mapped against the L1 assembly, which allowed to locally reconstruct haplotypes of L2-L11. However, the resulting phased segments are not suitable for carrying out annotation of protein coding genes. Moreover, inferring phylogeny based on orthogroups works well with different species where each gene from each species can be represented by a single sequence. In the case of *A. vaga* individuals L1-L11, both haplotypes need to be taken into account when estimating the phylogeny. Selecting one haplotype out of two at random at each locus probably would not allow to adequately assess phylogenetic relationships between the sequenced individuals.

Still, we have now added an overall neighbor-joining tree for the nuclear genome (Supplementary Fig. 8). This tree is built not from phased haplotypes, but from unphased genotypic data (L1-L11 biallelic SNPs). Specifically, the tree is based on distances calculated as fractions of alleles different between individuals (the corresponding methods description can be found in the section "Analysis of population structure" of Supplementary methods). We agree that this tree is useful, however we note that it does not reflect relationships between haplotypes, only an overall pattern of genetic similarity (some kind of "averaging" across the genome) between the analyzed individuals. Note that here fractions of different alleles are computed for biallelic sites (invariant sites are not included), therefore the resulting distances underlying the neighbor-joining tree (Supplementary Fig. 8) are by construction significantly larger than genotypic distances computed taking invariant sites in consideration (Supplementary Table 7, previously Supplementary Table 8).

Indeed, L6 and L8 are most closely related both with respect to genetic distances computed from unphased nuclear SNPs (Supplementary Fig. 8) and with respect to mitochondrial haplotypes (Supplementary Figs. 27-29). The observation that some individuals are clustered both with regard to nuclear and mitochondrial genomes is not unexpected for a sexually recombining population: assuming the existence of weak population structure may easily explain this. Some other groupings (e.g. positioning of L4 and L10) found in the nuclear tree also resemble those in the mitochondrial tree. And yet, notably, the overall tree for nuclear genome and the mitochondrial tree are largely incongruent. Together with other findings this observation argues for genetic exchange in *A. vaga*.

3) The recombination examples in figure 2 are helpful but look like weak evidence relative to the potential power of this approach to show clear cases. These are three cases hand-picked from the whole genome to show recombination, but they are very short fragments and have weak p values (supplementary table 11: case a, 3/6 tests significant with mean p=0.003; case b, 4/6 tests significant with mean p=0.01; case c, mean p=0.02 and 0.013). The figure seems to come too early in the manuscript, and the text is

too definitive there (“often reveals visible signatures of recombination” on Line 110, versus “suggestive” on Line 1391). It would fit better where you talk about between-individual recombination and incongruent gene trees.

We thank the Reviewer for this comment. We note that the figure showing examples of recombination was added during the course of previous revision in response to the request made by Reviewer 1. As discussed previously, phasing haplotypes from short Illumina reads allows to reconstruct haplotypes only for relatively short fragments. Requiring haplotypes to be simultaneously phased in several individuals results in a further decrease of the span of phased blocks available for the analysis. Nevertheless, following the suggestion of Reviewer 1, we added a figure presenting local examples of recombination to illustrate the general results of the manuscript. These examples (which were manually selected) are used purely for illustrative purposes. Evidence for recombination is inferred from other analyses (LD decay assessed using different approaches) exploiting a much larger dataset. Still, we tend to agree with Reviewer 1 that including local examples is useful to “strengthen the finding of global signatures”. We agree that the figure was placed too early in the previous version of the manuscript. As suggested, we now moved it to the part of the manuscript devoted to between-individual recombination and edited the relevant text implementing less definitive wording.

Specific comments:

Could delete “reveal the existence” from title and have a snappier title.

We thank the Reviewer for this suggestion. We agree that a shorter version of the title (such as “Recombination in a natural population of the bdelloid rotifer *Adineta vaga*” employed for the original version of the manuscript) appears to be more eye-catching. However, in the previous round of revision, we were advised by the Reviewer 1 that such a title implies a more detailed survey of recombination features in *A. vaga* than that carried out in our study. As such we were recommended by Reviewer 1 to change the title to a more specific one. As we agreed with Reviewer 1 that our study does not provide a detailed analysis of recombination properties in bdelloids (such as distribution of hotspots, etc.), in the previous revision we have replaced the original title with the version proposed by Reviewer 1 (“Genomic signatures reveal the existence of recombination in a natural population of the bdelloid rotifer *Adineta vaga*”). Now we delete “reveal the existence” from title employing a “consensus” version of the title.

Abstract – line 18, 19. The wording here implies that all of the signatures mentioned reject clonality, but the manuscript now carefully outlines which ones are indicative of recombination (potentially including intra-individual) versus recombination between individuals. This sentence also contradicts your final sentence of the paper, which says that the mechanism of genetic exchanges remains obscure- i.e. could still be compatible with clonal reproduction + HGT. Please modify the abstract to be consistent with the

manuscript.

Changed to "strict clonality and lack of genetic exchange", as per Fig. 8.

The abstract doesn't mention the hybridisation story, which you later state is the strongest evidence for sex in bdelloid rotifers. Suggest to add reference, e.g. something like "including evidence for hybridisation between divergent taxa" to final sentence.

Added to the abstract, thank you.

Line 68. "we confirmed the species identity of these individuals using mitochondrial marker-based phylogeny". Clarify here that this indicated 2 species – as this is important for the hybridisation story later.

We would rather not claim that the identified lineages are of a species status, as this would require additional evidence. We now specify the existence of two clades here.

The discussion implies there are "two options" now: sex or HGT. Based on the detail of your discussion, response letter, and conclusions, however it is clear that neither HGT nor sex provide a simple, easy explanation for your data. It might be prudent, especially given the history of this field, to add one sentence saying that there might be additional genetic mechanisms or scenarios not considered here that resolve some of these inconsistencies.

We now point out that neither option provides a simple explanation for our data.

Trying to visualise a tree from your mitogenome distance matrix, it looks like L10, a non-hybrid, is nested within a clade containing L2 and L3, i.e. L2 and L3 are separated by a non-hybrid mtDNA haplotype. If so, your data would require 3 hybridisation events – one to generate L1 with the mitogenome of the divergent species; and two to generate L2 and L3, otherwise L10 would also have to be a hybrid (which it isn't).

We agree, and the newly provided mitochondrial phylogeny (Supplementary Figs. 27-29) supports this. We now discuss this in the newly added final part of Supplementary Note 7.

Line 103. Why would selection against deleterious mutations be relaxed in A.vaga, if it isn't clonal…?

This is indeed a very interesting topic that, perhaps, merits a separate study. We note that we haven't carried out a detailed analysis of the strength of purifying selection in *A. vaga.* A more thorough analysis of purifying selection (which is not the focus of our manuscript) is required to further clarify this question. Such analysis should probably include comparison with other rotifer species (e.g. monogononts) and involve assessment of selective constraint in

different species using the same methods to allow direct comparison between datasets. As a side note, there is at least one example of a species with rare sexual reproduction exhibiting relaxed purifying selection – duckweed *Spirodela Polyrhiza* (https://pubmed.ncbi.nlm.nih.gov/31298732/).

Due to space limitations, we moved the relevant text on negative selection from the main text to Supplementary Discussion.

Line 190: To complement the cited references, a new preprint from March 2020 describes the recombinational landscape of another primarily asexual freshwater invertebrate, Daphnia pulex. One of the key findings might be relevant to this manuscript:
"Contrary to expectations for models in which crossing-over is the primary mechanism of recombination, and consistent with data for other species, the gradient of linkage disequilibrium with increasing physical distance between sites is far too high at short distances and far too low at long distances, suggesting an important role for factors such as the nonindependent appearance of pairs of mutations on haplotypes and long-range gene-conversion-like processes."

Tim Barraclough

Thank you, we now cite the preprint on recombination in *D. pulex* and briefly refer to its main findings in the Discussion section.
